# Unmasking the rising global burden of depression: A 32-year GBD analysis of gender disparities and regional hotspots in Sub-Saharan Africa

**DOI:** 10.1371/journal.pone.0326974

**Published:** 2025-07-31

**Authors:** Junping Liu, Zhongming Ye, Yan Cai, Jiaying Li, Zhe Dong, Xiaodan Zhang, Ming Lei

**Affiliations:** 1 Department of Traditional Medicine, Seventh People’s Hospital, Shanghai University of Traditional Chinese Medicine, Shanghai, China; 2 Department of Critical Care Medicine, Seventh People’s Hospital, Shanghai University of Traditional Chinese Medicine, Shanghai, China; National Center for Chronic and Noncommunicable Disease Control and Prevention, Chinese Center for Disease Control and Prevention, CHINA

## Abstract

**Aims:**

Depression, a leading contributor to the global disease burden, exhibits alarming increases in incidence and prevalence, with pronounced disparities across regions and genders. This study provides the first comprehensive analysis of depression burden from 1990 to 2021, integrating the latest Global Burden of Disease (GBD) 2021 data to identify critical hotspots and policy-relevant trends.

**Methods:**

Estimated global, regional, and national burden of disease for depression from 1990–2021 by extracting incidence, prevalence, and DALYS from the Global Burden of Disease(GBD) database 2021.

**Results:**

From 1990 to 2021, the global incidence of depression surged by 15.6% (3,749–4,334 per 100,000), with Sub-Saharan Africa emerging as an unexpected epicenter. Uganda and The Gambia recorded the highest incidence rates globally (9,644 and 7,624 per 100,000, respectively), likely linked to civil instability and healthcare deficits. Women bore a disproportionate burden, with adolescent females (15–19 years) showing 64% higher incidence than males (5,584 vs. 3,401 per 100,000). High-income regions paradoxically exhibited steeper annual percentage increases (EAPC: 1.0 in North America), suggesting improved detection or escalating stressors.

**Conclusions:**

This study highlights urgent priorities: (1) integrating mental health services into primary care in conflict-affected African nations. (2) gender-sensitive interventions targeting adolescent females. (3) global equity in mental health resource allocation.

## Introduction

Depression, as a pervasive mood disorder, has become an important challenge in the field of global public health, and the increasing trend of its incidence and prevalence has attracted widespread attention. According to the World Health Organization, more than 264 million people worldwide suffer from depression, a number that has shown an upward trend over the past few decades [1]. Depression not only affects an individual’s mental health, but can also lead to a range of health problems, including cardiovascular disease, decreased cancer survival, increased suicide rates, and increased cognitive impairment in Alzheimer’s disease [2–4]. In addition, depression places a heavy financial burden on patients and their families, with an average annual direct cost of between $1000 and $2500 per patient [5]. As the global population grows and ages, the disease burden of depression is expected to continue to increase and is expected to be the leading cause of the global disease burden by 2030 [6].

Since the 90s of the 20th century, the incidence and prevalence of depression have increased globally. According to data from the GBD, the global age-standardized incidence of depression declined between 1990 and 2019, but increased in regions with high sociodemographic indices [7]. This phenomenon may be related to social competitive pressure, individualistic culture, and social acceptance of awareness and help-seeking behavior in areas with high SDI [8]. In addition, the incidence of depression varies significantly among ages, periods, and birth cohorts, and these differences may be influenced by a variety of factors, including sociodemographic factors, economic status, and education level [9].

Previous studies on the global burden of depression have been limited in their scope and depth, often focusing on specific time periods or regions and failing to thoroughly analyze long-term trends. Moreover, due to data constraints, these studies have had difficulty in disentangling the independent effects of age, period, and birth cohort on depression onset. To address these gaps and provide a more nuanced understanding of depression’s disease burden, this study leverages the 2021 GBD Study data. We conduct a comprehensive analysis of depression’s incidence, prevalence, and DALYS across global, regional, and national levels from 1990 to 2021, with a particular emphasis on gender and age differences. Unlike prior studies, our approach delves deeper into the intricate interplay of various factors influencing depression burden. By doing so, this study not only enriches the existing knowledge base but also offers valuable insights for developing more effective and targeted prevention and control strategies. It provides a unique perspective on the global distribution of depression burden, which is not fully captured in the GBD document alone. Specifically, our analysis reveals how the burden varies across different socio-demographic groups and geographical regions, shedding light on areas that require urgent attention and resource allocation. This enables policymakers and healthcare professionals to better identify high-risk populations and implement tailored interventions to mitigate the impact of depression on a global scale.

## Methods

### Data sources

This study is based on the GBD 2021 database, which provides detailed epidemiological data on 371 diseases and injuries in 204 countries and territories between 1990 and 2021. The GBD 2021 database aggregates data from numerous sources, including but not limited to, health surveys, medical records, and epidemiological studies conducted in each country. However, the exact sample size or source count for each regional estimate may vary due to differences in data availability and quality across regions.We use standardized epidemiological analysis methods to ensure global comparability of data. These data are freely available through the Global Health Data Exchange website [10], and detailed methods and models have been described in previous reports [11]. Specifically, we extracted data on depressive disorders, including incidence, prevalence, and disability-adjusted life years. Data were accessed and downloaded through the Global Health Data Exchange (GHDx) platform (http://ghdx.healthdata.org/gbd-results-tool).

### Important definitions

The socio-demographic index is a comprehensive measure of the overall economic development of a society, taking into account the level of education, per capita income, and fertility rate [12]. SDI values range from 0 to 1, with higher values reflecting higher levels of social and economic development. Based on the SDI values of the GBD 2021 study, we categorized 204 countries into five SDI groups: High, High middle, Middle, Low middle, and Low. Uncertainty Interval (UI) is an important statistical tool calculated by multiple sampling and correlation matrix when GBD processes international data, taking into account the differences in methods and missing data. It reflects global differences in data collection and processing methods, as well as the reality that data quality is affected by a variety of factors, and is essential for assessing the reliability of data across countries and comparing the results of studies. Confidence intervals (CI) are statistically determined and used to estimate the possible range of population parameters, calculated based on sample data. In the present study, we obtained incidence and prevalence data from the GBD database and used the UI to identify uncertainties in these data to more accurately reflect the reliability of the findings.

### Statistical analysis

Age-Standardized Incidence Rate (ASR) and Estimated Annual Percentage Change (EAPC): Used to quantify trends in the incidence of depression. ASR is the incidence after excluding age. The ASR for depression does not reflect the actual incidence of depression, but is only used to compare the incidence of depression in different countries, regions, or different historical periods in the same region for data comparison. If the age structure of the population in two regions is very different, comparing the incidence rates alone does not reveal whether the high incidence in a given region is due to differences in age composition or other contributing factors. Therefore, it is necessary to standardize the incidence according to age. The methods used to calculate ASR have been previously reported [13]. The Estimated Annual Percentage Change (EAPC) is a statistical measure that describes the average annual percentage change in a rate over a specified time period. A positive EAPC indicates an increasing trend, while a negative EAPC indicates a decreasing trend.R Studio (version 4.4.0) was used for statistical analysis. Statistical significance was defined as P < 0.05 for all bilateral P values.

### Ethical standards

Ethics approval was exempted by the Ethics Committee of Seventh People’s Hospital Affiliated to Shanghai University of Traditional Chinese Medicine because the GBD 2021 study is a publicly available database and all data were anonymous.

## Results

### The burden of depression from a global perspective

The study included a total of 204 countries around the world to assess the incidence, prevalence, and DALYS of depression from 1990 to 2021. Studies have shown an upward trend in the incidence, prevalence, and DALYS of depression globally from 1990 to 2021 (Table 1). The incidence rate increased from 3,748.5 to 4,333.6 per 100,000 people, and the prevalence rate increased from 3,599.7 to 4,006.8 per 100,000 people.

**Table 1:** Age-standardised incidence, prevalence, and DALYS rates for depresssive disorders in 1990 and 2021 and their temporal trends from 1990 to 2021.

Characteristics	Age-standardised incidence rate per 100000 population			Age-standardised prevalence rate per 100000 population			Age-standardised DALYS rate per 100000 population		
	1990 (95% UI)	2021 (95% UI)	EAPC (95% CI)	1990 (95% UI)	2021 (95% UI)	EAPC (95% CI)	1990 (95% UI)	2021 (95% UI)	EAPC (95% CI)
**Global**	3748.490(3292.730 to 4353.004)	4333.617(3770.796 to 5093.614)	−0.005(−0.226 to 0.116)	3599.666(3251.907 to 4023.207)	4006.823(3581.258 to 4539.010)	−0.033(−0.153 to 0.088)	600.517(420.941 to 818.448)	681.142(475.189 to 923.825)	−0.042(−0.187 to 0.103)
**Sex**									
Male	2850.215(2494.733 to 3315.232)	3366.269(2922.760 to 3958.072)	0.000(−0.169 to 0.171)	2833.005(2551.623 to 3163.659)	3186.429(2853.403 to 3604.266)	−0.002(−0.121 to 0.116)	468.059(326.203 to 640.144)	540.509(377.310 to 735.480)	0.005(−0.139 to 0.149)
Female	4634.854(4075.957 to 5384.215)	5295.222(4606.321 to 6227.480)	−0.089(−0.261 to 0.084)	4358.153(3938.968 to 4883.831)	4822.117(4316.381 to 5483.345)	−0.053(−0.176 to 0.070)	731.704(512.353 to 996.697)	821.165(570.959 to 1110.427)	−0.073(−0.219 to 0.074)
**SDI quintile**									
High	3634.819(3252.825 to 4142.460)	5001.702(4364.285 to 5837.960)	0.487(0.287 to 0.687)	3446.858(3133.416 to 3829.183)	4312.343(3857.177 to 4866.007)	0.305(0.158 to 0.453)	584.721(407.230 to 792.382)	766.074(535.992 to 1026.587)	0.388(0.217 to 0.560)
High middle	3536.578(3133.570 to 4046.004)	3761.682(3279.494 to 4405.827)	−0.236(−0.392 to −0.079)	3478.403(3151.801 to 3862.921)	3666.901(3280.975 to 4119.096)	−0.155(−0.255 to −0.054)	575.268(404.608 to 784.668)	610.075(424.534 to 831.086)	−0.187(−0.314 to −0.060)
Middle	3239.599(2849.297 to 3766.397)	3762.030(3282.295 to 4388.678)	−0.019(−0.184 to 0.146)	3321.971(3002.473 to 3712.102)	3654.361(3272.501 to 4075.439)	−0.051(−0.160 to 0.058)	537.881(375.229 to 735.243)	605.910(424.522 to 824.659)	−0.045(−0.180 to 0.091)
Low middle	4743.865(4116.830 to 5567.431)	5151.929(4437.485 to 6092.242)	−0.391(−0.608 to −0.173)	4250.244(3815.691 to 4807.300)	4528.897(4043.408 to 5176.249)	−0.268(−0.424 to −0.112)	726.740(502.501 to 981.115)	784.069(542.466 to 1059.205)	−0.318(−0.505 to −0.131)
Low	5234.246(4495.408 to 6211.367)	5511.851(4713.360 to 6539.423)	−0.268(−0.415 to −0.120)	4664.768(4155.860 to 5346.198)	4849.640(4260.972 to 5554.658)	−0.189(−0.296 to −0.083)	795.503(545.583 to 1072.899)	837.533(569.846 to 1140.075)	−0.202(−0.327 to −0.076)
**GBD region**									
East Asia	2610.704(2296.983 to 2995.191)	2337.696(2058.015 to 2718.381)	−0.556(−0.710 to −0.401)	3059.656(2765.455 to 3392.613)	2870.608(2583.727 to 3205.354)	−0.425(−0.486 to −0.365)	470.811(329.328 to 635.952)	429.669(304.251 to 585.415)	−0.513(−0.607 to −0.419)
Southeast Asia	2255.428(1959.226 to 2659.947)	2646.507(2262.278 to 3152.367)	0.028(−0.156 to 0.212)	2742.546(2435.887 to 3111.360)	2991.564(2647.865 to 3401.761)	0.011(−0.089 to 0.112)	413.358(286.157 to 564.463)	467.628(323.743 to 634.876)	0.033(−0.103 to 0.170)
Oceania	2824.746(2404.361 to 3378.559)	2956.632(2307.715 to 3768.079)	−0.070(−0.130 to −0.010)	3116.257(2709.626 to 3653.805)	3201.990(2654.701 to 3811.000)	−0.043(−0.079 to −0.007)	490.410(337.659 to 672.597)	507.905(329.203 to 704.035)	−0.052(−0.098 to −0.007)
Central Europe	2859.032(2501.424 to 3287.726)	3225.415(2763.062 to 3788.210)	−0.366(−0.627 to −0.104)	2946.109(2624.378 to 3340.061)	3171.900(2789.030 to 3611.516)	−0.240(−0.405 to −0.075)	472.390(328.242 to 642.445)	521.748(358.803 to 710.096)	−0.292(−0.503 to −0.079)
Central Asia	3675.513(3152.032 to 4269.592)	4131.919(3444.954 to 4952.486)	−0.018(−0.166 to 0.131)	3482.871(3058.011 to 3966.884)	3773.707(3243.382 to 4386.790)	−0.017(−0.120 to 0.087)	582.558(401.134 to 795.955)	644.278(441.834 to 883.559)	−0.007(−0.133 to 0.119)
High-income Asia Pacific	2275.579(2028.978 to 2566.129)	2845.861(2452.418 to 3326.646)	0.491(0.333 to 0.650)	2168.624(1975.103 to 2399.341)	2545.156(2266.733 to 2892.377)	0.298(0.175 to 0.421)	367.926(252.399 to 504.464)	447.864(307.995 to 606.650)	0.405(0.263 to 0.547)
Western Europe	4766.264(4306.421 to 5410.055)	4833.938(4115.802 to 5669.693)	0.157(−0.035 to 0.350)	4212.387(3827.553 to 4693.037)	4778.950(4207.886 to 5528.960)	0.125(−0.021 to 0.271)	739.926(517.876 to 999.754)	858.162(600.366 to 1164.616)	0.143(−0.026 to 0.313)
Eastern Europe	4122.865(3579.308 to 4767.118)	5579.700(4387.273 to 7067.862)	−0.305(−0.524 to −0.084)	3774.062(3369.077 to 4255.228)	4231.791(3729.904 to 4771.111)	−0.218(−0.375 to −0.060)	638.265(441.051 to 874.642)	735.834(510.578 to 1005.600)	−0.238(−0.427 to −0.049)
Southern Latin America	3752.707(3288.815 to 4407.846)	5634.455(4852.739 to 6686.609)	−0.118(−0.351 to 0.116)	3231.930(2879.159 to 3675.036)	3605.125(3048.180 to 4246.275)	−0.148(−0.339 to 0.045)	575.287(398.251 to 784.187)	652.616(439.621 to 904.816)	−0.133(−0.346 to 0.081)
High-income North America	3907.663(3466.451 to 4476.331)	4330.318(3543.253 to 5341.734)	0.787(0.465 to 1.111)	3817.105(3439.494 to 4244.846)	5408.262(4846.896 to 6049.723)	0.439(0.232 to 0.646)	640.751(446.860 to 864.810)	982.776(685.334 to 1322.412)	0.593(0.334 to 0.852)
Australasia	5138.478(4487.900 to 5941.372)	6572.237(5787.265 to 7626.113)	0.121(0.000 to 0.243)	4407.642(3912.808 to 4998.583)	4691.880(3904.488 to 5719.887)	0.090(−0.008 to 0.188)	787.738(546.912 to 1065.625)	849.311(569.135 to 1201.644)	0.111(0.001 to 0.220)
North Africa and Middle East	5138.946(4371.200 to 6131.267)	4574.680(3953.813 to 5407.859)	0.256(0.117 to 0.395)	4468.969(3904.157 to 5216.627)	5024.694(4346.395 to 5857.380)	0.204(0.098 to 0.311)	788.135(536.471 to 1078.257)	900.679(598.457 to 1242.727)	0.231(0.108 to 0.354)
Central Latin America	3395.737(2968.171 to 3982.921)	4956.525(4094.797 to 6075.477)	0.551(0.374 to 0.729)	3046.790(2703.862 to 3461.755)	3825.604(3399.906 to 4375.352)	0.418(0.282 to 0.555)	522.112(361.212 to 711.516)	682.935(468.283 to 935.017)	0.502(0.344 to 0.660)
Caribbean	4605.729(3941.074 to 5390.404)	5983.105(4953.301 to 7214.680)	−0.253(−0.449 to −0.057)	3911.570(3420.758 to 4461.461)	4121.502(3512.088 to 4870.279)	−0.212(−0.365 to −0.059)	697.362(470.036 to 954.740)	737.808(507.642 to 1028.878)	−0.256(−0.432 to −0.080)
Andean Latin America	2985.028(2554.932 to 3527.330)	3764.830(3106.999 to 4617.212)	0.062(−0.243 to 0.367)	2821.688(2476.529 to 3232.064)	3325.811(2851.295 to 3880.446)	0.044(−0.172 to 0.261)	474.690(323.596 to 644.823)	578.737(389.315 to 804.971)	0.053(−0.208 to 0.314)
Tropical Latin America	4685.917(4140.040 to 5390.042)	5317.595(4592.416 to 6168.767)	−0.389(−0.759 to −0.016)	3931.871(3542.557 to 4398.764)	4352.088(3871.003 to 4948.916)	−0.303(−0.598 to −0.007)	694.297(485.379 to 940.861)	780.150(539.061 to 1062.152)	−0.341(−0.677 to −0.004)
Central Sub-Saharan Africa	7328.070(6128.838 to 8840.976)	5150.999(4461.450 to 6036.894)	−0.023(−0.125 to 0.079)	6100.874(5269.076 to 7235.874)	6337.031(5236.438 to 7669.981)	−0.024(−0.105 to 0.056)	1080.055(735.708 to 1470.357)	1136.909(756.883 to 1588.384)	0.010(−0.080 to 0.100)
Eastern Sub-Saharan Africa	6061.259(5226.476 to 7132.989)	7703.414(6194.218 to 9565.937)	−0.185(−0.304 to −0.065)	5330.371(4758.723 to 6057.556)	5576.415(4939.636 to 6372.720)	−0.150(−0.239 to −0.061)	917.788(628.421 to 1236.023)	974.649(668.672 to 1308.671)	−0.142(−0.244 to −0.040)
South Asia	4876.285(4256.822 to 5689.877)	6468.121(5519.803 to 7580.312)	−0.647(−0.923 to −0.371)	4307.023(3862.341 to 4834.861)	4500.484(4034.927 to 5106.633)	−0.451(−0.649 to −0.253)	737.680(508.977 to 996.629)	777.795(542.561 to 1049.654)	−0.539(−0.777 to −0.300)
Southern Sub-Saharan Africa	4708.445(4129.942 to 5473.825)	5878.905(5041.116 to 6920.416)	0.325(0.098 to 0.554)	4352.895(3926.747 to 4875.223)	5113.034(4540.526 to 5818.204)	0.234(0.071 to 0.398)	733.161(511.872 to 997.016)	880.694(609.091 to 1219.630)	0.256(0.056 to 0.457)
Western Sub-Saharan Africa	4746.000(4112.432 to 5549.906)	4739.638(4046.238 to 5556.828)	−0.153(−0.250 to −0.056)	4364.975(3895.290 to 4942.000)	4372.188(3880.291 to 4963.050)	−0.099(−0.168 to −0.030)	732.239(502.890 to 999.637)	736.530(502.606 to 1002.773)	−0.101(−0.182 to −0.019)

### Differences at the regional and national levels

**Fig 1:**
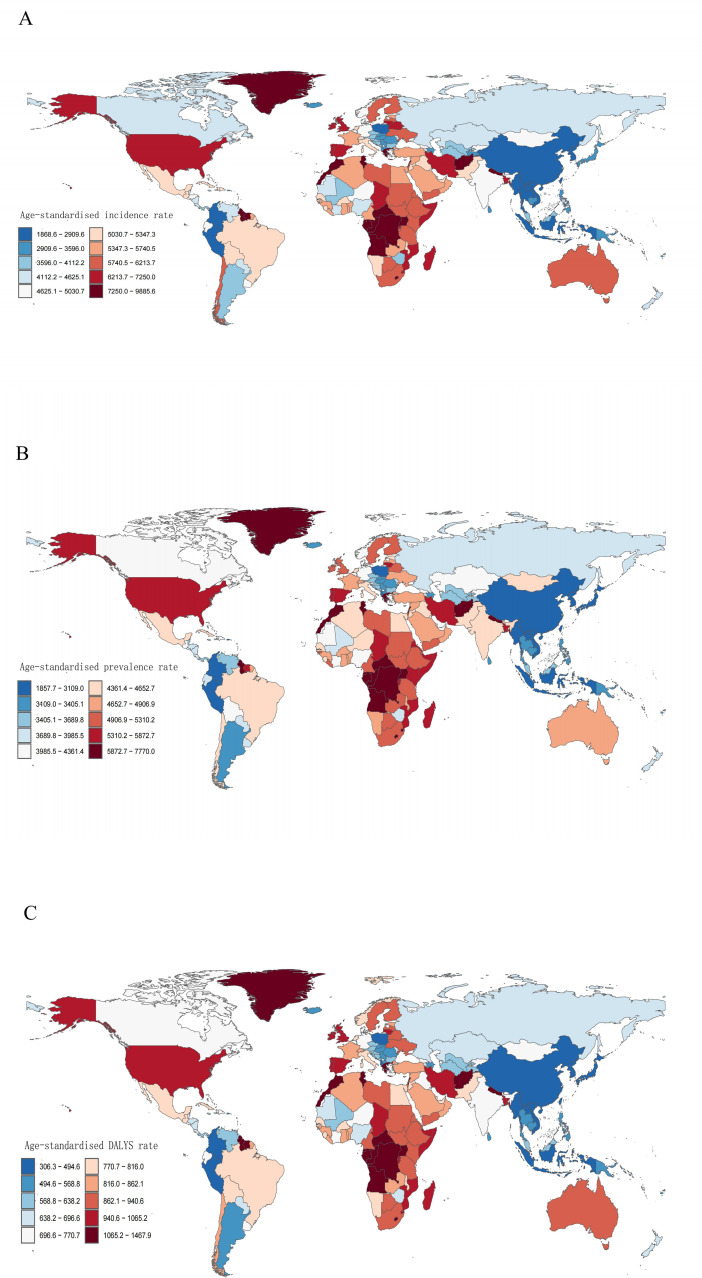
Global distribution of age-standardized incidence, prevalence and rates of DALYs.

African countries have higher rates of both incidence and prevalence of depression, with Uganda and the Gambia being particularly significant. Specifically, the incidence rate is about 9,644 cases per 100,000 people in Uganda and 7,624 cases in the Gambia (Fig 1A). In terms of prevalence, Uganda has about 7,770 cases and The Gambia has 7,222 cases (Fig 1B). In addition, Palestine has the highest DALYS for depression globally, with 1,357 cases per 100,000 people (Fig 1C).

### Socio-economic and health resource allocation implications

**Fig 2:**
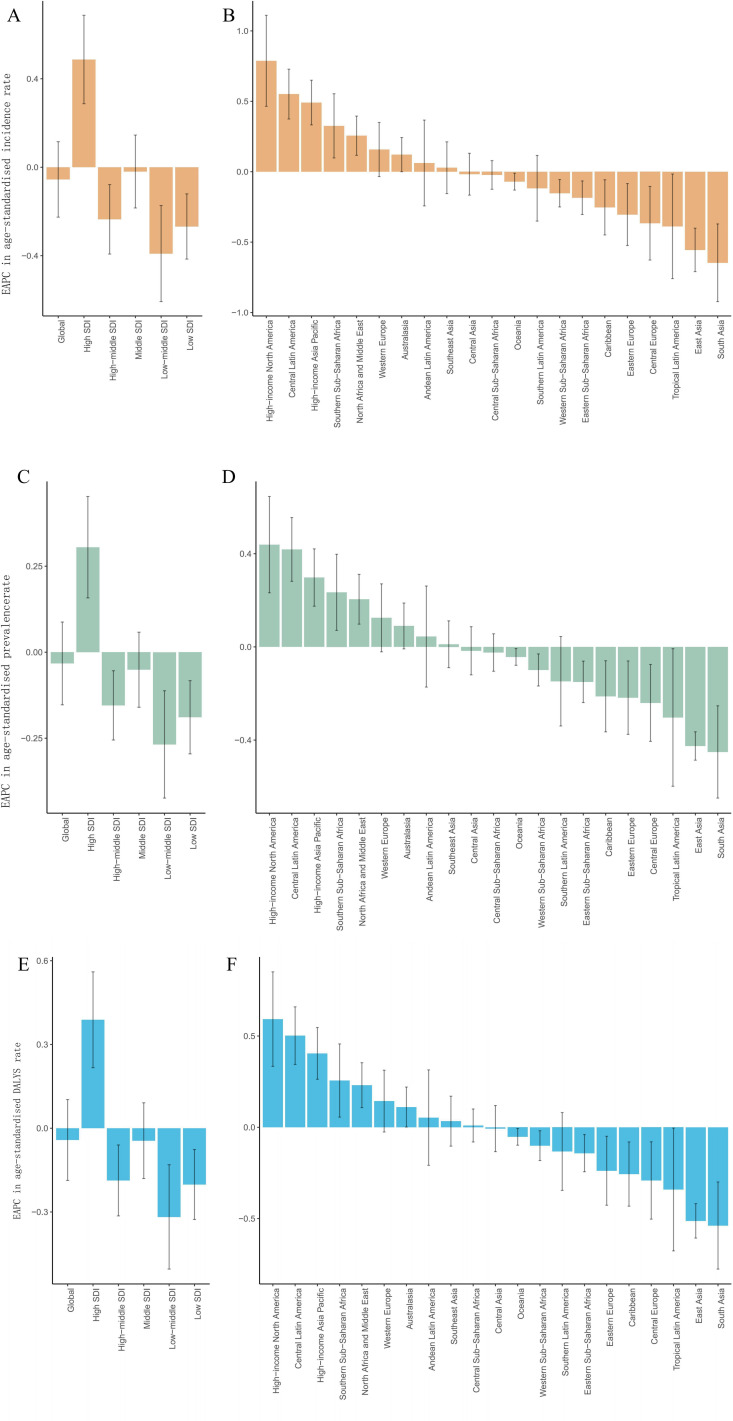
Estimated annual percentage change in age-standardized incidence, prevalence and rates of DALY’s, globally and by region (EAPC).

The study showed that EAPC values were generally higher in high-income areas and lower in low-income areas (Fig 2). Among them, the high-income North America region had the highest rate of change in incidence (1.0) (Fig 2A), while the lowest in Central Asia (−1.0) (Fig 2B), the highest rate of change in prevalence in high-income Asia-Pacific (0.4) (Fig 2C), the lowest in South Asia (−0.4) (Fig 2D), the highest rate of change in DALYS rate in high-income Asia Pacific (0.6) (Fig 2E), and the lowest rate of change in Eastern Europe (−0.3) (Fig 2F).

### Crowd analysis

The incidence of depression is generally higher in women than in men, especially during adolescence and early adulthood(S2 Table). The incidence of depression in women aged 15–19 was 5,583.94 per 100,000, compared with 3,400.62 per 100,000 for men in the same age group.

The global prevalence of depression among women aged 15–19 years in 2021 was 4,228.78 per 100,000, compared with 2,575.45 per 100,000 for men in the same age group(S3 Table). This gender difference is particularly significant in certain age groups, such as 6,014.01 per 100,000 in women and 3,962.54 per 100,000 in men in the 25–29 age group.(S4 Table) shows that DALYS are generally higher in women than in men in most age groups. In terms of age groups: DALYS are higher in adolescents and early adulthood (15–29 years).

**Fig 3:**
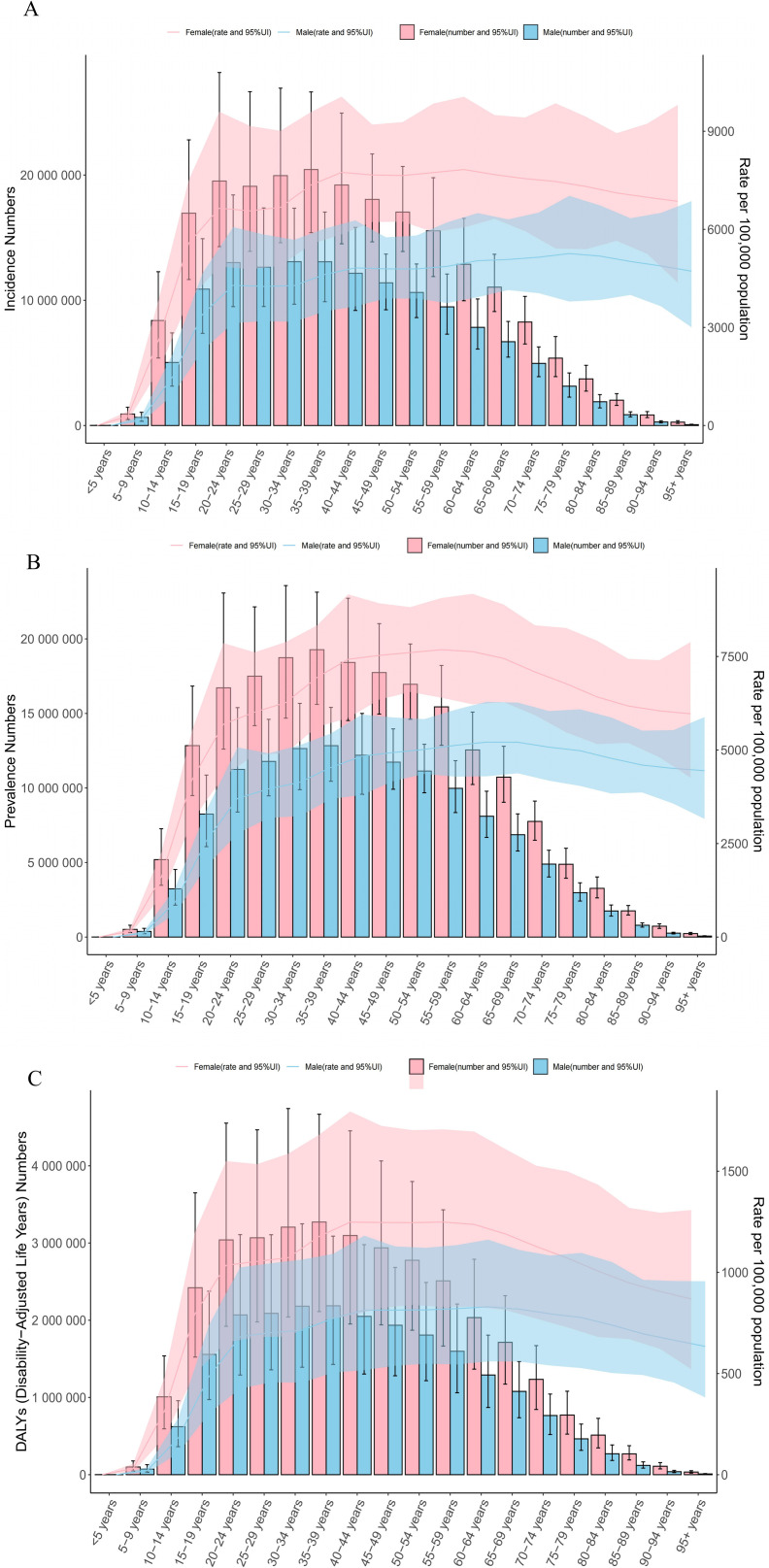
Distribution of incidence, prevalence and DALYs stratified by age and sex.

The global prevalence of depression varies significantly between age groups and genders(Fig 3). The prevalence of depression is significantly higher in women than in men, particularly in adolescence, and this trend continues into early adulthood. In addition, the prevalence of depression decreases in the older population with age, but remains at a high level.

### Regional gender differences

Globally, the incidence of depression in women (5,295.22 per 100,000 people) is significantly higher than that in men (3,366.27 per 100,000 people)(S5 Table). Especially in high-income North America, the incidence rate in females (8,587.86 per 100,000 population) is much higher than that in males (4,566.66 per 100,000 population). In addition, Eastern Europe, Central Asia and the Caribbean also show significant gender differences, with females all having a higher incidence than males.

Globally, the prevalence of depression in women (4,822.12 per 100,000 people) is higher than that in men (3,186.43 per 100,000 people)(S6 Table), and in high-income North America, the prevalence of depression in men is 3,853.58 per 100,000 people, and the prevalence rate in women is 6,964.76 per 100,000 people. In Eastern Europe: the prevalence of depression in men was 3,629.66 per 100,000 population and in women it was 4,766.92 per 100,000 population.

Globally, the rate of DALYS for depression (821.17 per 100,000 people) is significantly higher in women than in men (540.51 per 100,000 people) (S7 Table). In Western Europe, the DALYS rate for women (1,087.54 per 100,000 population) is much higher than that for men (630.85 per 100,000 population). In addition, Eastern Europe, South Asia and the Caribbean also showed significant gender differences, with women having higher rates of DALYS than men.

**Fig 4:**
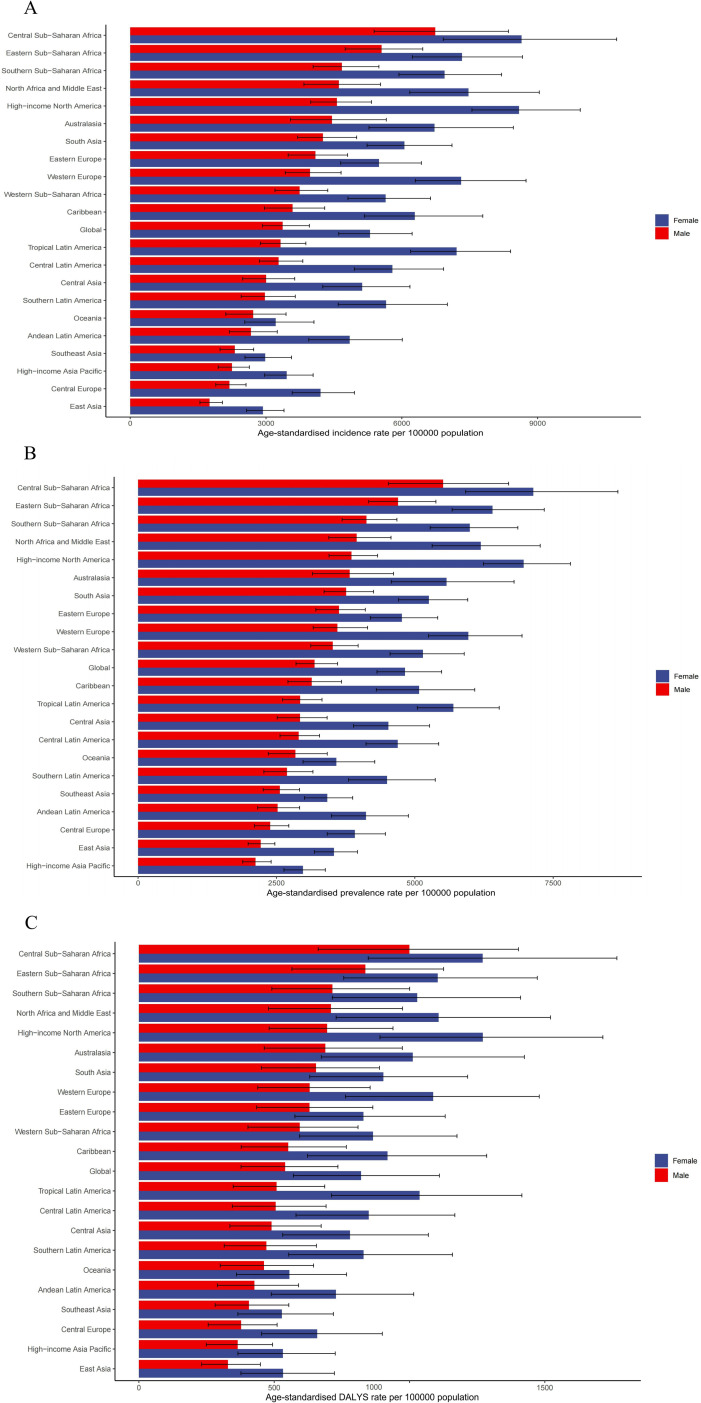
Sex differences in age-standardized incidence, prevalence andrates of DALYs by region.

The incidence, prevalence, and DALYS of depression were higher in women than in men in most regions (Fig 4), which may reflect differences in socioeconomic status, cultural factors, and perceptions of mental health problems and coping strategies in different regions.

### Regional time differences

The incidence of depression is highest in high-income North America, at 6,572 per 100,000 population, while in East Asia it is relatively low at 2,338 per 100,000 population(S8 Table). The prevalence of depression is highest in high-income North America, at 5,408 per 100,000 population, while in East Asia it is relatively low at 2,871 per 100,000 population (S9 Table).

Sub-Saharan Africa has the highest rate of DALYS for depression at 1137 per 100,000 people, while East Asia has a relatively low rate of DALYS at 430 per 100,000 people(S10 Table).

**Fig 5:**
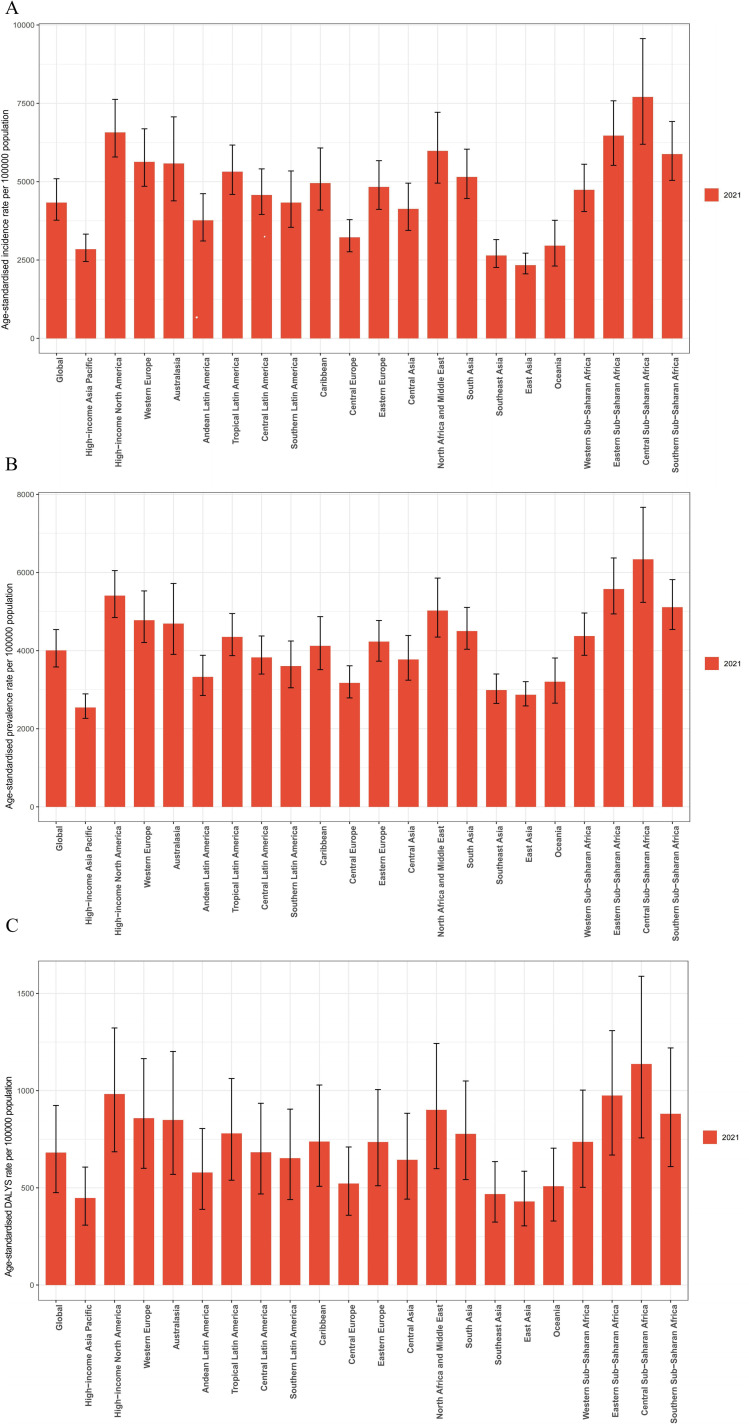
Global and regional age-standardized incidence, prevalence and DALYs rates, 2021.

Fig 5 shows that there are significant differences in the incidence, prevalence and disability-adjusted life years of depression in different regions.

Incidence is higher in High-income Asia Pacific and High-income North America, while lower in Central Asia and Central Sub-Saharan Africa(S11 Table and S6 FigA). The prevalence of depression in East Asia decreased from 3,059.66 per 100,000 population in 1990–2,870.61 per 100,000 population in 2021(S12 Table and S6 FigB).

From 1990 to 2021, the global rate of DALYs due to depression varied(S13 Table and S6 FigC). The global DALYs rate changed from 601 in 1990–681 in 2021. The DALYS rate in high-income North America increased from 641 in 1990–983 in 2021. In contrast, the DALYS rate in the East Asia Pacific region changed from 471 in 1990–430 in 2021, showing a downward trend.

## Discussion

Based on data from the 2021 Global Burden of Disease Study, this study provides a comprehensive analysis of global, regional, and national depression incidence, prevalence, and disability-adjusted life years between 1990 and 2021. We found that the incidence, prevalence, and disability-adjusted life years of depression increased globally over the past three decades. This finding is consistent with previous findings and further confirms the importance of depression as a global public health problem [2]. However, our results reveal significant regional and gender disparities, especially in high-income regions and among women, with a more significant increase in depression, providing deeper insights into the global distribution of depression [14].

The findings reveal unusually high values in the incidence and prevalence of depression in African countries. A striking aspect of our results is the pronounced regional disparities in depression burden, with Sub-Saharan Africa emerging as a critical area of concern. ongoing social conflict, economic hardship, and lack of medical resources could potentially be key factors contributing to the increased burden of depression in these countries [15], Uganda’s incidence (9,644/100,000) may be associated with prolonged civil conflict [16], where trauma and displacement exacerbate depressive symptoms. Moreover, the severe shortage of mental health resources in countries like the Gambia, where a mere two psychiatrists serve a population of 2.5 million [17], highlights the dire need for improved mental health infrastructure. These factors, combined with the challenges posed by infectious diseases and cultural stigma surrounding mental illness, create a complex web of issues that contribute to the region’s elevated depression burden.

The influence of socio-economic status and medical resource allocation on depression is another key highlight of this study. We found that high-income areas usually show higher EAPC values, which may reflect better access to mental health services and higher diagnosis rates. However, this association also hints at possible health inequalities, that is, areas with less resources may fail to adequately identify and treat depression [14]. This finding emphasizes the importance of fair distribution of medical resources on a global scale, especially in low- and middle-income countries. In addition, this also prompts the need to raise mental health awareness and improve mental health services in primary health care centers.

In addition to the socio-economic and healthcare resource factors, cultural factors and differences in perceptions of mental health may also influence the reporting and diagnosis rate of depression. In their study, Kirmayer et al. noted that the experience and manifestation of depression differ significantly between cultures, suggesting that cultural factors play an important role in the recognition and understanding of depression [18]. They emphasize that certain symptoms, such as loneliness, anger, crying, and diffuse pain, are not included in the diagnostic and statistical manual of mental disorders, fifth edition, diagnostic criteria for DSM-5, although prevalent in multiple cultures. The cultural relevance and presentation of these symptoms may vary from region to region, thus influencing the reporting and diagnosis of depression [18]. In addition, Kirmayer et al. discussed the “looping effects”, in which people perceive and act on the world through our constructions, resulting in the phenomena we are trying to describe, which may alter the individual’s experience and physiological state, further influencing the reporting and diagnosis of depression [18].

Gender differences are another notable finding of this study. “The 64% female predominance in adolescents mirrors estrogen’s neuro-modulatory effects [19]and societal pressures. Conversely, higher male underreporting—linked to stigma [20]—may mask true disparities.” In general, women exhibit a higher prevalence of depression than men across incidence, prevalence, and DALYs metrics, especially during adolescence and early adulthood. This phenomenon may be related to hormonal changes, social role expectations, and gender-specific life stressors [21,20]. Additionally, women are more likely to seek mental health services, which can lead to higher reporting rates. The cumulative effect of these factors may lead to gender differences in depression risk at different life stages, and in view of the significance of gender differences, we can use biopsychosocial models to fully understand the pathogenesis of depression in different genders. This model considers the interaction of biological, psychological, and sociocultural factors and how these factors work together to influence the formation of gender differences in depression. According to a review article in Progress in Neurobiology, gender differences are present in multiple aspects of depression, including epidemiology, symptomatology, treatment, and pathophysiology [19].

There are several limitations to this study: First, the study used incidence, prevalence, and disability-adjusted life years to assess the burden of depression, but these measures may not fully reflect the actual impact of depression on patients’ quality of life. Secondly, the study is mainly based on group data, and fails to deeply analyze the influencing factors at the individual level, such as personal experience and family background, which may have an important impact on the occurrence and development of depression. Finally, depression is often comorbid with other diseases, such as cardiovascular disease, diabetes, etc., which may have an impact on the disease burden of depression, but this has not been analysed in detail.

Additionally, reporting biases across regions are a concern. In areas with limited mental health infrastructure, such as The Gambia (only 2 psychiatrists for 2.5 million people), underdiagnosis of depression is likely, possibly underestimating the true depression burden. Cultural factors also play a significant role in reporting depressive symptoms. In some cultures, certain depression symptoms may be expressed or perceived differently, and may not align with the GBD study’s diagnostic criteria. This could influence reported depression rates and introduce data biases.

## Conclusion

This study, combined with the Global Burden of Disease database, found that the burden of depression has risen, especially in parts of Africa, such as Uganda and the Gambia, and among women. These disparities may be related to access to socioeconomic, cultural, and mental health services. The study highlights the need for strategies for the prevention, identification, and treatment of depression globally, especially in low- and middle-income countries, and the need to consider gender-specific factors in public health policies. Future research needs to adopt a multidisciplinary approach to deeply explore the pathogenesis of depression and provide scientific support for the management of depression worldwide.

## Supporting information

S2 Table Global incidence of depression (by age, sex). (DOCX)

S3 Table Global prevalence of depression (by age, sex). (DOCX)

S4 Table Global DALYs of depression (by age, sex). (DOCX)

S5 Table Global and regional gender-age standardized depression incidence data (2021). (DOCX)

S6 Table Global and regional gender-age standardized depression prevalence data (2021). (DOCX)

S7 Table Global and regional gender-age standardized depression DALYsdata (2021). (DOCX)

S8 Table Global and Regional Age-Standardized Depression Incidence Temporal Trend Data (2021). (DOCX)

S9 Table Global and Regional Age-Standardized Depression Prevalence Temporal Trend Data (2021). (DOCX)

S10 Table Global and Regional Age-Standardized Depression DALYs Temporal Trend Data (2021). (DOCX)

S11 Table Global and Regional Time Series Data on Depression Incidence (1990–2021). (DOCX)

S12 Table Global and Regional Time Series Data on Depression Prevalence (1990–2021). (DOCX)

S13 Table Global and Regional Time Series Data on Depression DALYs (1990–2021). (DOCX)

S6 Fig The relationship between global and regional health indicators and socio-demographic indicators: a comparative analysis of morbidity, Prevalence and DALYs rates. (TIF)
