# Supplementary material for: Unmasking the rising global burden of depression: A 32-year GBD analysis of gender disparities and regional hotspots in Sub-Saharan Africa
Source: PLoS One. 2025 Jul 31;20(7):e0326974. doi: 10.1371/journal.pone.0326974 (PMC12312894; doi:10.1371/journal.pone.0326974)
Supplement: S2 Table — (DOCX) [file pone.0326974.s001.docx]

| **Supplementary Table 2 Global incidence of depression (by age, sex)** | | | | | | | | | |
| --- | --- | --- | --- | --- | --- | --- | --- | --- | --- |
| **measure** | **location** | **sex** | **age** | **cause** | **metric** | **year** | **value** | **upper** | **lower** |
| Incidence | Global | Male | <5 years | Depressive disorders | Number | 2021 | 10874.67049 | 18588.02756 | 5417.458707 |
| Incidence | Global | Female | <5 years | Depressive disorders | Number | 2021 | 12300.89202 | 20481.21431 | 6223.814207 |
| Incidence | Global | Male | <5 years | Depressive disorders | Percent | 2021 | 5.47009E-06 | 9.35986E-06 | 2.68391E-06 |
| Incidence | Global | Female | <5 years | Depressive disorders | Percent | 2021 | 6.52811E-06 | 1.1131E-05 | 3.23215E-06 |
| Incidence | Global | Male | <5 years | Depressive disorders | Rate | 2021 | 3.198627846 | 5.46740084 | 1.593467526 |
| Incidence | Global | Female | <5 years | Depressive disorders | Rate | 2021 | 3.86586541 | 6.436737907 | 1.955990511 |
| Incidence | Global | Male | 5-9 years | Depressive disorders | Number | 2021 | 652159.2502 | 1057566.42 | 341140.9463 |
| Incidence | Global | Female | 5-9 years | Depressive disorders | Number | 2021 | 916199.0455 | 1462364.65 | 477062.723 |
| Incidence | Global | Male | 5-9 years | Depressive disorders | Percent | 2021 | 0.000318327 | 0.000535458 | 0.000163383 |
| Incidence | Global | Female | 5-9 years | Depressive disorders | Percent | 2021 | 0.0004721 | 0.000788379 | 0.000244121 |
| Incidence | Global | Male | 5-9 years | Depressive disorders | Rate | 2021 | 183.9781639 | 298.3460375 | 96.23797397 |
| Incidence | Global | Female | 5-9 years | Depressive disorders | Rate | 2021 | 275.4846832 | 439.7069221 | 143.4442371 |
| Incidence | Global | Male | 10-14 years | Depressive disorders | Number | 2021 | 5036893.49 | 7396173.874 | 3146357.318 |
| Incidence | Global | Female | 10-14 years | Depressive disorders | Number | 2021 | 8387213.221 | 12282257.69 | 5392383.419 |
| Incidence | Global | Male | 10-14 years | Depressive disorders | Percent | 2021 | 0.003065473 | 0.004484169 | 0.001898285 |
| Incidence | Global | Female | 10-14 years | Depressive disorders | Percent | 2021 | 0.005190668 | 0.007525014 | 0.003296919 |
| Incidence | Global | Male | 10-14 years | Depressive disorders | Rate | 2021 | 1465.449086 | 2151.865285 | 915.4107519 |
| Incidence | Global | Female | 10-14 years | Depressive disorders | Rate | 2021 | 2597.254958 | 3803.427173 | 1669.850784 |
| Incidence | Global | Male | 15-19 years | Depressive disorders | Number | 2021 | 10893208.87 | 14919910.22 | 7360853.266 |
| Incidence | Global | Female | 15-19 years | Depressive disorders | Number | 2021 | 16955625.11 | 22811613.96 | 11658045.74 |
| Incidence | Global | Male | 15-19 years | Depressive disorders | Percent | 2021 | 0.007809062 | 0.010812175 | 0.005356689 |
| Incidence | Global | Female | 15-19 years | Depressive disorders | Percent | 2021 | 0.011937478 | 0.016460249 | 0.00818513 |
| Incidence | Global | Male | 15-19 years | Depressive disorders | Rate | 2021 | 3400.621892 | 4657.670107 | 2297.897622 |
| Incidence | Global | Female | 15-19 years | Depressive disorders | Rate | 2021 | 5583.937752 | 7512.470433 | 3839.304141 |
| Incidence | Global | Male | 20-24 years | Depressive disorders | Number | 2021 | 13012569.56 | 18414610.02 | 9492598.492 |
| Incidence | Global | Female | 20-24 years | Depressive disorders | Number | 2021 | 19524170.6 | 28189084.79 | 14284978.17 |
| Incidence | Global | Male | 20-24 years | Depressive disorders | Percent | 2021 | 0.009841691 | 0.013743074 | 0.006947957 |
| Incidence | Global | Female | 20-24 years | Depressive disorders | Percent | 2021 | 0.013824179 | 0.019130371 | 0.009734324 |
| Incidence | Global | Male | 20-24 years | Depressive disorders | Rate | 2021 | 4288.829177 | 6069.294489 | 3128.677482 |
| Incidence | Global | Female | 20-24 years | Depressive disorders | Rate | 2021 | 6646.480165 | 9596.217774 | 4862.937639 |
| Incidence | Global | Male | 25-29 years | Depressive disorders | Number | 2021 | 12647476.79 | 17374495.93 | 9502953.37 |
| Incidence | Global | Female | 25-29 years | Depressive disorders | Number | 2021 | 19103089.32 | 26658737.4 | 13908914.35 |
| Incidence | Global | Male | 25-29 years | Depressive disorders | Percent | 2021 | 0.009845717 | 0.013305116 | 0.007210405 |
| Incidence | Global | Female | 25-29 years | Depressive disorders | Percent | 2021 | 0.013467049 | 0.018521065 | 0.009872481 |
| Incidence | Global | Male | 25-29 years | Depressive disorders | Rate | 2021 | 4253.312194 | 5842.995928 | 3195.817483 |
| Incidence | Global | Female | 25-29 years | Depressive disorders | Rate | 2021 | 6564.922676 | 9161.478896 | 4779.904743 |
| Incidence | Global | Male | 30-34 years | Depressive disorders | Number | 2021 | 13083568.92 | 17355444.47 | 9687602.996 |
| Incidence | Global | Female | 30-34 years | Depressive disorders | Number | 2021 | 19955617.99 | 26940779.18 | 14600131.74 |
| Incidence | Global | Male | 30-34 years | Depressive disorders | Percent | 2021 | 0.010229734 | 0.013577018 | 0.007458686 |
| Incidence | Global | Female | 30-34 years | Depressive disorders | Percent | 2021 | 0.014012576 | 0.018861584 | 0.010152848 |
| Incidence | Global | Male | 30-34 years | Depressive disorders | Rate | 2021 | 4281.982914 | 5680.07989 | 3170.553139 |
| Incidence | Global | Female | 30-34 years | Depressive disorders | Rate | 2021 | 6675.663112 | 9012.377663 | 4884.116392 |
| Incidence | Global | Male | 35-39 years | Depressive disorders | Number | 2021 | 13077385.96 | 17047239.97 | 9887826.672 |
| Incidence | Global | Female | 35-39 years | Depressive disorders | Number | 2021 | 20443426.77 | 26638877.57 | 15402041.27 |
| Incidence | Global | Male | 35-39 years | Depressive disorders | Percent | 2021 | 0.011152998 | 0.014637101 | 0.008239761 |
| Incidence | Global | Female | 35-39 years | Depressive disorders | Percent | 2021 | 0.015545379 | 0.020499937 | 0.011196669 |
| Incidence | Global | Male | 35-39 years | Depressive disorders | Rate | 2021 | 4619.949897 | 6022.411113 | 3493.149469 |
| Incidence | Global | Female | 35-39 years | Depressive disorders | Rate | 2021 | 7358.972491 | 9589.134416 | 5544.236751 |
| Incidence | Global | Male | 40-44 years | Depressive disorders | Number | 2021 | 12158703.39 | 15822172.74 | 9189237.29 |
| Incidence | Global | Female | 40-44 years | Depressive disorders | Number | 2021 | 19207159.45 | 24950926.37 | 14510511.52 |
| Incidence | Global | Male | 40-44 years | Depressive disorders | Percent | 2021 | 0.011800624 | 0.01572382 | 0.008644024 |
| Incidence | Global | Female | 40-44 years | Depressive disorders | Percent | 2021 | 0.016782255 | 0.022397285 | 0.012158266 |
| Incidence | Global | Male | 40-44 years | Depressive disorders | Rate | 2021 | 4821.802933 | 6274.632784 | 3644.195428 |
| Incidence | Global | Female | 40-44 years | Depressive disorders | Rate | 2021 | 7742.017129 | 10057.21329 | 5848.89343 |
| Incidence | Global | Male | 45-49 years | Depressive disorders | Number | 2021 | 11396586.17 | 13689448.07 | 9244293.483 |
| Incidence | Global | Female | 45-49 years | Depressive disorders | Number | 2021 | 18059504.26 | 21686443.61 | 14667827.67 |
| Incidence | Global | Male | 45-49 years | Depressive disorders | Percent | 2021 | 0.012228595 | 0.015394627 | 0.009558841 |
| Incidence | Global | Female | 45-49 years | Depressive disorders | Percent | 2021 | 0.017709101 | 0.022210439 | 0.013826763 |
| Incidence | Global | Male | 45-49 years | Depressive disorders | Rate | 2021 | 4791.258713 | 5755.204793 | 3886.409583 |
| Incidence | Global | Female | 45-49 years | Depressive disorders | Rate | 2021 | 7663.939399 | 9203.109191 | 6224.608424 |
| Incidence | Global | Male | 50-54 years | Depressive disorders | Number | 2021 | 10629651.41 | 12899762.06 | 8618357.067 |
| Incidence | Global | Female | 50-54 years | Depressive disorders | Number | 2021 | 17045851.79 | 20688226.89 | 13896700.96 |
| Incidence | Global | Male | 50-54 years | Depressive disorders | Percent | 2021 | 0.012507018 | 0.015515358 | 0.009773953 |
| Incidence | Global | Female | 50-54 years | Depressive disorders | Percent | 2021 | 0.018398745 | 0.022856526 | 0.014431244 |
| Incidence | Global | Male | 50-54 years | Depressive disorders | Rate | 2021 | 4788.553696 | 5811.216276 | 3882.485321 |
| Incidence | Global | Female | 50-54 years | Depressive disorders | Rate | 2021 | 7645.848897 | 9279.621737 | 6233.30984 |
| Incidence | Global | Male | 55-59 years | Depressive disorders | Number | 2021 | 9478347.149 | 12098585.19 | 7296932.996 |
| Incidence | Global | Female | 55-59 years | Depressive disorders | Number | 2021 | 15550185.66 | 19789244.13 | 11896608.62 |
| Incidence | Global | Male | 55-59 years | Depressive disorders | Percent | 2021 | 0.012797119 | 0.017344378 | 0.009540346 |
| Incidence | Global | Female | 55-59 years | Depressive disorders | Percent | 2021 | 0.018948016 | 0.025134219 | 0.014325806 |
| Incidence | Global | Male | 55-59 years | Depressive disorders | Rate | 2021 | 4867.568342 | 6213.181402 | 3747.311582 |
| Incidence | Global | Female | 55-59 years | Depressive disorders | Rate | 2021 | 7736.275148 | 9845.222487 | 5918.606992 |
| Incidence | Global | Male | 60-64 years | Depressive disorders | Number | 2021 | 7841049.766 | 10106970.33 | 6105589.112 |
| Incidence | Global | Female | 60-64 years | Depressive disorders | Number | 2021 | 12880896.55 | 16541856.2 | 9964185.884 |
| Incidence | Global | Male | 60-64 years | Depressive disorders | Percent | 2021 | 0.012902553 | 0.017142086 | 0.009869961 |
| Incidence | Global | Female | 60-64 years | Depressive disorders | Percent | 2021 | 0.0187689 | 0.024701407 | 0.01448047 |
| Incidence | Global | Male | 60-64 years | Depressive disorders | Rate | 2021 | 5041.278783 | 6498.116529 | 3925.491837 |
| Incidence | Global | Female | 60-64 years | Depressive disorders | Rate | 2021 | 7829.811865 | 10055.17135 | 6056.853308 |
| Incidence | Global | Male | 65-69 years | Depressive disorders | Number | 2021 | 6694300.273 | 8311630.721 | 5461797.537 |
| Incidence | Global | Female | 65-69 years | Depressive disorders | Number | 2021 | 11051298.89 | 13674756.96 | 9102533.658 |
| Incidence | Global | Male | 65-69 years | Depressive disorders | Percent | 2021 | 0.012777352 | 0.016311122 | 0.010220655 |
| Incidence | Global | Female | 65-69 years | Depressive disorders | Percent | 2021 | 0.018120386 | 0.022961857 | 0.014545274 |
| Incidence | Global | Male | 65-69 years | Depressive disorders | Rate | 2021 | 5077.854757 | 6304.654986 | 4142.959454 |
| Incidence | Global | Female | 65-69 years | Depressive disorders | Rate | 2021 | 7674.037651 | 9495.770659 | 6320.812303 |
| Incidence | Global | Male | 70-74 years | Depressive disorders | Number | 2021 | 4962742.402 | 6278278.171 | 3901713.44 |
| Incidence | Global | Female | 70-74 years | Depressive disorders | Number | 2021 | 8264311.913 | 10309868.59 | 6511644.389 |
| Incidence | Global | Male | 70-74 years | Depressive disorders | Percent | 2021 | 0.012406365 | 0.015854064 | 0.009677498 |
| Incidence | Global | Female | 70-74 years | Depressive disorders | Percent | 2021 | 0.017209252 | 0.021562365 | 0.013553228 |
| Incidence | Global | Male | 70-74 years | Depressive disorders | Rate | 2021 | 5148.531683 | 6513.316923 | 4047.781173 |
| Incidence | Global | Female | 70-74 years | Depressive disorders | Rate | 2021 | 7550.915873 | 9419.894991 | 5949.542986 |
| Incidence | Global | Male | 75-79 years | Depressive disorders | Number | 2021 | 3143936.183 | 4199794.353 | 2267877.261 |
| Incidence | Global | Female | 75-79 years | Depressive disorders | Number | 2021 | 5381283.883 | 7105328.663 | 3904897.394 |
| Incidence | Global | Male | 75-79 years | Depressive disorders | Percent | 2021 | 0.012188 | 0.016304868 | 0.008564451 |
| Incidence | Global | Female | 75-79 years | Depressive disorders | Percent | 2021 | 0.016500863 | 0.021951377 | 0.011697358 |
| Incidence | Global | Male | 75-79 years | Depressive disorders | Rate | 2021 | 5258.585879 | 7024.62709 | 3793.279077 |
| Incidence | Global | Female | 75-79 years | Depressive disorders | Rate | 2021 | 7463.879251 | 9855.141698 | 5416.120627 |
| Incidence | Global | Male | 80-84 years | Depressive disorders | Number | 2021 | 1899555.804 | 2468477.469 | 1407884.385 |
| Incidence | Global | Female | 80-84 years | Depressive disorders | Number | 2021 | 3723037.005 | 4808934.753 | 2746837.091 |
| Incidence | Global | Male | 80-84 years | Depressive disorders | Percent | 2021 | 0.01123477 | 0.015001827 | 0.008325813 |
| Incidence | Global | Female | 80-84 years | Depressive disorders | Percent | 2021 | 0.015280782 | 0.020272186 | 0.011171513 |
| Incidence | Global | Male | 80-84 years | Depressive disorders | Rate | 2021 | 5182.699295 | 6734.930561 | 3841.235616 |
| Incidence | Global | Female | 80-84 years | Depressive disorders | Rate | 2021 | 7309.934563 | 9442.022282 | 5393.231215 |
| Incidence | Global | Male | 85-89 years | Depressive disorders | Number | 2021 | 865623.7 | 1093759.46 | 689191.6192 |
| Incidence | Global | Female | 85-89 years | Depressive disorders | Number | 2021 | 2025047.44 | 2545135.296 | 1608316.859 |
| Incidence | Global | Male | 85-89 years | Depressive disorders | Percent | 2021 | 0.010065221 | 0.012955854 | 0.007885355 |
| Incidence | Global | Female | 85-89 years | Depressive disorders | Percent | 2021 | 0.014070751 | 0.017971074 | 0.011012341 |
| Incidence | Global | Male | 85-89 years | Depressive disorders | Rate | 2021 | 5017.334864 | 6339.65714 | 3994.697857 |
| Incidence | Global | Female | 85-89 years | Depressive disorders | Rate | 2021 | 7113.133875 | 8939.982209 | 5649.335865 |
| Incidence | Global | Male | 90-94 years | Depressive disorders | Number | 2021 | 284470.6638 | 379103.806 | 212549.1875 |
| Incidence | Global | Female | 90-94 years | Depressive disorders | Number | 2021 | 841936.0797 | 1113374.071 | 633344.2213 |
| Incidence | Global | Male | 90-94 years | Depressive disorders | Percent | 2021 | 0.009101128 | 0.012432264 | 0.006652887 |
| Incidence | Global | Female | 90-94 years | Depressive disorders | Percent | 2021 | 0.013009339 | 0.017586082 | 0.009583723 |
| Incidence | Global | Male | 90-94 years | Depressive disorders | Rate | 2021 | 4880.684766 | 6504.312769 | 3646.72254 |
| Incidence | Global | Female | 90-94 years | Depressive disorders | Rate | 2021 | 6980.721628 | 9231.288034 | 5251.229648 |
| Incidence | Global | Male | 95+ years | Depressive disorders | Number | 2021 | 71370.39197 | 103781.5611 | 45434.8836 |
| Incidence | Global | Female | 95+ years | Depressive disorders | Number | 2021 | 270098.9407 | 386287.466 | 172094.1101 |
| Incidence | Global | Male | 95+ years | Depressive disorders | Percent | 2021 | 0.008224337 | 0.012143049 | 0.005264437 |
| Incidence | Global | Female | 95+ years | Depressive disorders | Percent | 2021 | 0.011932861 | 0.017360452 | 0.007649673 |
| Incidence | Global | Male | 95+ years | Depressive disorders | Rate | 2021 | 4720.133273 | 6863.669737 | 3004.869386 |
| Incidence | Global | Female | 95+ years | Depressive disorders | Rate | 2021 | 6858.320041 | 9808.565195 | 4369.793087 |
