# Supplementary material for: Unmasking the rising global burden of depression: A 32-year GBD analysis of gender disparities and regional hotspots in Sub-Saharan Africa
Source: PLoS One. 2025 Jul 31;20(7):e0326974. doi: 10.1371/journal.pone.0326974 (PMC12312894; doi:10.1371/journal.pone.0326974)
Supplement: S3 Table — (DOCX) [file pone.0326974.s002.docx]

| **Supplementary Table 3 Global prevalence of depression (by age, sex)** | | | | | | | | | |
| --- | --- | --- | --- | --- | --- | --- | --- | --- | --- |
| **measure** | **location** | **sex** | **age** | **cause** | **metric** | **year** | **value** | **upper** | **lower** |
| Prevalence | Global | Male | <5 years | Depressive disorders | Number | 2021 | 4326.62546 | 7081.481133 | 2238.902423 |
| Prevalence | Global | Female | <5 years | Depressive disorders | Number | 2021 | 4875.294836 | 7766.535731 | 2536.729683 |
| Prevalence | Global | Male | <5 years | Depressive disorders | Percent | 2021 | 1.48229E-05 | 2.40918E-05 | 7.61886E-06 |
| Prevalence | Global | Female | <5 years | Depressive disorders | Percent | 2021 | 1.7333E-05 | 2.76057E-05 | 9.0225E-06 |
| Prevalence | Global | Male | <5 years | Depressive disorders | Rate | 2021 | 1.272614622 | 2.082915778 | 0.658540932 |
| Prevalence | Global | Female | <5 years | Depressive disorders | Rate | 2021 | 1.532184303 | 2.44082964 | 0.797231251 |
| Prevalence | Global | Male | 5-9 years | Depressive disorders | Number | 2021 | 376282.1936 | 585465.6829 | 228126.2867 |
| Prevalence | Global | Female | 5-9 years | Depressive disorders | Number | 2021 | 516651.1086 | 793834.2129 | 306866.2749 |
| Prevalence | Global | Male | 5-9 years | Depressive disorders | Percent | 2021 | 0.001198497 | 0.001867401 | 0.000727211 |
| Prevalence | Global | Female | 5-9 years | Depressive disorders | Percent | 2021 | 0.001699643 | 0.002608022 | 0.000998214 |
| Prevalence | Global | Male | 5-9 years | Depressive disorders | Rate | 2021 | 106.1515375 | 165.1634955 | 64.35583848 |
| Prevalence | Global | Female | 5-9 years | Depressive disorders | Rate | 2021 | 155.3477573 | 238.6917643 | 92.26920605 |
| Prevalence | Global | Male | 10-14 years | Depressive disorders | Number | 2021 | 3237374.677 | 4533992.497 | 2141340.749 |
| Prevalence | Global | Female | 10-14 years | Depressive disorders | Number | 2021 | 5195210.554 | 7266162.496 | 3473203.568 |
| Prevalence | Global | Male | 10-14 years | Depressive disorders | Percent | 2021 | 0.010447512 | 0.01455594 | 0.006927057 |
| Prevalence | Global | Female | 10-14 years | Depressive disorders | Percent | 2021 | 0.016954359 | 0.023739115 | 0.011346234 |
| Prevalence | Global | Male | 10-14 years | Depressive disorders | Rate | 2021 | 941.8916187 | 1319.133544 | 623.0081796 |
| Prevalence | Global | Female | 10-14 years | Depressive disorders | Rate | 2021 | 1608.792577 | 2250.100966 | 1075.541416 |
| Prevalence | Global | Male | 15-19 years | Depressive disorders | Number | 2021 | 8249935.039 | 10866198.81 | 6063650.953 |
| Prevalence | Global | Female | 15-19 years | Depressive disorders | Number | 2021 | 12840700.85 | 16836971.2 | 9489523.309 |
| Prevalence | Global | Male | 15-19 years | Depressive disorders | Percent | 2021 | 0.027326865 | 0.035968439 | 0.020160755 |
| Prevalence | Global | Female | 15-19 years | Depressive disorders | Percent | 2021 | 0.04302025 | 0.056368452 | 0.031875178 |
| Prevalence | Global | Male | 15-19 years | Depressive disorders | Rate | 2021 | 2575.449534 | 3392.189941 | 1892.939392 |
| Prevalence | Global | Female | 15-19 years | Depressive disorders | Rate | 2021 | 4228.783886 | 5544.861865 | 3125.152101 |
| Prevalence | Global | Male | 20-24 years | Depressive disorders | Number | 2021 | 11247414.68 | 15390109.02 | 8386918.205 |
| Prevalence | Global | Female | 20-24 years | Depressive disorders | Number | 2021 | 16718165.72 | 23077956.3 | 12607116.83 |
| Prevalence | Global | Male | 20-24 years | Depressive disorders | Percent | 2021 | 0.03853813 | 0.052592998 | 0.028778573 |
| Prevalence | Global | Female | 20-24 years | Depressive disorders | Percent | 2021 | 0.057329247 | 0.079036644 | 0.043276235 |
| Prevalence | Global | Male | 20-24 years | Depressive disorders | Rate | 2021 | 3707.049556 | 5072.445399 | 2764.254924 |
| Prevalence | Global | Female | 20-24 years | Depressive disorders | Rate | 2021 | 5691.251074 | 7856.27118 | 4291.754753 |
| Prevalence | Global | Male | 25-29 years | Depressive disorders | Number | 2021 | 11782836.77 | 14606201.67 | 9470974.296 |
| Prevalence | Global | Female | 25-29 years | Depressive disorders | Number | 2021 | 17500006.56 | 22147968.97 | 14180521.72 |
| Prevalence | Global | Male | 25-29 years | Depressive disorders | Percent | 2021 | 0.040629875 | 0.050542821 | 0.032748243 |
| Prevalence | Global | Female | 25-29 years | Depressive disorders | Percent | 2021 | 0.06038238 | 0.076372244 | 0.048933361 |
| Prevalence | Global | Male | 25-29 years | Depressive disorders | Rate | 2021 | 3962.536097 | 4912.026066 | 3185.063007 |
| Prevalence | Global | Female | 25-29 years | Depressive disorders | Rate | 2021 | 6014.01103 | 7611.318845 | 4873.244689 |
| Prevalence | Global | Male | 30-34 years | Depressive disorders | Number | 2021 | 12644234.19 | 15674209.34 | 9884789.081 |
| Prevalence | Global | Female | 30-34 years | Depressive disorders | Number | 2021 | 18739692.03 | 23575257.46 | 14685995.66 |
| Prevalence | Global | Male | 30-34 years | Depressive disorders | Percent | 2021 | 0.042112641 | 0.052165383 | 0.032983309 |
| Prevalence | Global | Female | 30-34 years | Depressive disorders | Percent | 2021 | 0.062845157 | 0.079052553 | 0.049274884 |
| Prevalence | Global | Male | 30-34 years | Depressive disorders | Rate | 2021 | 4138.197698 | 5129.846224 | 3235.088088 |
| Prevalence | Global | Female | 30-34 years | Depressive disorders | Rate | 2021 | 6268.904871 | 7886.524822 | 4912.840061 |
| Prevalence | Global | Male | 35-39 years | Depressive disorders | Number | 2021 | 12837823.33 | 15405684.97 | 10451141.9 |
| Prevalence | Global | Female | 35-39 years | Depressive disorders | Number | 2021 | 19275719.76 | 23134151.94 | 15606826.55 |
| Prevalence | Global | Male | 35-39 years | Depressive disorders | Percent | 2021 | 0.045903517 | 0.055053648 | 0.037332323 |
| Prevalence | Global | Female | 35-39 years | Depressive disorders | Percent | 2021 | 0.069479117 | 0.083367707 | 0.056244341 |
| Prevalence | Global | Male | 35-39 years | Depressive disorders | Rate | 2021 | 4535.317743 | 5442.486207 | 3692.156221 |
| Prevalence | Global | Female | 35-39 years | Depressive disorders | Rate | 2021 | 6938.635732 | 8327.546533 | 5617.952828 |
| Prevalence | Global | Male | 40-44 years | Depressive disorders | Number | 2021 | 12196231.4 | 15000550.25 | 9580389.553 |
| Prevalence | Global | Female | 40-44 years | Depressive disorders | Number | 2021 | 18420973.86 | 22726531.63 | 14521667.95 |
| Prevalence | Global | Male | 40-44 years | Depressive disorders | Percent | 2021 | 0.048756872 | 0.059867694 | 0.038299916 |
| Prevalence | Global | Female | 40-44 years | Depressive disorders | Percent | 2021 | 0.074315012 | 0.091685936 | 0.058581359 |
| Prevalence | Global | Male | 40-44 years | Depressive disorders | Rate | 2021 | 4836.685494 | 5948.800203 | 3799.315515 |
| Prevalence | Global | Female | 40-44 years | Depressive disorders | Rate | 2021 | 7425.121633 | 9160.604806 | 5853.390364 |
| Prevalence | Global | Male | 45-49 years | Depressive disorders | Number | 2021 | 11734233.92 | 13964526.43 | 9914810.33 |
| Prevalence | Global | Female | 45-49 years | Depressive disorders | Number | 2021 | 17742682.07 | 21023647.92 | 14956261.89 |
| Prevalence | Global | Male | 45-49 years | Depressive disorders | Percent | 2021 | 0.049600556 | 0.058985505 | 0.041940055 |
| Prevalence | Global | Female | 45-49 years | Depressive disorders | Percent | 2021 | 0.07536272 | 0.089297122 | 0.06352567 |
| Prevalence | Global | Male | 45-49 years | Depressive disorders | Rate | 2021 | 4933.209792 | 5870.850966 | 4168.302743 |
| Prevalence | Global | Female | 45-49 years | Depressive disorders | Rate | 2021 | 7529.489082 | 8921.83757 | 6347.011696 |
| Prevalence | Global | Male | 50-54 years | Depressive disorders | Number | 2021 | 11137281.58 | 12933131.51 | 9677452.692 |
| Prevalence | Global | Female | 50-54 years | Depressive disorders | Number | 2021 | 16956272.86 | 19659673.56 | 14607846.67 |
| Prevalence | Global | Male | 50-54 years | Depressive disorders | Percent | 2021 | 0.050334452 | 0.058469227 | 0.043767542 |
| Prevalence | Global | Female | 50-54 years | Depressive disorders | Percent | 2021 | 0.076125217 | 0.088273845 | 0.065569917 |
| Prevalence | Global | Male | 50-54 years | Depressive disorders | Rate | 2021 | 5017.236106 | 5826.248886 | 4359.597512 |
| Prevalence | Global | Female | 50-54 years | Depressive disorders | Rate | 2021 | 7605.668625 | 8818.268239 | 6552.291415 |
| Prevalence | Global | Male | 55-59 years | Depressive disorders | Number | 2021 | 9978202.044 | 11832431.48 | 8350630.368 |
| Prevalence | Global | Female | 55-59 years | Depressive disorders | Number | 2021 | 15441297.22 | 18218781.55 | 12860666.06 |
| Prevalence | Global | Male | 55-59 years | Depressive disorders | Percent | 2021 | 0.051323489 | 0.060843625 | 0.042945656 |
| Prevalence | Global | Female | 55-59 years | Depressive disorders | Percent | 2021 | 0.076869643 | 0.090697744 | 0.064033079 |
| Prevalence | Global | Male | 55-59 years | Depressive disorders | Rate | 2021 | 5124.266881 | 6076.499201 | 4288.433772 |
| Prevalence | Global | Female | 55-59 years | Depressive disorders | Rate | 2021 | 7682.102743 | 9063.911518 | 6398.229151 |
| Prevalence | Global | Male | 60-64 years | Depressive disorders | Number | 2021 | 8105312.847 | 9783033.39 | 6684874.502 |
| Prevalence | Global | Female | 60-64 years | Depressive disorders | Number | 2021 | 12552067.21 | 15090817.23 | 10233579.68 |
| Prevalence | Global | Male | 60-64 years | Depressive disorders | Percent | 2021 | 0.052152947 | 0.062937441 | 0.043007155 |
| Prevalence | Global | Female | 60-64 years | Depressive disorders | Percent | 2021 | 0.076329174 | 0.091767616 | 0.062227742 |
| Prevalence | Global | Male | 60-64 years | Depressive disorders | Rate | 2021 | 5211.182546 | 6289.846403 | 4297.934205 |
| Prevalence | Global | Female | 60-64 years | Depressive disorders | Rate | 2021 | 7629.928896 | 9173.139415 | 6220.607648 |
| Prevalence | Global | Male | 65-69 years | Depressive disorders | Number | 2021 | 6865600.628 | 8263316.894 | 5771691.361 |
| Prevalence | Global | Female | 65-69 years | Depressive disorders | Number | 2021 | 10727975.15 | 12798775.63 | 9043051.748 |
| Prevalence | Global | Male | 65-69 years | Depressive disorders | Percent | 2021 | 0.052094291 | 0.062692495 | 0.043790341 |
| Prevalence | Global | Female | 65-69 years | Depressive disorders | Percent | 2021 | 0.074508726 | 0.08889069 | 0.062805604 |
| Prevalence | Global | Male | 65-69 years | Depressive disorders | Rate | 2021 | 5207.79191 | 6268.007303 | 4378.024475 |
| Prevalence | Global | Female | 65-69 years | Depressive disorders | Rate | 2021 | 7449.521185 | 8887.48798 | 6279.50798 |
| Prevalence | Global | Male | 70-74 years | Depressive disorders | Number | 2021 | 4894350.577 | 5824865.053 | 4030990.997 |
| Prevalence | Global | Female | 70-74 years | Depressive disorders | Number | 2021 | 7756791.408 | 9121056.225 | 6495446.276 |
| Prevalence | Global | Male | 70-74 years | Depressive disorders | Percent | 2021 | 0.050782262 | 0.060440792 | 0.041822879 |
| Prevalence | Global | Female | 70-74 years | Depressive disorders | Percent | 2021 | 0.070878244 | 0.083347824 | 0.059353211 |
| Prevalence | Global | Male | 70-74 years | Depressive disorders | Rate | 2021 | 5077.579485 | 6042.929461 | 4181.89847 |
| Prevalence | Global | Female | 70-74 years | Depressive disorders | Rate | 2021 | 7087.205806 | 8333.703877 | 5934.743135 |
| Prevalence | Global | Male | 75-79 years | Depressive disorders | Number | 2021 | 2978118.509 | 3639937.953 | 2415527.33 |
| Prevalence | Global | Female | 75-79 years | Depressive disorders | Number | 2021 | 4884588.427 | 5957119.123 | 3947341.047 |
| Prevalence | Global | Male | 75-79 years | Depressive disorders | Percent | 2021 | 0.049815174 | 0.06088718 | 0.040405118 |
| Prevalence | Global | Female | 75-79 years | Depressive disorders | Percent | 2021 | 0.067752291 | 0.082629893 | 0.054752581 |
| Prevalence | Global | Male | 75-79 years | Depressive disorders | Rate | 2021 | 4981.237222 | 6088.20447 | 4040.240377 |
| Prevalence | Global | Female | 75-79 years | Depressive disorders | Rate | 2021 | 6774.9591 | 8262.566849 | 5474.990278 |
| Prevalence | Global | Male | 80-84 years | Depressive disorders | Number | 2021 | 1751295.364 | 2151971.651 | 1406673.381 |
| Prevalence | Global | Female | 80-84 years | Depressive disorders | Number | 2021 | 3269525.342 | 4034126.928 | 2627969.275 |
| Prevalence | Global | Male | 80-84 years | Depressive disorders | Percent | 2021 | 0.047783194 | 0.058715303 | 0.038380044 |
| Prevalence | Global | Female | 80-84 years | Depressive disorders | Percent | 2021 | 0.06419612 | 0.079208887 | 0.051599179 |
| Prevalence | Global | Male | 80-84 years | Depressive disorders | Rate | 2021 | 4778.189316 | 5871.384213 | 3837.931542 |
| Prevalence | Global | Female | 80-84 years | Depressive disorders | Rate | 2021 | 6419.494694 | 7920.738854 | 5159.842194 |
| Prevalence | Global | Male | 85-89 years | Depressive disorders | Number | 2021 | 793221.2092 | 952074.3448 | 667032.7329 |
| Prevalence | Global | Female | 85-89 years | Depressive disorders | Number | 2021 | 1758462.075 | 2117163.554 | 1477713.389 |
| Prevalence | Global | Male | 85-89 years | Depressive disorders | Percent | 2021 | 0.045977332 | 0.055184683 | 0.038663092 |
| Prevalence | Global | Female | 85-89 years | Depressive disorders | Percent | 2021 | 0.061767805 | 0.07436735 | 0.05190624 |
| Prevalence | Global | Male | 85-89 years | Depressive disorders | Rate | 2021 | 4597.674981 | 5518.420768 | 3866.260347 |
| Prevalence | Global | Female | 85-89 years | Depressive disorders | Rate | 2021 | 6176.732409 | 7436.698761 | 5190.581195 |
| Prevalence | Global | Male | 90-94 years | Depressive disorders | Number | 2021 | 262746.0237 | 322419.2089 | 207895.0674 |
| Prevalence | Global | Female | 90-94 years | Depressive disorders | Number | 2021 | 729500.309 | 892414.4093 | 579843.7232 |
| Prevalence | Global | Male | 90-94 years | Depressive disorders | Percent | 2021 | 0.045079714 | 0.055317945 | 0.035668819 |
| Prevalence | Global | Female | 90-94 years | Depressive disorders | Percent | 2021 | 0.060485033 | 0.073992724 | 0.048076582 |
| Prevalence | Global | Male | 90-94 years | Depressive disorders | Rate | 2021 | 4507.953468 | 5531.770836 | 3566.871448 |
| Prevalence | Global | Female | 90-94 years | Depressive disorders | Rate | 2021 | 6048.485992 | 7399.251224 | 4807.642428 |
| Prevalence | Global | Male | 95+ years | Depressive disorders | Number | 2021 | 67260.21706 | 88900.48375 | 47772.73706 |
| Prevalence | Global | Female | 95+ years | Depressive disorders | Number | 2021 | 235093.637 | 310614.6866 | 167856.5236 |
| Prevalence | Global | Male | 95+ years | Depressive disorders | Percent | 2021 | 0.04448311 | 0.058795087 | 0.031594918 |
| Prevalence | Global | Female | 95+ years | Depressive disorders | Percent | 2021 | 0.059694745 | 0.078870978 | 0.042621972 |
| Prevalence | Global | Male | 95+ years | Depressive disorders | Rate | 2021 | 4448.303838 | 5879.498764 | 3159.485041 |
| Prevalence | Global | Female | 95+ years | Depressive disorders | Rate | 2021 | 5969.46955 | 7887.09102 | 4262.192798 |
