# Supplementary material for: Unmasking the rising global burden of depression: A 32-year GBD analysis of gender disparities and regional hotspots in Sub-Saharan Africa
Source: PLoS One. 2025 Jul 31;20(7):e0326974. doi: 10.1371/journal.pone.0326974 (PMC12312894; doi:10.1371/journal.pone.0326974)
Supplement: S4 Table — (DOCX) [file pone.0326974.s003.docx]

| **Supplementary Table 4 Global DALYs of depression (by age, sex)** | | | | | | | | | |
| --- | --- | --- | --- | --- | --- | --- | --- | --- | --- |
| **measure** | **location** | **sex** | **age** | **cause** | **metric** | **year** | **value** | **upper** | **lower** |
| DALYs | Global | Male | <5 years | Depressive disorders | Number | 2021 | 821.5891821 | 1611.048871 | 346.1496243 |
| DALYs | Global | Female | <5 years | Depressive disorders | Number | 2021 | 930.0879849 | 1807.648376 | 400.2071348 |
| DALYs | Global | Male | <5 years | Depressive disorders | Percent | 2021 | 3.42073E-06 | 6.64204E-06 | 1.36056E-06 |
| DALYs | Global | Female | <5 years | Depressive disorders | Percent | 2021 | 4.66515E-06 | 8.93767E-06 | 1.93898E-06 |
| DALYs | Global | Male | <5 years | Depressive disorders | Rate | 2021 | 0.241658636 | 0.473866844 | 0.101814931 |
| DALYs | Global | Female | <5 years | Depressive disorders | Rate | 2021 | 0.292303596 | 0.568099071 | 0.125775181 |
| DALYs | Global | Male | 5-9 years | Depressive disorders | Number | 2021 | 71897.68378 | 129444.0515 | 33644.47056 |
| DALYs | Global | Female | 5-9 years | Depressive disorders | Number | 2021 | 99788.19001 | 178226.1292 | 46796.32529 |
| DALYs | Global | Male | 5-9 years | Depressive disorders | Percent | 2021 | 0.002263232 | 0.003924618 | 0.001111604 |
| DALYs | Global | Female | 5-9 years | Depressive disorders | Percent | 2021 | 0.003758767 | 0.006406286 | 0.001854537 |
| DALYs | Global | Male | 5-9 years | Depressive disorders | Rate | 2021 | 20.28278192 | 36.51696869 | 9.491313537 |
| DALYs | Global | Female | 5-9 years | Depressive disorders | Rate | 2021 | 30.00452581 | 53.58941267 | 14.0708189 |
| DALYs | Global | Male | 10-14 years | Depressive disorders | Number | 2021 | 621467.2638 | 957611.0921 | 362506.017 |
| DALYs | Global | Female | 10-14 years | Depressive disorders | Number | 2021 | 1008086.287 | 1538413.166 | 593669.4501 |
| DALYs | Global | Male | 10-14 years | Depressive disorders | Percent | 2021 | 0.019959411 | 0.029758809 | 0.012481263 |
| DALYs | Global | Female | 10-14 years | Depressive disorders | Percent | 2021 | 0.033112077 | 0.048437544 | 0.020430995 |
| DALYs | Global | Male | 10-14 years | Depressive disorders | Rate | 2021 | 180.8115728 | 278.6102789 | 105.4686013 |
| DALYs | Global | Female | 10-14 years | Depressive disorders | Rate | 2021 | 312.1724748 | 476.3979544 | 183.8406729 |
| DALYs | Global | Male | 15-19 years | Depressive disorders | Number | 2021 | 1557943.896 | 2375707.249 | 972594.0977 |
| DALYs | Global | Female | 15-19 years | Depressive disorders | Number | 2021 | 2421174.548 | 3649932.642 | 1522832.812 |
| DALYs | Global | Male | 15-19 years | Depressive disorders | Percent | 2021 | 0.034680338 | 0.050414244 | 0.02315852 |
| DALYs | Global | Female | 15-19 years | Depressive disorders | Percent | 2021 | 0.057970683 | 0.082223662 | 0.039563014 |
| DALYs | Global | Male | 15-19 years | Depressive disorders | Rate | 2021 | 486.3560575 | 741.6439156 | 303.622635 |
| DALYs | Global | Female | 15-19 years | Depressive disorders | Rate | 2021 | 797.3570938 | 1202.019774 | 501.5092968 |
| DALYs | Global | Male | 20-24 years | Depressive disorders | Number | 2021 | 2069031.952 | 3108479.463 | 1290591.797 |
| DALYs | Global | Female | 20-24 years | Depressive disorders | Number | 2021 | 3040490.863 | 4552662.73 | 1921336.888 |
| DALYs | Global | Male | 20-24 years | Depressive disorders | Percent | 2021 | 0.038970843 | 0.056291181 | 0.026559092 |
| DALYs | Global | Female | 20-24 years | Depressive disorders | Percent | 2021 | 0.06160319 | 0.08815693 | 0.042415884 |
| DALYs | Global | Male | 20-24 years | Depressive disorders | Rate | 2021 | 681.9348446 | 1024.527658 | 425.3677745 |
| DALYs | Global | Female | 20-24 years | Depressive disorders | Rate | 2021 | 1035.053557 | 1549.831906 | 654.0676057 |
| DALYs | Global | Male | 25-29 years | Depressive disorders | Number | 2021 | 2089211.635 | 3107431.76 | 1357915.973 |
| DALYs | Global | Female | 25-29 years | Depressive disorders | Number | 2021 | 3068097.728 | 4465907.839 | 1977866.962 |
| DALYs | Global | Male | 25-29 years | Depressive disorders | Percent | 2021 | 0.035903219 | 0.049645924 | 0.024876644 |
| DALYs | Global | Female | 25-29 years | Depressive disorders | Percent | 2021 | 0.056699732 | 0.07705046 | 0.040871302 |
| DALYs | Global | Male | 25-29 years | Depressive disorders | Rate | 2021 | 702.5962149 | 1045.020886 | 456.6634644 |
| DALYs | Global | Female | 25-29 years | Depressive disorders | Rate | 2021 | 1054.375238 | 1534.743368 | 679.7091009 |
| DALYs | Global | Male | 30-34 years | Depressive disorders | Number | 2021 | 2181968.293 | 3248606.745 | 1391483.576 |
| DALYs | Global | Female | 30-34 years | Depressive disorders | Number | 2021 | 3205876.372 | 4741597.382 | 2042397.008 |
| DALYs | Global | Male | 30-34 years | Depressive disorders | Percent | 2021 | 0.031458413 | 0.043796552 | 0.021432045 |
| DALYs | Global | Female | 30-34 years | Depressive disorders | Percent | 2021 | 0.052738779 | 0.071313249 | 0.03726976 |
| DALYs | Global | Male | 30-34 years | Depressive disorders | Rate | 2021 | 714.1133286 | 1063.202148 | 455.4039447 |
| DALYs | Global | Female | 30-34 years | Depressive disorders | Rate | 2021 | 1072.447401 | 1586.185242 | 683.233883 |
| DALYs | Global | Male | 35-39 years | Depressive disorders | Number | 2021 | 2187279.34 | 3089002.272 | 1427927.374 |
| DALYs | Global | Female | 35-39 years | Depressive disorders | Number | 2021 | 3271980.311 | 4667624.369 | 2112188.511 |
| DALYs | Global | Male | 35-39 years | Depressive disorders | Percent | 2021 | 0.028868793 | 0.03866746 | 0.020473589 |
| DALYs | Global | Female | 35-39 years | Depressive disorders | Percent | 2021 | 0.050913998 | 0.06703249 | 0.037562824 |
| DALYs | Global | Male | 35-39 years | Depressive disorders | Rate | 2021 | 772.7171923 | 1091.275869 | 504.4550146 |
| DALYs | Global | Female | 35-39 years | Depressive disorders | Rate | 2021 | 1177.807095 | 1680.19382 | 760.3195549 |
| DALYs | Global | Male | 40-44 years | Depressive disorders | Number | 2021 | 2050181.746 | 2978400.958 | 1303389.608 |
| DALYs | Global | Female | 40-44 years | Depressive disorders | Number | 2021 | 3097075.367 | 4452880.734 | 1951314.061 |
| DALYs | Global | Male | 40-44 years | Depressive disorders | Percent | 2021 | 0.024350294 | 0.034109566 | 0.016742467 |
| DALYs | Global | Female | 40-44 years | Depressive disorders | Percent | 2021 | 0.046027693 | 0.062047946 | 0.032453935 |
| DALYs | Global | Male | 40-44 years | Depressive disorders | Rate | 2021 | 813.0449469 | 1181.150819 | 516.8879963 |
| DALYs | Global | Female | 40-44 years | Depressive disorders | Rate | 2021 | 1248.368381 | 1794.866076 | 786.5351941 |
| DALYs | Global | Male | 45-49 years | Depressive disorders | Number | 2021 | 1935388.363 | 2683756.019 | 1281061.258 |
| DALYs | Global | Female | 45-49 years | Depressive disorders | Number | 2021 | 2937728.426 | 4062875.75 | 1940257.976 |
| DALYs | Global | Male | 45-49 years | Depressive disorders | Percent | 2021 | 0.020962381 | 0.02836185 | 0.014693414 |
| DALYs | Global | Female | 45-49 years | Depressive disorders | Percent | 2021 | 0.040684653 | 0.053997304 | 0.029781312 |
| DALYs | Global | Male | 45-49 years | Depressive disorders | Rate | 2021 | 813.660005 | 1128.282559 | 538.5732026 |
| DALYs | Global | Female | 45-49 years | Depressive disorders | Rate | 2021 | 1246.688298 | 1724.168786 | 823.3902402 |
| DALYs | Global | Male | 50-54 years | Depressive disorders | Number | 2021 | 1808077.866 | 2489358.628 | 1217187.137 |
| DALYs | Global | Female | 50-54 years | Depressive disorders | Number | 2021 | 2776968.529 | 3796452.784 | 1870943.632 |
| DALYs | Global | Male | 50-54 years | Depressive disorders | Percent | 2021 | 0.017151312 | 0.022840721 | 0.012143498 |
| DALYs | Global | Female | 50-54 years | Depressive disorders | Percent | 2021 | 0.034915076 | 0.04578701 | 0.025726108 |
| DALYs | Global | Male | 50-54 years | Depressive disorders | Rate | 2021 | 814.5213432 | 1121.431644 | 548.3308656 |
| DALYs | Global | Female | 50-54 years | Depressive disorders | Rate | 2021 | 1245.598168 | 1702.883768 | 839.2043108 |
| DALYs | Global | Male | 55-59 years | Depressive disorders | Number | 2021 | 1598335.695 | 2209957.554 | 1060785.944 |
| DALYs | Global | Female | 55-59 years | Depressive disorders | Number | 2021 | 2510197.116 | 3430149.76 | 1665758.031 |
| DALYs | Global | Male | 55-59 years | Depressive disorders | Percent | 2021 | 0.013699144 | 0.018466242 | 0.009582942 |
| DALYs | Global | Female | 55-59 years | Depressive disorders | Percent | 2021 | 0.028362646 | 0.037299573 | 0.020656568 |
| DALYs | Global | Male | 55-59 years | Depressive disorders | Rate | 2021 | 820.8190845 | 1134.915113 | 544.7624987 |
| DALYs | Global | Female | 55-59 years | Depressive disorders | Rate | 2021 | 1248.832392 | 1706.512251 | 828.7208101 |
| DALYs | Global | Male | 60-64 years | Depressive disorders | Number | 2021 | 1290728.406 | 1806930.033 | 870693.6677 |
| DALYs | Global | Female | 60-64 years | Depressive disorders | Number | 2021 | 2034468.404 | 2789676.535 | 1366790.259 |
| DALYs | Global | Male | 60-64 years | Depressive disorders | Percent | 2021 | 0.010738798 | 0.014771277 | 0.007421711 |
| DALYs | Global | Female | 60-64 years | Depressive disorders | Percent | 2021 | 0.022455504 | 0.029814201 | 0.015912616 |
| DALYs | Global | Male | 60-64 years | Depressive disorders | Rate | 2021 | 829.8533899 | 1161.737052 | 559.7987061 |
| DALYs | Global | Female | 60-64 years | Depressive disorders | Rate | 2021 | 1236.676716 | 1695.739295 | 830.8203197 |
| DALYs | Global | Male | 65-69 years | Depressive disorders | Number | 2021 | 1078688.117 | 1464664.324 | 737181.446 |
| DALYs | Global | Female | 65-69 years | Depressive disorders | Number | 2021 | 1714066.491 | 2317735.082 | 1172657.111 |
| DALYs | Global | Male | 65-69 years | Depressive disorders | Percent | 2021 | 0.008710581 | 0.011749298 | 0.00624906 |
| DALYs | Global | Female | 65-69 years | Depressive disorders | Percent | 2021 | 0.017668935 | 0.022972591 | 0.012870185 |
| DALYs | Global | Male | 65-69 years | Depressive disorders | Rate | 2021 | 818.2216756 | 1110.997774 | 559.1772344 |
| DALYs | Global | Female | 65-69 years | Depressive disorders | Rate | 2021 | 1190.250206 | 1609.438533 | 814.294764 |
| DALYs | Global | Male | 70-74 years | Depressive disorders | Number | 2021 | 766199.2439 | 1044054.468 | 520222.5513 |
| DALYs | Global | Female | 70-74 years | Depressive disorders | Number | 2021 | 1234622.107 | 1671250.359 | 844463.0391 |
| DALYs | Global | Male | 70-74 years | Depressive disorders | Percent | 2021 | 0.006956141 | 0.009321292 | 0.004913047 |
| DALYs | Global | Female | 70-74 years | Depressive disorders | Percent | 2021 | 0.013524409 | 0.017689825 | 0.009772139 |
| DALYs | Global | Male | 70-74 years | Depressive disorders | Rate | 2021 | 794.883305 | 1083.140545 | 539.6980279 |
| DALYs | Global | Female | 70-74 years | Depressive disorders | Rate | 2021 | 1128.046444 | 1526.983855 | 771.566881 |
| DALYs | Global | Male | 75-79 years | Depressive disorders | Number | 2021 | 464436.0275 | 655435.1179 | 315131.934 |
| DALYs | Global | Female | 75-79 years | Depressive disorders | Number | 2021 | 772142.4134 | 1080771.497 | 525347.992 |
| DALYs | Global | Male | 75-79 years | Depressive disorders | Percent | 2021 | 0.005721658 | 0.00792123 | 0.003995734 |
| DALYs | Global | Female | 75-79 years | Depressive disorders | Percent | 2021 | 0.010584169 | 0.01417881 | 0.007498911 |
| DALYs | Global | Male | 75-79 years | Depressive disorders | Rate | 2021 | 776.8213456 | 1096.288746 | 527.0935037 |
| DALYs | Global | Female | 75-79 years | Depressive disorders | Rate | 2021 | 1070.967052 | 1499.037799 | 728.661424 |
| DALYs | Global | Male | 80-84 years | Depressive disorders | Number | 2021 | 270882.0145 | 385146.6439 | 184223.1943 |
| DALYs | Global | Female | 80-84 years | Depressive disorders | Number | 2021 | 512520.0315 | 729589.8139 | 346622.8556 |
| DALYs | Global | Male | 80-84 years | Depressive disorders | Percent | 2021 | 0.004482512 | 0.006287353 | 0.0030956 |
| DALYs | Global | Female | 80-84 years | Depressive disorders | Percent | 2021 | 0.00781874 | 0.010843386 | 0.005434402 |
| DALYs | Global | Male | 80-84 years | Depressive disorders | Rate | 2021 | 739.0675349 | 1050.824217 | 502.6298343 |
| DALYs | Global | Female | 80-84 years | Depressive disorders | Rate | 2021 | 1006.29886 | 1432.500883 | 680.5708321 |
| DALYs | Global | Male | 85-89 years | Depressive disorders | Number | 2021 | 120072.4513 | 166188.7386 | 84950.47709 |
| DALYs | Global | Female | 85-89 years | Depressive disorders | Number | 2021 | 269777.0199 | 375450.8509 | 190735.5396 |
| DALYs | Global | Male | 85-89 years | Depressive disorders | Percent | 2021 | 0.003283135 | 0.004512153 | 0.002372905 |
| DALYs | Global | Female | 85-89 years | Depressive disorders | Percent | 2021 | 0.006030939 | 0.008192701 | 0.004433658 |
| DALYs | Global | Male | 85-89 years | Depressive disorders | Rate | 2021 | 695.9648818 | 963.264467 | 492.3906201 |
| DALYs | Global | Female | 85-89 years | Depressive disorders | Rate | 2021 | 947.612397 | 1318.799803 | 669.9731574 |
| DALYs | Global | Male | 90-94 years | Depressive disorders | Number | 2021 | 38737.49552 | 55795.99761 | 26526.19598 |
| DALYs | Global | Female | 90-94 years | Depressive disorders | Number | 2021 | 109233.1177 | 156148.4376 | 74965.47667 |
| DALYs | Global | Male | 90-94 years | Depressive disorders | Percent | 2021 | 0.002466772 | 0.003539844 | 0.001727568 |
| DALYs | Global | Female | 90-94 years | Depressive disorders | Percent | 2021 | 0.004310752 | 0.006065639 | 0.003054971 |
| DALYs | Global | Male | 90-94 years | Depressive disorders | Rate | 2021 | 664.6221504 | 957.2961652 | 455.1119574 |
| DALYs | Global | Female | 90-94 years | Depressive disorders | Rate | 2021 | 905.6815661 | 1294.669277 | 621.5592097 |
| DALYs | Global | Male | 95+ years | Depressive disorders | Number | 2021 | 9589.865641 | 14456.0622 | 5781.703271 |
| DALYs | Global | Female | 95+ years | Depressive disorders | Number | 2021 | 34198.79466 | 51512.8483 | 20550.22421 |
| DALYs | Global | Male | 95+ years | Depressive disorders | Percent | 2021 | 0.002110841 | 0.003102826 | 0.001290448 |
| DALYs | Global | Female | 95+ years | Depressive disorders | Percent | 2021 | 0.003122124 | 0.004640412 | 0.00196364 |
| DALYs | Global | Male | 95+ years | Depressive disorders | Rate | 2021 | 634.2328051 | 956.0622871 | 382.3771908 |
| DALYs | Global | Female | 95+ years | Depressive disorders | Rate | 2021 | 868.371709 | 1308.008091 | 521.8088386 |
