# Supplementary material for: Unmasking the rising global burden of depression: A 32-year GBD analysis of gender disparities and regional hotspots in Sub-Saharan Africa
Source: PLoS One. 2025 Jul 31;20(7):e0326974. doi: 10.1371/journal.pone.0326974 (PMC12312894; doi:10.1371/journal.pone.0326974)
Supplement: S5 Table — (DOCX) [file pone.0326974.s004.docx]

| **Supplementary Table 5 Global and regional gender-age standardized depression incidence data (2021)** | | | | | | | | | |
| --- | --- | --- | --- | --- | --- | --- | --- | --- | --- |
| **measure** | **location** | **sex** | **age** | **cause** | **metric** | **year** | **value** | **upper** | **lower** |
| Incidence | Global | Male | Age-standardized | Depressive disorders | Rate | 2021 | 3366.269026 | 3958.071676 | 2922.760023 |
| Incidence | Global | Female | Age-standardized | Depressive disorders | Rate | 2021 | 5295.221944 | 6227.480135 | 4606.32092 |
| Incidence | East Asia | Male | Age-standardized | Depressive disorders | Rate | 2021 | 1753.211641 | 2036.426028 | 1540.704499 |
| Incidence | East Asia | Female | Age-standardized | Depressive disorders | Rate | 2021 | 2930.808545 | 3397.322336 | 2571.312511 |
| Incidence | Southeast Asia | Male | Age-standardized | Depressive disorders | Rate | 2021 | 2309.107994 | 2727.60199 | 1984.98643 |
| Incidence | Southeast Asia | Female | Age-standardized | Depressive disorders | Rate | 2021 | 2980.664586 | 3563.458826 | 2535.610604 |
| Incidence | Central Asia | Male | Age-standardized | Depressive disorders | Rate | 2021 | 3002.354701 | 3634.582797 | 2484.290637 |
| Incidence | Central Asia | Female | Age-standardized | Depressive disorders | Rate | 2021 | 5122.396051 | 6179.241613 | 4253.769466 |
| Incidence | Oceania | Male | Age-standardized | Depressive disorders | Rate | 2021 | 2716.446472 | 3442.31722 | 2111.553587 |
| Incidence | Oceania | Female | Age-standardized | Depressive disorders | Rate | 2021 | 3214.358707 | 4059.241144 | 2530.568771 |
| Incidence | High-income Asia Pacific | Male | Age-standardized | Depressive disorders | Rate | 2021 | 2245.590933 | 2632.375859 | 1943.270542 |
| Incidence | High-income Asia Pacific | Female | Age-standardized | Depressive disorders | Rate | 2021 | 3457.266227 | 4040.62393 | 2969.152842 |
| Incidence | Eastern Europe | Male | Age-standardized | Depressive disorders | Rate | 2021 | 4091.076602 | 4802.055988 | 3489.30534 |
| Incidence | Eastern Europe | Female | Age-standardized | Depressive disorders | Rate | 2021 | 5496.326714 | 6430.93902 | 4649.080813 |
| Incidence | Central Europe | Male | Age-standardized | Depressive disorders | Rate | 2021 | 2189.323295 | 2558.601801 | 1890.621481 |
| Incidence | Central Europe | Female | Age-standardized | Depressive disorders | Rate | 2021 | 4202.581865 | 4953.326955 | 3578.132372 |
| Incidence | Western Europe | Male | Age-standardized | Depressive disorders | Rate | 2021 | 3970.030903 | 4658.246453 | 3426.639411 |
| Incidence | Western Europe | Female | Age-standardized | Depressive disorders | Rate | 2021 | 7310.327423 | 8744.621917 | 6297.487325 |
| Incidence | Australasia | Male | Age-standardized | Depressive disorders | Rate | 2021 | 4457.439927 | 5657.954814 | 3538.251982 |
| Incidence | Australasia | Female | Age-standardized | Depressive disorders | Rate | 2021 | 6720.817033 | 8464.006698 | 5274.715421 |
| Incidence | Southern Latin America | Male | Age-standardized | Depressive disorders | Rate | 2021 | 2974.559573 | 3646.714251 | 2450.529396 |
| Incidence | Southern Latin America | Female | Age-standardized | Depressive disorders | Rate | 2021 | 5653.194558 | 7006.160818 | 4594.872385 |
| Incidence | High-income North America | Male | Age-standardized | Depressive disorders | Rate | 2021 | 4566.603313 | 5329.207453 | 3987.358004 |
| Incidence | High-income North America | Female | Age-standardized | Depressive disorders | Rate | 2021 | 8587.855525 | 9941.54154 | 7549.405302 |
| Incidence | Caribbean | Male | Age-standardized | Depressive disorders | Rate | 2021 | 3586.806874 | 4296.962236 | 2964.663669 |
| Incidence | Caribbean | Female | Age-standardized | Depressive disorders | Rate | 2021 | 6287.226438 | 7788.919618 | 5174.090195 |
| Incidence | Central Latin America | Male | Age-standardized | Depressive disorders | Rate | 2021 | 3276.26343 | 3812.923116 | 2855.723783 |
| Incidence | Central Latin America | Female | Age-standardized | Depressive disorders | Rate | 2021 | 5790.806581 | 6920.673457 | 4951.12463 |
| Incidence | Andean Latin America | Male | Age-standardized | Depressive disorders | Rate | 2021 | 2664.562866 | 3248.77488 | 2193.647939 |
| Incidence | Andean Latin America | Female | Age-standardized | Depressive disorders | Rate | 2021 | 4849.683866 | 6011.040214 | 3944.327362 |
| Incidence | North Africa and Middle East | Male | Age-standardized | Depressive disorders | Rate | 2021 | 4608.858737 | 5529.70456 | 3837.999828 |
| Incidence | North Africa and Middle East | Female | Age-standardized | Depressive disorders | Rate | 2021 | 7472.51949 | 9036.592358 | 6176.776795 |
| Incidence | Tropical Latin America | Male | Age-standardized | Depressive disorders | Rate | 2021 | 3318.98813 | 3880.260622 | 2877.026731 |
| Incidence | Tropical Latin America | Female | Age-standardized | Depressive disorders | Rate | 2021 | 7210.128853 | 8402.365266 | 6195.409841 |
| Incidence | South Asia | Male | Age-standardized | Depressive disorders | Rate | 2021 | 4258.431039 | 5002.088507 | 3698.26132 |
| Incidence | South Asia | Female | Age-standardized | Depressive disorders | Rate | 2021 | 6058.10997 | 7108.567855 | 5231.940456 |
| Incidence | Central Sub-Saharan Africa | Male | Age-standardized | Depressive disorders | Rate | 2021 | 6737.449843 | 8355.312984 | 5388.759167 |
| Incidence | Central Sub-Saharan Africa | Female | Age-standardized | Depressive disorders | Rate | 2021 | 8643.486013 | 10746.19516 | 6914.705879 |
| Incidence | Eastern Sub-Saharan Africa | Male | Age-standardized | Depressive disorders | Rate | 2021 | 5552.513434 | 6463.313651 | 4749.169991 |
| Incidence | Eastern Sub-Saharan Africa | Female | Age-standardized | Depressive disorders | Rate | 2021 | 7328.625272 | 8665.159731 | 6232.955732 |
| Incidence | Southern Sub-Saharan Africa | Male | Age-standardized | Depressive disorders | Rate | 2021 | 4674.496219 | 5494.332657 | 4040.094507 |
| Incidence | Southern Sub-Saharan Africa | Female | Age-standardized | Depressive disorders | Rate | 2021 | 6945.779312 | 8202.916721 | 5937.026588 |
| Incidence | Western Sub-Saharan Africa | Male | Age-standardized | Depressive disorders | Rate | 2021 | 3742.054324 | 4364.8165 | 3198.074644 |
| Incidence | Western Sub-Saharan Africa | Female | Age-standardized | Depressive disorders | Rate | 2021 | 5643.47897 | 6635.049587 | 4810.327083 |
