# Supplementary material for: Unmasking the rising global burden of depression: A 32-year GBD analysis of gender disparities and regional hotspots in Sub-Saharan Africa
Source: PLoS One. 2025 Jul 31;20(7):e0326974. doi: 10.1371/journal.pone.0326974 (PMC12312894; doi:10.1371/journal.pone.0326974)
Supplement: S6 Table — (DOCX) [file pone.0326974.s005.docx]

| **Supplementary Table 6 Global and regional gender-age standardized depression prevalence data (2021)** | | | | | | | | | |
| --- | --- | --- | --- | --- | --- | --- | --- | --- | --- |
| **measure** | **location** | **sex** | **age** | **cause** | **metric** | **year** | **value** | **upper** | **lower** |
| Prevalence | East Asia | Male | Age-standardized | Depressive disorders | Rate | 2021 | 2214.700207 | 2471.42389 | 1988.094128 |
| Prevalence | East Asia | Female | Age-standardized | Depressive disorders | Rate | 2021 | 3539.953516 | 3962.739642 | 3185.701153 |
| Prevalence | Global | Male | Age-standardized | Depressive disorders | Rate | 2021 | 3186.429231 | 3604.265597 | 2853.402954 |
| Prevalence | Global | Female | Age-standardized | Depressive disorders | Rate | 2021 | 4822.117157 | 5483.345259 | 4316.380501 |
| Prevalence | Oceania | Male | Age-standardized | Depressive disorders | Rate | 2021 | 2842.21942 | 3417.685774 | 2352.615518 |
| Prevalence | Oceania | Female | Age-standardized | Depressive disorders | Rate | 2021 | 3582.469991 | 4276.374723 | 2980.182049 |
| Prevalence | Central Asia | Male | Age-standardized | Depressive disorders | Rate | 2021 | 2925.25976 | 3415.626012 | 2513.946181 |
| Prevalence | Central Asia | Female | Age-standardized | Depressive disorders | Rate | 2021 | 4521.792108 | 5264.98056 | 3893.032054 |
| Prevalence | Southeast Asia | Male | Age-standardized | Depressive disorders | Rate | 2021 | 2559.827178 | 2917.713613 | 2260.249991 |
| Prevalence | Southeast Asia | Female | Age-standardized | Depressive disorders | Rate | 2021 | 3417.221832 | 3877.741365 | 3007.019543 |
| Prevalence | Australasia | Male | Age-standardized | Depressive disorders | Rate | 2021 | 3821.642039 | 4615.295666 | 3151.493632 |
| Prevalence | Australasia | Female | Age-standardized | Depressive disorders | Rate | 2021 | 5572.6471 | 6793.063617 | 4574.920745 |
| Prevalence | High-income Asia Pacific | Male | Age-standardized | Depressive disorders | Rate | 2021 | 2120.720871 | 2403.73522 | 1890.945421 |
| Prevalence | High-income Asia Pacific | Female | Age-standardized | Depressive disorders | Rate | 2021 | 2978.319656 | 3382.822324 | 2635.313605 |
| Prevalence | Central Europe | Male | Age-standardized | Depressive disorders | Rate | 2021 | 2385.164289 | 2723.346171 | 2102.78794 |
| Prevalence | Central Europe | Female | Age-standardized | Depressive disorders | Rate | 2021 | 3916.172573 | 4467.558301 | 3417.342568 |
| Prevalence | High-income North America | Male | Age-standardized | Depressive disorders | Rate | 2021 | 3853.577158 | 4326.389285 | 3449.860267 |
| Prevalence | High-income North America | Female | Age-standardized | Depressive disorders | Rate | 2021 | 6964.759086 | 7810.645384 | 6240.371576 |
| Prevalence | Southern Latin America | Male | Age-standardized | Depressive disorders | Rate | 2021 | 2689.531155 | 3159.197673 | 2272.596318 |
| Prevalence | Southern Latin America | Female | Age-standardized | Depressive disorders | Rate | 2021 | 4498.255469 | 5368.960247 | 3803.047145 |
| Prevalence | Tropical Latin America | Male | Age-standardized | Depressive disorders | Rate | 2021 | 2926.964932 | 3323.020806 | 2611.345323 |
| Prevalence | Tropical Latin America | Female | Age-standardized | Depressive disorders | Rate | 2021 | 5697.123557 | 6524.680229 | 5044.327193 |
| Prevalence | Eastern Europe | Male | Age-standardized | Depressive disorders | Rate | 2021 | 3629.694979 | 4105.138402 | 3212.526973 |
| Prevalence | Eastern Europe | Female | Age-standardized | Depressive disorders | Rate | 2021 | 4766.919472 | 5413.50237 | 4198.43779 |
| Prevalence | Western Europe | Male | Age-standardized | Depressive disorders | Rate | 2021 | 3600.283011 | 4146.273166 | 3164.088702 |
| Prevalence | Western Europe | Female | Age-standardized | Depressive disorders | Rate | 2021 | 5966.88002 | 6937.011278 | 5248.660731 |
| Prevalence | Central Sub-Saharan Africa | Male | Age-standardized | Depressive disorders | Rate | 2021 | 5510.254145 | 6694.304231 | 4525.61047 |
| Prevalence | Central Sub-Saharan Africa | Female | Age-standardized | Depressive disorders | Rate | 2021 | 7139.264587 | 8669.133785 | 5917.841411 |
| Prevalence | Central Latin America | Male | Age-standardized | Depressive disorders | Rate | 2021 | 2899.802811 | 3278.75294 | 2564.210791 |
| Prevalence | Central Latin America | Female | Age-standardized | Depressive disorders | Rate | 2021 | 4689.582788 | 5431.281643 | 4117.758642 |
| Prevalence | Andean Latin America | Male | Age-standardized | Depressive disorders | Rate | 2021 | 2520.385527 | 2919.093522 | 2161.151342 |
| Prevalence | Andean Latin America | Female | Age-standardized | Depressive disorders | Rate | 2021 | 4117.423034 | 4884.059952 | 3494.756171 |
| Prevalence | Caribbean | Male | Age-standardized | Depressive disorders | Rate | 2021 | 3136.855809 | 3674.87418 | 2707.328019 |
| Prevalence | Caribbean | Female | Age-standardized | Depressive disorders | Rate | 2021 | 5076.189311 | 6083.121912 | 4303.441351 |
| Prevalence | South Asia | Male | Age-standardized | Depressive disorders | Rate | 2021 | 3758.450554 | 4254.259934 | 3360.280421 |
| Prevalence | South Asia | Female | Age-standardized | Depressive disorders | Rate | 2021 | 5254.094942 | 5956.156839 | 4706.2708 |
| Prevalence | Western Sub-Saharan Africa | Male | Age-standardized | Depressive disorders | Rate | 2021 | 3516.206245 | 3975.605512 | 3120.078722 |
| Prevalence | Western Sub-Saharan Africa | Female | Age-standardized | Depressive disorders | Rate | 2021 | 5146.817732 | 5892.50614 | 4552.705024 |
| Prevalence | North Africa and Middle East | Male | Age-standardized | Depressive disorders | Rate | 2021 | 3949.571085 | 4569.665232 | 3445.978686 |
| Prevalence | North Africa and Middle East | Female | Age-standardized | Depressive disorders | Rate | 2021 | 6190.387904 | 7265.071115 | 5312.894264 |
| Prevalence | Eastern Sub-Saharan Africa | Male | Age-standardized | Depressive disorders | Rate | 2021 | 4695.967003 | 5381.085941 | 4166.734869 |
| Prevalence | Eastern Sub-Saharan Africa | Female | Age-standardized | Depressive disorders | Rate | 2021 | 6404.325485 | 7338.22534 | 5673.973756 |
| Prevalence | Southern Sub-Saharan Africa | Male | Age-standardized | Depressive disorders | Rate | 2021 | 4124.847352 | 4676.694976 | 3686.19511 |
| Prevalence | Southern Sub-Saharan Africa | Female | Age-standardized | Depressive disorders | Rate | 2021 | 5994.099984 | 6861.653931 | 5277.645608 |
