# Supplementary material for: Unmasking the rising global burden of depression: A 32-year GBD analysis of gender disparities and regional hotspots in Sub-Saharan Africa
Source: PLoS One. 2025 Jul 31;20(7):e0326974. doi: 10.1371/journal.pone.0326974 (PMC12312894; doi:10.1371/journal.pone.0326974)
Supplement: S7 Table — (DOCX) [file pone.0326974.s006.docx]

| **Supplementary Table 7 Global and regional gender-age standardized depression DALYsdata (2021)** | | | | | | | | | |
| --- | --- | --- | --- | --- | --- | --- | --- | --- | --- |
| **measure** | **location** | **sex** | **age** | **cause** | **metric** | **year** | **value** | **upper** | **lower** |
| DALYs | Central Asia | Male | Age-standardized | Depressive disorders | Rate | 2021 | 490.271535 | 673.6208591 | 336.2678954 |
| DALYs | Central Asia | Female | Age-standardized | Depressive disorders | Rate | 2021 | 780.1875365 | 1069.763847 | 531.4729212 |
| DALYs | Western Europe | Male | Age-standardized | Depressive disorders | Rate | 2021 | 630.8476716 | 854.464331 | 439.3350559 |
| DALYs | Western Europe | Female | Age-standardized | Depressive disorders | Rate | 2021 | 1087.540809 | 1479.483094 | 763.6396185 |
| DALYs | Eastern Europe | Male | Age-standardized | Depressive disorders | Rate | 2021 | 630.5715615 | 864.5546294 | 435.0704667 |
| DALYs | Eastern Europe | Female | Age-standardized | Depressive disorders | Rate | 2021 | 829.7733888 | 1131.812892 | 576.4557073 |
| DALYs | South Asia | Male | Age-standardized | Depressive disorders | Rate | 2021 | 654.1773099 | 889.077753 | 452.8108846 |
| DALYs | South Asia | Female | Age-standardized | Depressive disorders | Rate | 2021 | 903.5756691 | 1214.855329 | 631.3794525 |
| DALYs | Caribbean | Male | Age-standardized | Depressive disorders | Rate | 2021 | 551.5577434 | 766.5700553 | 377.8995327 |
| DALYs | Caribbean | Female | Age-standardized | Depressive disorders | Rate | 2021 | 918.7364731 | 1285.227463 | 623.0157654 |
| DALYs | Oceania | Male | Age-standardized | Depressive disorders | Rate | 2021 | 462.4413263 | 645.5362003 | 300.5549024 |
| DALYs | Oceania | Female | Age-standardized | Depressive disorders | Rate | 2021 | 556.4020844 | 767.1641731 | 360.4029779 |
| DALYs | Southern Sub-Saharan Africa | Male | Age-standardized | Depressive disorders | Rate | 2021 | 715.0266271 | 1000.392969 | 491.3091849 |
| DALYs | Southern Sub-Saharan Africa | Female | Age-standardized | Depressive disorders | Rate | 2021 | 1028.476153 | 1410.265862 | 714.8601728 |
| DALYs | Central Sub-Saharan Africa | Male | Age-standardized | Depressive disorders | Rate | 2021 | 999.6466815 | 1402.586771 | 662.5621121 |
| DALYs | Central Sub-Saharan Africa | Female | Age-standardized | Depressive disorders | Rate | 2021 | 1270.481797 | 1766.412574 | 847.4945661 |
| DALYs | Tropical Latin America | Male | Age-standardized | Depressive disorders | Rate | 2021 | 508.459149 | 686.7897856 | 348.4530373 |
| DALYs | Tropical Latin America | Female | Age-standardized | Depressive disorders | Rate | 2021 | 1037.31421 | 1415.316344 | 711.6910053 |
| DALYs | Central Latin America | Male | Age-standardized | Depressive disorders | Rate | 2021 | 505.2115334 | 691.7573477 | 345.1974663 |
| DALYs | Central Latin America | Female | Age-standardized | Depressive disorders | Rate | 2021 | 849.1932307 | 1167.570715 | 580.5746226 |
| DALYs | High-income North America | Male | Age-standardized | Depressive disorders | Rate | 2021 | 695.620045 | 939.0374056 | 481.3685437 |
| DALYs | High-income North America | Female | Age-standardized | Depressive disorders | Rate | 2021 | 1271.108615 | 1714.618984 | 890.8946957 |
| DALYs | Australasia | Male | Age-standardized | Depressive disorders | Rate | 2021 | 688.9526341 | 973.6247623 | 463.1413566 |
| DALYs | Australasia | Female | Age-standardized | Depressive disorders | Rate | 2021 | 1012.120418 | 1424.421638 | 674.0180574 |
| DALYs | Southern Latin America | Male | Age-standardized | Depressive disorders | Rate | 2021 | 470.6896296 | 656.1387885 | 315.3058171 |
| DALYs | Southern Latin America | Female | Age-standardized | Depressive disorders | Rate | 2021 | 830.3956776 | 1158.574848 | 553.563675 |
| DALYs | Andean Latin America | Male | Age-standardized | Depressive disorders | Rate | 2021 | 426.8727263 | 589.9913926 | 289.9050437 |
| DALYs | Andean Latin America | Female | Age-standardized | Depressive disorders | Rate | 2021 | 728.3776034 | 1015.544021 | 489.1268084 |
| DALYs | High-income Asia Pacific | Male | Age-standardized | Depressive disorders | Rate | 2021 | 365.229403 | 493.8662825 | 249.0204457 |
| DALYs | High-income Asia Pacific | Female | Age-standardized | Depressive disorders | Rate | 2021 | 532.3384995 | 725.6603568 | 365.9310425 |
| DALYs | Global | Male | Age-standardized | Depressive disorders | Rate | 2021 | 540.508822 | 735.4804758 | 377.3098289 |
| DALYs | Global | Female | Age-standardized | Depressive disorders | Rate | 2021 | 821.1650998 | 1110.42715 | 570.9587993 |
| DALYs | East Asia | Male | Age-standardized | Depressive disorders | Rate | 2021 | 329.0335163 | 448.8768401 | 231.7624502 |
| DALYs | East Asia | Female | Age-standardized | Depressive disorders | Rate | 2021 | 532.537016 | 722.4814945 | 377.0110384 |
| DALYs | Southeast Asia | Male | Age-standardized | Depressive disorders | Rate | 2021 | 406.3239085 | 554.0323351 | 281.7673201 |
| DALYs | Southeast Asia | Female | Age-standardized | Depressive disorders | Rate | 2021 | 528.2929975 | 718.7119116 | 365.7835058 |
| DALYs | Central Europe | Male | Age-standardized | Depressive disorders | Rate | 2021 | 377.4242645 | 510.7961309 | 256.1342591 |
| DALYs | Central Europe | Female | Age-standardized | Depressive disorders | Rate | 2021 | 658.5479592 | 899.5553387 | 453.4162133 |
| DALYs | North Africa and Middle East | Male | Age-standardized | Depressive disorders | Rate | 2021 | 709.7181049 | 974.2067897 | 478.7336645 |
| DALYs | North Africa and Middle East | Female | Age-standardized | Depressive disorders | Rate | 2021 | 1107.678235 | 1520.936082 | 728.8597683 |
| DALYs | Western Sub-Saharan Africa | Male | Age-standardized | Depressive disorders | Rate | 2021 | 594.2590093 | 809.7593689 | 403.0505249 |
| DALYs | Western Sub-Saharan Africa | Female | Age-standardized | Depressive disorders | Rate | 2021 | 865.379167 | 1175.494876 | 594.1464559 |
| DALYs | Eastern Sub-Saharan Africa | Male | Age-standardized | Depressive disorders | Rate | 2021 | 836.6682217 | 1125.833454 | 565.4349868 |
| DALYs | Eastern Sub-Saharan Africa | Female | Age-standardized | Depressive disorders | Rate | 2021 | 1104.493063 | 1472.248563 | 757.0501907 |
