# Supplementary material for: Unmasking the rising global burden of depression: A 32-year GBD analysis of gender disparities and regional hotspots in Sub-Saharan Africa
Source: PLoS One. 2025 Jul 31;20(7):e0326974. doi: 10.1371/journal.pone.0326974 (PMC12312894; doi:10.1371/journal.pone.0326974)
Supplement: S8 Table — (DOCX) [file pone.0326974.s007.docx]

| **Supplementary Table 8 Global and Regional Age-Standardized Depression Incidence Temporal Trend Data (2021)** | | | | | | | | | |
| --- | --- | --- | --- | --- | --- | --- | --- | --- | --- |
| **measure** | **location** | **sex** | **age** | **cause** | **metric** | **year** | **value** | **upper** | **lower** |
| Incidence | Global | Both | Age-standardized | Depressive disorders | Rate | 2021 | 4333.617222 | 5093.613992 | 3770.796473 |
| Incidence | East Asia | Both | Age-standardized | Depressive disorders | Rate | 2021 | 2337.695813 | 2718.381011 | 2058.014722 |
| Incidence | Southeast Asia | Both | Age-standardized | Depressive disorders | Rate | 2021 | 2646.506877 | 3152.367135 | 2262.278495 |
| Incidence | Central Asia | Both | Age-standardized | Depressive disorders | Rate | 2021 | 4131.919109 | 4952.485645 | 3444.953841 |
| Incidence | Oceania | Both | Age-standardized | Depressive disorders | Rate | 2021 | 2956.632109 | 3768.079016 | 2307.715045 |
| Incidence | High-income Asia Pacific | Both | Age-standardized | Depressive disorders | Rate | 2021 | 2845.861406 | 3326.64608 | 2452.418247 |
| Incidence | Eastern Europe | Both | Age-standardized | Depressive disorders | Rate | 2021 | 4833.938495 | 5669.692664 | 4115.802244 |
| Incidence | Central Europe | Both | Age-standardized | Depressive disorders | Rate | 2021 | 3225.415075 | 3788.20978 | 2763.062153 |
| Incidence | Western Europe | Both | Age-standardized | Depressive disorders | Rate | 2021 | 5634.45546 | 6686.608852 | 4852.738879 |
| Incidence | Australasia | Both | Age-standardized | Depressive disorders | Rate | 2021 | 5579.699502 | 7067.861879 | 4387.273384 |
| Incidence | Southern Latin America | Both | Age-standardized | Depressive disorders | Rate | 2021 | 4330.317772 | 5341.733585 | 3543.253346 |
| Incidence | High-income North America | Both | Age-standardized | Depressive disorders | Rate | 2021 | 6572.237183 | 7626.112583 | 5787.265286 |
| Incidence | Caribbean | Both | Age-standardized | Depressive disorders | Rate | 2021 | 4956.525321 | 6075.476881 | 4094.79706 |
| Incidence | Central Latin America | Both | Age-standardized | Depressive disorders | Rate | 2021 | 4574.680341 | 5407.859223 | 3953.812701 |
| Incidence | Andean Latin America | Both | Age-standardized | Depressive disorders | Rate | 2021 | 3764.829728 | 4617.212391 | 3106.999389 |
| Incidence | North Africa and Middle East | Both | Age-standardized | Depressive disorders | Rate | 2021 | 5983.104933 | 7214.680169 | 4953.301373 |
| Incidence | Tropical Latin America | Both | Age-standardized | Depressive disorders | Rate | 2021 | 5317.594576 | 6168.766832 | 4592.415857 |
| Incidence | South Asia | Both | Age-standardized | Depressive disorders | Rate | 2021 | 5150.99858 | 6036.893936 | 4461.449688 |
| Incidence | Central Sub-Saharan Africa | Both | Age-standardized | Depressive disorders | Rate | 2021 | 7703.41432 | 9565.937104 | 6194.217846 |
| Incidence | Eastern Sub-Saharan Africa | Both | Age-standardized | Depressive disorders | Rate | 2021 | 6468.120973 | 7580.312231 | 5519.803186 |
| Incidence | Southern Sub-Saharan Africa | Both | Age-standardized | Depressive disorders | Rate | 2021 | 5878.90535 | 6920.415667 | 5041.115985 |
| Incidence | Western Sub-Saharan Africa | Both | Age-standardized | Depressive disorders | Rate | 2021 | 4739.638423 | 5556.82845 | 4046.237948 |
