# Supplementary material for: Unmasking the rising global burden of depression: A 32-year GBD analysis of gender disparities and regional hotspots in Sub-Saharan Africa
Source: PLoS One. 2025 Jul 31;20(7):e0326974. doi: 10.1371/journal.pone.0326974 (PMC12312894; doi:10.1371/journal.pone.0326974)
Supplement: S9 Table — (DOCX) [file pone.0326974.s008.docx]

| **Supplementary Table 9 Global and Regional Age-Standardized Depression Prevalence Temporal Trend Data (2021)** | | | | | | | | | |
| --- | --- | --- | --- | --- | --- | --- | --- | --- | --- |
| **measure** | **location** | **sex** | **age** | **cause** | **metric** | **year** | **value** | **upper** | **lower** |
| Prevalence | East Asia | Both | Age-standardized | Depressive disorders | Rate | 2021 | 2870.607766 | 3205.354485 | 2583.726598 |
| Prevalence | Oceania | Both | Age-standardized | Depressive disorders | Rate | 2021 | 3201.990236 | 3811.000277 | 2654.700954 |
| Prevalence | Global | Both | Age-standardized | Depressive disorders | Rate | 2021 | 4006.822667 | 4539.010321 | 3581.258396 |
| Prevalence | High-income Asia Pacific | Both | Age-standardized | Depressive disorders | Rate | 2021 | 2545.156188 | 2892.376535 | 2266.732892 |
| Prevalence | Central Asia | Both | Age-standardized | Depressive disorders | Rate | 2021 | 3773.706559 | 4386.790191 | 3243.382033 |
| Prevalence | Southeast Asia | Both | Age-standardized | Depressive disorders | Rate | 2021 | 2991.563797 | 3401.761331 | 2647.86478 |
| Prevalence | Australasia | Both | Age-standardized | Depressive disorders | Rate | 2021 | 4691.879978 | 5719.886801 | 3904.487911 |
| Prevalence | Central Europe | Both | Age-standardized | Depressive disorders | Rate | 2021 | 3171.900109 | 3611.516498 | 2789.030359 |
| Prevalence | Eastern Europe | Both | Age-standardized | Depressive disorders | Rate | 2021 | 4231.790631 | 4771.111187 | 3729.903861 |
| Prevalence | High-income North America | Both | Age-standardized | Depressive disorders | Rate | 2021 | 5408.261749 | 6049.723387 | 4846.89577 |
| Prevalence | Tropical Latin America | Both | Age-standardized | Depressive disorders | Rate | 2021 | 4352.08785 | 4948.916093 | 3871.002837 |
| Prevalence | Southern Latin America | Both | Age-standardized | Depressive disorders | Rate | 2021 | 3605.125044 | 4246.275133 | 3048.17954 |
| Prevalence | Western Europe | Both | Age-standardized | Depressive disorders | Rate | 2021 | 4778.950481 | 5528.960001 | 4207.886496 |
| Prevalence | Central Sub-Saharan Africa | Both | Age-standardized | Depressive disorders | Rate | 2021 | 6337.030541 | 7669.980773 | 5236.437556 |
| Prevalence | Central Latin America | Both | Age-standardized | Depressive disorders | Rate | 2021 | 3825.604032 | 4375.351881 | 3399.906076 |
| Prevalence | North Africa and Middle East | Both | Age-standardized | Depressive disorders | Rate | 2021 | 5024.694477 | 5857.37982 | 4346.394973 |
| Prevalence | Caribbean | Both | Age-standardized | Depressive disorders | Rate | 2021 | 4121.501704 | 4870.279431 | 3512.088316 |
| Prevalence | Western Sub-Saharan Africa | Both | Age-standardized | Depressive disorders | Rate | 2021 | 4372.187964 | 4963.050446 | 3880.291031 |
| Prevalence | Andean Latin America | Both | Age-standardized | Depressive disorders | Rate | 2021 | 3325.811476 | 3880.446 | 2851.295192 |
| Prevalence | Eastern Sub-Saharan Africa | Both | Age-standardized | Depressive disorders | Rate | 2021 | 5576.415475 | 6372.719537 | 4939.635877 |
| Prevalence | South Asia | Both | Age-standardized | Depressive disorders | Rate | 2021 | 4500.484212 | 5106.633364 | 4034.926777 |
| Prevalence | Southern Sub-Saharan Africa | Both | Age-standardized | Depressive disorders | Rate | 2021 | 5113.034266 | 5818.203593 | 4540.526393 |
