# Supplementary material for: Unmasking the rising global burden of depression: A 32-year GBD analysis of gender disparities and regional hotspots in Sub-Saharan Africa
Source: PLoS One. 2025 Jul 31;20(7):e0326974. doi: 10.1371/journal.pone.0326974 (PMC12312894; doi:10.1371/journal.pone.0326974)
Supplement: S10 Table — (DOCX) [file pone.0326974.s009.docx]

| **Supplementary Table 10 Global and Regional Age-Standardized Depression DALYs Temporal Trend Data (2021)** | | | | | | | | | |
| --- | --- | --- | --- | --- | --- | --- | --- | --- | --- |
| **measure** | **location** | **sex** | **age** | **cause** | **metric** | **year** | **value** | **upper** | **lower** |
| DALYs | Central Asia | Both | Age-standardized | Depressive disorders | Rate | 2021 | 644.2781469 | 883.5587875 | 441.8339362 |
| DALYs | Western Europe | Both | Age-standardized | Depressive disorders | Rate | 2021 | 858.1624201 | 1164.615743 | 600.3664055 |
| DALYs | Eastern Europe | Both | Age-standardized | Depressive disorders | Rate | 2021 | 735.8342749 | 1005.599558 | 510.5775931 |
| DALYs | South Asia | Both | Age-standardized | Depressive disorders | Rate | 2021 | 777.7950463 | 1049.654105 | 542.5608142 |
| DALYs | Caribbean | Both | Age-standardized | Depressive disorders | Rate | 2021 | 737.8083433 | 1028.878141 | 507.6419274 |
| DALYs | Oceania | Both | Age-standardized | Depressive disorders | Rate | 2021 | 507.9046168 | 704.0350686 | 329.2026797 |
| DALYs | Southern Sub-Saharan Africa | Both | Age-standardized | Depressive disorders | Rate | 2021 | 880.6939773 | 1219.629846 | 609.0909378 |
| DALYs | Central Sub-Saharan Africa | Both | Age-standardized | Depressive disorders | Rate | 2021 | 1136.909012 | 1588.383818 | 756.8827797 |
| DALYs | Tropical Latin America | Both | Age-standardized | Depressive disorders | Rate | 2021 | 780.1498801 | 1062.15175 | 539.0607293 |
| DALYs | Central Latin America | Both | Age-standardized | Depressive disorders | Rate | 2021 | 682.9349609 | 935.0172863 | 468.2830689 |
| DALYs | High-income North America | Both | Age-standardized | Depressive disorders | Rate | 2021 | 982.776437 | 1322.412345 | 685.3341279 |
| DALYs | Australasia | Both | Age-standardized | Depressive disorders | Rate | 2021 | 849.3108817 | 1201.644152 | 569.1345866 |
| DALYs | Southern Latin America | Both | Age-standardized | Depressive disorders | Rate | 2021 | 652.6160483 | 904.8159892 | 439.6207463 |
| DALYs | Andean Latin America | Both | Age-standardized | Depressive disorders | Rate | 2021 | 578.7370473 | 804.9708557 | 389.3150346 |
| DALYs | High-income Asia Pacific | Both | Age-standardized | Depressive disorders | Rate | 2021 | 447.8641967 | 606.649809 | 307.9948624 |
| DALYs | Global | Both | Age-standardized | Depressive disorders | Rate | 2021 | 681.1421969 | 923.8253658 | 475.1893022 |
| DALYs | East Asia | Both | Age-standardized | Depressive disorders | Rate | 2021 | 429.6691133 | 585.4152713 | 304.2511206 |
| DALYs | Southeast Asia | Both | Age-standardized | Depressive disorders | Rate | 2021 | 467.6284287 | 634.8758512 | 323.7427426 |
| DALYs | Central Europe | Both | Age-standardized | Depressive disorders | Rate | 2021 | 521.7481851 | 710.0964912 | 358.8027634 |
| DALYs | North Africa and Middle East | Both | Age-standardized | Depressive disorders | Rate | 2021 | 900.6785672 | 1242.727109 | 598.4567371 |
| DALYs | Western Sub-Saharan Africa | Both | Age-standardized | Depressive disorders | Rate | 2021 | 736.5297464 | 1002.773451 | 502.6058301 |
| DALYs | Eastern Sub-Saharan Africa | Both | Age-standardized | Depressive disorders | Rate | 2021 | 974.6488995 | 1308.671338 | 668.6723228 |
