# Supplementary material for: Unmasking the rising global burden of depression: A 32-year GBD analysis of gender disparities and regional hotspots in Sub-Saharan Africa
Source: PLoS One. 2025 Jul 31;20(7):e0326974. doi: 10.1371/journal.pone.0326974 (PMC12312894; doi:10.1371/journal.pone.0326974)
Supplement: S11 Table — (DOCX) [file pone.0326974.s010.docx]

| **Supplementary Table 11 Global and Regional Time Series Data on Depression Incidence (1990-2021)** | | | | | | | | | |
| --- | --- | --- | --- | --- | --- | --- | --- | --- | --- |
| **measure** | **location** | **sex** | **age** | **cause** | **metric** | **year** | **value** | **upper** | **lower** |
| Incidence | East Asia | Both | Age-standardized | Depressive disorders | Rate | 1990 | 2610.704484 | 2995.190707 | 2296.983013 |
| Incidence | East Asia | Both | Age-standardized | Depressive disorders | Rate | 1991 | 2692.723107 | 3081.904445 | 2380.267391 |
| Incidence | East Asia | Both | Age-standardized | Depressive disorders | Rate | 1992 | 2756.092627 | 3146.358494 | 2444.03125 |
| Incidence | East Asia | Both | Age-standardized | Depressive disorders | Rate | 1993 | 2799.338591 | 3191.686617 | 2480.315313 |
| Incidence | East Asia | Both | Age-standardized | Depressive disorders | Rate | 1994 | 2820.719568 | 3214.354082 | 2496.112645 |
| Incidence | East Asia | Both | Age-standardized | Depressive disorders | Rate | 1995 | 2818.802296 | 3211.018096 | 2495.441151 |
| Incidence | East Asia | Both | Age-standardized | Depressive disorders | Rate | 1996 | 2780.849574 | 3153.701702 | 2464.099738 |
| Incidence | East Asia | Both | Age-standardized | Depressive disorders | Rate | 1997 | 2709.057078 | 3057.559027 | 2404.796929 |
| Incidence | East Asia | Both | Age-standardized | Depressive disorders | Rate | 1998 | 2624.625239 | 2952.743905 | 2336.94721 |
| Incidence | East Asia | Both | Age-standardized | Depressive disorders | Rate | 1999 | 2548.519295 | 2858.194634 | 2274.540354 |
| Incidence | East Asia | Both | Age-standardized | Depressive disorders | Rate | 2000 | 2501.819011 | 2798.396939 | 2236.830689 |
| Incidence | East Asia | Both | Age-standardized | Depressive disorders | Rate | 2001 | 2482.814814 | 2780.402474 | 2219.174679 |
| Incidence | East Asia | Both | Age-standardized | Depressive disorders | Rate | 2002 | 2472.397247 | 2771.864199 | 2209.007996 |
| Incidence | East Asia | Both | Age-standardized | Depressive disorders | Rate | 2003 | 2465.688532 | 2765.62989 | 2201.98075 |
| Incidence | East Asia | Both | Age-standardized | Depressive disorders | Rate | 2004 | 2457.583245 | 2756.607969 | 2194.621669 |
| Incidence | East Asia | Both | Age-standardized | Depressive disorders | Rate | 2005 | 2443.079736 | 2739.390421 | 2181.505728 |
| Incidence | East Asia | Both | Age-standardized | Depressive disorders | Rate | 2006 | 2413.898397 | 2706.895867 | 2158.746729 |
| Incidence | East Asia | Both | Age-standardized | Depressive disorders | Rate | 2007 | 2372.436835 | 2657.298668 | 2127.061306 |
| Incidence | East Asia | Both | Age-standardized | Depressive disorders | Rate | 2008 | 2329.932389 | 2604.825175 | 2094.660545 |
| Incidence | East Asia | Both | Age-standardized | Depressive disorders | Rate | 2009 | 2297.455058 | 2564.229323 | 2066.754645 |
| Incidence | East Asia | Both | Age-standardized | Depressive disorders | Rate | 2010 | 2286.127616 | 2550.298083 | 2057.354514 |
| Incidence | East Asia | Both | Age-standardized | Depressive disorders | Rate | 2011 | 2301.918957 | 2574.483032 | 2067.557974 |
| Incidence | East Asia | Both | Age-standardized | Depressive disorders | Rate | 2012 | 2335.390218 | 2622.17833 | 2091.188859 |
| Incidence | East Asia | Both | Age-standardized | Depressive disorders | Rate | 2013 | 2374.68515 | 2673.737288 | 2118.875582 |
| Incidence | East Asia | Both | Age-standardized | Depressive disorders | Rate | 2014 | 2407.828699 | 2715.339973 | 2146.498291 |
| Incidence | East Asia | Both | Age-standardized | Depressive disorders | Rate | 2015 | 2423.060697 | 2741.014772 | 2158.064041 |
| Incidence | East Asia | Both | Age-standardized | Depressive disorders | Rate | 2016 | 2419.030743 | 2736.634667 | 2152.180238 |
| Incidence | East Asia | Both | Age-standardized | Depressive disorders | Rate | 2017 | 2406.643617 | 2727.455301 | 2136.388753 |
| Incidence | East Asia | Both | Age-standardized | Depressive disorders | Rate | 2018 | 2392.744534 | 2712.406183 | 2126.475611 |
| Incidence | East Asia | Both | Age-standardized | Depressive disorders | Rate | 2019 | 2383.988516 | 2709.039944 | 2116.69723 |
| Incidence | East Asia | Both | Age-standardized | Depressive disorders | Rate | 2020 | 2475.146134 | 2875.012697 | 2177.296727 |
| Incidence | East Asia | Both | Age-standardized | Depressive disorders | Rate | 2021 | 2337.695813 | 2718.381011 | 2058.014722 |
| Incidence | Global | Both | Age-standardized | Depressive disorders | Rate | 1990 | 3748.490177 | 4353.004299 | 3292.729979 |
| Incidence | Global | Both | Age-standardized | Depressive disorders | Rate | 1991 | 3794.765592 | 4397.368346 | 3338.435384 |
| Incidence | Global | Both | Age-standardized | Depressive disorders | Rate | 1992 | 3835.369611 | 4435.836006 | 3378.682921 |
| Incidence | Global | Both | Age-standardized | Depressive disorders | Rate | 1993 | 3868.140922 | 4456.217479 | 3415.354379 |
| Incidence | Global | Both | Age-standardized | Depressive disorders | Rate | 1994 | 3890.998705 | 4469.360302 | 3438.339142 |
| Incidence | Global | Both | Age-standardized | Depressive disorders | Rate | 1995 | 3902.293504 | 4474.827017 | 3455.622503 |
| Incidence | Global | Both | Age-standardized | Depressive disorders | Rate | 1996 | 3905.34147 | 4474.385851 | 3460.797145 |
| Incidence | Global | Both | Age-standardized | Depressive disorders | Rate | 1997 | 3904.079451 | 4468.444058 | 3463.316179 |
| Incidence | Global | Both | Age-standardized | Depressive disorders | Rate | 1998 | 3900.108275 | 4457.971892 | 3463.615158 |
| Incidence | Global | Both | Age-standardized | Depressive disorders | Rate | 1999 | 3895.436267 | 4447.723891 | 3462.936541 |
| Incidence | Global | Both | Age-standardized | Depressive disorders | Rate | 2000 | 3891.878713 | 4439.665804 | 3461.374737 |
| Incidence | Global | Both | Age-standardized | Depressive disorders | Rate | 2001 | 3892.486582 | 4442.998947 | 3462.829427 |
| Incidence | Global | Both | Age-standardized | Depressive disorders | Rate | 2002 | 3896.034764 | 4450.157307 | 3466.247759 |
| Incidence | Global | Both | Age-standardized | Depressive disorders | Rate | 2003 | 3899.981825 | 4458.759432 | 3469.711831 |
| Incidence | Global | Both | Age-standardized | Depressive disorders | Rate | 2004 | 3901.631686 | 4463.967445 | 3471.160554 |
| Incidence | Global | Both | Age-standardized | Depressive disorders | Rate | 2005 | 3897.978769 | 4462.288188 | 3469.220707 |
| Incidence | Global | Both | Age-standardized | Depressive disorders | Rate | 2006 | 3865.741453 | 4420.867837 | 3446.999909 |
| Incidence | Global | Both | Age-standardized | Depressive disorders | Rate | 2007 | 3797.997238 | 4335.621272 | 3394.312393 |
| Incidence | Global | Both | Age-standardized | Depressive disorders | Rate | 2008 | 3718.676137 | 4236.981467 | 3326.238288 |
| Incidence | Global | Both | Age-standardized | Depressive disorders | Rate | 2009 | 3652.353494 | 4154.182224 | 3274.9385 |
| Incidence | Global | Both | Age-standardized | Depressive disorders | Rate | 2010 | 3623.673932 | 4116.755724 | 3254.340901 |
| Incidence | Global | Both | Age-standardized | Depressive disorders | Rate | 2011 | 3626.207543 | 4125.788296 | 3246.433923 |
| Incidence | Global | Both | Age-standardized | Depressive disorders | Rate | 2012 | 3634.177916 | 4143.844245 | 3249.321368 |
| Incidence | Global | Both | Age-standardized | Depressive disorders | Rate | 2013 | 3645.192162 | 4165.707935 | 3249.293506 |
| Incidence | Global | Both | Age-standardized | Depressive disorders | Rate | 2014 | 3656.998146 | 4187.517683 | 3248.171549 |
| Incidence | Global | Both | Age-standardized | Depressive disorders | Rate | 2015 | 3666.784483 | 4207.478894 | 3243.932163 |
| Incidence | Global | Both | Age-standardized | Depressive disorders | Rate | 2016 | 3674.036356 | 4241.325513 | 3235.031971 |
| Incidence | Global | Both | Age-standardized | Depressive disorders | Rate | 2017 | 3680.128874 | 4266.851575 | 3224.436836 |
| Incidence | Global | Both | Age-standardized | Depressive disorders | Rate | 2018 | 3685.963568 | 4298.352895 | 3220.966907 |
| Incidence | Global | Both | Age-standardized | Depressive disorders | Rate | 2019 | 3692.495468 | 4328.389848 | 3224.730621 |
| Incidence | Global | Both | Age-standardized | Depressive disorders | Rate | 2020 | 4310.823693 | 5053.945742 | 3751.802824 |
| Incidence | Global | Both | Age-standardized | Depressive disorders | Rate | 2021 | 4333.617222 | 5093.613992 | 3770.796473 |
| Incidence | Southeast Asia | Both | Age-standardized | Depressive disorders | Rate | 1990 | 2255.427982 | 2659.946772 | 1959.225631 |
| Incidence | Southeast Asia | Both | Age-standardized | Depressive disorders | Rate | 1991 | 2245.158628 | 2640.059466 | 1957.389708 |
| Incidence | Southeast Asia | Both | Age-standardized | Depressive disorders | Rate | 1992 | 2234.585375 | 2619.430217 | 1950.632643 |
| Incidence | Southeast Asia | Both | Age-standardized | Depressive disorders | Rate | 1993 | 2224.343718 | 2600.697782 | 1943.160719 |
| Incidence | Southeast Asia | Both | Age-standardized | Depressive disorders | Rate | 1994 | 2214.946901 | 2585.955394 | 1939.006841 |
| Incidence | Southeast Asia | Both | Age-standardized | Depressive disorders | Rate | 1995 | 2206.883454 | 2567.709901 | 1936.036518 |
| Incidence | Southeast Asia | Both | Age-standardized | Depressive disorders | Rate | 1996 | 2195.877549 | 2553.335573 | 1926.46524 |
| Incidence | Southeast Asia | Both | Age-standardized | Depressive disorders | Rate | 1997 | 2179.654413 | 2527.044249 | 1912.187184 |
| Incidence | Southeast Asia | Both | Age-standardized | Depressive disorders | Rate | 1998 | 2161.85143 | 2506.05172 | 1897.740717 |
| Incidence | Southeast Asia | Both | Age-standardized | Depressive disorders | Rate | 1999 | 2146.035003 | 2481.413045 | 1884.81583 |
| Incidence | Southeast Asia | Both | Age-standardized | Depressive disorders | Rate | 2000 | 2135.953496 | 2462.259556 | 1879.209463 |
| Incidence | Southeast Asia | Both | Age-standardized | Depressive disorders | Rate | 2001 | 2132.657011 | 2456.439179 | 1877.736143 |
| Incidence | Southeast Asia | Both | Age-standardized | Depressive disorders | Rate | 2002 | 2133.153707 | 2458.89293 | 1879.728789 |
| Incidence | Southeast Asia | Both | Age-standardized | Depressive disorders | Rate | 2003 | 2135.337657 | 2463.248905 | 1881.354064 |
| Incidence | Southeast Asia | Both | Age-standardized | Depressive disorders | Rate | 2004 | 2136.910769 | 2464.540631 | 1882.415744 |
| Incidence | Southeast Asia | Both | Age-standardized | Depressive disorders | Rate | 2005 | 2135.396529 | 2460.898418 | 1882.833074 |
| Incidence | Southeast Asia | Both | Age-standardized | Depressive disorders | Rate | 2006 | 2128.305248 | 2454.512445 | 1878.134099 |
| Incidence | Southeast Asia | Both | Age-standardized | Depressive disorders | Rate | 2007 | 2116.832105 | 2441.842247 | 1870.039209 |
| Incidence | Southeast Asia | Both | Age-standardized | Depressive disorders | Rate | 2008 | 2104.794936 | 2426.828823 | 1861.283812 |
| Incidence | Southeast Asia | Both | Age-standardized | Depressive disorders | Rate | 2009 | 2095.155262 | 2412.301555 | 1852.589836 |
| Incidence | Southeast Asia | Both | Age-standardized | Depressive disorders | Rate | 2010 | 2089.774828 | 2405.09495 | 1848.409021 |
| Incidence | Southeast Asia | Both | Age-standardized | Depressive disorders | Rate | 2011 | 2090.489792 | 2411.789548 | 1845.889381 |
| Incidence | Southeast Asia | Both | Age-standardized | Depressive disorders | Rate | 2012 | 2096.676322 | 2426.394843 | 1852.904546 |
| Incidence | Southeast Asia | Both | Age-standardized | Depressive disorders | Rate | 2013 | 2106.044411 | 2441.374097 | 1855.971261 |
| Incidence | Southeast Asia | Both | Age-standardized | Depressive disorders | Rate | 2014 | 2115.959644 | 2452.251096 | 1857.64228 |
| Incidence | Southeast Asia | Both | Age-standardized | Depressive disorders | Rate | 2015 | 2124.126508 | 2463.916759 | 1860.132993 |
| Incidence | Southeast Asia | Both | Age-standardized | Depressive disorders | Rate | 2016 | 2133.941247 | 2480.899031 | 1866.398244 |
| Incidence | Southeast Asia | Both | Age-standardized | Depressive disorders | Rate | 2017 | 2147.371888 | 2502.47394 | 1874.029827 |
| Incidence | Southeast Asia | Both | Age-standardized | Depressive disorders | Rate | 2018 | 2159.67405 | 2521.412857 | 1882.278617 |
| Incidence | Southeast Asia | Both | Age-standardized | Depressive disorders | Rate | 2019 | 2165.993513 | 2536.693515 | 1884.099849 |
| Incidence | Southeast Asia | Both | Age-standardized | Depressive disorders | Rate | 2020 | 2409.838283 | 2850.596972 | 2064.027383 |
| Incidence | Southeast Asia | Both | Age-standardized | Depressive disorders | Rate | 2021 | 2646.506877 | 3152.367135 | 2262.278495 |
| Incidence | Oceania | Both | Age-standardized | Depressive disorders | Rate | 1990 | 2824.745584 | 3378.558828 | 2404.361029 |
| Incidence | Oceania | Both | Age-standardized | Depressive disorders | Rate | 1991 | 2818.475849 | 3359.758128 | 2409.297495 |
| Incidence | Oceania | Both | Age-standardized | Depressive disorders | Rate | 1992 | 2813.001419 | 3347.03194 | 2407.179089 |
| Incidence | Oceania | Both | Age-standardized | Depressive disorders | Rate | 1993 | 2808.14848 | 3344.152994 | 2412.052355 |
| Incidence | Oceania | Both | Age-standardized | Depressive disorders | Rate | 1994 | 2804.100368 | 3330.832181 | 2405.753547 |
| Incidence | Oceania | Both | Age-standardized | Depressive disorders | Rate | 1995 | 2801.045717 | 3313.283113 | 2394.697756 |
| Incidence | Oceania | Both | Age-standardized | Depressive disorders | Rate | 1996 | 2797.756997 | 3315.444855 | 2392.699768 |
| Incidence | Oceania | Both | Age-standardized | Depressive disorders | Rate | 1997 | 2793.568351 | 3321.628596 | 2390.665859 |
| Incidence | Oceania | Both | Age-standardized | Depressive disorders | Rate | 1998 | 2789.025485 | 3329.846304 | 2391.700441 |
| Incidence | Oceania | Both | Age-standardized | Depressive disorders | Rate | 1999 | 2785.195346 | 3336.553562 | 2385.489014 |
| Incidence | Oceania | Both | Age-standardized | Depressive disorders | Rate | 2000 | 2783.264755 | 3348.78313 | 2382.980477 |
| Incidence | Oceania | Both | Age-standardized | Depressive disorders | Rate | 2001 | 2783.956729 | 3347.087386 | 2382.830969 |
| Incidence | Oceania | Both | Age-standardized | Depressive disorders | Rate | 2002 | 2786.241357 | 3348.580384 | 2382.597405 |
| Incidence | Oceania | Both | Age-standardized | Depressive disorders | Rate | 2003 | 2788.537311 | 3342.347736 | 2383.175393 |
| Incidence | Oceania | Both | Age-standardized | Depressive disorders | Rate | 2004 | 2789.644156 | 3324.031099 | 2382.973123 |
| Incidence | Oceania | Both | Age-standardized | Depressive disorders | Rate | 2005 | 2788.725435 | 3315.321532 | 2379.613359 |
| Incidence | Oceania | Both | Age-standardized | Depressive disorders | Rate | 2006 | 2781.291395 | 3304.22611 | 2375.864873 |
| Incidence | Oceania | Both | Age-standardized | Depressive disorders | Rate | 2007 | 2766.52421 | 3285.064238 | 2374.638164 |
| Incidence | Oceania | Both | Age-standardized | Depressive disorders | Rate | 2008 | 2749.196165 | 3259.570262 | 2371.049109 |
| Incidence | Oceania | Both | Age-standardized | Depressive disorders | Rate | 2009 | 2734.140554 | 3228.957307 | 2367.48614 |
| Incidence | Oceania | Both | Age-standardized | Depressive disorders | Rate | 2010 | 2726.37723 | 3213.253113 | 2350.850443 |
| Incidence | Oceania | Both | Age-standardized | Depressive disorders | Rate | 2011 | 2725.282835 | 3232.999794 | 2349.066072 |
| Incidence | Oceania | Both | Age-standardized | Depressive disorders | Rate | 2012 | 2726.162034 | 3247.268619 | 2348.03011 |
| Incidence | Oceania | Both | Age-standardized | Depressive disorders | Rate | 2013 | 2728.079962 | 3263.697555 | 2348.512256 |
| Incidence | Oceania | Both | Age-standardized | Depressive disorders | Rate | 2014 | 2730.163902 | 3264.882614 | 2339.14986 |
| Incidence | Oceania | Both | Age-standardized | Depressive disorders | Rate | 2015 | 2731.615793 | 3260.833779 | 2324.95376 |
| Incidence | Oceania | Both | Age-standardized | Depressive disorders | Rate | 2016 | 2732.238465 | 3259.944095 | 2330.432873 |
| Incidence | Oceania | Both | Age-standardized | Depressive disorders | Rate | 2017 | 2732.394911 | 3265.698989 | 2325.311234 |
| Incidence | Oceania | Both | Age-standardized | Depressive disorders | Rate | 2018 | 2732.554809 | 3274.70991 | 2321.832247 |
| Incidence | Oceania | Both | Age-standardized | Depressive disorders | Rate | 2019 | 2733.338757 | 3289.154215 | 2315.203416 |
| Incidence | Oceania | Both | Age-standardized | Depressive disorders | Rate | 2020 | 2763.594466 | 3465.211415 | 2175.62684 |
| Incidence | Oceania | Both | Age-standardized | Depressive disorders | Rate | 2021 | 2956.632109 | 3768.079016 | 2307.715045 |
| Incidence | Central Europe | Both | Age-standardized | Depressive disorders | Rate | 1990 | 2859.03185 | 3287.726146 | 2501.424237 |
| Incidence | Central Europe | Both | Age-standardized | Depressive disorders | Rate | 1991 | 2854.284041 | 3263.59311 | 2513.786672 |
| Incidence | Central Europe | Both | Age-standardized | Depressive disorders | Rate | 1992 | 2848.326792 | 3243.143137 | 2517.201963 |
| Incidence | Central Europe | Both | Age-standardized | Depressive disorders | Rate | 1993 | 2841.273613 | 3227.329223 | 2510.169066 |
| Incidence | Central Europe | Both | Age-standardized | Depressive disorders | Rate | 1994 | 2832.866397 | 3211.313278 | 2512.451956 |
| Incidence | Central Europe | Both | Age-standardized | Depressive disorders | Rate | 1995 | 2824.5902 | 3194.856984 | 2498.356462 |
| Incidence | Central Europe | Both | Age-standardized | Depressive disorders | Rate | 1996 | 2813.630562 | 3181.555568 | 2495.315673 |
| Incidence | Central Europe | Both | Age-standardized | Depressive disorders | Rate | 1997 | 2797.55907 | 3163.180249 | 2487.325374 |
| Incidence | Central Europe | Both | Age-standardized | Depressive disorders | Rate | 1998 | 2779.396808 | 3148.152428 | 2472.312716 |
| Incidence | Central Europe | Both | Age-standardized | Depressive disorders | Rate | 1999 | 2760.962892 | 3127.67758 | 2457.667152 |
| Incidence | Central Europe | Both | Age-standardized | Depressive disorders | Rate | 2000 | 2743.823426 | 3117.353184 | 2437.844651 |
| Incidence | Central Europe | Both | Age-standardized | Depressive disorders | Rate | 2001 | 2718.12366 | 3088.259392 | 2418.889862 |
| Incidence | Central Europe | Both | Age-standardized | Depressive disorders | Rate | 2002 | 2678.285096 | 3043.868509 | 2383.397676 |
| Incidence | Central Europe | Both | Age-standardized | Depressive disorders | Rate | 2003 | 2633.037957 | 2990.608942 | 2342.72288 |
| Incidence | Central Europe | Both | Age-standardized | Depressive disorders | Rate | 2004 | 2591.072085 | 2937.171545 | 2301.851667 |
| Incidence | Central Europe | Both | Age-standardized | Depressive disorders | Rate | 2005 | 2561.185045 | 2909.283596 | 2272.828311 |
| Incidence | Central Europe | Both | Age-standardized | Depressive disorders | Rate | 2006 | 2537.281672 | 2882.072049 | 2255.381559 |
| Incidence | Central Europe | Both | Age-standardized | Depressive disorders | Rate | 2007 | 2510.3895 | 2851.441188 | 2231.444997 |
| Incidence | Central Europe | Both | Age-standardized | Depressive disorders | Rate | 2008 | 2484.56208 | 2826.043965 | 2209.239546 |
| Incidence | Central Europe | Both | Age-standardized | Depressive disorders | Rate | 2009 | 2463.017625 | 2803.541378 | 2190.33577 |
| Incidence | Central Europe | Both | Age-standardized | Depressive disorders | Rate | 2010 | 2449.164914 | 2782.694502 | 2178.05462 |
| Incidence | Central Europe | Both | Age-standardized | Depressive disorders | Rate | 2011 | 2441.026705 | 2777.332316 | 2169.503423 |
| Incidence | Central Europe | Both | Age-standardized | Depressive disorders | Rate | 2012 | 2434.463473 | 2775.530614 | 2159.178005 |
| Incidence | Central Europe | Both | Age-standardized | Depressive disorders | Rate | 2013 | 2429.679625 | 2776.659312 | 2148.454645 |
| Incidence | Central Europe | Both | Age-standardized | Depressive disorders | Rate | 2014 | 2426.476855 | 2781.340007 | 2140.320567 |
| Incidence | Central Europe | Both | Age-standardized | Depressive disorders | Rate | 2015 | 2424.770924 | 2783.90716 | 2129.746151 |
| Incidence | Central Europe | Both | Age-standardized | Depressive disorders | Rate | 2016 | 2437.394043 | 2809.035434 | 2139.273343 |
| Incidence | Central Europe | Both | Age-standardized | Depressive disorders | Rate | 2017 | 2466.930799 | 2845.439391 | 2157.893451 |
| Incidence | Central Europe | Both | Age-standardized | Depressive disorders | Rate | 2018 | 2498.023696 | 2892.248054 | 2184.407656 |
| Incidence | Central Europe | Both | Age-standardized | Depressive disorders | Rate | 2019 | 2515.138194 | 2921.840372 | 2191.953944 |
| Incidence | Central Europe | Both | Age-standardized | Depressive disorders | Rate | 2020 | 2975.476253 | 3480.905488 | 2545.428227 |
| Incidence | Central Europe | Both | Age-standardized | Depressive disorders | Rate | 2021 | 3225.415075 | 3788.20978 | 2763.062153 |
| Incidence | High-income Asia Pacific | Both | Age-standardized | Depressive disorders | Rate | 1990 | 2275.579466 | 2566.129209 | 2028.978389 |
| Incidence | High-income Asia Pacific | Both | Age-standardized | Depressive disorders | Rate | 1991 | 2222.91795 | 2511.520524 | 1985.66741 |
| Incidence | High-income Asia Pacific | Both | Age-standardized | Depressive disorders | Rate | 1992 | 2185.31412 | 2476.776817 | 1954.336712 |
| Incidence | High-income Asia Pacific | Both | Age-standardized | Depressive disorders | Rate | 1993 | 2160.308654 | 2442.353487 | 1935.443437 |
| Incidence | High-income Asia Pacific | Both | Age-standardized | Depressive disorders | Rate | 1994 | 2145.850025 | 2421.925678 | 1928.179648 |
| Incidence | High-income Asia Pacific | Both | Age-standardized | Depressive disorders | Rate | 1995 | 2139.741055 | 2416.413893 | 1920.796799 |
| Incidence | High-income Asia Pacific | Both | Age-standardized | Depressive disorders | Rate | 1996 | 2161.340849 | 2439.884922 | 1944.547977 |
| Incidence | High-income Asia Pacific | Both | Age-standardized | Depressive disorders | Rate | 1997 | 2217.618622 | 2497.32128 | 1994.629644 |
| Incidence | High-income Asia Pacific | Both | Age-standardized | Depressive disorders | Rate | 1998 | 2288.327021 | 2573.915462 | 2053.88551 |
| Incidence | High-income Asia Pacific | Both | Age-standardized | Depressive disorders | Rate | 1999 | 2353.292598 | 2647.007495 | 2111.169599 |
| Incidence | High-income Asia Pacific | Both | Age-standardized | Depressive disorders | Rate | 2000 | 2392.830553 | 2687.624068 | 2145.583491 |
| Incidence | High-income Asia Pacific | Both | Age-standardized | Depressive disorders | Rate | 2001 | 2412.051327 | 2708.335171 | 2168.156338 |
| Incidence | High-income Asia Pacific | Both | Age-standardized | Depressive disorders | Rate | 2002 | 2428.626967 | 2721.058901 | 2188.058278 |
| Incidence | High-income Asia Pacific | Both | Age-standardized | Depressive disorders | Rate | 2003 | 2441.468638 | 2733.191355 | 2206.683735 |
| Incidence | High-income Asia Pacific | Both | Age-standardized | Depressive disorders | Rate | 2004 | 2450.06161 | 2741.746045 | 2218.968933 |
| Incidence | High-income Asia Pacific | Both | Age-standardized | Depressive disorders | Rate | 2005 | 2453.058381 | 2744.925396 | 2225.481259 |
| Incidence | High-income Asia Pacific | Both | Age-standardized | Depressive disorders | Rate | 2006 | 2451.693863 | 2739.678065 | 2226.704073 |
| Incidence | High-income Asia Pacific | Both | Age-standardized | Depressive disorders | Rate | 2007 | 2448.232191 | 2737.74417 | 2221.833689 |
| Incidence | High-income Asia Pacific | Both | Age-standardized | Depressive disorders | Rate | 2008 | 2443.338221 | 2733.093803 | 2218.170619 |
| Incidence | High-income Asia Pacific | Both | Age-standardized | Depressive disorders | Rate | 2009 | 2438.455826 | 2720.365256 | 2220.348394 |
| Incidence | High-income Asia Pacific | Both | Age-standardized | Depressive disorders | Rate | 2010 | 2433.998948 | 2712.797622 | 2214.993621 |
| Incidence | High-income Asia Pacific | Both | Age-standardized | Depressive disorders | Rate | 2011 | 2428.034532 | 2711.307854 | 2206.756381 |
| Incidence | High-income Asia Pacific | Both | Age-standardized | Depressive disorders | Rate | 2012 | 2419.438181 | 2709.408241 | 2194.855313 |
| Incidence | High-income Asia Pacific | Both | Age-standardized | Depressive disorders | Rate | 2013 | 2410.440692 | 2706.518679 | 2181.63502 |
| Incidence | High-income Asia Pacific | Both | Age-standardized | Depressive disorders | Rate | 2014 | 2403.198677 | 2703.381539 | 2169.530974 |
| Incidence | High-income Asia Pacific | Both | Age-standardized | Depressive disorders | Rate | 2015 | 2399.351311 | 2703.567698 | 2163.734886 |
| Incidence | High-income Asia Pacific | Both | Age-standardized | Depressive disorders | Rate | 2016 | 2395.269228 | 2706.497297 | 2159.216602 |
| Incidence | High-income Asia Pacific | Both | Age-standardized | Depressive disorders | Rate | 2017 | 2388.788225 | 2700.895064 | 2147.594528 |
| Incidence | High-income Asia Pacific | Both | Age-standardized | Depressive disorders | Rate | 2018 | 2383.52134 | 2703.06608 | 2130.533787 |
| Incidence | High-income Asia Pacific | Both | Age-standardized | Depressive disorders | Rate | 2019 | 2383.84358 | 2714.840997 | 2116.722683 |
| Incidence | High-income Asia Pacific | Both | Age-standardized | Depressive disorders | Rate | 2020 | 2677.028725 | 3119.019985 | 2304.946354 |
| Incidence | High-income Asia Pacific | Both | Age-standardized | Depressive disorders | Rate | 2021 | 2845.861406 | 3326.64608 | 2452.418247 |
| Incidence | Central Asia | Both | Age-standardized | Depressive disorders | Rate | 1990 | 3675.513222 | 4269.592341 | 3152.031593 |
| Incidence | Central Asia | Both | Age-standardized | Depressive disorders | Rate | 1991 | 3656.427408 | 4223.825773 | 3156.360625 |
| Incidence | Central Asia | Both | Age-standardized | Depressive disorders | Rate | 1992 | 3640.652841 | 4190.971533 | 3165.017979 |
| Incidence | Central Asia | Both | Age-standardized | Depressive disorders | Rate | 1993 | 3628.207722 | 4164.041105 | 3173.306084 |
| Incidence | Central Asia | Both | Age-standardized | Depressive disorders | Rate | 1994 | 3618.882867 | 4143.787968 | 3181.942647 |
| Incidence | Central Asia | Both | Age-standardized | Depressive disorders | Rate | 1995 | 3612.465013 | 4128.39024 | 3184.383647 |
| Incidence | Central Asia | Both | Age-standardized | Depressive disorders | Rate | 1996 | 3610.495857 | 4127.032109 | 3192.258367 |
| Incidence | Central Asia | Both | Age-standardized | Depressive disorders | Rate | 1997 | 3612.569375 | 4123.681223 | 3195.483705 |
| Incidence | Central Asia | Both | Age-standardized | Depressive disorders | Rate | 1998 | 3615.72951 | 4134.010891 | 3197.266034 |
| Incidence | Central Asia | Both | Age-standardized | Depressive disorders | Rate | 1999 | 3617.358775 | 4144.713076 | 3197.34043 |
| Incidence | Central Asia | Both | Age-standardized | Depressive disorders | Rate | 2000 | 3614.984162 | 4148.23912 | 3187.937733 |
| Incidence | Central Asia | Both | Age-standardized | Depressive disorders | Rate | 2001 | 3609.245699 | 4124.921575 | 3196.588105 |
| Incidence | Central Asia | Both | Age-standardized | Depressive disorders | Rate | 2002 | 3602.758216 | 4106.870468 | 3187.80796 |
| Incidence | Central Asia | Both | Age-standardized | Depressive disorders | Rate | 2003 | 3595.157739 | 4095.11641 | 3171.585905 |
| Incidence | Central Asia | Both | Age-standardized | Depressive disorders | Rate | 2004 | 3586.351683 | 4093.717337 | 3164.307695 |
| Incidence | Central Asia | Both | Age-standardized | Depressive disorders | Rate | 2005 | 3575.997416 | 4102.802959 | 3154.696258 |
| Incidence | Central Asia | Both | Age-standardized | Depressive disorders | Rate | 2006 | 3559.520186 | 4075.703182 | 3148.643926 |
| Incidence | Central Asia | Both | Age-standardized | Depressive disorders | Rate | 2007 | 3536.352026 | 4041.692635 | 3135.871191 |
| Incidence | Central Asia | Both | Age-standardized | Depressive disorders | Rate | 2008 | 3511.346344 | 3993.621372 | 3114.700992 |
| Incidence | Central Asia | Both | Age-standardized | Depressive disorders | Rate | 2009 | 3489.481467 | 3978.029836 | 3096.689761 |
| Incidence | Central Asia | Both | Age-standardized | Depressive disorders | Rate | 2010 | 3475.811403 | 4001.136845 | 3079.051027 |
| Incidence | Central Asia | Both | Age-standardized | Depressive disorders | Rate | 2011 | 3471.497225 | 3962.858739 | 3069.418731 |
| Incidence | Central Asia | Both | Age-standardized | Depressive disorders | Rate | 2012 | 3472.758359 | 3965.146249 | 3055.724351 |
| Incidence | Central Asia | Both | Age-standardized | Depressive disorders | Rate | 2013 | 3476.943395 | 3984.692735 | 3052.409352 |
| Incidence | Central Asia | Both | Age-standardized | Depressive disorders | Rate | 2014 | 3481.423602 | 4007.59358 | 3041.513532 |
| Incidence | Central Asia | Both | Age-standardized | Depressive disorders | Rate | 2015 | 3483.812915 | 4030.023787 | 3044.477767 |
| Incidence | Central Asia | Both | Age-standardized | Depressive disorders | Rate | 2016 | 3486.375396 | 4039.563478 | 3031.353764 |
| Incidence | Central Asia | Both | Age-standardized | Depressive disorders | Rate | 2017 | 3491.682989 | 4056.333563 | 3030.959916 |
| Incidence | Central Asia | Both | Age-standardized | Depressive disorders | Rate | 2018 | 3496.959319 | 4085.473309 | 3030.972668 |
| Incidence | Central Asia | Both | Age-standardized | Depressive disorders | Rate | 2019 | 3499.479865 | 4092.324805 | 3021.794491 |
| Incidence | Central Asia | Both | Age-standardized | Depressive disorders | Rate | 2020 | 3994.296557 | 4784.29656 | 3332.185087 |
| Incidence | Central Asia | Both | Age-standardized | Depressive disorders | Rate | 2021 | 4131.919109 | 4952.485645 | 3444.953841 |
| Incidence | Western Europe | Both | Age-standardized | Depressive disorders | Rate | 1990 | 4766.263523 | 5410.054989 | 4306.421335 |
| Incidence | Western Europe | Both | Age-standardized | Depressive disorders | Rate | 1991 | 4710.689354 | 5334.116283 | 4264.428181 |
| Incidence | Western Europe | Both | Age-standardized | Depressive disorders | Rate | 1992 | 4662.501958 | 5278.151491 | 4224.852785 |
| Incidence | Western Europe | Both | Age-standardized | Depressive disorders | Rate | 1993 | 4623.837044 | 5229.632281 | 4182.165902 |
| Incidence | Western Europe | Both | Age-standardized | Depressive disorders | Rate | 1994 | 4596.247407 | 5196.708352 | 4153.360057 |
| Incidence | Western Europe | Both | Age-standardized | Depressive disorders | Rate | 1995 | 4580.605781 | 5171.690199 | 4142.801684 |
| Incidence | Western Europe | Both | Age-standardized | Depressive disorders | Rate | 1996 | 4585.610545 | 5169.861679 | 4148.7415 |
| Incidence | Western Europe | Both | Age-standardized | Depressive disorders | Rate | 1997 | 4610.552414 | 5183.085261 | 4175.613504 |
| Incidence | Western Europe | Both | Age-standardized | Depressive disorders | Rate | 1998 | 4643.487951 | 5223.87532 | 4212.800434 |
| Incidence | Western Europe | Both | Age-standardized | Depressive disorders | Rate | 1999 | 4671.930976 | 5266.736265 | 4244.82973 |
| Incidence | Western Europe | Both | Age-standardized | Depressive disorders | Rate | 2000 | 4683.062642 | 5275.456496 | 4250.681596 |
| Incidence | Western Europe | Both | Age-standardized | Depressive disorders | Rate | 2001 | 4670.690547 | 5269.319047 | 4235.565265 |
| Incidence | Western Europe | Both | Age-standardized | Depressive disorders | Rate | 2002 | 4641.941055 | 5231.362208 | 4207.599823 |
| Incidence | Western Europe | Both | Age-standardized | Depressive disorders | Rate | 2003 | 4605.970636 | 5190.247343 | 4169.169928 |
| Incidence | Western Europe | Both | Age-standardized | Depressive disorders | Rate | 2004 | 4571.812999 | 5162.539566 | 4131.968937 |
| Incidence | Western Europe | Both | Age-standardized | Depressive disorders | Rate | 2005 | 4548.30206 | 5151.399761 | 4094.51785 |
| Incidence | Western Europe | Both | Age-standardized | Depressive disorders | Rate | 2006 | 4541.522362 | 5138.268265 | 4090.80282 |
| Incidence | Western Europe | Both | Age-standardized | Depressive disorders | Rate | 2007 | 4547.720902 | 5140.945238 | 4100.65129 |
| Incidence | Western Europe | Both | Age-standardized | Depressive disorders | Rate | 2008 | 4561.677797 | 5156.545296 | 4118.247672 |
| Incidence | Western Europe | Both | Age-standardized | Depressive disorders | Rate | 2009 | 4577.711154 | 5169.520976 | 4131.087622 |
| Incidence | Western Europe | Both | Age-standardized | Depressive disorders | Rate | 2010 | 4589.816301 | 5182.1298 | 4136.458422 |
| Incidence | Western Europe | Both | Age-standardized | Depressive disorders | Rate | 2011 | 4591.956664 | 5194.375218 | 4141.246752 |
| Incidence | Western Europe | Both | Age-standardized | Depressive disorders | Rate | 2012 | 4585.348548 | 5201.81321 | 4124.516433 |
| Incidence | Western Europe | Both | Age-standardized | Depressive disorders | Rate | 2013 | 4575.581605 | 5203.873343 | 4106.906371 |
| Incidence | Western Europe | Both | Age-standardized | Depressive disorders | Rate | 2014 | 4568.254944 | 5211.729234 | 4087.367694 |
| Incidence | Western Europe | Both | Age-standardized | Depressive disorders | Rate | 2015 | 4568.798645 | 5229.625086 | 4075.835429 |
| Incidence | Western Europe | Both | Age-standardized | Depressive disorders | Rate | 2016 | 4578.954616 | 5256.166095 | 4064.17375 |
| Incidence | Western Europe | Both | Age-standardized | Depressive disorders | Rate | 2017 | 4591.688936 | 5293.661296 | 4052.700569 |
| Incidence | Western Europe | Both | Age-standardized | Depressive disorders | Rate | 2018 | 4599.973442 | 5337.471102 | 4035.001042 |
| Incidence | Western Europe | Both | Age-standardized | Depressive disorders | Rate | 2019 | 4597.024308 | 5356.545209 | 4015.253939 |
| Incidence | Western Europe | Both | Age-standardized | Depressive disorders | Rate | 2020 | 5627.923828 | 6701.700463 | 4849.384479 |
| Incidence | Western Europe | Both | Age-standardized | Depressive disorders | Rate | 2021 | 5634.45546 | 6686.608852 | 4852.738879 |
| Incidence | Eastern Europe | Both | Age-standardized | Depressive disorders | Rate | 1990 | 4122.864912 | 4767.117564 | 3579.307529 |
| Incidence | Eastern Europe | Both | Age-standardized | Depressive disorders | Rate | 1991 | 4159.540859 | 4816.788865 | 3612.203721 |
| Incidence | Eastern Europe | Both | Age-standardized | Depressive disorders | Rate | 1992 | 4189.532644 | 4859.363932 | 3638.143385 |
| Incidence | Eastern Europe | Both | Age-standardized | Depressive disorders | Rate | 1993 | 4213.348864 | 4885.959161 | 3657.606121 |
| Incidence | Eastern Europe | Both | Age-standardized | Depressive disorders | Rate | 1994 | 4232.284364 | 4906.07591 | 3672.863199 |
| Incidence | Eastern Europe | Both | Age-standardized | Depressive disorders | Rate | 1995 | 4243.866888 | 4925.890555 | 3677.937369 |
| Incidence | Eastern Europe | Both | Age-standardized | Depressive disorders | Rate | 1996 | 4247.678256 | 4927.946355 | 3689.095986 |
| Incidence | Eastern Europe | Both | Age-standardized | Depressive disorders | Rate | 1997 | 4243.582845 | 4917.228867 | 3694.157362 |
| Incidence | Eastern Europe | Both | Age-standardized | Depressive disorders | Rate | 1998 | 4235.089156 | 4893.612521 | 3687.559898 |
| Incidence | Eastern Europe | Both | Age-standardized | Depressive disorders | Rate | 1999 | 4225.053075 | 4879.460165 | 3678.688522 |
| Incidence | Eastern Europe | Both | Age-standardized | Depressive disorders | Rate | 2000 | 4214.00266 | 4868.79631 | 3674.101239 |
| Incidence | Eastern Europe | Both | Age-standardized | Depressive disorders | Rate | 2001 | 4192.54639 | 4845.369859 | 3647.610985 |
| Incidence | Eastern Europe | Both | Age-standardized | Depressive disorders | Rate | 2002 | 4153.333561 | 4794.292508 | 3610.23739 |
| Incidence | Eastern Europe | Both | Age-standardized | Depressive disorders | Rate | 2003 | 4105.398481 | 4737.383679 | 3566.362715 |
| Incidence | Eastern Europe | Both | Age-standardized | Depressive disorders | Rate | 2004 | 4055.870119 | 4682.680916 | 3521.312413 |
| Incidence | Eastern Europe | Both | Age-standardized | Depressive disorders | Rate | 2005 | 4010.744302 | 4634.857509 | 3480.886796 |
| Incidence | Eastern Europe | Both | Age-standardized | Depressive disorders | Rate | 2006 | 3961.090914 | 4573.239951 | 3435.817731 |
| Incidence | Eastern Europe | Both | Age-standardized | Depressive disorders | Rate | 2007 | 3899.157037 | 4495.733482 | 3380.904361 |
| Incidence | Eastern Europe | Both | Age-standardized | Depressive disorders | Rate | 2008 | 3837.259609 | 4425.10429 | 3327.739872 |
| Incidence | Eastern Europe | Both | Age-standardized | Depressive disorders | Rate | 2009 | 3785.750095 | 4365.316852 | 3281.367206 |
| Incidence | Eastern Europe | Both | Age-standardized | Depressive disorders | Rate | 2010 | 3753.949899 | 4329.636917 | 3252.671058 |
| Incidence | Eastern Europe | Both | Age-standardized | Depressive disorders | Rate | 2011 | 3743.837253 | 4320.229305 | 3246.617709 |
| Incidence | Eastern Europe | Both | Age-standardized | Depressive disorders | Rate | 2012 | 3746.052875 | 4328.332738 | 3250.976638 |
| Incidence | Eastern Europe | Both | Age-standardized | Depressive disorders | Rate | 2013 | 3755.308141 | 4346.559527 | 3262.911965 |
| Incidence | Eastern Europe | Both | Age-standardized | Depressive disorders | Rate | 2014 | 3765.091489 | 4363.894174 | 3274.449187 |
| Incidence | Eastern Europe | Both | Age-standardized | Depressive disorders | Rate | 2015 | 3767.727599 | 4373.362026 | 3276.699165 |
| Incidence | Eastern Europe | Both | Age-standardized | Depressive disorders | Rate | 2016 | 3761.907009 | 4369.351515 | 3269.867986 |
| Incidence | Eastern Europe | Both | Age-standardized | Depressive disorders | Rate | 2017 | 3751.958118 | 4360.435308 | 3259.291518 |
| Incidence | Eastern Europe | Both | Age-standardized | Depressive disorders | Rate | 2018 | 3740.917547 | 4351.128597 | 3247.028241 |
| Incidence | Eastern Europe | Both | Age-standardized | Depressive disorders | Rate | 2019 | 3730.949984 | 4344.186575 | 3236.070857 |
| Incidence | Eastern Europe | Both | Age-standardized | Depressive disorders | Rate | 2020 | 4294.164028 | 5021.544548 | 3671.771134 |
| Incidence | Eastern Europe | Both | Age-standardized | Depressive disorders | Rate | 2021 | 4833.938495 | 5669.692664 | 4115.802244 |
| Incidence | Southern Latin America | Both | Age-standardized | Depressive disorders | Rate | 1990 | 3752.707002 | 4407.846025 | 3288.814942 |
| Incidence | Southern Latin America | Both | Age-standardized | Depressive disorders | Rate | 1991 | 3749.031995 | 4364.581993 | 3302.445924 |
| Incidence | Southern Latin America | Both | Age-standardized | Depressive disorders | Rate | 1992 | 3745.104193 | 4330.338761 | 3309.278978 |
| Incidence | Southern Latin America | Both | Age-standardized | Depressive disorders | Rate | 1993 | 3741.236786 | 4302.477564 | 3318.458735 |
| Incidence | Southern Latin America | Both | Age-standardized | Depressive disorders | Rate | 1994 | 3737.549522 | 4278.454758 | 3323.398876 |
| Incidence | Southern Latin America | Both | Age-standardized | Depressive disorders | Rate | 1995 | 3734.563556 | 4284.406619 | 3329.05772 |
| Incidence | Southern Latin America | Both | Age-standardized | Depressive disorders | Rate | 1996 | 3730.137424 | 4302.842801 | 3333.52547 |
| Incidence | Southern Latin America | Both | Age-standardized | Depressive disorders | Rate | 1997 | 3722.948328 | 4287.325271 | 3332.227673 |
| Incidence | Southern Latin America | Both | Age-standardized | Depressive disorders | Rate | 1998 | 3715.483114 | 4286.402706 | 3330.328962 |
| Incidence | Southern Latin America | Both | Age-standardized | Depressive disorders | Rate | 1999 | 3710.380409 | 4269.345503 | 3328.35575 |
| Incidence | Southern Latin America | Both | Age-standardized | Depressive disorders | Rate | 2000 | 3710.206809 | 4263.397533 | 3325.72717 |
| Incidence | Southern Latin America | Both | Age-standardized | Depressive disorders | Rate | 2001 | 3715.610414 | 4267.941557 | 3319.182628 |
| Incidence | Southern Latin America | Both | Age-standardized | Depressive disorders | Rate | 2002 | 3723.423419 | 4269.80468 | 3325.458637 |
| Incidence | Southern Latin America | Both | Age-standardized | Depressive disorders | Rate | 2003 | 3730.506995 | 4266.503843 | 3318.591781 |
| Incidence | Southern Latin America | Both | Age-standardized | Depressive disorders | Rate | 2004 | 3733.4153 | 4270.426557 | 3310.304073 |
| Incidence | Southern Latin America | Both | Age-standardized | Depressive disorders | Rate | 2005 | 3728.826681 | 4258.175577 | 3291.144636 |
| Incidence | Southern Latin America | Both | Age-standardized | Depressive disorders | Rate | 2006 | 3692.794034 | 4215.218227 | 3261.68846 |
| Incidence | Southern Latin America | Both | Age-standardized | Depressive disorders | Rate | 2007 | 3620.075703 | 4127.709108 | 3216.470009 |
| Incidence | Southern Latin America | Both | Age-standardized | Depressive disorders | Rate | 2008 | 3535.716451 | 4017.991334 | 3152.389811 |
| Incidence | Southern Latin America | Both | Age-standardized | Depressive disorders | Rate | 2009 | 3464.748916 | 3935.861689 | 3092.082748 |
| Incidence | Southern Latin America | Both | Age-standardized | Depressive disorders | Rate | 2010 | 3432.311079 | 3899.871531 | 3052.379052 |
| Incidence | Southern Latin America | Both | Age-standardized | Depressive disorders | Rate | 2011 | 3432.429595 | 3885.323102 | 3061.061101 |
| Incidence | Southern Latin America | Both | Age-standardized | Depressive disorders | Rate | 2012 | 3439.30606 | 3906.320331 | 3061.746449 |
| Incidence | Southern Latin America | Both | Age-standardized | Depressive disorders | Rate | 2013 | 3448.512011 | 3922.332776 | 3077.654627 |
| Incidence | Southern Latin America | Both | Age-standardized | Depressive disorders | Rate | 2014 | 3455.934396 | 3935.830946 | 3086.26687 |
| Incidence | Southern Latin America | Both | Age-standardized | Depressive disorders | Rate | 2015 | 3457.410784 | 3948.105636 | 3087.493239 |
| Incidence | Southern Latin America | Both | Age-standardized | Depressive disorders | Rate | 2016 | 3450.446676 | 3959.058771 | 3078.732836 |
| Incidence | Southern Latin America | Both | Age-standardized | Depressive disorders | Rate | 2017 | 3438.285108 | 3969.884639 | 3058.507023 |
| Incidence | Southern Latin America | Both | Age-standardized | Depressive disorders | Rate | 2018 | 3426.027728 | 3970.201854 | 3031.187161 |
| Incidence | Southern Latin America | Both | Age-standardized | Depressive disorders | Rate | 2019 | 3418.269308 | 3951.187346 | 3004.199359 |
| Incidence | Southern Latin America | Both | Age-standardized | Depressive disorders | Rate | 2020 | 4399.453948 | 5429.500286 | 3592.448686 |
| Incidence | Southern Latin America | Both | Age-standardized | Depressive disorders | Rate | 2021 | 4330.317772 | 5341.733585 | 3543.253346 |
| Incidence | Australasia | Both | Age-standardized | Depressive disorders | Rate | 1990 | 5138.477864 | 5941.371894 | 4487.899755 |
| Incidence | Australasia | Both | Age-standardized | Depressive disorders | Rate | 1991 | 5175.268863 | 5936.93405 | 4531.780595 |
| Incidence | Australasia | Both | Age-standardized | Depressive disorders | Rate | 1992 | 5208.029565 | 5983.034954 | 4599.964032 |
| Incidence | Australasia | Both | Age-standardized | Depressive disorders | Rate | 1993 | 5236.978526 | 6026.785226 | 4651.616817 |
| Incidence | Australasia | Both | Age-standardized | Depressive disorders | Rate | 1994 | 5262.201657 | 6018.95304 | 4675.655483 |
| Incidence | Australasia | Both | Age-standardized | Depressive disorders | Rate | 1995 | 5284.092072 | 6036.190754 | 4686.66416 |
| Incidence | Australasia | Both | Age-standardized | Depressive disorders | Rate | 1996 | 5294.252142 | 6003.367003 | 4738.67448 |
| Incidence | Australasia | Both | Age-standardized | Depressive disorders | Rate | 1997 | 5291.854963 | 5968.561916 | 4779.160812 |
| Incidence | Australasia | Both | Age-standardized | Depressive disorders | Rate | 1998 | 5287.871707 | 5929.942706 | 4806.873535 |
| Incidence | Australasia | Both | Age-standardized | Depressive disorders | Rate | 1999 | 5293.492755 | 5900.651476 | 4825.351154 |
| Incidence | Australasia | Both | Age-standardized | Depressive disorders | Rate | 2000 | 5318.561316 | 5915.155012 | 4845.046544 |
| Incidence | Australasia | Both | Age-standardized | Depressive disorders | Rate | 2001 | 5383.349208 | 5997.401841 | 4915.346194 |
| Incidence | Australasia | Both | Age-standardized | Depressive disorders | Rate | 2002 | 5481.293748 | 6121.73884 | 4998.995816 |
| Incidence | Australasia | Both | Age-standardized | Depressive disorders | Rate | 2003 | 5584.480889 | 6276.696516 | 5077.721866 |
| Incidence | Australasia | Both | Age-standardized | Depressive disorders | Rate | 2004 | 5668.397399 | 6383.05195 | 5116.958837 |
| Incidence | Australasia | Both | Age-standardized | Depressive disorders | Rate | 2005 | 5706.302438 | 6451.440309 | 5122.258224 |
| Incidence | Australasia | Both | Age-standardized | Depressive disorders | Rate | 2006 | 5696.506841 | 6423.801723 | 5120.896892 |
| Incidence | Australasia | Both | Age-standardized | Depressive disorders | Rate | 2007 | 5663.25292 | 6352.236831 | 5093.416807 |
| Incidence | Australasia | Both | Age-standardized | Depressive disorders | Rate | 2008 | 5617.337721 | 6273.567029 | 5039.296427 |
| Incidence | Australasia | Both | Age-standardized | Depressive disorders | Rate | 2009 | 5569.802013 | 6249.211366 | 4981.100954 |
| Incidence | Australasia | Both | Age-standardized | Depressive disorders | Rate | 2010 | 5531.326399 | 6208.714522 | 4920.829782 |
| Incidence | Australasia | Both | Age-standardized | Depressive disorders | Rate | 2011 | 5501.348069 | 6191.698918 | 4884.640405 |
| Incidence | Australasia | Both | Age-standardized | Depressive disorders | Rate | 2012 | 5471.207727 | 6180.695994 | 4838.254236 |
| Incidence | Australasia | Both | Age-standardized | Depressive disorders | Rate | 2013 | 5439.588486 | 6180.039332 | 4778.948481 |
| Incidence | Australasia | Both | Age-standardized | Depressive disorders | Rate | 2014 | 5405.669877 | 6203.295085 | 4738.631597 |
| Incidence | Australasia | Both | Age-standardized | Depressive disorders | Rate | 2015 | 5369.459595 | 6222.179069 | 4678.574394 |
| Incidence | Australasia | Both | Age-standardized | Depressive disorders | Rate | 2016 | 5315.677655 | 6171.235345 | 4640.368888 |
| Incidence | Australasia | Both | Age-standardized | Depressive disorders | Rate | 2017 | 5244.776411 | 6159.331021 | 4578.257866 |
| Incidence | Australasia | Both | Age-standardized | Depressive disorders | Rate | 2018 | 5180.301742 | 6098.649117 | 4502.294396 |
| Incidence | Australasia | Both | Age-standardized | Depressive disorders | Rate | 2019 | 5145.253805 | 6095.563068 | 4468.000365 |
| Incidence | Australasia | Both | Age-standardized | Depressive disorders | Rate | 2020 | 5618.18178 | 6999.899219 | 4402.276188 |
| Incidence | Australasia | Both | Age-standardized | Depressive disorders | Rate | 2021 | 5579.699502 | 7067.861879 | 4387.273384 |
| Incidence | Central Latin America | Both | Age-standardized | Depressive disorders | Rate | 1990 | 3395.737323 | 3982.921064 | 2968.17085 |
| Incidence | Central Latin America | Both | Age-standardized | Depressive disorders | Rate | 1991 | 3415.072054 | 3990.568921 | 2984.528648 |
| Incidence | Central Latin America | Both | Age-standardized | Depressive disorders | Rate | 1992 | 3434.747594 | 4002.198133 | 3002.484388 |
| Incidence | Central Latin America | Both | Age-standardized | Depressive disorders | Rate | 1993 | 3453.631334 | 4014.295107 | 3026.608086 |
| Incidence | Central Latin America | Both | Age-standardized | Depressive disorders | Rate | 1994 | 3470.665261 | 4024.208214 | 3048.603183 |
| Incidence | Central Latin America | Both | Age-standardized | Depressive disorders | Rate | 1995 | 3484.686188 | 4030.747787 | 3057.917604 |
| Incidence | Central Latin America | Both | Age-standardized | Depressive disorders | Rate | 1996 | 3502.405484 | 4047.328402 | 3070.074245 |
| Incidence | Central Latin America | Both | Age-standardized | Depressive disorders | Rate | 1997 | 3527.394632 | 4073.280117 | 3096.295262 |
| Incidence | Central Latin America | Both | Age-standardized | Depressive disorders | Rate | 1998 | 3554.475502 | 4101.301632 | 3126.682944 |
| Incidence | Central Latin America | Both | Age-standardized | Depressive disorders | Rate | 1999 | 3578.489612 | 4123.926552 | 3155.00034 |
| Incidence | Central Latin America | Both | Age-standardized | Depressive disorders | Rate | 2000 | 3594.065417 | 4141.413291 | 3171.189668 |
| Incidence | Central Latin America | Both | Age-standardized | Depressive disorders | Rate | 2001 | 3603.242596 | 4142.795686 | 3184.344094 |
| Incidence | Central Latin America | Both | Age-standardized | Depressive disorders | Rate | 2002 | 3611.512264 | 4141.483161 | 3189.192158 |
| Incidence | Central Latin America | Both | Age-standardized | Depressive disorders | Rate | 2003 | 3619.118021 | 4142.554704 | 3196.601565 |
| Incidence | Central Latin America | Both | Age-standardized | Depressive disorders | Rate | 2004 | 3626.317014 | 4151.788239 | 3203.721152 |
| Incidence | Central Latin America | Both | Age-standardized | Depressive disorders | Rate | 2005 | 3633.262101 | 4158.797035 | 3213.044977 |
| Incidence | Central Latin America | Both | Age-standardized | Depressive disorders | Rate | 2006 | 3646.644268 | 4169.461186 | 3228.998016 |
| Incidence | Central Latin America | Both | Age-standardized | Depressive disorders | Rate | 2007 | 3668.783061 | 4195.239805 | 3247.485895 |
| Incidence | Central Latin America | Both | Age-standardized | Depressive disorders | Rate | 2008 | 3693.687653 | 4233.765707 | 3273.574375 |
| Incidence | Central Latin America | Both | Age-standardized | Depressive disorders | Rate | 2009 | 3715.303872 | 4270.181191 | 3295.607848 |
| Incidence | Central Latin America | Both | Age-standardized | Depressive disorders | Rate | 2010 | 3727.31836 | 4282.172275 | 3307.045595 |
| Incidence | Central Latin America | Both | Age-standardized | Depressive disorders | Rate | 2011 | 3729.331272 | 4287.059125 | 3304.758667 |
| Incidence | Central Latin America | Both | Age-standardized | Depressive disorders | Rate | 2012 | 3726.689838 | 4290.486508 | 3297.597322 |
| Incidence | Central Latin America | Both | Age-standardized | Depressive disorders | Rate | 2013 | 3722.013415 | 4288.755191 | 3285.632409 |
| Incidence | Central Latin America | Both | Age-standardized | Depressive disorders | Rate | 2014 | 3717.950873 | 4295.504561 | 3273.944398 |
| Incidence | Central Latin America | Both | Age-standardized | Depressive disorders | Rate | 2015 | 3717.310031 | 4301.748001 | 3263.998462 |
| Incidence | Central Latin America | Both | Age-standardized | Depressive disorders | Rate | 2016 | 3721.776769 | 4319.940174 | 3262.573381 |
| Incidence | Central Latin America | Both | Age-standardized | Depressive disorders | Rate | 2017 | 3728.860126 | 4337.073894 | 3264.849245 |
| Incidence | Central Latin America | Both | Age-standardized | Depressive disorders | Rate | 2018 | 3734.657077 | 4346.096263 | 3268.329044 |
| Incidence | Central Latin America | Both | Age-standardized | Depressive disorders | Rate | 2019 | 3735.699259 | 4355.65932 | 3266.253251 |
| Incidence | Central Latin America | Both | Age-standardized | Depressive disorders | Rate | 2020 | 4690.686317 | 5547.269305 | 4048.452797 |
| Incidence | Central Latin America | Both | Age-standardized | Depressive disorders | Rate | 2021 | 4574.680341 | 5407.859223 | 3953.812701 |
| Incidence | High-income North America | Both | Age-standardized | Depressive disorders | Rate | 1990 | 3907.663053 | 4476.331089 | 3466.451373 |
| Incidence | High-income North America | Both | Age-standardized | Depressive disorders | Rate | 1991 | 4062.9362 | 4652.142794 | 3600.057447 |
| Incidence | High-income North America | Both | Age-standardized | Depressive disorders | Rate | 1992 | 4220.718093 | 4839.620137 | 3741.747914 |
| Incidence | High-income North America | Both | Age-standardized | Depressive disorders | Rate | 1993 | 4375.086544 | 5025.214652 | 3885.82 |
| Incidence | High-income North America | Both | Age-standardized | Depressive disorders | Rate | 1994 | 4520.05709 | 5181.970033 | 4016.495082 |
| Incidence | High-income North America | Both | Age-standardized | Depressive disorders | Rate | 1995 | 4649.477259 | 5323.074312 | 4130.985408 |
| Incidence | High-income North America | Both | Age-standardized | Depressive disorders | Rate | 1996 | 4803.602548 | 5498.161844 | 4271.622281 |
| Incidence | High-income North America | Both | Age-standardized | Depressive disorders | Rate | 1997 | 4998.630256 | 5719.602841 | 4449.019688 |
| Incidence | High-income North America | Both | Age-standardized | Depressive disorders | Rate | 1998 | 5192.842766 | 5924.894689 | 4624.275725 |
| Incidence | High-income North America | Both | Age-standardized | Depressive disorders | Rate | 1999 | 5344.668091 | 6093.98748 | 4763.90603 |
| Incidence | High-income North America | Both | Age-standardized | Depressive disorders | Rate | 2000 | 5412.415352 | 6167.271511 | 4817.564345 |
| Incidence | High-income North America | Both | Age-standardized | Depressive disorders | Rate | 2001 | 5406.779273 | 6159.928533 | 4817.509655 |
| Incidence | High-income North America | Both | Age-standardized | Depressive disorders | Rate | 2002 | 5376.099092 | 6121.080259 | 4793.676001 |
| Incidence | High-income North America | Both | Age-standardized | Depressive disorders | Rate | 2003 | 5335.610417 | 6076.205154 | 4768.657021 |
| Incidence | High-income North America | Both | Age-standardized | Depressive disorders | Rate | 2004 | 5300.546914 | 6031.02329 | 4747.969247 |
| Incidence | High-income North America | Both | Age-standardized | Depressive disorders | Rate | 2005 | 5286.102987 | 6010.766988 | 4741.640128 |
| Incidence | High-income North America | Both | Age-standardized | Depressive disorders | Rate | 2006 | 5297.17301 | 6005.277818 | 4749.115539 |
| Incidence | High-income North America | Both | Age-standardized | Depressive disorders | Rate | 2007 | 5321.950364 | 6026.623234 | 4766.451904 |
| Incidence | High-income North America | Both | Age-standardized | Depressive disorders | Rate | 2008 | 5351.077703 | 6039.191265 | 4783.404246 |
| Incidence | High-income North America | Both | Age-standardized | Depressive disorders | Rate | 2009 | 5374.995478 | 6061.430065 | 4798.226049 |
| Incidence | High-income North America | Both | Age-standardized | Depressive disorders | Rate | 2010 | 5383.903984 | 6070.933768 | 4795.434986 |
| Incidence | High-income North America | Both | Age-standardized | Depressive disorders | Rate | 2011 | 5347.739929 | 6020.041997 | 4769.637656 |
| Incidence | High-income North America | Both | Age-standardized | Depressive disorders | Rate | 2012 | 5263.176633 | 5919.393299 | 4695.373559 |
| Incidence | High-income North America | Both | Age-standardized | Depressive disorders | Rate | 2013 | 5160.910167 | 5798.945817 | 4618.036496 |
| Incidence | High-income North America | Both | Age-standardized | Depressive disorders | Rate | 2014 | 5071.264004 | 5709.167009 | 4542.227665 |
| Incidence | High-income North America | Both | Age-standardized | Depressive disorders | Rate | 2015 | 5024.891338 | 5667.290371 | 4506.501068 |
| Incidence | High-income North America | Both | Age-standardized | Depressive disorders | Rate | 2016 | 5014.086769 | 5656.247993 | 4498.309422 |
| Incidence | High-income North America | Both | Age-standardized | Depressive disorders | Rate | 2017 | 5011.006281 | 5663.052618 | 4495.019089 |
| Incidence | High-income North America | Both | Age-standardized | Depressive disorders | Rate | 2018 | 5016.225655 | 5688.751312 | 4482.262903 |
| Incidence | High-income North America | Both | Age-standardized | Depressive disorders | Rate | 2019 | 5030.217333 | 5723.008259 | 4493.691048 |
| Incidence | High-income North America | Both | Age-standardized | Depressive disorders | Rate | 2020 | 6559.506211 | 7590.959738 | 5769.37038 |
| Incidence | High-income North America | Both | Age-standardized | Depressive disorders | Rate | 2021 | 6572.237183 | 7626.112583 | 5787.265286 |
| Incidence | North Africa and Middle East | Both | Age-standardized | Depressive disorders | Rate | 1990 | 5138.945901 | 6131.266723 | 4371.200365 |
| Incidence | North Africa and Middle East | Both | Age-standardized | Depressive disorders | Rate | 1991 | 5129.954231 | 6127.317789 | 4374.398194 |
| Incidence | North Africa and Middle East | Both | Age-standardized | Depressive disorders | Rate | 1992 | 5120.471366 | 6115.753677 | 4381.85118 |
| Incidence | North Africa and Middle East | Both | Age-standardized | Depressive disorders | Rate | 1993 | 5111.316793 | 6107.230464 | 4379.556402 |
| Incidence | North Africa and Middle East | Both | Age-standardized | Depressive disorders | Rate | 1994 | 5103.495761 | 6087.155594 | 4379.217676 |
| Incidence | North Africa and Middle East | Both | Age-standardized | Depressive disorders | Rate | 1995 | 5098.03981 | 6059.54338 | 4382.7964 |
| Incidence | North Africa and Middle East | Both | Age-standardized | Depressive disorders | Rate | 1996 | 5087.275799 | 6025.769312 | 4398.720472 |
| Incidence | North Africa and Middle East | Both | Age-standardized | Depressive disorders | Rate | 1997 | 5067.349154 | 5966.877128 | 4409.228734 |
| Incidence | North Africa and Middle East | Both | Age-standardized | Depressive disorders | Rate | 1998 | 5045.490767 | 5910.217416 | 4415.010937 |
| Incidence | North Africa and Middle East | Both | Age-standardized | Depressive disorders | Rate | 1999 | 5028.62733 | 5866.203405 | 4423.725647 |
| Incidence | North Africa and Middle East | Both | Age-standardized | Depressive disorders | Rate | 2000 | 5023.450999 | 5852.130206 | 4432.609722 |
| Incidence | North Africa and Middle East | Both | Age-standardized | Depressive disorders | Rate | 2001 | 5033.062372 | 5872.521308 | 4440.639998 |
| Incidence | North Africa and Middle East | Both | Age-standardized | Depressive disorders | Rate | 2002 | 5052.249132 | 5908.479924 | 4455.807859 |
| Incidence | North Africa and Middle East | Both | Age-standardized | Depressive disorders | Rate | 2003 | 5074.781495 | 5949.103618 | 4463.077465 |
| Incidence | North Africa and Middle East | Both | Age-standardized | Depressive disorders | Rate | 2004 | 5093.493418 | 5987.560531 | 4466.268767 |
| Incidence | North Africa and Middle East | Both | Age-standardized | Depressive disorders | Rate | 2005 | 5102.329804 | 6002.735363 | 4458.852299 |
| Incidence | North Africa and Middle East | Both | Age-standardized | Depressive disorders | Rate | 2006 | 5104.160353 | 5997.757527 | 4470.414963 |
| Incidence | North Africa and Middle East | Both | Age-standardized | Depressive disorders | Rate | 2007 | 5105.307819 | 5989.312054 | 4473.293242 |
| Incidence | North Africa and Middle East | Both | Age-standardized | Depressive disorders | Rate | 2008 | 5104.660625 | 5984.833177 | 4463.257531 |
| Incidence | North Africa and Middle East | Both | Age-standardized | Depressive disorders | Rate | 2009 | 5103.896655 | 5979.966527 | 4457.203317 |
| Incidence | North Africa and Middle East | Both | Age-standardized | Depressive disorders | Rate | 2010 | 5105.617392 | 5979.867141 | 4459.92381 |
| Incidence | North Africa and Middle East | Both | Age-standardized | Depressive disorders | Rate | 2011 | 5120.664908 | 6003.653443 | 4458.955068 |
| Incidence | North Africa and Middle East | Both | Age-standardized | Depressive disorders | Rate | 2012 | 5153.624343 | 6057.793746 | 4466.211702 |
| Incidence | North Africa and Middle East | Both | Age-standardized | Depressive disorders | Rate | 2013 | 5193.26814 | 6130.193746 | 4480.008819 |
| Incidence | North Africa and Middle East | Both | Age-standardized | Depressive disorders | Rate | 2014 | 5227.477175 | 6195.498225 | 4483.353774 |
| Incidence | North Africa and Middle East | Both | Age-standardized | Depressive disorders | Rate | 2015 | 5242.663038 | 6228.822471 | 4467.837967 |
| Incidence | North Africa and Middle East | Both | Age-standardized | Depressive disorders | Rate | 2016 | 5237.980683 | 6241.123962 | 4447.77591 |
| Incidence | North Africa and Middle East | Both | Age-standardized | Depressive disorders | Rate | 2017 | 5224.30494 | 6237.769732 | 4421.267128 |
| Incidence | North Africa and Middle East | Both | Age-standardized | Depressive disorders | Rate | 2018 | 5205.776452 | 6234.13857 | 4394.372371 |
| Incidence | North Africa and Middle East | Both | Age-standardized | Depressive disorders | Rate | 2019 | 5186.285875 | 6233.466823 | 4368.129854 |
| Incidence | North Africa and Middle East | Both | Age-standardized | Depressive disorders | Rate | 2020 | 6097.872652 | 7364.611461 | 5049.368927 |
| Incidence | North Africa and Middle East | Both | Age-standardized | Depressive disorders | Rate | 2021 | 5983.104933 | 7214.680169 | 4953.301373 |
| Incidence | Andean Latin America | Both | Age-standardized | Depressive disorders | Rate | 1990 | 2985.027891 | 3527.329549 | 2554.931509 |
| Incidence | Andean Latin America | Both | Age-standardized | Depressive disorders | Rate | 1991 | 2982.477614 | 3516.366813 | 2555.633473 |
| Incidence | Andean Latin America | Both | Age-standardized | Depressive disorders | Rate | 1992 | 2980.010725 | 3516.102747 | 2559.388367 |
| Incidence | Andean Latin America | Both | Age-standardized | Depressive disorders | Rate | 1993 | 2977.745107 | 3497.878508 | 2560.027937 |
| Incidence | Andean Latin America | Both | Age-standardized | Depressive disorders | Rate | 1994 | 2975.652264 | 3485.439672 | 2555.348563 |
| Incidence | Andean Latin America | Both | Age-standardized | Depressive disorders | Rate | 1995 | 2973.562234 | 3480.616821 | 2555.781998 |
| Incidence | Andean Latin America | Both | Age-standardized | Depressive disorders | Rate | 1996 | 2973.337178 | 3472.111152 | 2569.045141 |
| Incidence | Andean Latin America | Both | Age-standardized | Depressive disorders | Rate | 1997 | 2975.456613 | 3467.065001 | 2590.491431 |
| Incidence | Andean Latin America | Both | Age-standardized | Depressive disorders | Rate | 1998 | 2978.248054 | 3451.815275 | 2611.659698 |
| Incidence | Andean Latin America | Both | Age-standardized | Depressive disorders | Rate | 1999 | 2979.716417 | 3424.641748 | 2609.409979 |
| Incidence | Andean Latin America | Both | Age-standardized | Depressive disorders | Rate | 2000 | 2977.829751 | 3425.300643 | 2611.265797 |
| Incidence | Andean Latin America | Both | Age-standardized | Depressive disorders | Rate | 2001 | 2972.489338 | 3405.545764 | 2608.608658 |
| Incidence | Andean Latin America | Both | Age-standardized | Depressive disorders | Rate | 2002 | 2964.679924 | 3391.610371 | 2600.74579 |
| Incidence | Andean Latin America | Both | Age-standardized | Depressive disorders | Rate | 2003 | 2954.799815 | 3382.942483 | 2594.420378 |
| Incidence | Andean Latin America | Both | Age-standardized | Depressive disorders | Rate | 2004 | 2943.466519 | 3377.051268 | 2579.086486 |
| Incidence | Andean Latin America | Both | Age-standardized | Depressive disorders | Rate | 2005 | 2930.924107 | 3372.693811 | 2567.49265 |
| Incidence | Andean Latin America | Both | Age-standardized | Depressive disorders | Rate | 2006 | 2909.833019 | 3345.774498 | 2550.261454 |
| Incidence | Andean Latin America | Both | Age-standardized | Depressive disorders | Rate | 2007 | 2877.915049 | 3301.899353 | 2532.762191 |
| Incidence | Andean Latin America | Both | Age-standardized | Depressive disorders | Rate | 2008 | 2843.687233 | 3266.801729 | 2510.000193 |
| Incidence | Andean Latin America | Both | Age-standardized | Depressive disorders | Rate | 2009 | 2815.739229 | 3231.56899 | 2486.74694 |
| Incidence | Andean Latin America | Both | Age-standardized | Depressive disorders | Rate | 2010 | 2802.313814 | 3221.934144 | 2474.417213 |
| Incidence | Andean Latin America | Both | Age-standardized | Depressive disorders | Rate | 2011 | 2798.968705 | 3221.87528 | 2462.817726 |
| Incidence | Andean Latin America | Both | Age-standardized | Depressive disorders | Rate | 2012 | 2795.533224 | 3234.483041 | 2459.301601 |
| Incidence | Andean Latin America | Both | Age-standardized | Depressive disorders | Rate | 2013 | 2791.838764 | 3245.047536 | 2442.149929 |
| Incidence | Andean Latin America | Both | Age-standardized | Depressive disorders | Rate | 2014 | 2789.583952 | 3255.062342 | 2410.406551 |
| Incidence | Andean Latin America | Both | Age-standardized | Depressive disorders | Rate | 2015 | 2789.5237 | 3248.698597 | 2393.888518 |
| Incidence | Andean Latin America | Both | Age-standardized | Depressive disorders | Rate | 2016 | 2791.34767 | 3291.747732 | 2401.303864 |
| Incidence | Andean Latin America | Both | Age-standardized | Depressive disorders | Rate | 2017 | 2792.301523 | 3329.300917 | 2398.756994 |
| Incidence | Andean Latin America | Both | Age-standardized | Depressive disorders | Rate | 2018 | 2791.718089 | 3361.879608 | 2401.204452 |
| Incidence | Andean Latin America | Both | Age-standardized | Depressive disorders | Rate | 2019 | 2789.179123 | 3363.625248 | 2396.482883 |
| Incidence | Andean Latin America | Both | Age-standardized | Depressive disorders | Rate | 2020 | 3971.00597 | 4953.067599 | 3272.527168 |
| Incidence | Andean Latin America | Both | Age-standardized | Depressive disorders | Rate | 2021 | 3764.829728 | 4617.212391 | 3106.999389 |
| Incidence | Tropical Latin America | Both | Age-standardized | Depressive disorders | Rate | 1990 | 4685.917018 | 5390.041915 | 4140.039711 |
| Incidence | Tropical Latin America | Both | Age-standardized | Depressive disorders | Rate | 1991 | 4661.494875 | 5355.793363 | 4128.628195 |
| Incidence | Tropical Latin America | Both | Age-standardized | Depressive disorders | Rate | 1992 | 4653.72087 | 5338.041075 | 4116.500492 |
| Incidence | Tropical Latin America | Both | Age-standardized | Depressive disorders | Rate | 1993 | 4659.31573 | 5336.590994 | 4128.906138 |
| Incidence | Tropical Latin America | Both | Age-standardized | Depressive disorders | Rate | 1994 | 4674.851934 | 5351.17198 | 4147.86749 |
| Incidence | Tropical Latin America | Both | Age-standardized | Depressive disorders | Rate | 1995 | 4696.958411 | 5377.366863 | 4160.755467 |
| Incidence | Tropical Latin America | Both | Age-standardized | Depressive disorders | Rate | 1996 | 4790.479308 | 5490.831286 | 4245.431469 |
| Incidence | Tropical Latin America | Both | Age-standardized | Depressive disorders | Rate | 1997 | 4979.50588 | 5708.238574 | 4414.203566 |
| Incidence | Tropical Latin America | Both | Age-standardized | Depressive disorders | Rate | 1998 | 5200.452869 | 5965.330456 | 4620.503824 |
| Incidence | Tropical Latin America | Both | Age-standardized | Depressive disorders | Rate | 1999 | 5389.717885 | 6173.87019 | 4788.921771 |
| Incidence | Tropical Latin America | Both | Age-standardized | Depressive disorders | Rate | 2000 | 5483.811153 | 6281.032498 | 4872.380398 |
| Incidence | Tropical Latin America | Both | Age-standardized | Depressive disorders | Rate | 2001 | 5508.680872 | 6310.454433 | 4901.270822 |
| Incidence | Tropical Latin America | Both | Age-standardized | Depressive disorders | Rate | 2002 | 5529.871253 | 6326.024825 | 4930.439654 |
| Incidence | Tropical Latin America | Both | Age-standardized | Depressive disorders | Rate | 2003 | 5543.909021 | 6338.660917 | 4934.093365 |
| Incidence | Tropical Latin America | Both | Age-standardized | Depressive disorders | Rate | 2004 | 5547.321152 | 6337.195714 | 4934.959177 |
| Incidence | Tropical Latin America | Both | Age-standardized | Depressive disorders | Rate | 2005 | 5536.542396 | 6325.505718 | 4921.999502 |
| Incidence | Tropical Latin America | Both | Age-standardized | Depressive disorders | Rate | 2006 | 5428.288792 | 6180.247159 | 4846.113543 |
| Incidence | Tropical Latin America | Both | Age-standardized | Depressive disorders | Rate | 2007 | 5193.239388 | 5871.067526 | 4669.635902 |
| Incidence | Tropical Latin America | Both | Age-standardized | Depressive disorders | Rate | 2008 | 4908.857485 | 5503.611066 | 4439.673133 |
| Incidence | Tropical Latin America | Both | Age-standardized | Depressive disorders | Rate | 2009 | 4652.714941 | 5174.550948 | 4235.193791 |
| Incidence | Tropical Latin America | Both | Age-standardized | Depressive disorders | Rate | 2010 | 4502.378616 | 4987.999255 | 4100.962914 |
| Incidence | Tropical Latin America | Both | Age-standardized | Depressive disorders | Rate | 2011 | 4432.77664 | 4919.005238 | 4039.385649 |
| Incidence | Tropical Latin America | Both | Age-standardized | Depressive disorders | Rate | 2012 | 4368.965019 | 4867.444711 | 3973.921362 |
| Incidence | Tropical Latin America | Both | Age-standardized | Depressive disorders | Rate | 2013 | 4313.58672 | 4832.9648 | 3909.574108 |
| Incidence | Tropical Latin America | Both | Age-standardized | Depressive disorders | Rate | 2014 | 4269.33864 | 4815.073255 | 3858.044534 |
| Incidence | Tropical Latin America | Both | Age-standardized | Depressive disorders | Rate | 2015 | 4238.781536 | 4806.439559 | 3820.43251 |
| Incidence | Tropical Latin America | Both | Age-standardized | Depressive disorders | Rate | 2016 | 4214.844278 | 4780.851886 | 3790.84691 |
| Incidence | Tropical Latin America | Both | Age-standardized | Depressive disorders | Rate | 2017 | 4191.420561 | 4749.455847 | 3762.666271 |
| Incidence | Tropical Latin America | Both | Age-standardized | Depressive disorders | Rate | 2018 | 4172.667852 | 4731.686858 | 3739.33927 |
| Incidence | Tropical Latin America | Both | Age-standardized | Depressive disorders | Rate | 2019 | 4162.722845 | 4724.111385 | 3719.855765 |
| Incidence | Tropical Latin America | Both | Age-standardized | Depressive disorders | Rate | 2020 | 5004.201884 | 5753.592992 | 4403.840901 |
| Incidence | Tropical Latin America | Both | Age-standardized | Depressive disorders | Rate | 2021 | 5317.594576 | 6168.766832 | 4592.415857 |
| Incidence | Caribbean | Both | Age-standardized | Depressive disorders | Rate | 1990 | 4605.729311 | 5390.403564 | 3941.074275 |
| Incidence | Caribbean | Both | Age-standardized | Depressive disorders | Rate | 1991 | 4581.474852 | 5358.754608 | 3938.369045 |
| Incidence | Caribbean | Both | Age-standardized | Depressive disorders | Rate | 1992 | 4555.013215 | 5323.994319 | 3924.841585 |
| Incidence | Caribbean | Both | Age-standardized | Depressive disorders | Rate | 1993 | 4527.457948 | 5298.720731 | 3915.330825 |
| Incidence | Caribbean | Both | Age-standardized | Depressive disorders | Rate | 1994 | 4499.767214 | 5278.654265 | 3901.133667 |
| Incidence | Caribbean | Both | Age-standardized | Depressive disorders | Rate | 1995 | 4473.207757 | 5253.833436 | 3881.820814 |
| Incidence | Caribbean | Both | Age-standardized | Depressive disorders | Rate | 1996 | 4446.70846 | 5192.436585 | 3856.686805 |
| Incidence | Caribbean | Both | Age-standardized | Depressive disorders | Rate | 1997 | 4419.432842 | 5137.159677 | 3847.010643 |
| Incidence | Caribbean | Both | Age-standardized | Depressive disorders | Rate | 1998 | 4392.18568 | 5090.446156 | 3828.021143 |
| Incidence | Caribbean | Both | Age-standardized | Depressive disorders | Rate | 1999 | 4365.307277 | 5070.459619 | 3813.313423 |
| Incidence | Caribbean | Both | Age-standardized | Depressive disorders | Rate | 2000 | 4338.973701 | 5037.239952 | 3778.598713 |
| Incidence | Caribbean | Both | Age-standardized | Depressive disorders | Rate | 2001 | 4314.435627 | 4999.654386 | 3773.186229 |
| Incidence | Caribbean | Both | Age-standardized | Depressive disorders | Rate | 2002 | 4292.311004 | 4973.098591 | 3762.353181 |
| Incidence | Caribbean | Both | Age-standardized | Depressive disorders | Rate | 2003 | 4271.296288 | 4953.37522 | 3754.748897 |
| Incidence | Caribbean | Both | Age-standardized | Depressive disorders | Rate | 2004 | 4249.923727 | 4915.121028 | 3728.329213 |
| Incidence | Caribbean | Both | Age-standardized | Depressive disorders | Rate | 2005 | 4227.011029 | 4890.365533 | 3708.184694 |
| Incidence | Caribbean | Both | Age-standardized | Depressive disorders | Rate | 2006 | 4193.450052 | 4845.341122 | 3677.854796 |
| Incidence | Caribbean | Both | Age-standardized | Depressive disorders | Rate | 2007 | 4148.26709 | 4785.390705 | 3641.66503 |
| Incidence | Caribbean | Both | Age-standardized | Depressive disorders | Rate | 2008 | 4102.058577 | 4717.907458 | 3603.684767 |
| Incidence | Caribbean | Both | Age-standardized | Depressive disorders | Rate | 2009 | 4065.52323 | 4675.584325 | 3573.922288 |
| Incidence | Caribbean | Both | Age-standardized | Depressive disorders | Rate | 2010 | 4048.720431 | 4659.343037 | 3553.710031 |
| Incidence | Caribbean | Both | Age-standardized | Depressive disorders | Rate | 2011 | 4045.93614 | 4657.134885 | 3544.112622 |
| Incidence | Caribbean | Both | Age-standardized | Depressive disorders | Rate | 2012 | 4044.959271 | 4671.294461 | 3533.051001 |
| Incidence | Caribbean | Both | Age-standardized | Depressive disorders | Rate | 2013 | 4045.593425 | 4694.887849 | 3525.024659 |
| Incidence | Caribbean | Both | Age-standardized | Depressive disorders | Rate | 2014 | 4048.358818 | 4718.167069 | 3507.154654 |
| Incidence | Caribbean | Both | Age-standardized | Depressive disorders | Rate | 2015 | 4053.793598 | 4744.412031 | 3501.245623 |
| Incidence | Caribbean | Both | Age-standardized | Depressive disorders | Rate | 2016 | 4061.045005 | 4769.381731 | 3498.85468 |
| Incidence | Caribbean | Both | Age-standardized | Depressive disorders | Rate | 2017 | 4070.084718 | 4811.07575 | 3488.814559 |
| Incidence | Caribbean | Both | Age-standardized | Depressive disorders | Rate | 2018 | 4079.716574 | 4857.804233 | 3480.512001 |
| Incidence | Caribbean | Both | Age-standardized | Depressive disorders | Rate | 2019 | 4088.837412 | 4900.775217 | 3494.133964 |
| Incidence | Caribbean | Both | Age-standardized | Depressive disorders | Rate | 2020 | 4777.653638 | 5843.597866 | 3945.615358 |
| Incidence | Caribbean | Both | Age-standardized | Depressive disorders | Rate | 2021 | 4956.525321 | 6075.476881 | 4094.79706 |
| Incidence | Central Sub-Saharan Africa | Both | Age-standardized | Depressive disorders | Rate | 1990 | 7328.069762 | 8840.975892 | 6128.837799 |
| Incidence | Central Sub-Saharan Africa | Both | Age-standardized | Depressive disorders | Rate | 1991 | 7310.85151 | 8780.313071 | 6146.566811 |
| Incidence | Central Sub-Saharan Africa | Both | Age-standardized | Depressive disorders | Rate | 1992 | 7294.933097 | 8752.459657 | 6121.510285 |
| Incidence | Central Sub-Saharan Africa | Both | Age-standardized | Depressive disorders | Rate | 1993 | 7280.856024 | 8707.529727 | 6114.870745 |
| Incidence | Central Sub-Saharan Africa | Both | Age-standardized | Depressive disorders | Rate | 1994 | 7269.138913 | 8630.596428 | 6116.481963 |
| Incidence | Central Sub-Saharan Africa | Both | Age-standardized | Depressive disorders | Rate | 1995 | 7260.601565 | 8589.323635 | 6105.884151 |
| Incidence | Central Sub-Saharan Africa | Both | Age-standardized | Depressive disorders | Rate | 1996 | 7254.573077 | 8594.268345 | 6132.813249 |
| Incidence | Central Sub-Saharan Africa | Both | Age-standardized | Depressive disorders | Rate | 1997 | 7249.577668 | 8571.429937 | 6142.729621 |
| Incidence | Central Sub-Saharan Africa | Both | Age-standardized | Depressive disorders | Rate | 1998 | 7244.904978 | 8571.583731 | 6160.937738 |
| Incidence | Central Sub-Saharan Africa | Both | Age-standardized | Depressive disorders | Rate | 1999 | 7240.032933 | 8584.3868 | 6149.183069 |
| Incidence | Central Sub-Saharan Africa | Both | Age-standardized | Depressive disorders | Rate | 2000 | 7234.638983 | 8619.265205 | 6148.003927 |
| Incidence | Central Sub-Saharan Africa | Both | Age-standardized | Depressive disorders | Rate | 2001 | 7226.675233 | 8556.773158 | 6154.070135 |
| Incidence | Central Sub-Saharan Africa | Both | Age-standardized | Depressive disorders | Rate | 2002 | 7215.481217 | 8563.160723 | 6145.042745 |
| Incidence | Central Sub-Saharan Africa | Both | Age-standardized | Depressive disorders | Rate | 2003 | 7202.230269 | 8561.23253 | 6140.911123 |
| Incidence | Central Sub-Saharan Africa | Both | Age-standardized | Depressive disorders | Rate | 2004 | 7188.178436 | 8567.927154 | 6113.928563 |
| Incidence | Central Sub-Saharan Africa | Both | Age-standardized | Depressive disorders | Rate | 2005 | 7174.80991 | 8562.654045 | 6092.335052 |
| Incidence | Central Sub-Saharan Africa | Both | Age-standardized | Depressive disorders | Rate | 2006 | 7154.603127 | 8509.752943 | 6097.039495 |
| Incidence | Central Sub-Saharan Africa | Both | Age-standardized | Depressive disorders | Rate | 2007 | 7124.706465 | 8426.869633 | 6089.18097 |
| Incidence | Central Sub-Saharan Africa | Both | Age-standardized | Depressive disorders | Rate | 2008 | 7092.466731 | 8365.698912 | 6080.105188 |
| Incidence | Central Sub-Saharan Africa | Both | Age-standardized | Depressive disorders | Rate | 2009 | 7065.454128 | 8338.590306 | 6049.923467 |
| Incidence | Central Sub-Saharan Africa | Both | Age-standardized | Depressive disorders | Rate | 2010 | 7051.276025 | 8291.51444 | 6053.889494 |
| Incidence | Central Sub-Saharan Africa | Both | Age-standardized | Depressive disorders | Rate | 2011 | 7047.677122 | 8301.025126 | 6021.566261 |
| Incidence | Central Sub-Saharan Africa | Both | Age-standardized | Depressive disorders | Rate | 2012 | 7046.936045 | 8325.284849 | 6039.632533 |
| Incidence | Central Sub-Saharan Africa | Both | Age-standardized | Depressive disorders | Rate | 2013 | 7048.397687 | 8328.614976 | 6020.540964 |
| Incidence | Central Sub-Saharan Africa | Both | Age-standardized | Depressive disorders | Rate | 2014 | 7051.06528 | 8363.358234 | 6036.827104 |
| Incidence | Central Sub-Saharan Africa | Both | Age-standardized | Depressive disorders | Rate | 2015 | 7054.456922 | 8444.601662 | 6017.850329 |
| Incidence | Central Sub-Saharan Africa | Both | Age-standardized | Depressive disorders | Rate | 2016 | 7060.703046 | 8441.510769 | 6019.282073 |
| Incidence | Central Sub-Saharan Africa | Both | Age-standardized | Depressive disorders | Rate | 2017 | 7069.8222 | 8442.322146 | 6022.926793 |
| Incidence | Central Sub-Saharan Africa | Both | Age-standardized | Depressive disorders | Rate | 2018 | 7078.28497 | 8437.28338 | 6036.369844 |
| Incidence | Central Sub-Saharan Africa | Both | Age-standardized | Depressive disorders | Rate | 2019 | 7082.865086 | 8504.158694 | 6015.897956 |
| Incidence | Central Sub-Saharan Africa | Both | Age-standardized | Depressive disorders | Rate | 2020 | 7955.1595 | 9929.136618 | 6387.818615 |
| Incidence | Central Sub-Saharan Africa | Both | Age-standardized | Depressive disorders | Rate | 2021 | 7703.41432 | 9565.937104 | 6194.217846 |
| Incidence | Eastern Sub-Saharan Africa | Both | Age-standardized | Depressive disorders | Rate | 1990 | 6061.258697 | 7132.989373 | 5226.476154 |
| Incidence | Eastern Sub-Saharan Africa | Both | Age-standardized | Depressive disorders | Rate | 1991 | 6071.022295 | 7118.957299 | 5253.771026 |
| Incidence | Eastern Sub-Saharan Africa | Both | Age-standardized | Depressive disorders | Rate | 1992 | 6081.327418 | 7114.407142 | 5283.420212 |
| Incidence | Eastern Sub-Saharan Africa | Both | Age-standardized | Depressive disorders | Rate | 1993 | 6091.559807 | 7108.380432 | 5312.827094 |
| Incidence | Eastern Sub-Saharan Africa | Both | Age-standardized | Depressive disorders | Rate | 1994 | 6098.594071 | 7097.422139 | 5334.96546 |
| Incidence | Eastern Sub-Saharan Africa | Both | Age-standardized | Depressive disorders | Rate | 1995 | 6101.529798 | 7090.319291 | 5347.782344 |
| Incidence | Eastern Sub-Saharan Africa | Both | Age-standardized | Depressive disorders | Rate | 1996 | 6114.33482 | 7089.973634 | 5367.813398 |
| Incidence | Eastern Sub-Saharan Africa | Both | Age-standardized | Depressive disorders | Rate | 1997 | 6138.00189 | 7103.666676 | 5394.490447 |
| Incidence | Eastern Sub-Saharan Africa | Both | Age-standardized | Depressive disorders | Rate | 1998 | 6161.615096 | 7109.094299 | 5425.618671 |
| Incidence | Eastern Sub-Saharan Africa | Both | Age-standardized | Depressive disorders | Rate | 1999 | 6177.331979 | 7109.204389 | 5446.08697 |
| Incidence | Eastern Sub-Saharan Africa | Both | Age-standardized | Depressive disorders | Rate | 2000 | 6178.053775 | 7107.13942 | 5455.299903 |
| Incidence | Eastern Sub-Saharan Africa | Both | Age-standardized | Depressive disorders | Rate | 2001 | 6166.466579 | 7095.871552 | 5444.340378 |
| Incidence | Eastern Sub-Saharan Africa | Both | Age-standardized | Depressive disorders | Rate | 2002 | 6149.24448 | 7086.458551 | 5428.794687 |
| Incidence | Eastern Sub-Saharan Africa | Both | Age-standardized | Depressive disorders | Rate | 2003 | 6127.88914 | 7062.449178 | 5416.086338 |
| Incidence | Eastern Sub-Saharan Africa | Both | Age-standardized | Depressive disorders | Rate | 2004 | 6104.066782 | 7040.997107 | 5396.410033 |
| Incidence | Eastern Sub-Saharan Africa | Both | Age-standardized | Depressive disorders | Rate | 2005 | 6078.981588 | 7015.430978 | 5371.458673 |
| Incidence | Eastern Sub-Saharan Africa | Both | Age-standardized | Depressive disorders | Rate | 2006 | 6041.201127 | 6956.389166 | 5351.952332 |
| Incidence | Eastern Sub-Saharan Africa | Both | Age-standardized | Depressive disorders | Rate | 2007 | 5983.885818 | 6885.730061 | 5295.439052 |
| Incidence | Eastern Sub-Saharan Africa | Both | Age-standardized | Depressive disorders | Rate | 2008 | 5919.062889 | 6800.519106 | 5241.639206 |
| Incidence | Eastern Sub-Saharan Africa | Both | Age-standardized | Depressive disorders | Rate | 2009 | 5858.928166 | 6721.475638 | 5177.822308 |
| Incidence | Eastern Sub-Saharan Africa | Both | Age-standardized | Depressive disorders | Rate | 2010 | 5815.452675 | 6667.369618 | 5133.069191 |
| Incidence | Eastern Sub-Saharan Africa | Both | Age-standardized | Depressive disorders | Rate | 2011 | 5781.966769 | 6647.69315 | 5092.180374 |
| Incidence | Eastern Sub-Saharan Africa | Both | Age-standardized | Depressive disorders | Rate | 2012 | 5745.98729 | 6621.830542 | 5054.570097 |
| Incidence | Eastern Sub-Saharan Africa | Both | Age-standardized | Depressive disorders | Rate | 2013 | 5713.322055 | 6603.080303 | 5021.747788 |
| Incidence | Eastern Sub-Saharan Africa | Both | Age-standardized | Depressive disorders | Rate | 2014 | 5690.05995 | 6607.289907 | 5001.145688 |
| Incidence | Eastern Sub-Saharan Africa | Both | Age-standardized | Depressive disorders | Rate | 2015 | 5682.075719 | 6611.900779 | 4998.660054 |
| Incidence | Eastern Sub-Saharan Africa | Both | Age-standardized | Depressive disorders | Rate | 2016 | 5687.794545 | 6625.139218 | 4991.273182 |
| Incidence | Eastern Sub-Saharan Africa | Both | Age-standardized | Depressive disorders | Rate | 2017 | 5698.284718 | 6638.949051 | 4990.028563 |
| Incidence | Eastern Sub-Saharan Africa | Both | Age-standardized | Depressive disorders | Rate | 2018 | 5708.831061 | 6660.445838 | 4980.869276 |
| Incidence | Eastern Sub-Saharan Africa | Both | Age-standardized | Depressive disorders | Rate | 2019 | 5715.003972 | 6692.351635 | 4961.926543 |
| Incidence | Eastern Sub-Saharan Africa | Both | Age-standardized | Depressive disorders | Rate | 2020 | 6244.774801 | 7332.615071 | 5360.444746 |
| Incidence | Eastern Sub-Saharan Africa | Both | Age-standardized | Depressive disorders | Rate | 2021 | 6468.120973 | 7580.312231 | 5519.803186 |
| Incidence | South Asia | Both | Age-standardized | Depressive disorders | Rate | 1990 | 4876.284927 | 5689.876623 | 4256.821869 |
| Incidence | South Asia | Both | Age-standardized | Depressive disorders | Rate | 1991 | 5022.60336 | 5848.5012 | 4391.327058 |
| Incidence | South Asia | Both | Age-standardized | Depressive disorders | Rate | 1992 | 5151.161449 | 5989.777243 | 4512.706371 |
| Incidence | South Asia | Both | Age-standardized | Depressive disorders | Rate | 1993 | 5254.443141 | 6089.224131 | 4609.789705 |
| Incidence | South Asia | Both | Age-standardized | Depressive disorders | Rate | 1994 | 5324.878267 | 6169.815163 | 4678.290192 |
| Incidence | South Asia | Both | Age-standardized | Depressive disorders | Rate | 1995 | 5354.730135 | 6201.952549 | 4714.964632 |
| Incidence | South Asia | Both | Age-standardized | Depressive disorders | Rate | 1996 | 5348.393475 | 6189.697 | 4706.340744 |
| Incidence | South Asia | Both | Age-standardized | Depressive disorders | Rate | 1997 | 5323.474536 | 6156.269381 | 4674.567138 |
| Incidence | South Asia | Both | Age-standardized | Depressive disorders | Rate | 1998 | 5292.016389 | 6114.945086 | 4637.610969 |
| Incidence | South Asia | Both | Age-standardized | Depressive disorders | Rate | 1999 | 5266.033688 | 6082.144044 | 4608.856762 |
| Incidence | South Asia | Both | Age-standardized | Depressive disorders | Rate | 2000 | 5257.730705 | 6074.884888 | 4597.254839 |
| Incidence | South Asia | Both | Age-standardized | Depressive disorders | Rate | 2001 | 5278.135042 | 6098.20041 | 4616.491732 |
| Incidence | South Asia | Both | Age-standardized | Depressive disorders | Rate | 2002 | 5319.147407 | 6144.313436 | 4652.949351 |
| Incidence | South Asia | Both | Age-standardized | Depressive disorders | Rate | 2003 | 5364.97179 | 6205.171976 | 4701.606982 |
| Incidence | South Asia | Both | Age-standardized | Depressive disorders | Rate | 2004 | 5399.691334 | 6254.857528 | 4741.507439 |
| Incidence | South Asia | Both | Age-standardized | Depressive disorders | Rate | 2005 | 5407.029182 | 6269.10372 | 4757.973118 |
| Incidence | South Asia | Both | Age-standardized | Depressive disorders | Rate | 2006 | 5291.858485 | 6117.28608 | 4659.497907 |
| Incidence | South Asia | Both | Age-standardized | Depressive disorders | Rate | 2007 | 5033.660441 | 5779.646532 | 4443.106194 |
| Incidence | South Asia | Both | Age-standardized | Depressive disorders | Rate | 2008 | 4727.721662 | 5403.732999 | 4185.646994 |
| Incidence | South Asia | Both | Age-standardized | Depressive disorders | Rate | 2009 | 4468.345548 | 5087.407146 | 3959.55792 |
| Incidence | South Asia | Both | Age-standardized | Depressive disorders | Rate | 2010 | 4349.179043 | 4939.055911 | 3851.147029 |
| Incidence | South Asia | Both | Age-standardized | Depressive disorders | Rate | 2011 | 4335.628749 | 4932.406849 | 3843.666451 |
| Incidence | South Asia | Both | Age-standardized | Depressive disorders | Rate | 2012 | 4329.162998 | 4935.615973 | 3829.115725 |
| Incidence | South Asia | Both | Age-standardized | Depressive disorders | Rate | 2013 | 4327.391072 | 4945.100181 | 3814.346462 |
| Incidence | South Asia | Both | Age-standardized | Depressive disorders | Rate | 2014 | 4327.930796 | 4955.74422 | 3803.210269 |
| Incidence | South Asia | Both | Age-standardized | Depressive disorders | Rate | 2015 | 4327.878153 | 4966.772204 | 3796.372598 |
| Incidence | South Asia | Both | Age-standardized | Depressive disorders | Rate | 2016 | 4327.592749 | 4971.410302 | 3803.505828 |
| Incidence | South Asia | Both | Age-standardized | Depressive disorders | Rate | 2017 | 4328.823312 | 4982.29944 | 3813.648487 |
| Incidence | South Asia | Both | Age-standardized | Depressive disorders | Rate | 2018 | 4330.568193 | 5011.248433 | 3804.945906 |
| Incidence | South Asia | Both | Age-standardized | Depressive disorders | Rate | 2019 | 4331.587391 | 5034.627086 | 3790.522868 |
| Incidence | South Asia | Both | Age-standardized | Depressive disorders | Rate | 2020 | 5141.994907 | 6057.337134 | 4469.083156 |
| Incidence | South Asia | Both | Age-standardized | Depressive disorders | Rate | 2021 | 5150.99858 | 6036.893936 | 4461.449688 |
| Incidence | Western Sub-Saharan Africa | Both | Age-standardized | Depressive disorders | Rate | 1990 | 4745.999645 | 5549.906259 | 4112.432419 |
| Incidence | Western Sub-Saharan Africa | Both | Age-standardized | Depressive disorders | Rate | 1991 | 4737.480589 | 5527.966579 | 4110.768869 |
| Incidence | Western Sub-Saharan Africa | Both | Age-standardized | Depressive disorders | Rate | 1992 | 4732.616526 | 5516.122999 | 4108.343867 |
| Incidence | Western Sub-Saharan Africa | Both | Age-standardized | Depressive disorders | Rate | 1993 | 4730.771361 | 5497.670749 | 4114.319593 |
| Incidence | Western Sub-Saharan Africa | Both | Age-standardized | Depressive disorders | Rate | 1994 | 4731.118785 | 5489.222028 | 4115.200496 |
| Incidence | Western Sub-Saharan Africa | Both | Age-standardized | Depressive disorders | Rate | 1995 | 4733.139825 | 5489.210953 | 4113.07189 |
| Incidence | Western Sub-Saharan Africa | Both | Age-standardized | Depressive disorders | Rate | 1996 | 4747.773523 | 5516.567624 | 4132.246343 |
| Incidence | Western Sub-Saharan Africa | Both | Age-standardized | Depressive disorders | Rate | 1997 | 4778.852403 | 5544.486263 | 4168.903131 |
| Incidence | Western Sub-Saharan Africa | Both | Age-standardized | Depressive disorders | Rate | 1998 | 4815.533731 | 5574.035036 | 4209.619663 |
| Incidence | Western Sub-Saharan Africa | Both | Age-standardized | Depressive disorders | Rate | 1999 | 4847.333734 | 5600.823619 | 4246.003603 |
| Incidence | Western Sub-Saharan Africa | Both | Age-standardized | Depressive disorders | Rate | 2000 | 4863.97394 | 5619.265216 | 4262.450755 |
| Incidence | Western Sub-Saharan Africa | Both | Age-standardized | Depressive disorders | Rate | 2001 | 4871.436257 | 5630.061795 | 4269.172414 |
| Incidence | Western Sub-Saharan Africa | Both | Age-standardized | Depressive disorders | Rate | 2002 | 4880.830378 | 5638.297498 | 4278.125745 |
| Incidence | Western Sub-Saharan Africa | Both | Age-standardized | Depressive disorders | Rate | 2003 | 4889.640094 | 5639.207589 | 4287.872355 |
| Incidence | Western Sub-Saharan Africa | Both | Age-standardized | Depressive disorders | Rate | 2004 | 4895.716579 | 5641.206328 | 4298.515696 |
| Incidence | Western Sub-Saharan Africa | Both | Age-standardized | Depressive disorders | Rate | 2005 | 4896.843706 | 5638.049483 | 4301.206956 |
| Incidence | Western Sub-Saharan Africa | Both | Age-standardized | Depressive disorders | Rate | 2006 | 4873.639525 | 5599.451719 | 4281.13902 |
| Incidence | Western Sub-Saharan Africa | Both | Age-standardized | Depressive disorders | Rate | 2007 | 4819.90196 | 5523.93955 | 4235.566076 |
| Incidence | Western Sub-Saharan Africa | Both | Age-standardized | Depressive disorders | Rate | 2008 | 4752.760738 | 5438.496691 | 4178.091429 |
| Incidence | Western Sub-Saharan Africa | Both | Age-standardized | Depressive disorders | Rate | 2009 | 4689.647805 | 5360.088215 | 4127.182784 |
| Incidence | Western Sub-Saharan Africa | Both | Age-standardized | Depressive disorders | Rate | 2010 | 4647.82987 | 5312.904405 | 4094.072749 |
| Incidence | Western Sub-Saharan Africa | Both | Age-standardized | Depressive disorders | Rate | 2011 | 4617.8203 | 5282.937421 | 4059.30419 |
| Incidence | Western Sub-Saharan Africa | Both | Age-standardized | Depressive disorders | Rate | 2012 | 4583.046241 | 5259.755156 | 4022.721072 |
| Incidence | Western Sub-Saharan Africa | Both | Age-standardized | Depressive disorders | Rate | 2013 | 4550.09701 | 5242.611373 | 3981.454178 |
| Incidence | Western Sub-Saharan Africa | Both | Age-standardized | Depressive disorders | Rate | 2014 | 4526.003971 | 5227.744634 | 3952.334616 |
| Incidence | Western Sub-Saharan Africa | Both | Age-standardized | Depressive disorders | Rate | 2015 | 4517.965287 | 5226.448403 | 3939.685714 |
| Incidence | Western Sub-Saharan Africa | Both | Age-standardized | Depressive disorders | Rate | 2016 | 4520.433801 | 5237.416311 | 3927.576437 |
| Incidence | Western Sub-Saharan Africa | Both | Age-standardized | Depressive disorders | Rate | 2017 | 4522.32887 | 5256.769341 | 3922.1069 |
| Incidence | Western Sub-Saharan Africa | Both | Age-standardized | Depressive disorders | Rate | 2018 | 4523.788091 | 5275.264 | 3919.05182 |
| Incidence | Western Sub-Saharan Africa | Both | Age-standardized | Depressive disorders | Rate | 2019 | 4525.081257 | 5289.571998 | 3914.51157 |
| Incidence | Western Sub-Saharan Africa | Both | Age-standardized | Depressive disorders | Rate | 2020 | 4892.267274 | 5719.271701 | 4174.32428 |
| Incidence | Western Sub-Saharan Africa | Both | Age-standardized | Depressive disorders | Rate | 2021 | 4739.638423 | 5556.82845 | 4046.237948 |
| Incidence | Southern Sub-Saharan Africa | Both | Age-standardized | Depressive disorders | Rate | 1990 | 4708.444831 | 5473.824604 | 4129.941845 |
| Incidence | Southern Sub-Saharan Africa | Both | Age-standardized | Depressive disorders | Rate | 1991 | 4645.436428 | 5385.149309 | 4102.475318 |
| Incidence | Southern Sub-Saharan Africa | Both | Age-standardized | Depressive disorders | Rate | 1992 | 4587.582776 | 5301.318356 | 4058.878634 |
| Incidence | Southern Sub-Saharan Africa | Both | Age-standardized | Depressive disorders | Rate | 1993 | 4538.622182 | 5227.728868 | 4025.340064 |
| Incidence | Southern Sub-Saharan Africa | Both | Age-standardized | Depressive disorders | Rate | 1994 | 4502.19841 | 5181.262657 | 3990.204183 |
| Incidence | Southern Sub-Saharan Africa | Both | Age-standardized | Depressive disorders | Rate | 1995 | 4482.23469 | 5132.13848 | 3980.622289 |
| Incidence | Southern Sub-Saharan Africa | Both | Age-standardized | Depressive disorders | Rate | 1996 | 4470.078364 | 5090.82725 | 3970.873374 |
| Incidence | Southern Sub-Saharan Africa | Both | Age-standardized | Depressive disorders | Rate | 1997 | 4455.817417 | 5051.722073 | 3964.027125 |
| Incidence | Southern Sub-Saharan Africa | Both | Age-standardized | Depressive disorders | Rate | 1998 | 4441.565639 | 5031.076548 | 3955.682147 |
| Incidence | Southern Sub-Saharan Africa | Both | Age-standardized | Depressive disorders | Rate | 1999 | 4429.217127 | 5012.207908 | 3942.512714 |
| Incidence | Southern Sub-Saharan Africa | Both | Age-standardized | Depressive disorders | Rate | 2000 | 4420.758066 | 5003.096001 | 3929.768062 |
| Incidence | Southern Sub-Saharan Africa | Both | Age-standardized | Depressive disorders | Rate | 2001 | 4409.557141 | 4988.148236 | 3916.943188 |
| Incidence | Southern Sub-Saharan Africa | Both | Age-standardized | Depressive disorders | Rate | 2002 | 4390.972386 | 4964.523221 | 3905.042154 |
| Incidence | Southern Sub-Saharan Africa | Both | Age-standardized | Depressive disorders | Rate | 2003 | 4370.790008 | 4956.694602 | 3897.374542 |
| Incidence | Southern Sub-Saharan Africa | Both | Age-standardized | Depressive disorders | Rate | 2004 | 4354.990538 | 4946.159406 | 3892.173087 |
| Incidence | Southern Sub-Saharan Africa | Both | Age-standardized | Depressive disorders | Rate | 2005 | 4349.725779 | 4951.097895 | 3883.987104 |
| Incidence | Southern Sub-Saharan Africa | Both | Age-standardized | Depressive disorders | Rate | 2006 | 4358.102059 | 4944.935334 | 3902.173238 |
| Incidence | Southern Sub-Saharan Africa | Both | Age-standardized | Depressive disorders | Rate | 2007 | 4376.274643 | 4949.389224 | 3925.983048 |
| Incidence | Southern Sub-Saharan Africa | Both | Age-standardized | Depressive disorders | Rate | 2008 | 4400.302783 | 4965.174053 | 3935.570519 |
| Incidence | Southern Sub-Saharan Africa | Both | Age-standardized | Depressive disorders | Rate | 2009 | 4426.235327 | 4988.164087 | 3944.730217 |
| Incidence | Southern Sub-Saharan Africa | Both | Age-standardized | Depressive disorders | Rate | 2010 | 4450.369788 | 5007.160761 | 3955.51438 |
| Incidence | Southern Sub-Saharan Africa | Both | Age-standardized | Depressive disorders | Rate | 2011 | 4484.296399 | 5043.424511 | 3992.942274 |
| Incidence | Southern Sub-Saharan Africa | Both | Age-standardized | Depressive disorders | Rate | 2012 | 4533.210215 | 5104.881619 | 4038.000751 |
| Incidence | Southern Sub-Saharan Africa | Both | Age-standardized | Depressive disorders | Rate | 2013 | 4584.758529 | 5186.538922 | 4075.590913 |
| Incidence | Southern Sub-Saharan Africa | Both | Age-standardized | Depressive disorders | Rate | 2014 | 4626.530484 | 5248.779222 | 4107.66168 |
| Incidence | Southern Sub-Saharan Africa | Both | Age-standardized | Depressive disorders | Rate | 2015 | 4646.168307 | 5277.903811 | 4114.994743 |
| Incidence | Southern Sub-Saharan Africa | Both | Age-standardized | Depressive disorders | Rate | 2016 | 4653.64507 | 5308.903215 | 4123.137494 |
| Incidence | Southern Sub-Saharan Africa | Both | Age-standardized | Depressive disorders | Rate | 2017 | 4664.86801 | 5346.244043 | 4132.445468 |
| Incidence | Southern Sub-Saharan Africa | Both | Age-standardized | Depressive disorders | Rate | 2018 | 4677.215394 | 5374.955646 | 4136.017734 |
| Incidence | Southern Sub-Saharan Africa | Both | Age-standardized | Depressive disorders | Rate | 2019 | 4687.827023 | 5419.520791 | 4131.426228 |
| Incidence | Southern Sub-Saharan Africa | Both | Age-standardized | Depressive disorders | Rate | 2020 | 5598.249995 | 6517.363051 | 4817.143575 |
| Incidence | Southern Sub-Saharan Africa | Both | Age-standardized | Depressive disorders | Rate | 2021 | 5878.90535 | 6920.415667 | 5041.115985 |
