# Supplementary material for: Unmasking the rising global burden of depression: A 32-year GBD analysis of gender disparities and regional hotspots in Sub-Saharan Africa
Source: PLoS One. 2025 Jul 31;20(7):e0326974. doi: 10.1371/journal.pone.0326974 (PMC12312894; doi:10.1371/journal.pone.0326974)
Supplement: S12 Table — (DOCX) [file pone.0326974.s011.docx]

| **Supplementary Table 12 Global and Regional Time Series Data on Depression Prevalence (1990-2021)** | | | | | | | | | |
| --- | --- | --- | --- | --- | --- | --- | --- | --- | --- |
| **measure** | **location** | **sex** | **age** | **cause** | **metric** | **year** | **value** | **upper** | **lower** |
| Prevalence | East Asia | Both | Age-standardized | Depressive disorders | Rate | 1990 | 3059.655619 | 3392.613125 | 2765.455149 |
| Prevalence | East Asia | Both | Age-standardized | Depressive disorders | Rate | 1991 | 3112.690946 | 3447.713201 | 2819.401756 |
| Prevalence | East Asia | Both | Age-standardized | Depressive disorders | Rate | 1992 | 3153.652247 | 3490.522077 | 2860.741302 |
| Prevalence | East Asia | Both | Age-standardized | Depressive disorders | Rate | 1993 | 3181.603005 | 3520.972615 | 2885.53818 |
| Prevalence | East Asia | Both | Age-standardized | Depressive disorders | Rate | 1994 | 3195.43221 | 3539.476194 | 2901.623076 |
| Prevalence | East Asia | Both | Age-standardized | Depressive disorders | Rate | 1995 | 3194.224104 | 3542.389105 | 2899.4026 |
| Prevalence | East Asia | Both | Age-standardized | Depressive disorders | Rate | 1996 | 3175.695247 | 3516.959855 | 2888.800769 |
| Prevalence | East Asia | Both | Age-standardized | Depressive disorders | Rate | 1997 | 3143.463924 | 3479.366993 | 2860.890279 |
| Prevalence | East Asia | Both | Age-standardized | Depressive disorders | Rate | 1998 | 3105.712978 | 3436.663343 | 2825.873973 |
| Prevalence | East Asia | Both | Age-standardized | Depressive disorders | Rate | 1999 | 3070.4932 | 3396.829403 | 2787.998905 |
| Prevalence | East Asia | Both | Age-standardized | Depressive disorders | Rate | 2000 | 3045.948916 | 3369.130164 | 2757.733025 |
| Prevalence | East Asia | Both | Age-standardized | Depressive disorders | Rate | 2001 | 3033.45361 | 3358.162394 | 2745.680662 |
| Prevalence | East Asia | Both | Age-standardized | Depressive disorders | Rate | 2002 | 3026.670238 | 3352.045502 | 2740.371888 |
| Prevalence | East Asia | Both | Age-standardized | Depressive disorders | Rate | 2003 | 3022.350763 | 3349.290775 | 2737.557637 |
| Prevalence | East Asia | Both | Age-standardized | Depressive disorders | Rate | 2004 | 3017.079236 | 3341.050424 | 2732.49572 |
| Prevalence | East Asia | Both | Age-standardized | Depressive disorders | Rate | 2005 | 3007.460161 | 3327.618523 | 2723.129057 |
| Prevalence | East Asia | Both | Age-standardized | Depressive disorders | Rate | 2006 | 2985.764792 | 3304.963312 | 2701.634859 |
| Prevalence | East Asia | Both | Age-standardized | Depressive disorders | Rate | 2007 | 2952.214016 | 3261.147591 | 2669.247208 |
| Prevalence | East Asia | Both | Age-standardized | Depressive disorders | Rate | 2008 | 2915.608115 | 3215.783701 | 2635.837849 |
| Prevalence | East Asia | Both | Age-standardized | Depressive disorders | Rate | 2009 | 2884.622544 | 3181.506229 | 2608.790159 |
| Prevalence | East Asia | Both | Age-standardized | Depressive disorders | Rate | 2010 | 2867.918155 | 3168.906935 | 2595.849032 |
| Prevalence | East Asia | Both | Age-standardized | Depressive disorders | Rate | 2011 | 2861.166706 | 3163.257657 | 2589.776648 |
| Prevalence | East Asia | Both | Age-standardized | Depressive disorders | Rate | 2012 | 2854.709524 | 3164.786249 | 2583.538103 |
| Prevalence | East Asia | Both | Age-standardized | Depressive disorders | Rate | 2013 | 2849.443128 | 3162.904765 | 2570.615493 |
| Prevalence | East Asia | Both | Age-standardized | Depressive disorders | Rate | 2014 | 2846.114147 | 3161.675724 | 2568.302646 |
| Prevalence | East Asia | Both | Age-standardized | Depressive disorders | Rate | 2015 | 2845.634448 | 3164.147895 | 2566.861898 |
| Prevalence | East Asia | Both | Age-standardized | Depressive disorders | Rate | 2016 | 2842.760994 | 3161.526885 | 2564.292313 |
| Prevalence | East Asia | Both | Age-standardized | Depressive disorders | Rate | 2017 | 2834.445878 | 3152.74127 | 2557.939086 |
| Prevalence | East Asia | Both | Age-standardized | Depressive disorders | Rate | 2018 | 2825.111016 | 3141.115896 | 2551.086933 |
| Prevalence | East Asia | Both | Age-standardized | Depressive disorders | Rate | 2019 | 2819.014156 | 3134.147817 | 2543.0922 |
| Prevalence | East Asia | Both | Age-standardized | Depressive disorders | Rate | 2020 | 2877.249604 | 3194.861807 | 2588.630811 |
| Prevalence | East Asia | Both | Age-standardized | Depressive disorders | Rate | 2021 | 2870.607766 | 3205.354485 | 2583.726598 |
| Prevalence | Oceania | Both | Age-standardized | Depressive disorders | Rate | 1990 | 3116.257345 | 3653.805076 | 2709.626181 |
| Prevalence | Oceania | Both | Age-standardized | Depressive disorders | Rate | 1991 | 3112.338632 | 3630.468111 | 2709.156286 |
| Prevalence | Oceania | Both | Age-standardized | Depressive disorders | Rate | 1992 | 3108.982566 | 3624.598871 | 2712.969716 |
| Prevalence | Oceania | Both | Age-standardized | Depressive disorders | Rate | 1993 | 3106.011163 | 3607.928915 | 2713.416639 |
| Prevalence | Oceania | Both | Age-standardized | Depressive disorders | Rate | 1994 | 3103.501262 | 3597.529054 | 2708.219263 |
| Prevalence | Oceania | Both | Age-standardized | Depressive disorders | Rate | 1995 | 3101.54915 | 3584.255108 | 2700.714756 |
| Prevalence | Oceania | Both | Age-standardized | Depressive disorders | Rate | 1996 | 3099.395218 | 3590.00865 | 2697.784508 |
| Prevalence | Oceania | Both | Age-standardized | Depressive disorders | Rate | 1997 | 3096.540703 | 3588.721913 | 2695.426379 |
| Prevalence | Oceania | Both | Age-standardized | Depressive disorders | Rate | 1998 | 3093.424263 | 3591.740534 | 2691.139054 |
| Prevalence | Oceania | Both | Age-standardized | Depressive disorders | Rate | 1999 | 3090.774178 | 3595.550321 | 2695.021344 |
| Prevalence | Oceania | Both | Age-standardized | Depressive disorders | Rate | 2000 | 3089.396786 | 3593.542817 | 2694.63572 |
| Prevalence | Oceania | Both | Age-standardized | Depressive disorders | Rate | 2001 | 3089.706449 | 3592.012493 | 2696.418485 |
| Prevalence | Oceania | Both | Age-standardized | Depressive disorders | Rate | 2002 | 3090.910926 | 3595.566161 | 2697.559723 |
| Prevalence | Oceania | Both | Age-standardized | Depressive disorders | Rate | 2003 | 3092.127908 | 3593.446221 | 2700.261239 |
| Prevalence | Oceania | Both | Age-standardized | Depressive disorders | Rate | 2004 | 3092.632381 | 3586.408339 | 2699.136258 |
| Prevalence | Oceania | Both | Age-standardized | Depressive disorders | Rate | 2005 | 3091.879885 | 3588.631534 | 2696.841835 |
| Prevalence | Oceania | Both | Age-standardized | Depressive disorders | Rate | 2006 | 3086.902531 | 3578.780186 | 2694.185339 |
| Prevalence | Oceania | Both | Age-standardized | Depressive disorders | Rate | 2007 | 3077.058549 | 3565.768882 | 2685.551259 |
| Prevalence | Oceania | Both | Age-standardized | Depressive disorders | Rate | 2008 | 3065.527261 | 3547.962181 | 2678.481595 |
| Prevalence | Oceania | Both | Age-standardized | Depressive disorders | Rate | 2009 | 3055.563976 | 3531.877694 | 2668.88806 |
| Prevalence | Oceania | Both | Age-standardized | Depressive disorders | Rate | 2010 | 3050.450648 | 3528.350134 | 2661.211635 |
| Prevalence | Oceania | Both | Age-standardized | Depressive disorders | Rate | 2011 | 3049.723738 | 3526.315285 | 2659.465764 |
| Prevalence | Oceania | Both | Age-standardized | Depressive disorders | Rate | 2012 | 3050.346674 | 3534.76069 | 2660.583344 |
| Prevalence | Oceania | Both | Age-standardized | Depressive disorders | Rate | 2013 | 3051.68496 | 3537.552642 | 2666.325027 |
| Prevalence | Oceania | Both | Age-standardized | Depressive disorders | Rate | 2014 | 3053.198498 | 3534.95258 | 2665.784889 |
| Prevalence | Oceania | Both | Age-standardized | Depressive disorders | Rate | 2015 | 3054.312566 | 3547.454415 | 2664.789796 |
| Prevalence | Oceania | Both | Age-standardized | Depressive disorders | Rate | 2016 | 3054.801196 | 3556.420012 | 2664.487691 |
| Prevalence | Oceania | Both | Age-standardized | Depressive disorders | Rate | 2017 | 3055.017508 | 3558.567297 | 2657.576083 |
| Prevalence | Oceania | Both | Age-standardized | Depressive disorders | Rate | 2018 | 3055.233946 | 3552.521985 | 2657.413264 |
| Prevalence | Oceania | Both | Age-standardized | Depressive disorders | Rate | 2019 | 3055.876217 | 3552.482025 | 2655.673982 |
| Prevalence | Oceania | Both | Age-standardized | Depressive disorders | Rate | 2020 | 3075.593332 | 3650.909392 | 2557.444025 |
| Prevalence | Oceania | Both | Age-standardized | Depressive disorders | Rate | 2021 | 3201.990236 | 3811.000277 | 2654.700954 |
| Prevalence | Global | Both | Age-standardized | Depressive disorders | Rate | 1990 | 3599.665531 | 4023.20672 | 3251.906522 |
| Prevalence | Global | Both | Age-standardized | Depressive disorders | Rate | 1991 | 3630.736594 | 4053.472286 | 3282.194258 |
| Prevalence | Global | Both | Age-standardized | Depressive disorders | Rate | 1992 | 3657.911689 | 4077.105129 | 3304.415498 |
| Prevalence | Global | Both | Age-standardized | Depressive disorders | Rate | 1993 | 3679.8645 | 4095.015197 | 3322.360031 |
| Prevalence | Global | Both | Age-standardized | Depressive disorders | Rate | 1994 | 3695.493988 | 4106.13265 | 3336.33427 |
| Prevalence | Global | Both | Age-standardized | Depressive disorders | Rate | 1995 | 3703.458633 | 4112.330547 | 3348.096396 |
| Prevalence | Global | Both | Age-standardized | Depressive disorders | Rate | 1996 | 3705.809266 | 4112.876399 | 3350.948292 |
| Prevalence | Global | Both | Age-standardized | Depressive disorders | Rate | 1997 | 3704.720867 | 4108.450909 | 3352.359382 |
| Prevalence | Global | Both | Age-standardized | Depressive disorders | Rate | 1998 | 3701.789024 | 4102.070388 | 3353.347258 |
| Prevalence | Global | Both | Age-standardized | Depressive disorders | Rate | 1999 | 3698.933796 | 4096.10386 | 3353.655652 |
| Prevalence | Global | Both | Age-standardized | Depressive disorders | Rate | 2000 | 3697.21916 | 4093.933134 | 3354.199786 |
| Prevalence | Global | Both | Age-standardized | Depressive disorders | Rate | 2001 | 3698.955908 | 4096.947028 | 3356.544132 |
| Prevalence | Global | Both | Age-standardized | Depressive disorders | Rate | 2002 | 3702.788392 | 4103.569616 | 3360.318593 |
| Prevalence | Global | Both | Age-standardized | Depressive disorders | Rate | 2003 | 3707.036972 | 4110.733515 | 3364.315528 |
| Prevalence | Global | Both | Age-standardized | Depressive disorders | Rate | 2004 | 3709.966548 | 4117.084883 | 3366.523052 |
| Prevalence | Global | Both | Age-standardized | Depressive disorders | Rate | 2005 | 3709.094225 | 4117.971484 | 3366.474629 |
| Prevalence | Global | Both | Age-standardized | Depressive disorders | Rate | 2006 | 3688.945735 | 4093.34338 | 3348.70792 |
| Prevalence | Global | Both | Age-standardized | Depressive disorders | Rate | 2007 | 3644.376681 | 4044.544474 | 3308.538342 |
| Prevalence | Global | Both | Age-standardized | Depressive disorders | Rate | 2008 | 3591.568422 | 3976.785951 | 3263.504164 |
| Prevalence | Global | Both | Age-standardized | Depressive disorders | Rate | 2009 | 3547.161328 | 3919.982961 | 3224.363437 |
| Prevalence | Global | Both | Age-standardized | Depressive disorders | Rate | 2010 | 3527.340531 | 3893.161568 | 3205.657077 |
| Prevalence | Global | Both | Age-standardized | Depressive disorders | Rate | 2011 | 3527.276887 | 3897.500809 | 3204.931062 |
| Prevalence | Global | Both | Age-standardized | Depressive disorders | Rate | 2012 | 3529.362573 | 3908.677034 | 3203.873126 |
| Prevalence | Global | Both | Age-standardized | Depressive disorders | Rate | 2013 | 3533.258588 | 3923.748866 | 3201.339589 |
| Prevalence | Global | Both | Age-standardized | Depressive disorders | Rate | 2014 | 3538.700086 | 3940.667152 | 3200.088617 |
| Prevalence | Global | Both | Age-standardized | Depressive disorders | Rate | 2015 | 3544.936806 | 3948.517374 | 3200.205497 |
| Prevalence | Global | Both | Age-standardized | Depressive disorders | Rate | 2016 | 3550.665264 | 3957.423134 | 3200.317737 |
| Prevalence | Global | Both | Age-standardized | Depressive disorders | Rate | 2017 | 3555.109725 | 3971.927232 | 3201.067952 |
| Prevalence | Global | Both | Age-standardized | Depressive disorders | Rate | 2018 | 3559.437038 | 3986.8488 | 3204.575654 |
| Prevalence | Global | Both | Age-standardized | Depressive disorders | Rate | 2019 | 3564.77338 | 4002.895632 | 3211.820967 |
| Prevalence | Global | Both | Age-standardized | Depressive disorders | Rate | 2020 | 3969.98529 | 4500.56053 | 3554.020206 |
| Prevalence | Global | Both | Age-standardized | Depressive disorders | Rate | 2021 | 4006.822667 | 4539.010321 | 3581.258396 |
| Prevalence | High-income Asia Pacific | Both | Age-standardized | Depressive disorders | Rate | 1990 | 2168.623842 | 2399.341077 | 1975.10252 |
| Prevalence | High-income Asia Pacific | Both | Age-standardized | Depressive disorders | Rate | 1991 | 2133.239757 | 2354.059359 | 1944.174857 |
| Prevalence | High-income Asia Pacific | Both | Age-standardized | Depressive disorders | Rate | 1992 | 2108.070086 | 2327.418129 | 1919.497751 |
| Prevalence | High-income Asia Pacific | Both | Age-standardized | Depressive disorders | Rate | 1993 | 2091.424335 | 2311.170173 | 1901.947933 |
| Prevalence | High-income Asia Pacific | Both | Age-standardized | Depressive disorders | Rate | 1994 | 2081.836778 | 2300.509061 | 1890.98571 |
| Prevalence | High-income Asia Pacific | Both | Age-standardized | Depressive disorders | Rate | 1995 | 2077.784074 | 2295.32745 | 1883.66492 |
| Prevalence | High-income Asia Pacific | Both | Age-standardized | Depressive disorders | Rate | 1996 | 2092.593185 | 2308.770497 | 1899.594454 |
| Prevalence | High-income Asia Pacific | Both | Age-standardized | Depressive disorders | Rate | 1997 | 2131.100857 | 2350.833718 | 1935.870084 |
| Prevalence | High-income Asia Pacific | Both | Age-standardized | Depressive disorders | Rate | 1998 | 2179.437352 | 2402.672801 | 1982.566698 |
| Prevalence | High-income Asia Pacific | Both | Age-standardized | Depressive disorders | Rate | 1999 | 2223.778016 | 2447.738486 | 2025.771023 |
| Prevalence | High-income Asia Pacific | Both | Age-standardized | Depressive disorders | Rate | 2000 | 2250.596611 | 2477.387677 | 2052.232069 |
| Prevalence | High-income Asia Pacific | Both | Age-standardized | Depressive disorders | Rate | 2001 | 2263.118289 | 2488.925015 | 2067.089543 |
| Prevalence | High-income Asia Pacific | Both | Age-standardized | Depressive disorders | Rate | 2002 | 2273.388806 | 2501.091155 | 2079.646086 |
| Prevalence | High-income Asia Pacific | Both | Age-standardized | Depressive disorders | Rate | 2003 | 2281.008578 | 2507.995068 | 2086.81524 |
| Prevalence | High-income Asia Pacific | Both | Age-standardized | Depressive disorders | Rate | 2004 | 2285.807672 | 2513.530443 | 2093.950958 |
| Prevalence | High-income Asia Pacific | Both | Age-standardized | Depressive disorders | Rate | 2005 | 2287.16604 | 2515.364717 | 2094.505333 |
| Prevalence | High-income Asia Pacific | Both | Age-standardized | Depressive disorders | Rate | 2006 | 2283.154281 | 2505.758813 | 2091.488357 |
| Prevalence | High-income Asia Pacific | Both | Age-standardized | Depressive disorders | Rate | 2007 | 2274.147745 | 2498.455469 | 2082.652913 |
| Prevalence | High-income Asia Pacific | Both | Age-standardized | Depressive disorders | Rate | 2008 | 2263.074799 | 2486.167566 | 2071.298144 |
| Prevalence | High-income Asia Pacific | Both | Age-standardized | Depressive disorders | Rate | 2009 | 2253.441009 | 2472.192037 | 2060.197568 |
| Prevalence | High-income Asia Pacific | Both | Age-standardized | Depressive disorders | Rate | 2010 | 2247.909908 | 2463.680036 | 2052.118192 |
| Prevalence | High-income Asia Pacific | Both | Age-standardized | Depressive disorders | Rate | 2011 | 2243.849353 | 2463.553393 | 2048.279254 |
| Prevalence | High-income Asia Pacific | Both | Age-standardized | Depressive disorders | Rate | 2012 | 2237.67271 | 2466.076914 | 2039.734474 |
| Prevalence | High-income Asia Pacific | Both | Age-standardized | Depressive disorders | Rate | 2013 | 2231.087818 | 2463.580195 | 2033.57642 |
| Prevalence | High-income Asia Pacific | Both | Age-standardized | Depressive disorders | Rate | 2014 | 2225.742846 | 2459.845706 | 2027.094843 |
| Prevalence | High-income Asia Pacific | Both | Age-standardized | Depressive disorders | Rate | 2015 | 2222.922204 | 2456.256754 | 2022.088923 |
| Prevalence | High-income Asia Pacific | Both | Age-standardized | Depressive disorders | Rate | 2016 | 2220.518581 | 2455.992909 | 2020.796408 |
| Prevalence | High-income Asia Pacific | Both | Age-standardized | Depressive disorders | Rate | 2017 | 2217.10309 | 2451.798936 | 2019.593181 |
| Prevalence | High-income Asia Pacific | Both | Age-standardized | Depressive disorders | Rate | 2018 | 2214.571276 | 2461.721915 | 2016.496743 |
| Prevalence | High-income Asia Pacific | Both | Age-standardized | Depressive disorders | Rate | 2019 | 2215.250066 | 2479.436219 | 2011.262078 |
| Prevalence | High-income Asia Pacific | Both | Age-standardized | Depressive disorders | Rate | 2020 | 2409.733428 | 2740.176282 | 2140.105194 |
| Prevalence | High-income Asia Pacific | Both | Age-standardized | Depressive disorders | Rate | 2021 | 2545.156188 | 2892.376535 | 2266.732892 |
| Prevalence | Central Asia | Both | Age-standardized | Depressive disorders | Rate | 1990 | 3482.870577 | 3966.883659 | 3058.010783 |
| Prevalence | Central Asia | Both | Age-standardized | Depressive disorders | Rate | 1991 | 3470.020084 | 3940.287196 | 3056.980453 |
| Prevalence | Central Asia | Both | Age-standardized | Depressive disorders | Rate | 1992 | 3459.41947 | 3913.998195 | 3055.487137 |
| Prevalence | Central Asia | Both | Age-standardized | Depressive disorders | Rate | 1993 | 3451.130229 | 3901.314068 | 3050.873183 |
| Prevalence | Central Asia | Both | Age-standardized | Depressive disorders | Rate | 1994 | 3444.852064 | 3899.631236 | 3051.979895 |
| Prevalence | Central Asia | Both | Age-standardized | Depressive disorders | Rate | 1995 | 3440.499421 | 3894.09476 | 3053.764484 |
| Prevalence | Central Asia | Both | Age-standardized | Depressive disorders | Rate | 1996 | 3439.155646 | 3900.503584 | 3051.854168 |
| Prevalence | Central Asia | Both | Age-standardized | Depressive disorders | Rate | 1997 | 3440.576556 | 3894.331589 | 3055.226226 |
| Prevalence | Central Asia | Both | Age-standardized | Depressive disorders | Rate | 1998 | 3442.660097 | 3896.044627 | 3059.347397 |
| Prevalence | Central Asia | Both | Age-standardized | Depressive disorders | Rate | 1999 | 3443.629519 | 3894.10209 | 3056.706953 |
| Prevalence | Central Asia | Both | Age-standardized | Depressive disorders | Rate | 2000 | 3441.784867 | 3887.072074 | 3055.230323 |
| Prevalence | Central Asia | Both | Age-standardized | Depressive disorders | Rate | 2001 | 3437.637736 | 3885.3579 | 3050.244439 |
| Prevalence | Central Asia | Both | Age-standardized | Depressive disorders | Rate | 2002 | 3433.1114 | 3885.281165 | 3046.152218 |
| Prevalence | Central Asia | Both | Age-standardized | Depressive disorders | Rate | 2003 | 3427.92819 | 3879.99815 | 3033.65171 |
| Prevalence | Central Asia | Both | Age-standardized | Depressive disorders | Rate | 2004 | 3421.895258 | 3867.643738 | 3026.157682 |
| Prevalence | Central Asia | Both | Age-standardized | Depressive disorders | Rate | 2005 | 3414.763607 | 3855.588528 | 3026.032077 |
| Prevalence | Central Asia | Both | Age-standardized | Depressive disorders | Rate | 2006 | 3403.534611 | 3840.152663 | 3018.540227 |
| Prevalence | Central Asia | Both | Age-standardized | Depressive disorders | Rate | 2007 | 3387.96677 | 3822.403005 | 3001.359172 |
| Prevalence | Central Asia | Both | Age-standardized | Depressive disorders | Rate | 2008 | 3371.201363 | 3808.992828 | 2990.628969 |
| Prevalence | Central Asia | Both | Age-standardized | Depressive disorders | Rate | 2009 | 3356.481275 | 3795.546354 | 2985.090175 |
| Prevalence | Central Asia | Both | Age-standardized | Depressive disorders | Rate | 2010 | 3347.126497 | 3782.231701 | 2975.105013 |
| Prevalence | Central Asia | Both | Age-standardized | Depressive disorders | Rate | 2011 | 3344.025375 | 3784.322031 | 2976.583615 |
| Prevalence | Central Asia | Both | Age-standardized | Depressive disorders | Rate | 2012 | 3344.794713 | 3791.090846 | 2966.933987 |
| Prevalence | Central Asia | Both | Age-standardized | Depressive disorders | Rate | 2013 | 3347.546951 | 3794.7782 | 2962.980126 |
| Prevalence | Central Asia | Both | Age-standardized | Depressive disorders | Rate | 2014 | 3350.519382 | 3792.295088 | 2959.87942 |
| Prevalence | Central Asia | Both | Age-standardized | Depressive disorders | Rate | 2015 | 3352.072521 | 3803.016112 | 2958.627007 |
| Prevalence | Central Asia | Both | Age-standardized | Depressive disorders | Rate | 2016 | 3353.663249 | 3811.934801 | 2953.570656 |
| Prevalence | Central Asia | Both | Age-standardized | Depressive disorders | Rate | 2017 | 3357.096453 | 3821.960889 | 2952.950238 |
| Prevalence | Central Asia | Both | Age-standardized | Depressive disorders | Rate | 2018 | 3360.464925 | 3840.951207 | 2952.46646 |
| Prevalence | Central Asia | Both | Age-standardized | Depressive disorders | Rate | 2019 | 3361.954994 | 3850.8346 | 2945.574263 |
| Prevalence | Central Asia | Both | Age-standardized | Depressive disorders | Rate | 2020 | 3684.481743 | 4276.215762 | 3165.131682 |
| Prevalence | Central Asia | Both | Age-standardized | Depressive disorders | Rate | 2021 | 3773.706559 | 4386.790191 | 3243.382033 |
| Prevalence | Southeast Asia | Both | Age-standardized | Depressive disorders | Rate | 1990 | 2742.545807 | 3111.359537 | 2435.886605 |
| Prevalence | Southeast Asia | Both | Age-standardized | Depressive disorders | Rate | 1991 | 2735.716736 | 3101.255149 | 2427.092105 |
| Prevalence | Southeast Asia | Both | Age-standardized | Depressive disorders | Rate | 1992 | 2728.662833 | 3092.709119 | 2420.187417 |
| Prevalence | Southeast Asia | Both | Age-standardized | Depressive disorders | Rate | 1993 | 2721.783227 | 3084.175539 | 2412.645991 |
| Prevalence | Southeast Asia | Both | Age-standardized | Depressive disorders | Rate | 1994 | 2715.415979 | 3076.778245 | 2405.249635 |
| Prevalence | Southeast Asia | Both | Age-standardized | Depressive disorders | Rate | 1995 | 2709.907025 | 3069.675753 | 2398.279927 |
| Prevalence | Southeast Asia | Both | Age-standardized | Depressive disorders | Rate | 1996 | 2702.379419 | 3059.102433 | 2393.260315 |
| Prevalence | Southeast Asia | Both | Age-standardized | Depressive disorders | Rate | 1997 | 2691.279362 | 3041.682204 | 2386.075071 |
| Prevalence | Southeast Asia | Both | Age-standardized | Depressive disorders | Rate | 1998 | 2679.096852 | 3022.982292 | 2377.381794 |
| Prevalence | Southeast Asia | Both | Age-standardized | Depressive disorders | Rate | 1999 | 2668.250281 | 3006.377428 | 2367.582778 |
| Prevalence | Southeast Asia | Both | Age-standardized | Depressive disorders | Rate | 2000 | 2661.292275 | 2996.856784 | 2360.922033 |
| Prevalence | Southeast Asia | Both | Age-standardized | Depressive disorders | Rate | 2001 | 2658.969519 | 2996.262467 | 2357.812435 |
| Prevalence | Southeast Asia | Both | Age-standardized | Depressive disorders | Rate | 2002 | 2659.290766 | 2998.591842 | 2356.872255 |
| Prevalence | Southeast Asia | Both | Age-standardized | Depressive disorders | Rate | 2003 | 2660.772115 | 3002.206171 | 2356.971653 |
| Prevalence | Southeast Asia | Both | Age-standardized | Depressive disorders | Rate | 2004 | 2661.798906 | 3004.768379 | 2358.892137 |
| Prevalence | Southeast Asia | Both | Age-standardized | Depressive disorders | Rate | 2005 | 2660.665633 | 3005.525007 | 2359.787664 |
| Prevalence | Southeast Asia | Both | Age-standardized | Depressive disorders | Rate | 2006 | 2655.752737 | 3000.182055 | 2354.837879 |
| Prevalence | Southeast Asia | Both | Age-standardized | Depressive disorders | Rate | 2007 | 2647.956766 | 2991.640192 | 2347.379244 |
| Prevalence | Southeast Asia | Both | Age-standardized | Depressive disorders | Rate | 2008 | 2639.763331 | 2981.237012 | 2340.677956 |
| Prevalence | Southeast Asia | Both | Age-standardized | Depressive disorders | Rate | 2009 | 2633.132724 | 2971.241825 | 2336.776728 |
| Prevalence | Southeast Asia | Both | Age-standardized | Depressive disorders | Rate | 2010 | 2629.364353 | 2964.45927 | 2335.347967 |
| Prevalence | Southeast Asia | Both | Age-standardized | Depressive disorders | Rate | 2011 | 2629.771529 | 2965.267563 | 2336.16345 |
| Prevalence | Southeast Asia | Both | Age-standardized | Depressive disorders | Rate | 2012 | 2633.861563 | 2969.823163 | 2340.199283 |
| Prevalence | Southeast Asia | Both | Age-standardized | Depressive disorders | Rate | 2013 | 2640.071377 | 2977.792519 | 2345.394679 |
| Prevalence | Southeast Asia | Both | Age-standardized | Depressive disorders | Rate | 2014 | 2646.624128 | 2987.886233 | 2349.594336 |
| Prevalence | Southeast Asia | Both | Age-standardized | Depressive disorders | Rate | 2015 | 2651.956187 | 2995.012919 | 2352.710622 |
| Prevalence | Southeast Asia | Both | Age-standardized | Depressive disorders | Rate | 2016 | 2658.305606 | 3004.746729 | 2360.024602 |
| Prevalence | Southeast Asia | Both | Age-standardized | Depressive disorders | Rate | 2017 | 2667.010037 | 3018.963884 | 2370.560849 |
| Prevalence | Southeast Asia | Both | Age-standardized | Depressive disorders | Rate | 2018 | 2674.938503 | 3033.255967 | 2377.222427 |
| Prevalence | Southeast Asia | Both | Age-standardized | Depressive disorders | Rate | 2019 | 2678.900454 | 3044.95176 | 2377.692649 |
| Prevalence | Southeast Asia | Both | Age-standardized | Depressive disorders | Rate | 2020 | 2837.561526 | 3208.85019 | 2511.167716 |
| Prevalence | Southeast Asia | Both | Age-standardized | Depressive disorders | Rate | 2021 | 2991.563797 | 3401.761331 | 2647.86478 |
| Prevalence | Australasia | Both | Age-standardized | Depressive disorders | Rate | 1990 | 4407.642283 | 4998.582607 | 3912.807879 |
| Prevalence | Australasia | Both | Age-standardized | Depressive disorders | Rate | 1991 | 4432.442911 | 5005.142424 | 3954.618373 |
| Prevalence | Australasia | Both | Age-standardized | Depressive disorders | Rate | 1992 | 4454.729636 | 5017.38184 | 3996.853958 |
| Prevalence | Australasia | Both | Age-standardized | Depressive disorders | Rate | 1993 | 4474.532148 | 5013.766566 | 4020.28524 |
| Prevalence | Australasia | Both | Age-standardized | Depressive disorders | Rate | 1994 | 4491.847522 | 5022.477146 | 4035.254966 |
| Prevalence | Australasia | Both | Age-standardized | Depressive disorders | Rate | 1995 | 4506.791011 | 5033.992941 | 4055.233528 |
| Prevalence | Australasia | Both | Age-standardized | Depressive disorders | Rate | 1996 | 4513.711483 | 5002.871942 | 4087.061619 |
| Prevalence | Australasia | Both | Age-standardized | Depressive disorders | Rate | 1997 | 4511.895338 | 4986.527957 | 4119.23129 |
| Prevalence | Australasia | Both | Age-standardized | Depressive disorders | Rate | 1998 | 4509.127054 | 4983.588953 | 4137.191056 |
| Prevalence | Australasia | Both | Age-standardized | Depressive disorders | Rate | 1999 | 4512.960898 | 4977.22114 | 4148.196837 |
| Prevalence | Australasia | Both | Age-standardized | Depressive disorders | Rate | 2000 | 4530.190364 | 5001.727455 | 4168.354689 |
| Prevalence | Australasia | Both | Age-standardized | Depressive disorders | Rate | 2001 | 4574.337462 | 5051.63569 | 4215.658662 |
| Prevalence | Australasia | Both | Age-standardized | Depressive disorders | Rate | 2002 | 4640.866049 | 5136.023916 | 4264.977464 |
| Prevalence | Australasia | Both | Age-standardized | Depressive disorders | Rate | 2003 | 4711.007296 | 5205.615717 | 4324.848504 |
| Prevalence | Australasia | Both | Age-standardized | Depressive disorders | Rate | 2004 | 4768.242975 | 5301.149795 | 4351.007854 |
| Prevalence | Australasia | Both | Age-standardized | Depressive disorders | Rate | 2005 | 4794.703581 | 5350.20154 | 4349.674918 |
| Prevalence | Australasia | Both | Age-standardized | Depressive disorders | Rate | 2006 | 4788.648255 | 5337.495469 | 4362.750136 |
| Prevalence | Australasia | Both | Age-standardized | Depressive disorders | Rate | 2007 | 4765.976396 | 5308.951636 | 4335.381393 |
| Prevalence | Australasia | Both | Age-standardized | Depressive disorders | Rate | 2008 | 4734.332093 | 5273.175367 | 4307.264191 |
| Prevalence | Australasia | Both | Age-standardized | Depressive disorders | Rate | 2009 | 4701.172651 | 5221.393527 | 4280.793575 |
| Prevalence | Australasia | Both | Age-standardized | Depressive disorders | Rate | 2010 | 4674.154566 | 5198.426793 | 4254.814753 |
| Prevalence | Australasia | Both | Age-standardized | Depressive disorders | Rate | 2011 | 4652.630709 | 5171.686615 | 4224.735427 |
| Prevalence | Australasia | Both | Age-standardized | Depressive disorders | Rate | 2012 | 4631.035637 | 5167.448895 | 4187.003494 |
| Prevalence | Australasia | Both | Age-standardized | Depressive disorders | Rate | 2013 | 4608.483609 | 5170.508415 | 4145.635333 |
| Prevalence | Australasia | Both | Age-standardized | Depressive disorders | Rate | 2014 | 4584.460609 | 5185.053001 | 4099.534451 |
| Prevalence | Australasia | Both | Age-standardized | Depressive disorders | Rate | 2015 | 4559.121798 | 5179.146263 | 4057.159882 |
| Prevalence | Australasia | Both | Age-standardized | Depressive disorders | Rate | 2016 | 4522.023071 | 5148.561076 | 4015.746162 |
| Prevalence | Australasia | Both | Age-standardized | Depressive disorders | Rate | 2017 | 4473.322989 | 5094.229102 | 3956.925974 |
| Prevalence | Australasia | Both | Age-standardized | Depressive disorders | Rate | 2018 | 4429.29057 | 5071.616345 | 3908.241397 |
| Prevalence | Australasia | Both | Age-standardized | Depressive disorders | Rate | 2019 | 4405.952408 | 5058.428892 | 3883.872454 |
| Prevalence | Australasia | Both | Age-standardized | Depressive disorders | Rate | 2020 | 4718.87236 | 5652.971728 | 3913.838967 |
| Prevalence | Australasia | Both | Age-standardized | Depressive disorders | Rate | 2021 | 4691.879978 | 5719.886801 | 3904.487911 |
| Prevalence | Central Europe | Both | Age-standardized | Depressive disorders | Rate | 1990 | 2946.109018 | 3340.061253 | 2624.378237 |
| Prevalence | Central Europe | Both | Age-standardized | Depressive disorders | Rate | 1991 | 2943.060429 | 3331.512277 | 2620.418349 |
| Prevalence | Central Europe | Both | Age-standardized | Depressive disorders | Rate | 1992 | 2939.160842 | 3325.043756 | 2622.118986 |
| Prevalence | Central Europe | Both | Age-standardized | Depressive disorders | Rate | 1993 | 2934.448415 | 3314.618912 | 2625.094841 |
| Prevalence | Central Europe | Both | Age-standardized | Depressive disorders | Rate | 1994 | 2928.743671 | 3302.922942 | 2625.928031 |
| Prevalence | Central Europe | Both | Age-standardized | Depressive disorders | Rate | 1995 | 2923.050279 | 3293.426032 | 2623.847828 |
| Prevalence | Central Europe | Both | Age-standardized | Depressive disorders | Rate | 1996 | 2915.492457 | 3285.50798 | 2614.700568 |
| Prevalence | Central Europe | Both | Age-standardized | Depressive disorders | Rate | 1997 | 2904.410529 | 3273.977009 | 2606.897862 |
| Prevalence | Central Europe | Both | Age-standardized | Depressive disorders | Rate | 1998 | 2891.85601 | 3261.337361 | 2593.358812 |
| Prevalence | Central Europe | Both | Age-standardized | Depressive disorders | Rate | 1999 | 2879.077303 | 3247.451217 | 2579.706131 |
| Prevalence | Central Europe | Both | Age-standardized | Depressive disorders | Rate | 2000 | 2867.172241 | 3235.244442 | 2567.349838 |
| Prevalence | Central Europe | Both | Age-standardized | Depressive disorders | Rate | 2001 | 2849.671943 | 3214.937688 | 2552.026176 |
| Prevalence | Central Europe | Both | Age-standardized | Depressive disorders | Rate | 2002 | 2822.84864 | 3185.368701 | 2525.06597 |
| Prevalence | Central Europe | Both | Age-standardized | Depressive disorders | Rate | 2003 | 2792.46861 | 3152.617099 | 2493.661286 |
| Prevalence | Central Europe | Both | Age-standardized | Depressive disorders | Rate | 2004 | 2764.251773 | 3120.458162 | 2466.718196 |
| Prevalence | Central Europe | Both | Age-standardized | Depressive disorders | Rate | 2005 | 2743.975104 | 3096.481311 | 2449.348226 |
| Prevalence | Central Europe | Both | Age-standardized | Depressive disorders | Rate | 2006 | 2727.662521 | 3076.81191 | 2435.431651 |
| Prevalence | Central Europe | Both | Age-standardized | Depressive disorders | Rate | 2007 | 2709.42575 | 3055.452459 | 2422.267541 |
| Prevalence | Central Europe | Both | Age-standardized | Depressive disorders | Rate | 2008 | 2691.936548 | 3035.136702 | 2407.652809 |
| Prevalence | Central Europe | Both | Age-standardized | Depressive disorders | Rate | 2009 | 2677.32647 | 3018.932774 | 2395.380139 |
| Prevalence | Central Europe | Both | Age-standardized | Depressive disorders | Rate | 2010 | 2667.832146 | 3012.649423 | 2387.541367 |
| Prevalence | Central Europe | Both | Age-standardized | Depressive disorders | Rate | 2011 | 2662.094344 | 3005.990653 | 2380.874423 |
| Prevalence | Central Europe | Both | Age-standardized | Depressive disorders | Rate | 2012 | 2657.369051 | 3007.337785 | 2372.574386 |
| Prevalence | Central Europe | Both | Age-standardized | Depressive disorders | Rate | 2013 | 2653.839388 | 3006.798387 | 2369.479522 |
| Prevalence | Central Europe | Both | Age-standardized | Depressive disorders | Rate | 2014 | 2651.383893 | 3001.574712 | 2367.27258 |
| Prevalence | Central Europe | Both | Age-standardized | Depressive disorders | Rate | 2015 | 2650.040611 | 3000.339084 | 2366.743187 |
| Prevalence | Central Europe | Both | Age-standardized | Depressive disorders | Rate | 2016 | 2658.309053 | 3009.102029 | 2370.362576 |
| Prevalence | Central Europe | Both | Age-standardized | Depressive disorders | Rate | 2017 | 2677.814068 | 3028.990674 | 2382.921842 |
| Prevalence | Central Europe | Both | Age-standardized | Depressive disorders | Rate | 2018 | 2698.391743 | 3052.870044 | 2395.806569 |
| Prevalence | Central Europe | Both | Age-standardized | Depressive disorders | Rate | 2019 | 2709.67465 | 3070.910875 | 2401.27541 |
| Prevalence | Central Europe | Both | Age-standardized | Depressive disorders | Rate | 2020 | 3009.387404 | 3424.988061 | 2652.847396 |
| Prevalence | Central Europe | Both | Age-standardized | Depressive disorders | Rate | 2021 | 3171.900109 | 3611.516498 | 2789.030359 |
| Prevalence | Eastern Europe | Both | Age-standardized | Depressive disorders | Rate | 1990 | 3774.062032 | 4255.227649 | 3369.07731 |
| Prevalence | Eastern Europe | Both | Age-standardized | Depressive disorders | Rate | 1991 | 3798.292676 | 4280.595963 | 3392.151953 |
| Prevalence | Eastern Europe | Both | Age-standardized | Depressive disorders | Rate | 1992 | 3818.026697 | 4300.570493 | 3415.43641 |
| Prevalence | Eastern Europe | Both | Age-standardized | Depressive disorders | Rate | 1993 | 3833.719444 | 4317.511587 | 3434.180522 |
| Prevalence | Eastern Europe | Both | Age-standardized | Depressive disorders | Rate | 1994 | 3846.224125 | 4330.86073 | 3449.748535 |
| Prevalence | Eastern Europe | Both | Age-standardized | Depressive disorders | Rate | 1995 | 3853.857481 | 4336.727659 | 3458.548959 |
| Prevalence | Eastern Europe | Both | Age-standardized | Depressive disorders | Rate | 1996 | 3856.200122 | 4338.54325 | 3457.243222 |
| Prevalence | Eastern Europe | Both | Age-standardized | Depressive disorders | Rate | 1997 | 3853.07905 | 4334.667417 | 3451.850009 |
| Prevalence | Eastern Europe | Both | Age-standardized | Depressive disorders | Rate | 1998 | 3846.977636 | 4329.776982 | 3442.782456 |
| Prevalence | Eastern Europe | Both | Age-standardized | Depressive disorders | Rate | 1999 | 3839.914359 | 4325.999241 | 3432.892652 |
| Prevalence | Eastern Europe | Both | Age-standardized | Depressive disorders | Rate | 2000 | 3832.346834 | 4319.034456 | 3424.26566 |
| Prevalence | Eastern Europe | Both | Age-standardized | Depressive disorders | Rate | 2001 | 3818.062649 | 4304.638216 | 3412.321473 |
| Prevalence | Eastern Europe | Both | Age-standardized | Depressive disorders | Rate | 2002 | 3792.189953 | 4279.303871 | 3390.42952 |
| Prevalence | Eastern Europe | Both | Age-standardized | Depressive disorders | Rate | 2003 | 3760.704709 | 4245.011002 | 3363.725287 |
| Prevalence | Eastern Europe | Both | Age-standardized | Depressive disorders | Rate | 2004 | 3728.222292 | 4208.967811 | 3335.723492 |
| Prevalence | Eastern Europe | Both | Age-standardized | Depressive disorders | Rate | 2005 | 3698.607651 | 4175.47498 | 3309.207633 |
| Prevalence | Eastern Europe | Both | Age-standardized | Depressive disorders | Rate | 2006 | 3665.935889 | 4137.476551 | 3282.455733 |
| Prevalence | Eastern Europe | Both | Age-standardized | Depressive disorders | Rate | 2007 | 3625.115246 | 4086.160323 | 3251.545122 |
| Prevalence | Eastern Europe | Both | Age-standardized | Depressive disorders | Rate | 2008 | 3584.384947 | 4038.73356 | 3220.327639 |
| Prevalence | Eastern Europe | Both | Age-standardized | Depressive disorders | Rate | 2009 | 3550.52497 | 4001.005317 | 3192.664468 |
| Prevalence | Eastern Europe | Both | Age-standardized | Depressive disorders | Rate | 2010 | 3529.581149 | 3981.102499 | 3175.0388 |
| Prevalence | Eastern Europe | Both | Age-standardized | Depressive disorders | Rate | 2011 | 3522.984699 | 3974.954375 | 3164.801328 |
| Prevalence | Eastern Europe | Both | Age-standardized | Depressive disorders | Rate | 2012 | 3524.602465 | 3978.704252 | 3161.577536 |
| Prevalence | Eastern Europe | Both | Age-standardized | Depressive disorders | Rate | 2013 | 3530.941279 | 3986.405756 | 3163.32589 |
| Prevalence | Eastern Europe | Both | Age-standardized | Depressive disorders | Rate | 2014 | 3537.560009 | 3997.619128 | 3164.164851 |
| Prevalence | Eastern Europe | Both | Age-standardized | Depressive disorders | Rate | 2015 | 3539.285395 | 4003.322149 | 3161.146718 |
| Prevalence | Eastern Europe | Both | Age-standardized | Depressive disorders | Rate | 2016 | 3535.206025 | 3994.34762 | 3157.715361 |
| Prevalence | Eastern Europe | Both | Age-standardized | Depressive disorders | Rate | 2017 | 3528.300245 | 3983.689218 | 3151.88001 |
| Prevalence | Eastern Europe | Both | Age-standardized | Depressive disorders | Rate | 2018 | 3520.666449 | 3978.349554 | 3144.338272 |
| Prevalence | Eastern Europe | Both | Age-standardized | Depressive disorders | Rate | 2019 | 3513.738895 | 3975.346848 | 3139.076799 |
| Prevalence | Eastern Europe | Both | Age-standardized | Depressive disorders | Rate | 2020 | 3880.533103 | 4365.239896 | 3425.218235 |
| Prevalence | Eastern Europe | Both | Age-standardized | Depressive disorders | Rate | 2021 | 4231.790631 | 4771.111187 | 3729.903861 |
| Prevalence | High-income North America | Both | Age-standardized | Depressive disorders | Rate | 1990 | 3817.104882 | 4244.845778 | 3439.49407 |
| Prevalence | High-income North America | Both | Age-standardized | Depressive disorders | Rate | 1991 | 3924.17917 | 4353.573743 | 3541.113968 |
| Prevalence | High-income North America | Both | Age-standardized | Depressive disorders | Rate | 1992 | 4030.487413 | 4464.237814 | 3641.345172 |
| Prevalence | High-income North America | Both | Age-standardized | Depressive disorders | Rate | 1993 | 4132.336407 | 4571.396516 | 3735.30182 |
| Prevalence | High-income North America | Both | Age-standardized | Depressive disorders | Rate | 1994 | 4226.093267 | 4669.039896 | 3826.754936 |
| Prevalence | High-income North America | Both | Age-standardized | Depressive disorders | Rate | 1995 | 4307.856856 | 4752.811924 | 3896.463186 |
| Prevalence | High-income North America | Both | Age-standardized | Depressive disorders | Rate | 1996 | 4377.778466 | 4826.932312 | 3959.421075 |
| Prevalence | High-income North America | Both | Age-standardized | Depressive disorders | Rate | 1997 | 4437.175545 | 4894.058432 | 4010.592383 |
| Prevalence | High-income North America | Both | Age-standardized | Depressive disorders | Rate | 1998 | 4484.285784 | 4952.518433 | 4049.759128 |
| Prevalence | High-income North America | Both | Age-standardized | Depressive disorders | Rate | 1999 | 4517.135685 | 4991.854698 | 4088.308531 |
| Prevalence | High-income North America | Both | Age-standardized | Depressive disorders | Rate | 2000 | 4533.423506 | 5022.850492 | 4102.804729 |
| Prevalence | High-income North America | Both | Age-standardized | Depressive disorders | Rate | 2001 | 4529.718009 | 5014.812107 | 4099.895853 |
| Prevalence | High-income North America | Both | Age-standardized | Depressive disorders | Rate | 2002 | 4510.221113 | 4990.174979 | 4088.839805 |
| Prevalence | High-income North America | Both | Age-standardized | Depressive disorders | Rate | 2003 | 4484.524577 | 4956.57332 | 4070.031013 |
| Prevalence | High-income North America | Both | Age-standardized | Depressive disorders | Rate | 2004 | 4462.251158 | 4926.67666 | 4049.010911 |
| Prevalence | High-income North America | Both | Age-standardized | Depressive disorders | Rate | 2005 | 4452.959454 | 4923.405117 | 4039.799775 |
| Prevalence | High-income North America | Both | Age-standardized | Depressive disorders | Rate | 2006 | 4450.867696 | 4917.9874 | 4042.417452 |
| Prevalence | High-income North America | Both | Age-standardized | Depressive disorders | Rate | 2007 | 4445.106658 | 4923.515144 | 4039.248555 |
| Prevalence | High-income North America | Both | Age-standardized | Depressive disorders | Rate | 2008 | 4437.991108 | 4919.186659 | 4032.041128 |
| Prevalence | High-income North America | Both | Age-standardized | Depressive disorders | Rate | 2009 | 4431.749053 | 4912.652658 | 4023.812019 |
| Prevalence | High-income North America | Both | Age-standardized | Depressive disorders | Rate | 2010 | 4428.415265 | 4917.039437 | 4022.383663 |
| Prevalence | High-income North America | Both | Age-standardized | Depressive disorders | Rate | 2011 | 4425.461017 | 4900.705189 | 4023.788436 |
| Prevalence | High-income North America | Both | Age-standardized | Depressive disorders | Rate | 2012 | 4419.782784 | 4881.201415 | 4024.507448 |
| Prevalence | High-income North America | Both | Age-standardized | Depressive disorders | Rate | 2013 | 4412.536907 | 4861.47372 | 4011.976026 |
| Prevalence | High-income North America | Both | Age-standardized | Depressive disorders | Rate | 2014 | 4404.49359 | 4854.805284 | 4006.438766 |
| Prevalence | High-income North America | Both | Age-standardized | Depressive disorders | Rate | 2015 | 4396.325872 | 4840.74025 | 4005.176674 |
| Prevalence | High-income North America | Both | Age-standardized | Depressive disorders | Rate | 2016 | 4389.020988 | 4836.833036 | 4011.427789 |
| Prevalence | High-income North America | Both | Age-standardized | Depressive disorders | Rate | 2017 | 4384.552541 | 4844.102146 | 4007.494567 |
| Prevalence | High-income North America | Both | Age-standardized | Depressive disorders | Rate | 2018 | 4385.234826 | 4857.898475 | 3996.923463 |
| Prevalence | High-income North America | Both | Age-standardized | Depressive disorders | Rate | 2019 | 4393.292391 | 4894.949345 | 3986.617271 |
| Prevalence | High-income North America | Both | Age-standardized | Depressive disorders | Rate | 2020 | 5400.137166 | 6010.853217 | 4839.05131 |
| Prevalence | High-income North America | Both | Age-standardized | Depressive disorders | Rate | 2021 | 5408.261749 | 6049.723387 | 4846.89577 |
| Prevalence | Tropical Latin America | Both | Age-standardized | Depressive disorders | Rate | 1990 | 3931.871363 | 4398.763588 | 3542.556891 |
| Prevalence | Tropical Latin America | Both | Age-standardized | Depressive disorders | Rate | 1991 | 3916.345005 | 4372.105911 | 3532.17598 |
| Prevalence | Tropical Latin America | Both | Age-standardized | Depressive disorders | Rate | 1992 | 3911.725628 | 4365.735893 | 3534.602556 |
| Prevalence | Tropical Latin America | Both | Age-standardized | Depressive disorders | Rate | 1993 | 3915.847678 | 4371.369581 | 3539.743562 |
| Prevalence | Tropical Latin America | Both | Age-standardized | Depressive disorders | Rate | 1994 | 3926.546784 | 4376.207717 | 3550.840694 |
| Prevalence | Tropical Latin America | Both | Age-standardized | Depressive disorders | Rate | 1995 | 3941.633983 | 4387.050438 | 3564.42231 |
| Prevalence | Tropical Latin America | Both | Age-standardized | Depressive disorders | Rate | 1996 | 4004.366467 | 4457.427814 | 3624.185662 |
| Prevalence | Tropical Latin America | Both | Age-standardized | Depressive disorders | Rate | 1997 | 4130.776379 | 4597.683185 | 3743.517185 |
| Prevalence | Tropical Latin America | Both | Age-standardized | Depressive disorders | Rate | 1998 | 4278.514508 | 4761.545472 | 3889.13322 |
| Prevalence | Tropical Latin America | Both | Age-standardized | Depressive disorders | Rate | 1999 | 4405.161676 | 4904.416077 | 4002.616534 |
| Prevalence | Tropical Latin America | Both | Age-standardized | Depressive disorders | Rate | 2000 | 4468.396134 | 4974.818628 | 4059.533208 |
| Prevalence | Tropical Latin America | Both | Age-standardized | Depressive disorders | Rate | 2001 | 4484.407107 | 4989.646706 | 4071.802662 |
| Prevalence | Tropical Latin America | Both | Age-standardized | Depressive disorders | Rate | 2002 | 4496.429505 | 5004.901487 | 4078.865279 |
| Prevalence | Tropical Latin America | Both | Age-standardized | Depressive disorders | Rate | 2003 | 4503.123893 | 5013.920458 | 4084.487518 |
| Prevalence | Tropical Latin America | Both | Age-standardized | Depressive disorders | Rate | 2004 | 4503.133872 | 5019.16846 | 4084.382449 |
| Prevalence | Tropical Latin America | Both | Age-standardized | Depressive disorders | Rate | 2005 | 4495.052124 | 5013.519401 | 4072.858306 |
| Prevalence | Tropical Latin America | Both | Age-standardized | Depressive disorders | Rate | 2006 | 4423.163159 | 4919.778519 | 4025.317911 |
| Prevalence | Tropical Latin America | Both | Age-standardized | Depressive disorders | Rate | 2007 | 4266.98056 | 4724.611358 | 3909.283766 |
| Prevalence | Tropical Latin America | Both | Age-standardized | Depressive disorders | Rate | 2008 | 4078.092541 | 4505.469395 | 3747.440843 |
| Prevalence | Tropical Latin America | Both | Age-standardized | Depressive disorders | Rate | 2009 | 3908.109326 | 4290.064302 | 3601.105277 |
| Prevalence | Tropical Latin America | Both | Age-standardized | Depressive disorders | Rate | 2010 | 3808.641236 | 4171.001206 | 3513.044838 |
| Prevalence | Tropical Latin America | Both | Age-standardized | Depressive disorders | Rate | 2011 | 3763.672705 | 4125.13275 | 3464.830206 |
| Prevalence | Tropical Latin America | Both | Age-standardized | Depressive disorders | Rate | 2012 | 3723.5069 | 4083.445807 | 3424.548827 |
| Prevalence | Tropical Latin America | Both | Age-standardized | Depressive disorders | Rate | 2013 | 3689.2474 | 4052.297738 | 3392.343037 |
| Prevalence | Tropical Latin America | Both | Age-standardized | Depressive disorders | Rate | 2014 | 3661.90524 | 4025.129301 | 3363.784954 |
| Prevalence | Tropical Latin America | Both | Age-standardized | Depressive disorders | Rate | 2015 | 3642.482653 | 4005.846085 | 3343.384343 |
| Prevalence | Tropical Latin America | Both | Age-standardized | Depressive disorders | Rate | 2016 | 3626.562347 | 3993.355371 | 3326.440947 |
| Prevalence | Tropical Latin America | Both | Age-standardized | Depressive disorders | Rate | 2017 | 3610.961798 | 3983.808713 | 3311.763733 |
| Prevalence | Tropical Latin America | Both | Age-standardized | Depressive disorders | Rate | 2018 | 3598.538834 | 3973.968802 | 3290.830113 |
| Prevalence | Tropical Latin America | Both | Age-standardized | Depressive disorders | Rate | 2019 | 3592.098676 | 3963.186982 | 3274.302075 |
| Prevalence | Tropical Latin America | Both | Age-standardized | Depressive disorders | Rate | 2020 | 4144.581275 | 4664.698874 | 3703.116775 |
| Prevalence | Tropical Latin America | Both | Age-standardized | Depressive disorders | Rate | 2021 | 4352.08785 | 4948.916093 | 3871.002837 |
| Prevalence | Southern Latin America | Both | Age-standardized | Depressive disorders | Rate | 1990 | 3231.93011 | 3675.035896 | 2879.158951 |
| Prevalence | Southern Latin America | Both | Age-standardized | Depressive disorders | Rate | 1991 | 3229.713317 | 3663.802041 | 2887.665587 |
| Prevalence | Southern Latin America | Both | Age-standardized | Depressive disorders | Rate | 1992 | 3227.334819 | 3660.923595 | 2895.193626 |
| Prevalence | Southern Latin America | Both | Age-standardized | Depressive disorders | Rate | 1993 | 3224.921604 | 3652.871111 | 2908.661414 |
| Prevalence | Southern Latin America | Both | Age-standardized | Depressive disorders | Rate | 1994 | 3222.479718 | 3632.173767 | 2902.370621 |
| Prevalence | Southern Latin America | Both | Age-standardized | Depressive disorders | Rate | 1995 | 3220.41694 | 3646.589404 | 2899.103734 |
| Prevalence | Southern Latin America | Both | Age-standardized | Depressive disorders | Rate | 1996 | 3217.179173 | 3633.736337 | 2898.926659 |
| Prevalence | Southern Latin America | Both | Age-standardized | Depressive disorders | Rate | 1997 | 3211.958221 | 3626.333967 | 2900.459331 |
| Prevalence | Southern Latin America | Both | Age-standardized | Depressive disorders | Rate | 1998 | 3206.463602 | 3630.731367 | 2903.099162 |
| Prevalence | Southern Latin America | Both | Age-standardized | Depressive disorders | Rate | 1999 | 3202.612726 | 3623.842537 | 2902.121274 |
| Prevalence | Southern Latin America | Both | Age-standardized | Depressive disorders | Rate | 2000 | 3202.369715 | 3620.899291 | 2896.810219 |
| Prevalence | Southern Latin America | Both | Age-standardized | Depressive disorders | Rate | 2001 | 3206.165725 | 3628.398563 | 2894.916231 |
| Prevalence | Southern Latin America | Both | Age-standardized | Depressive disorders | Rate | 2002 | 3211.763409 | 3621.068416 | 2889.118946 |
| Prevalence | Southern Latin America | Both | Age-standardized | Depressive disorders | Rate | 2003 | 3216.906662 | 3622.352127 | 2888.667191 |
| Prevalence | Southern Latin America | Both | Age-standardized | Depressive disorders | Rate | 2004 | 3219.291884 | 3638.372026 | 2887.169496 |
| Prevalence | Southern Latin America | Both | Age-standardized | Depressive disorders | Rate | 2005 | 3216.617896 | 3643.879946 | 2875.211182 |
| Prevalence | Southern Latin America | Both | Age-standardized | Depressive disorders | Rate | 2006 | 3188.073925 | 3597.034972 | 2856.979372 |
| Prevalence | Southern Latin America | Both | Age-standardized | Depressive disorders | Rate | 2007 | 3128.628537 | 3518.362039 | 2816.701589 |
| Prevalence | Southern Latin America | Both | Age-standardized | Depressive disorders | Rate | 2008 | 3059.179114 | 3436.087924 | 2747.408673 |
| Prevalence | Southern Latin America | Both | Age-standardized | Depressive disorders | Rate | 2009 | 3000.809527 | 3371.813985 | 2696.825872 |
| Prevalence | Southern Latin America | Both | Age-standardized | Depressive disorders | Rate | 2010 | 2974.630424 | 3344.257631 | 2683.244988 |
| Prevalence | Southern Latin America | Both | Age-standardized | Depressive disorders | Rate | 2011 | 2974.886008 | 3336.781047 | 2682.623305 |
| Prevalence | Southern Latin America | Both | Age-standardized | Depressive disorders | Rate | 2012 | 2979.687953 | 3346.182206 | 2681.472584 |
| Prevalence | Southern Latin America | Both | Age-standardized | Depressive disorders | Rate | 2013 | 2986.045567 | 3351.440839 | 2688.422622 |
| Prevalence | Southern Latin America | Both | Age-standardized | Depressive disorders | Rate | 2014 | 2991.072873 | 3360.829767 | 2692.43847 |
| Prevalence | Southern Latin America | Both | Age-standardized | Depressive disorders | Rate | 2015 | 2992.011538 | 3368.831857 | 2684.316232 |
| Prevalence | Southern Latin America | Both | Age-standardized | Depressive disorders | Rate | 2016 | 2986.826471 | 3372.643333 | 2671.410103 |
| Prevalence | Southern Latin America | Both | Age-standardized | Depressive disorders | Rate | 2017 | 2977.605639 | 3366.665078 | 2658.959067 |
| Prevalence | Southern Latin America | Both | Age-standardized | Depressive disorders | Rate | 2018 | 2968.337043 | 3379.251299 | 2657.749269 |
| Prevalence | Southern Latin America | Both | Age-standardized | Depressive disorders | Rate | 2019 | 2962.635321 | 3369.973128 | 2645.21395 |
| Prevalence | Southern Latin America | Both | Age-standardized | Depressive disorders | Rate | 2020 | 3611.808508 | 4278.845763 | 3061.300408 |
| Prevalence | Southern Latin America | Both | Age-standardized | Depressive disorders | Rate | 2021 | 3605.125044 | 4246.275133 | 3048.17954 |
| Prevalence | Western Europe | Both | Age-standardized | Depressive disorders | Rate | 1990 | 4212.387003 | 4693.036659 | 3827.552659 |
| Prevalence | Western Europe | Both | Age-standardized | Depressive disorders | Rate | 1991 | 4175.692283 | 4645.436052 | 3803.312978 |
| Prevalence | Western Europe | Both | Age-standardized | Depressive disorders | Rate | 1992 | 4143.629583 | 4600.072391 | 3780.988682 |
| Prevalence | Western Europe | Both | Age-standardized | Depressive disorders | Rate | 1993 | 4117.732747 | 4571.588847 | 3760.341568 |
| Prevalence | Western Europe | Both | Age-standardized | Depressive disorders | Rate | 1994 | 4099.099316 | 4546.627443 | 3742.664416 |
| Prevalence | Western Europe | Both | Age-standardized | Depressive disorders | Rate | 1995 | 4088.391245 | 4532.499017 | 3732.428579 |
| Prevalence | Western Europe | Both | Age-standardized | Depressive disorders | Rate | 1996 | 4090.160954 | 4532.808787 | 3729.37359 |
| Prevalence | Western Europe | Both | Age-standardized | Depressive disorders | Rate | 1997 | 4103.38508 | 4545.168821 | 3737.591641 |
| Prevalence | Western Europe | Both | Age-standardized | Depressive disorders | Rate | 1998 | 4121.226285 | 4562.597189 | 3749.31278 |
| Prevalence | Western Europe | Both | Age-standardized | Depressive disorders | Rate | 1999 | 4136.35053 | 4582.478067 | 3758.824892 |
| Prevalence | Western Europe | Both | Age-standardized | Depressive disorders | Rate | 2000 | 4141.252873 | 4594.927192 | 3760.127145 |
| Prevalence | Western Europe | Both | Age-standardized | Depressive disorders | Rate | 2001 | 4130.998374 | 4578.845608 | 3748.255825 |
| Prevalence | Western Europe | Both | Age-standardized | Depressive disorders | Rate | 2002 | 4109.361491 | 4564.677223 | 3726.297238 |
| Prevalence | Western Europe | Both | Age-standardized | Depressive disorders | Rate | 2003 | 4083.011235 | 4541.043158 | 3698.593466 |
| Prevalence | Western Europe | Both | Age-standardized | Depressive disorders | Rate | 2004 | 4058.535728 | 4522.71102 | 3673.383346 |
| Prevalence | Western Europe | Both | Age-standardized | Depressive disorders | Rate | 2005 | 4042.344957 | 4512.118259 | 3657.973394 |
| Prevalence | Western Europe | Both | Age-standardized | Depressive disorders | Rate | 2006 | 4040.946097 | 4507.93944 | 3656.085852 |
| Prevalence | Western Europe | Both | Age-standardized | Depressive disorders | Rate | 2007 | 4052.045919 | 4524.381231 | 3663.76692 |
| Prevalence | Western Europe | Both | Age-standardized | Depressive disorders | Rate | 2008 | 4069.587788 | 4546.928254 | 3683.128898 |
| Prevalence | Western Europe | Both | Age-standardized | Depressive disorders | Rate | 2009 | 4087.158178 | 4569.656401 | 3702.38632 |
| Prevalence | Western Europe | Both | Age-standardized | Depressive disorders | Rate | 2010 | 4098.261803 | 4577.45455 | 3717.545587 |
| Prevalence | Western Europe | Both | Age-standardized | Depressive disorders | Rate | 2011 | 4099.609612 | 4584.18687 | 3714.444568 |
| Prevalence | Western Europe | Both | Age-standardized | Depressive disorders | Rate | 2012 | 4094.674121 | 4584.081762 | 3704.551516 |
| Prevalence | Western Europe | Both | Age-standardized | Depressive disorders | Rate | 2013 | 4087.437177 | 4583.11534 | 3695.896597 |
| Prevalence | Western Europe | Both | Age-standardized | Depressive disorders | Rate | 2014 | 4081.937929 | 4586.422448 | 3687.167734 |
| Prevalence | Western Europe | Both | Age-standardized | Depressive disorders | Rate | 2015 | 4082.075358 | 4600.848933 | 3685.320828 |
| Prevalence | Western Europe | Both | Age-standardized | Depressive disorders | Rate | 2016 | 4088.947823 | 4618.871386 | 3677.935016 |
| Prevalence | Western Europe | Both | Age-standardized | Depressive disorders | Rate | 2017 | 4097.543894 | 4645.312783 | 3666.754815 |
| Prevalence | Western Europe | Both | Age-standardized | Depressive disorders | Rate | 2018 | 4103.098861 | 4665.927875 | 3654.756503 |
| Prevalence | Western Europe | Both | Age-standardized | Depressive disorders | Rate | 2019 | 4101.067024 | 4676.449142 | 3646.159499 |
| Prevalence | Western Europe | Both | Age-standardized | Depressive disorders | Rate | 2020 | 4774.425309 | 5522.564041 | 4214.412749 |
| Prevalence | Western Europe | Both | Age-standardized | Depressive disorders | Rate | 2021 | 4778.950481 | 5528.960001 | 4207.886496 |
| Prevalence | Central Sub-Saharan Africa | Both | Age-standardized | Depressive disorders | Rate | 1990 | 6100.874374 | 7235.874297 | 5269.076271 |
| Prevalence | Central Sub-Saharan Africa | Both | Age-standardized | Depressive disorders | Rate | 1991 | 6089.553385 | 7183.323392 | 5260.481295 |
| Prevalence | Central Sub-Saharan Africa | Both | Age-standardized | Depressive disorders | Rate | 1992 | 6079.036023 | 7140.449818 | 5256.65835 |
| Prevalence | Central Sub-Saharan Africa | Both | Age-standardized | Depressive disorders | Rate | 1993 | 6069.720325 | 7079.549765 | 5253.722089 |
| Prevalence | Central Sub-Saharan Africa | Both | Age-standardized | Depressive disorders | Rate | 1994 | 6061.80095 | 7070.544833 | 5232.346993 |
| Prevalence | Central Sub-Saharan Africa | Both | Age-standardized | Depressive disorders | Rate | 1995 | 6055.922132 | 7069.557256 | 5231.68075 |
| Prevalence | Central Sub-Saharan Africa | Both | Age-standardized | Depressive disorders | Rate | 1996 | 6051.420532 | 7059.44891 | 5220.819542 |
| Prevalence | Central Sub-Saharan Africa | Both | Age-standardized | Depressive disorders | Rate | 1997 | 6047.724662 | 7042.556824 | 5227.831342 |
| Prevalence | Central Sub-Saharan Africa | Both | Age-standardized | Depressive disorders | Rate | 1998 | 6044.207944 | 7025.127547 | 5234.925926 |
| Prevalence | Central Sub-Saharan Africa | Both | Age-standardized | Depressive disorders | Rate | 1999 | 6040.591437 | 7003.665603 | 5239.314629 |
| Prevalence | Central Sub-Saharan Africa | Both | Age-standardized | Depressive disorders | Rate | 2000 | 6036.691541 | 6984.855985 | 5231.986197 |
| Prevalence | Central Sub-Saharan Africa | Both | Age-standardized | Depressive disorders | Rate | 2001 | 6030.97191 | 6975.472879 | 5234.124587 |
| Prevalence | Central Sub-Saharan Africa | Both | Age-standardized | Depressive disorders | Rate | 2002 | 6023.143482 | 6978.855617 | 5238.503014 |
| Prevalence | Central Sub-Saharan Africa | Both | Age-standardized | Depressive disorders | Rate | 2003 | 6013.973373 | 6960.674565 | 5236.52924 |
| Prevalence | Central Sub-Saharan Africa | Both | Age-standardized | Depressive disorders | Rate | 2004 | 6004.289915 | 6958.649956 | 5225.390877 |
| Prevalence | Central Sub-Saharan Africa | Both | Age-standardized | Depressive disorders | Rate | 2005 | 5994.997995 | 6979.084894 | 5217.51889 |
| Prevalence | Central Sub-Saharan Africa | Both | Age-standardized | Depressive disorders | Rate | 2006 | 5981.081173 | 6909.504524 | 5224.663666 |
| Prevalence | Central Sub-Saharan Africa | Both | Age-standardized | Depressive disorders | Rate | 2007 | 5960.691828 | 6881.760097 | 5215.195879 |
| Prevalence | Central Sub-Saharan Africa | Both | Age-standardized | Depressive disorders | Rate | 2008 | 5938.866661 | 6855.488869 | 5214.893958 |
| Prevalence | Central Sub-Saharan Africa | Both | Age-standardized | Depressive disorders | Rate | 2009 | 5920.642061 | 6831.114042 | 5210.468768 |
| Prevalence | Central Sub-Saharan Africa | Both | Age-standardized | Depressive disorders | Rate | 2010 | 5911.079452 | 6811.036574 | 5199.101199 |
| Prevalence | Central Sub-Saharan Africa | Both | Age-standardized | Depressive disorders | Rate | 2011 | 5908.332001 | 6822.824332 | 5179.324132 |
| Prevalence | Central Sub-Saharan Africa | Both | Age-standardized | Depressive disorders | Rate | 2012 | 5907.466556 | 6797.292872 | 5154.656516 |
| Prevalence | Central Sub-Saharan Africa | Both | Age-standardized | Depressive disorders | Rate | 2013 | 5907.97744 | 6788.777156 | 5142.107119 |
| Prevalence | Central Sub-Saharan Africa | Both | Age-standardized | Depressive disorders | Rate | 2014 | 5909.33347 | 6838.292941 | 5129.414676 |
| Prevalence | Central Sub-Saharan Africa | Both | Age-standardized | Depressive disorders | Rate | 2015 | 5911.453965 | 6846.002794 | 5114.59321 |
| Prevalence | Central Sub-Saharan Africa | Both | Age-standardized | Depressive disorders | Rate | 2016 | 5915.57279 | 6891.347519 | 5114.277539 |
| Prevalence | Central Sub-Saharan Africa | Both | Age-standardized | Depressive disorders | Rate | 2017 | 5921.864768 | 6910.247595 | 5144.573084 |
| Prevalence | Central Sub-Saharan Africa | Both | Age-standardized | Depressive disorders | Rate | 2018 | 5927.628251 | 6930.059021 | 5152.686826 |
| Prevalence | Central Sub-Saharan Africa | Both | Age-standardized | Depressive disorders | Rate | 2019 | 5930.625305 | 6948.274938 | 5162.098184 |
| Prevalence | Central Sub-Saharan Africa | Both | Age-standardized | Depressive disorders | Rate | 2020 | 6498.883212 | 7920.102418 | 5399.921511 |
| Prevalence | Central Sub-Saharan Africa | Both | Age-standardized | Depressive disorders | Rate | 2021 | 6337.030541 | 7669.980773 | 5236.437556 |
| Prevalence | Central Latin America | Both | Age-standardized | Depressive disorders | Rate | 1990 | 3046.7905 | 3461.755394 | 2703.861597 |
| Prevalence | Central Latin America | Both | Age-standardized | Depressive disorders | Rate | 1991 | 3059.402051 | 3468.19107 | 2718.608808 |
| Prevalence | Central Latin America | Both | Age-standardized | Depressive disorders | Rate | 1992 | 3072.32599 | 3476.404651 | 2734.125456 |
| Prevalence | Central Latin America | Both | Age-standardized | Depressive disorders | Rate | 1993 | 3084.817419 | 3483.911761 | 2746.12922 |
| Prevalence | Central Latin America | Both | Age-standardized | Depressive disorders | Rate | 1994 | 3096.15816 | 3492.348047 | 2761.148494 |
| Prevalence | Central Latin America | Both | Age-standardized | Depressive disorders | Rate | 1995 | 3105.632191 | 3497.542858 | 2775.287817 |
| Prevalence | Central Latin America | Both | Age-standardized | Depressive disorders | Rate | 1996 | 3117.424705 | 3506.827084 | 2789.028761 |
| Prevalence | Central Latin America | Both | Age-standardized | Depressive disorders | Rate | 1997 | 3133.781544 | 3520.51427 | 2806.828469 |
| Prevalence | Central Latin America | Both | Age-standardized | Depressive disorders | Rate | 1998 | 3151.420746 | 3537.925111 | 2827.193614 |
| Prevalence | Central Latin America | Both | Age-standardized | Depressive disorders | Rate | 1999 | 3167.103417 | 3553.989247 | 2844.710514 |
| Prevalence | Central Latin America | Both | Age-standardized | Depressive disorders | Rate | 2000 | 3177.450926 | 3566.580175 | 2856.677361 |
| Prevalence | Central Latin America | Both | Age-standardized | Depressive disorders | Rate | 2001 | 3183.705751 | 3569.069168 | 2862.367061 |
| Prevalence | Central Latin America | Both | Age-standardized | Depressive disorders | Rate | 2002 | 3189.282491 | 3571.495945 | 2867.611392 |
| Prevalence | Central Latin America | Both | Age-standardized | Depressive disorders | Rate | 2003 | 3194.383793 | 3574.12684 | 2873.24704 |
| Prevalence | Central Latin America | Both | Age-standardized | Depressive disorders | Rate | 2004 | 3199.178634 | 3581.677954 | 2878.397728 |
| Prevalence | Central Latin America | Both | Age-standardized | Depressive disorders | Rate | 2005 | 3203.793266 | 3586.727511 | 2883.966132 |
| Prevalence | Central Latin America | Both | Age-standardized | Depressive disorders | Rate | 2006 | 3212.293283 | 3595.095116 | 2893.770255 |
| Prevalence | Central Latin America | Both | Age-standardized | Depressive disorders | Rate | 2007 | 3226.092007 | 3605.508508 | 2910.556277 |
| Prevalence | Central Latin America | Both | Age-standardized | Depressive disorders | Rate | 2008 | 3241.588336 | 3618.530447 | 2927.973642 |
| Prevalence | Central Latin America | Both | Age-standardized | Depressive disorders | Rate | 2009 | 3255.126007 | 3631.468717 | 2940.759659 |
| Prevalence | Central Latin America | Both | Age-standardized | Depressive disorders | Rate | 2010 | 3262.880247 | 3633.089751 | 2941.544854 |
| Prevalence | Central Latin America | Both | Age-standardized | Depressive disorders | Rate | 2011 | 3264.537796 | 3637.644208 | 2938.509238 |
| Prevalence | Central Latin America | Both | Age-standardized | Depressive disorders | Rate | 2012 | 3263.313079 | 3644.328449 | 2931.264706 |
| Prevalence | Central Latin America | Both | Age-standardized | Depressive disorders | Rate | 2013 | 3260.798079 | 3649.03744 | 2926.944648 |
| Prevalence | Central Latin America | Both | Age-standardized | Depressive disorders | Rate | 2014 | 3258.616386 | 3652.295908 | 2922.025093 |
| Prevalence | Central Latin America | Both | Age-standardized | Depressive disorders | Rate | 2015 | 3258.522422 | 3658.668695 | 2918.240809 |
| Prevalence | Central Latin America | Both | Age-standardized | Depressive disorders | Rate | 2016 | 3262.391867 | 3667.621234 | 2917.233702 |
| Prevalence | Central Latin America | Both | Age-standardized | Depressive disorders | Rate | 2017 | 3268.787884 | 3687.141101 | 2914.265312 |
| Prevalence | Central Latin America | Both | Age-standardized | Depressive disorders | Rate | 2018 | 3274.218049 | 3699.618115 | 2915.101425 |
| Prevalence | Central Latin America | Both | Age-standardized | Depressive disorders | Rate | 2019 | 3275.539389 | 3704.560298 | 2914.299103 |
| Prevalence | Central Latin America | Both | Age-standardized | Depressive disorders | Rate | 2020 | 3901.284924 | 4478.288092 | 3468.66605 |
| Prevalence | Central Latin America | Both | Age-standardized | Depressive disorders | Rate | 2021 | 3825.604032 | 4375.351881 | 3399.906076 |
| Prevalence | North Africa and Middle East | Both | Age-standardized | Depressive disorders | Rate | 1990 | 4468.968673 | 5216.627039 | 3904.157021 |
| Prevalence | North Africa and Middle East | Both | Age-standardized | Depressive disorders | Rate | 1991 | 4461.984833 | 5198.82847 | 3908.68834 |
| Prevalence | North Africa and Middle East | Both | Age-standardized | Depressive disorders | Rate | 1992 | 4454.728287 | 5178.077408 | 3909.412425 |
| Prevalence | North Africa and Middle East | Both | Age-standardized | Depressive disorders | Rate | 1993 | 4447.815776 | 5162.885515 | 3913.075694 |
| Prevalence | North Africa and Middle East | Both | Age-standardized | Depressive disorders | Rate | 1994 | 4441.965222 | 5140.340909 | 3910.153811 |
| Prevalence | North Africa and Middle East | Both | Age-standardized | Depressive disorders | Rate | 1995 | 4437.850715 | 5131.871257 | 3908.386586 |
| Prevalence | North Africa and Middle East | Both | Age-standardized | Depressive disorders | Rate | 1996 | 4430.091294 | 5099.913306 | 3915.260696 |
| Prevalence | North Africa and Middle East | Both | Age-standardized | Depressive disorders | Rate | 1997 | 4415.985598 | 5062.56308 | 3913.853789 |
| Prevalence | North Africa and Middle East | Both | Age-standardized | Depressive disorders | Rate | 1998 | 4400.563107 | 5028.340927 | 3912.470778 |
| Prevalence | North Africa and Middle East | Both | Age-standardized | Depressive disorders | Rate | 1999 | 4388.688735 | 4994.347159 | 3914.095973 |
| Prevalence | North Africa and Middle East | Both | Age-standardized | Depressive disorders | Rate | 2000 | 4384.984739 | 4980.643011 | 3916.28993 |
| Prevalence | North Africa and Middle East | Both | Age-standardized | Depressive disorders | Rate | 2001 | 4391.555356 | 4982.301469 | 3921.327983 |
| Prevalence | North Africa and Middle East | Both | Age-standardized | Depressive disorders | Rate | 2002 | 4404.779194 | 5001.235948 | 3932.734986 |
| Prevalence | North Africa and Middle East | Both | Age-standardized | Depressive disorders | Rate | 2003 | 4420.31966 | 5024.394158 | 3946.464664 |
| Prevalence | North Africa and Middle East | Both | Age-standardized | Depressive disorders | Rate | 2004 | 4433.223981 | 5039.465427 | 3955.114921 |
| Prevalence | North Africa and Middle East | Both | Age-standardized | Depressive disorders | Rate | 2005 | 4439.276521 | 5045.78507 | 3954.949423 |
| Prevalence | North Africa and Middle East | Both | Age-standardized | Depressive disorders | Rate | 2006 | 4441.547938 | 5056.589228 | 3964.821103 |
| Prevalence | North Africa and Middle East | Both | Age-standardized | Depressive disorders | Rate | 2007 | 4444.852297 | 5064.234599 | 3966.99976 |
| Prevalence | North Africa and Middle East | Both | Age-standardized | Depressive disorders | Rate | 2008 | 4447.39146 | 5075.736861 | 3962.33453 |
| Prevalence | North Africa and Middle East | Both | Age-standardized | Depressive disorders | Rate | 2009 | 4449.404372 | 5084.273118 | 3957.675857 |
| Prevalence | North Africa and Middle East | Both | Age-standardized | Depressive disorders | Rate | 2010 | 4451.779326 | 5092.429352 | 3958.935835 |
| Prevalence | North Africa and Middle East | Both | Age-standardized | Depressive disorders | Rate | 2011 | 4462.053919 | 5109.441092 | 3964.963262 |
| Prevalence | North Africa and Middle East | Both | Age-standardized | Depressive disorders | Rate | 2012 | 4484.370538 | 5152.380006 | 3976.021272 |
| Prevalence | North Africa and Middle East | Both | Age-standardized | Depressive disorders | Rate | 2013 | 4511.175236 | 5202.843127 | 3983.926333 |
| Prevalence | North Africa and Middle East | Both | Age-standardized | Depressive disorders | Rate | 2014 | 4534.271659 | 5250.680635 | 3990.277941 |
| Prevalence | North Africa and Middle East | Both | Age-standardized | Depressive disorders | Rate | 2015 | 4544.500878 | 5278.460486 | 3986.127213 |
| Prevalence | North Africa and Middle East | Both | Age-standardized | Depressive disorders | Rate | 2016 | 4540.950474 | 5285.17693 | 3974.380032 |
| Prevalence | North Africa and Middle East | Both | Age-standardized | Depressive disorders | Rate | 2017 | 4530.884725 | 5296.164542 | 3956.807479 |
| Prevalence | North Africa and Middle East | Both | Age-standardized | Depressive disorders | Rate | 2018 | 4517.619777 | 5292.922794 | 3937.022066 |
| Prevalence | North Africa and Middle East | Both | Age-standardized | Depressive disorders | Rate | 2019 | 4504.334852 | 5279.85045 | 3920.099382 |
| Prevalence | North Africa and Middle East | Both | Age-standardized | Depressive disorders | Rate | 2020 | 5099.917868 | 5950.55198 | 4387.353413 |
| Prevalence | North Africa and Middle East | Both | Age-standardized | Depressive disorders | Rate | 2021 | 5024.694477 | 5857.37982 | 4346.394973 |
| Prevalence | Caribbean | Both | Age-standardized | Depressive disorders | Rate | 1990 | 3911.569598 | 4461.461184 | 3420.758011 |
| Prevalence | Caribbean | Both | Age-standardized | Depressive disorders | Rate | 1991 | 3895.384612 | 4439.766986 | 3420.334894 |
| Prevalence | Caribbean | Both | Age-standardized | Depressive disorders | Rate | 1992 | 3877.575668 | 4412.724311 | 3422.338119 |
| Prevalence | Caribbean | Both | Age-standardized | Depressive disorders | Rate | 1993 | 3858.844533 | 4389.327429 | 3408.780147 |
| Prevalence | Caribbean | Both | Age-standardized | Depressive disorders | Rate | 1994 | 3839.817376 | 4372.823397 | 3399.850236 |
| Prevalence | Caribbean | Both | Age-standardized | Depressive disorders | Rate | 1995 | 3821.313287 | 4355.390085 | 3388.305877 |
| Prevalence | Caribbean | Both | Age-standardized | Depressive disorders | Rate | 1996 | 3802.710268 | 4334.564633 | 3370.790639 |
| Prevalence | Caribbean | Both | Age-standardized | Depressive disorders | Rate | 1997 | 3783.471119 | 4306.063553 | 3353.687592 |
| Prevalence | Caribbean | Both | Age-standardized | Depressive disorders | Rate | 1998 | 3764.224462 | 4282.22938 | 3335.236907 |
| Prevalence | Caribbean | Both | Age-standardized | Depressive disorders | Rate | 1999 | 3745.18013 | 4254.544574 | 3320.230556 |
| Prevalence | Caribbean | Both | Age-standardized | Depressive disorders | Rate | 2000 | 3726.453049 | 4226.816211 | 3295.466465 |
| Prevalence | Caribbean | Both | Age-standardized | Depressive disorders | Rate | 2001 | 3708.947267 | 4213.503035 | 3292.071889 |
| Prevalence | Caribbean | Both | Age-standardized | Depressive disorders | Rate | 2002 | 3693.103545 | 4202.808652 | 3285.854737 |
| Prevalence | Caribbean | Both | Age-standardized | Depressive disorders | Rate | 2003 | 3678.124499 | 4184.09995 | 3273.90901 |
| Prevalence | Caribbean | Both | Age-standardized | Depressive disorders | Rate | 2004 | 3663.035833 | 4169.919799 | 3256.155211 |
| Prevalence | Caribbean | Both | Age-standardized | Depressive disorders | Rate | 2005 | 3647.064771 | 4154.303678 | 3236.601326 |
| Prevalence | Caribbean | Both | Age-standardized | Depressive disorders | Rate | 2006 | 3624.119988 | 4129.534842 | 3216.029212 |
| Prevalence | Caribbean | Both | Age-standardized | Depressive disorders | Rate | 2007 | 3593.536523 | 4087.638297 | 3190.92006 |
| Prevalence | Caribbean | Both | Age-standardized | Depressive disorders | Rate | 2008 | 3562.339752 | 4057.189281 | 3166.168252 |
| Prevalence | Caribbean | Both | Age-standardized | Depressive disorders | Rate | 2009 | 3537.76898 | 4017.532938 | 3144.906473 |
| Prevalence | Caribbean | Both | Age-standardized | Depressive disorders | Rate | 2010 | 3526.424748 | 3993.457115 | 3141.477444 |
| Prevalence | Caribbean | Both | Age-standardized | Depressive disorders | Rate | 2011 | 3524.391716 | 4008.830495 | 3136.068033 |
| Prevalence | Caribbean | Both | Age-standardized | Depressive disorders | Rate | 2012 | 3523.415385 | 4012.907129 | 3124.543388 |
| Prevalence | Caribbean | Both | Age-standardized | Depressive disorders | Rate | 2013 | 3523.483441 | 4031.056768 | 3123.443319 |
| Prevalence | Caribbean | Both | Age-standardized | Depressive disorders | Rate | 2014 | 3525.043665 | 4041.172907 | 3121.454248 |
| Prevalence | Caribbean | Both | Age-standardized | Depressive disorders | Rate | 2015 | 3528.549673 | 4045.408485 | 3111.442683 |
| Prevalence | Caribbean | Both | Age-standardized | Depressive disorders | Rate | 2016 | 3533.415863 | 4059.32845 | 3118.65062 |
| Prevalence | Caribbean | Both | Age-standardized | Depressive disorders | Rate | 2017 | 3539.404518 | 4082.387649 | 3116.734085 |
| Prevalence | Caribbean | Both | Age-standardized | Depressive disorders | Rate | 2018 | 3545.833244 | 4103.177802 | 3113.940608 |
| Prevalence | Caribbean | Both | Age-standardized | Depressive disorders | Rate | 2019 | 3551.903735 | 4119.444362 | 3108.907761 |
| Prevalence | Caribbean | Both | Age-standardized | Depressive disorders | Rate | 2020 | 4003.805509 | 4716.031331 | 3416.766463 |
| Prevalence | Caribbean | Both | Age-standardized | Depressive disorders | Rate | 2021 | 4121.501704 | 4870.279431 | 3512.088316 |
| Prevalence | Western Sub-Saharan Africa | Both | Age-standardized | Depressive disorders | Rate | 1990 | 4364.974669 | 4941.999527 | 3895.289571 |
| Prevalence | Western Sub-Saharan Africa | Both | Age-standardized | Depressive disorders | Rate | 1991 | 4359.432908 | 4927.489526 | 3898.775975 |
| Prevalence | Western Sub-Saharan Africa | Both | Age-standardized | Depressive disorders | Rate | 1992 | 4356.315806 | 4918.606134 | 3899.181425 |
| Prevalence | Western Sub-Saharan Africa | Both | Age-standardized | Depressive disorders | Rate | 1993 | 4355.191252 | 4913.039204 | 3902.734111 |
| Prevalence | Western Sub-Saharan Africa | Both | Age-standardized | Depressive disorders | Rate | 1994 | 4355.547713 | 4908.516192 | 3907.230519 |
| Prevalence | Western Sub-Saharan Africa | Both | Age-standardized | Depressive disorders | Rate | 1995 | 4357.071145 | 4906.37576 | 3913.130036 |
| Prevalence | Western Sub-Saharan Africa | Both | Age-standardized | Depressive disorders | Rate | 1996 | 4366.956066 | 4914.921338 | 3928.005454 |
| Prevalence | Western Sub-Saharan Africa | Both | Age-standardized | Depressive disorders | Rate | 1997 | 4387.72151 | 4938.213004 | 3950.356968 |
| Prevalence | Western Sub-Saharan Africa | Both | Age-standardized | Depressive disorders | Rate | 1998 | 4412.206258 | 4964.075655 | 3974.883719 |
| Prevalence | Western Sub-Saharan Africa | Both | Age-standardized | Depressive disorders | Rate | 1999 | 4433.472613 | 4985.911067 | 3995.429781 |
| Prevalence | Western Sub-Saharan Africa | Both | Age-standardized | Depressive disorders | Rate | 2000 | 4444.72689 | 4997.405272 | 4004.86535 |
| Prevalence | Western Sub-Saharan Africa | Both | Age-standardized | Depressive disorders | Rate | 2001 | 4449.997876 | 5002.400697 | 4008.700845 |
| Prevalence | Western Sub-Saharan Africa | Both | Age-standardized | Depressive disorders | Rate | 2002 | 4456.641416 | 5009.077501 | 4014.051399 |
| Prevalence | Western Sub-Saharan Africa | Both | Age-standardized | Depressive disorders | Rate | 2003 | 4462.959894 | 5015.142762 | 4019.420669 |
| Prevalence | Western Sub-Saharan Africa | Both | Age-standardized | Depressive disorders | Rate | 2004 | 4467.494771 | 5018.969924 | 4023.092187 |
| Prevalence | Western Sub-Saharan Africa | Both | Age-standardized | Depressive disorders | Rate | 2005 | 4468.770762 | 5020.61647 | 4023.493946 |
| Prevalence | Western Sub-Saharan Africa | Both | Age-standardized | Depressive disorders | Rate | 2006 | 4453.875059 | 4998.357419 | 4010.603019 |
| Prevalence | Western Sub-Saharan Africa | Both | Age-standardized | Depressive disorders | Rate | 2007 | 4418.712334 | 4950.062663 | 3980.313224 |
| Prevalence | Western Sub-Saharan Africa | Both | Age-standardized | Depressive disorders | Rate | 2008 | 4374.71443 | 4890.949998 | 3942.506675 |
| Prevalence | Western Sub-Saharan Africa | Both | Age-standardized | Depressive disorders | Rate | 2009 | 4333.457448 | 4836.687014 | 3909.572025 |
| Prevalence | Western Sub-Saharan Africa | Both | Age-standardized | Depressive disorders | Rate | 2010 | 4306.413035 | 4804.578086 | 3889.999565 |
| Prevalence | Western Sub-Saharan Africa | Both | Age-standardized | Depressive disorders | Rate | 2011 | 4287.392028 | 4786.275118 | 3866.749817 |
| Prevalence | Western Sub-Saharan Africa | Both | Age-standardized | Depressive disorders | Rate | 2012 | 4265.381254 | 4764.069635 | 3844.463264 |
| Prevalence | Western Sub-Saharan Africa | Both | Age-standardized | Depressive disorders | Rate | 2013 | 4244.629572 | 4745.524539 | 3823.434594 |
| Prevalence | Western Sub-Saharan Africa | Both | Age-standardized | Depressive disorders | Rate | 2014 | 4229.673152 | 4737.520849 | 3807.749036 |
| Prevalence | Western Sub-Saharan Africa | Both | Age-standardized | Depressive disorders | Rate | 2015 | 4225.152172 | 4742.88014 | 3802.049584 |
| Prevalence | Western Sub-Saharan Africa | Both | Age-standardized | Depressive disorders | Rate | 2016 | 4227.324564 | 4751.832553 | 3808.041387 |
| Prevalence | Western Sub-Saharan Africa | Both | Age-standardized | Depressive disorders | Rate | 2017 | 4228.908304 | 4762.009014 | 3805.805985 |
| Prevalence | Western Sub-Saharan Africa | Both | Age-standardized | Depressive disorders | Rate | 2018 | 4230.152953 | 4778.536165 | 3796.677072 |
| Prevalence | Western Sub-Saharan Africa | Both | Age-standardized | Depressive disorders | Rate | 2019 | 4231.370087 | 4787.846002 | 3792.143075 |
| Prevalence | Western Sub-Saharan Africa | Both | Age-standardized | Depressive disorders | Rate | 2020 | 4470.89599 | 5064.621032 | 3966.82173 |
| Prevalence | Western Sub-Saharan Africa | Both | Age-standardized | Depressive disorders | Rate | 2021 | 4372.187964 | 4963.050446 | 3880.291031 |
| Prevalence | Andean Latin America | Both | Age-standardized | Depressive disorders | Rate | 1990 | 2821.688417 | 3232.064305 | 2476.528751 |
| Prevalence | Andean Latin America | Both | Age-standardized | Depressive disorders | Rate | 1991 | 2819.878367 | 3235.465769 | 2473.060777 |
| Prevalence | Andean Latin America | Both | Age-standardized | Depressive disorders | Rate | 1992 | 2818.162571 | 3231.607295 | 2471.849656 |
| Prevalence | Andean Latin America | Both | Age-standardized | Depressive disorders | Rate | 1993 | 2816.55912 | 3227.682929 | 2471.006826 |
| Prevalence | Andean Latin America | Both | Age-standardized | Depressive disorders | Rate | 1994 | 2815.058015 | 3214.713708 | 2472.741311 |
| Prevalence | Andean Latin America | Both | Age-standardized | Depressive disorders | Rate | 1995 | 2813.53595 | 3211.606116 | 2473.748424 |
| Prevalence | Andean Latin America | Both | Age-standardized | Depressive disorders | Rate | 1996 | 2813.247842 | 3199.084479 | 2476.50724 |
| Prevalence | Andean Latin America | Both | Age-standardized | Depressive disorders | Rate | 1997 | 2814.555035 | 3196.140809 | 2481.990504 |
| Prevalence | Andean Latin America | Both | Age-standardized | Depressive disorders | Rate | 1998 | 2816.264881 | 3187.86104 | 2489.686995 |
| Prevalence | Andean Latin America | Both | Age-standardized | Depressive disorders | Rate | 1999 | 2817.137361 | 3184.67604 | 2495.749971 |
| Prevalence | Andean Latin America | Both | Age-standardized | Depressive disorders | Rate | 2000 | 2815.765144 | 3187.845361 | 2502.687178 |
| Prevalence | Andean Latin America | Both | Age-standardized | Depressive disorders | Rate | 2001 | 2812.252363 | 3182.311304 | 2500.001074 |
| Prevalence | Andean Latin America | Both | Age-standardized | Depressive disorders | Rate | 2002 | 2807.179951 | 3179.110935 | 2497.320344 |
| Prevalence | Andean Latin America | Both | Age-standardized | Depressive disorders | Rate | 2003 | 2800.772337 | 3175.285257 | 2492.377042 |
| Prevalence | Andean Latin America | Both | Age-standardized | Depressive disorders | Rate | 2004 | 2793.332046 | 3170.077492 | 2488.859894 |
| Prevalence | Andean Latin America | Both | Age-standardized | Depressive disorders | Rate | 2005 | 2784.928178 | 3163.984199 | 2484.624376 |
| Prevalence | Andean Latin America | Both | Age-standardized | Depressive disorders | Rate | 2006 | 2770.720848 | 3148.183487 | 2474.612223 |
| Prevalence | Andean Latin America | Both | Age-standardized | Depressive disorders | Rate | 2007 | 2749.207564 | 3116.814919 | 2457.003065 |
| Prevalence | Andean Latin America | Both | Age-standardized | Depressive disorders | Rate | 2008 | 2726.123271 | 3091.103179 | 2440.103422 |
| Prevalence | Andean Latin America | Both | Age-standardized | Depressive disorders | Rate | 2009 | 2707.241852 | 3073.809828 | 2421.075275 |
| Prevalence | Andean Latin America | Both | Age-standardized | Depressive disorders | Rate | 2010 | 2698.096805 | 3067.955644 | 2408.191148 |
| Prevalence | Andean Latin America | Both | Age-standardized | Depressive disorders | Rate | 2011 | 2695.689899 | 3056.826468 | 2402.157029 |
| Prevalence | Andean Latin America | Both | Age-standardized | Depressive disorders | Rate | 2012 | 2693.200551 | 3052.231539 | 2391.510981 |
| Prevalence | Andean Latin America | Both | Age-standardized | Depressive disorders | Rate | 2013 | 2690.498232 | 3063.181407 | 2379.588179 |
| Prevalence | Andean Latin America | Both | Age-standardized | Depressive disorders | Rate | 2014 | 2688.792569 | 3087.601681 | 2367.134008 |
| Prevalence | Andean Latin America | Both | Age-standardized | Depressive disorders | Rate | 2015 | 2688.637636 | 3080.311952 | 2356.842319 |
| Prevalence | Andean Latin America | Both | Age-standardized | Depressive disorders | Rate | 2016 | 2689.809955 | 3097.30641 | 2362.29316 |
| Prevalence | Andean Latin America | Both | Age-standardized | Depressive disorders | Rate | 2017 | 2690.435288 | 3109.092654 | 2370.009488 |
| Prevalence | Andean Latin America | Both | Age-standardized | Depressive disorders | Rate | 2018 | 2689.968662 | 3107.0519 | 2357.07958 |
| Prevalence | Andean Latin America | Both | Age-standardized | Depressive disorders | Rate | 2019 | 2688.120586 | 3109.92364 | 2347.889253 |
| Prevalence | Andean Latin America | Both | Age-standardized | Depressive disorders | Rate | 2020 | 3460.079787 | 4112.81009 | 2951.216553 |
| Prevalence | Andean Latin America | Both | Age-standardized | Depressive disorders | Rate | 2021 | 3325.811476 | 3880.446 | 2851.295192 |
| Prevalence | Eastern Sub-Saharan Africa | Both | Age-standardized | Depressive disorders | Rate | 1990 | 5330.371326 | 6057.556181 | 4758.722561 |
| Prevalence | Eastern Sub-Saharan Africa | Both | Age-standardized | Depressive disorders | Rate | 1991 | 5337.005004 | 6058.394681 | 4771.450802 |
| Prevalence | Eastern Sub-Saharan Africa | Both | Age-standardized | Depressive disorders | Rate | 1992 | 5343.91231 | 6044.500424 | 4775.860564 |
| Prevalence | Eastern Sub-Saharan Africa | Both | Age-standardized | Depressive disorders | Rate | 1993 | 5350.783184 | 6049.444328 | 4791.147792 |
| Prevalence | Eastern Sub-Saharan Africa | Both | Age-standardized | Depressive disorders | Rate | 1994 | 5355.695496 | 6063.055679 | 4803.409414 |
| Prevalence | Eastern Sub-Saharan Africa | Both | Age-standardized | Depressive disorders | Rate | 1995 | 5357.532883 | 6057.979771 | 4814.57513 |
| Prevalence | Eastern Sub-Saharan Africa | Both | Age-standardized | Depressive disorders | Rate | 1996 | 5364.744724 | 6053.763545 | 4827.993425 |
| Prevalence | Eastern Sub-Saharan Africa | Both | Age-standardized | Depressive disorders | Rate | 1997 | 5377.900981 | 6055.82381 | 4849.041698 |
| Prevalence | Eastern Sub-Saharan Africa | Both | Age-standardized | Depressive disorders | Rate | 1998 | 5390.545503 | 6058.196303 | 4869.944739 |
| Prevalence | Eastern Sub-Saharan Africa | Both | Age-standardized | Depressive disorders | Rate | 1999 | 5398.371065 | 6056.397174 | 4881.306382 |
| Prevalence | Eastern Sub-Saharan Africa | Both | Age-standardized | Depressive disorders | Rate | 2000 | 5397.568603 | 6046.464313 | 4877.779385 |
| Prevalence | Eastern Sub-Saharan Africa | Both | Age-standardized | Depressive disorders | Rate | 2001 | 5389.563119 | 6041.491073 | 4874.057992 |
| Prevalence | Eastern Sub-Saharan Africa | Both | Age-standardized | Depressive disorders | Rate | 2002 | 5377.829587 | 6034.007658 | 4866.583085 |
| Prevalence | Eastern Sub-Saharan Africa | Both | Age-standardized | Depressive disorders | Rate | 2003 | 5363.392718 | 6024.460263 | 4852.438292 |
| Prevalence | Eastern Sub-Saharan Africa | Both | Age-standardized | Depressive disorders | Rate | 2004 | 5347.36651 | 6011.543227 | 4833.973724 |
| Prevalence | Eastern Sub-Saharan Africa | Both | Age-standardized | Depressive disorders | Rate | 2005 | 5330.548197 | 5994.382043 | 4815.664742 |
| Prevalence | Eastern Sub-Saharan Africa | Both | Age-standardized | Depressive disorders | Rate | 2006 | 5305.216946 | 5956.348163 | 4790.221429 |
| Prevalence | Eastern Sub-Saharan Africa | Both | Age-standardized | Depressive disorders | Rate | 2007 | 5266.719044 | 5910.388203 | 4753.009132 |
| Prevalence | Eastern Sub-Saharan Africa | Both | Age-standardized | Depressive disorders | Rate | 2008 | 5223.153718 | 5853.581833 | 4711.548217 |
| Prevalence | Eastern Sub-Saharan Africa | Both | Age-standardized | Depressive disorders | Rate | 2009 | 5182.778136 | 5806.066377 | 4668.03865 |
| Prevalence | Eastern Sub-Saharan Africa | Both | Age-standardized | Depressive disorders | Rate | 2010 | 5153.656823 | 5764.757951 | 4632.373774 |
| Prevalence | Eastern Sub-Saharan Africa | Both | Age-standardized | Depressive disorders | Rate | 2011 | 5131.347885 | 5747.411946 | 4614.228842 |
| Prevalence | Eastern Sub-Saharan Africa | Both | Age-standardized | Depressive disorders | Rate | 2012 | 5107.368394 | 5734.451533 | 4585.077093 |
| Prevalence | Eastern Sub-Saharan Africa | Both | Age-standardized | Depressive disorders | Rate | 2013 | 5085.572632 | 5716.570267 | 4558.537594 |
| Prevalence | Eastern Sub-Saharan Africa | Both | Age-standardized | Depressive disorders | Rate | 2014 | 5070.062489 | 5717.266637 | 4537.579329 |
| Prevalence | Eastern Sub-Saharan Africa | Both | Age-standardized | Depressive disorders | Rate | 2015 | 5064.847885 | 5715.96377 | 4528.72882 |
| Prevalence | Eastern Sub-Saharan Africa | Both | Age-standardized | Depressive disorders | Rate | 2016 | 5068.8035 | 5716.647335 | 4540.046982 |
| Prevalence | Eastern Sub-Saharan Africa | Both | Age-standardized | Depressive disorders | Rate | 2017 | 5075.895677 | 5725.678973 | 4555.833103 |
| Prevalence | Eastern Sub-Saharan Africa | Both | Age-standardized | Depressive disorders | Rate | 2018 | 5082.998177 | 5740.329247 | 4566.007921 |
| Prevalence | Eastern Sub-Saharan Africa | Both | Age-standardized | Depressive disorders | Rate | 2019 | 5087.139606 | 5754.191363 | 4564.314888 |
| Prevalence | Eastern Sub-Saharan Africa | Both | Age-standardized | Depressive disorders | Rate | 2020 | 5431.398659 | 6218.034748 | 4822.297787 |
| Prevalence | Eastern Sub-Saharan Africa | Both | Age-standardized | Depressive disorders | Rate | 2021 | 5576.415475 | 6372.719537 | 4939.635877 |
| Prevalence | South Asia | Both | Age-standardized | Depressive disorders | Rate | 1990 | 4307.023432 | 4834.861305 | 3862.340507 |
| Prevalence | South Asia | Both | Age-standardized | Depressive disorders | Rate | 1991 | 4402.978876 | 4932.584672 | 3952.423585 |
| Prevalence | South Asia | Both | Age-standardized | Depressive disorders | Rate | 1992 | 4487.28669 | 5025.868916 | 4034.554344 |
| Prevalence | South Asia | Both | Age-standardized | Depressive disorders | Rate | 1993 | 4554.975981 | 5104.071221 | 4096.528497 |
| Prevalence | South Asia | Both | Age-standardized | Depressive disorders | Rate | 1994 | 4601.104233 | 5158.497099 | 4140.752269 |
| Prevalence | South Asia | Both | Age-standardized | Depressive disorders | Rate | 1995 | 4620.548987 | 5178.883635 | 4154.772254 |
| Prevalence | South Asia | Both | Age-standardized | Depressive disorders | Rate | 1996 | 4616.191815 | 5168.618424 | 4153.903125 |
| Prevalence | South Asia | Both | Age-standardized | Depressive disorders | Rate | 1997 | 4599.665993 | 5149.37212 | 4139.296176 |
| Prevalence | South Asia | Both | Age-standardized | Depressive disorders | Rate | 1998 | 4578.851208 | 5124.530213 | 4117.642502 |
| Prevalence | South Asia | Both | Age-standardized | Depressive disorders | Rate | 1999 | 4561.633401 | 5104.179185 | 4098.297274 |
| Prevalence | South Asia | Both | Age-standardized | Depressive disorders | Rate | 2000 | 4556.082119 | 5099.521438 | 4097.220506 |
| Prevalence | South Asia | Both | Age-standardized | Depressive disorders | Rate | 2001 | 4570.41445 | 5109.486637 | 4113.118988 |
| Prevalence | South Asia | Both | Age-standardized | Depressive disorders | Rate | 2002 | 4599.809422 | 5138.675979 | 4141.935242 |
| Prevalence | South Asia | Both | Age-standardized | Depressive disorders | Rate | 2003 | 4633.149295 | 5182.905287 | 4172.163359 |
| Prevalence | South Asia | Both | Age-standardized | Depressive disorders | Rate | 2004 | 4659.255186 | 5213.722255 | 4189.853025 |
| Prevalence | South Asia | Both | Age-standardized | Depressive disorders | Rate | 2005 | 4666.697095 | 5225.202054 | 4188.423589 |
| Prevalence | South Asia | Both | Age-standardized | Depressive disorders | Rate | 2006 | 4594.385828 | 5124.169202 | 4139.512872 |
| Prevalence | South Asia | Both | Age-standardized | Depressive disorders | Rate | 2007 | 4429.951664 | 4926.446223 | 4007.723907 |
| Prevalence | South Asia | Both | Age-standardized | Depressive disorders | Rate | 2008 | 4234.599898 | 4698.562004 | 3838.521294 |
| Prevalence | South Asia | Both | Age-standardized | Depressive disorders | Rate | 2009 | 4068.826723 | 4504.540801 | 3698.08504 |
| Prevalence | South Asia | Both | Age-standardized | Depressive disorders | Rate | 2010 | 3992.634651 | 4406.109585 | 3638.576252 |
| Prevalence | South Asia | Both | Age-standardized | Depressive disorders | Rate | 2011 | 3984.05192 | 4401.216997 | 3625.032003 |
| Prevalence | South Asia | Both | Age-standardized | Depressive disorders | Rate | 2012 | 3980.071593 | 4401.892836 | 3618.525589 |
| Prevalence | South Asia | Both | Age-standardized | Depressive disorders | Rate | 2013 | 3979.155332 | 4401.889179 | 3615.16833 |
| Prevalence | South Asia | Both | Age-standardized | Depressive disorders | Rate | 2014 | 3979.742376 | 4401.780224 | 3612.491601 |
| Prevalence | South Asia | Both | Age-standardized | Depressive disorders | Rate | 2015 | 3979.868351 | 4408.338153 | 3610.583704 |
| Prevalence | South Asia | Both | Age-standardized | Depressive disorders | Rate | 2016 | 3977.298955 | 4404.47328 | 3610.081239 |
| Prevalence | South Asia | Both | Age-standardized | Depressive disorders | Rate | 2017 | 3972.695263 | 4408.265321 | 3603.524915 |
| Prevalence | South Asia | Both | Age-standardized | Depressive disorders | Rate | 2018 | 3968.417302 | 4416.086145 | 3598.434129 |
| Prevalence | South Asia | Both | Age-standardized | Depressive disorders | Rate | 2019 | 3966.679897 | 4429.78787 | 3588.980654 |
| Prevalence | South Asia | Both | Age-standardized | Depressive disorders | Rate | 2020 | 4494.834104 | 5086.532837 | 4035.332081 |
| Prevalence | South Asia | Both | Age-standardized | Depressive disorders | Rate | 2021 | 4500.484212 | 5106.633364 | 4034.926777 |
| Prevalence | Southern Sub-Saharan Africa | Both | Age-standardized | Depressive disorders | Rate | 1990 | 4352.894966 | 4875.222591 | 3926.746636 |
| Prevalence | Southern Sub-Saharan Africa | Both | Age-standardized | Depressive disorders | Rate | 1991 | 4310.822919 | 4814.718698 | 3886.459873 |
| Prevalence | Southern Sub-Saharan Africa | Both | Age-standardized | Depressive disorders | Rate | 1992 | 4272.319074 | 4764.128413 | 3859.937671 |
| Prevalence | Southern Sub-Saharan Africa | Both | Age-standardized | Depressive disorders | Rate | 1993 | 4239.766241 | 4716.062087 | 3835.265226 |
| Prevalence | Southern Sub-Saharan Africa | Both | Age-standardized | Depressive disorders | Rate | 1994 | 4215.628157 | 4689.265623 | 3823.905792 |
| Prevalence | Southern Sub-Saharan Africa | Both | Age-standardized | Depressive disorders | Rate | 1995 | 4202.506761 | 4673.972247 | 3807.07572 |
| Prevalence | Southern Sub-Saharan Africa | Both | Age-standardized | Depressive disorders | Rate | 1996 | 4194.830151 | 4651.726851 | 3804.445769 |
| Prevalence | Southern Sub-Saharan Africa | Both | Age-standardized | Depressive disorders | Rate | 1997 | 4185.976439 | 4636.7786 | 3797.527118 |
| Prevalence | Southern Sub-Saharan Africa | Both | Age-standardized | Depressive disorders | Rate | 1998 | 4177.197528 | 4626.595191 | 3786.66265 |
| Prevalence | Southern Sub-Saharan Africa | Both | Age-standardized | Depressive disorders | Rate | 1999 | 4169.602766 | 4617.083676 | 3777.749301 |
| Prevalence | Southern Sub-Saharan Africa | Both | Age-standardized | Depressive disorders | Rate | 2000 | 4164.30953 | 4611.02177 | 3770.733311 |
| Prevalence | Southern Sub-Saharan Africa | Both | Age-standardized | Depressive disorders | Rate | 2001 | 4156.888422 | 4603.056822 | 3761.528296 |
| Prevalence | Southern Sub-Saharan Africa | Both | Age-standardized | Depressive disorders | Rate | 2002 | 4144.408568 | 4584.846813 | 3755.703742 |
| Prevalence | Southern Sub-Saharan Africa | Both | Age-standardized | Depressive disorders | Rate | 2003 | 4130.76815 | 4567.389443 | 3747.726442 |
| Prevalence | Southern Sub-Saharan Africa | Both | Age-standardized | Depressive disorders | Rate | 2004 | 4119.99685 | 4550.954869 | 3739.702362 |
| Prevalence | Southern Sub-Saharan Africa | Both | Age-standardized | Depressive disorders | Rate | 2005 | 4116.285597 | 4546.587951 | 3733.297641 |
| Prevalence | Southern Sub-Saharan Africa | Both | Age-standardized | Depressive disorders | Rate | 2006 | 4121.773227 | 4544.88469 | 3733.807041 |
| Prevalence | Southern Sub-Saharan Africa | Both | Age-standardized | Depressive disorders | Rate | 2007 | 4133.895188 | 4561.993318 | 3743.993643 |
| Prevalence | Southern Sub-Saharan Africa | Both | Age-standardized | Depressive disorders | Rate | 2008 | 4149.978709 | 4581.274751 | 3753.896345 |
| Prevalence | Southern Sub-Saharan Africa | Both | Age-standardized | Depressive disorders | Rate | 2009 | 4167.342205 | 4606.851778 | 3774.138762 |
| Prevalence | Southern Sub-Saharan Africa | Both | Age-standardized | Depressive disorders | Rate | 2010 | 4183.415441 | 4626.190875 | 3796.715927 |
| Prevalence | Southern Sub-Saharan Africa | Both | Age-standardized | Depressive disorders | Rate | 2011 | 4205.854583 | 4652.189755 | 3809.788909 |
| Prevalence | Southern Sub-Saharan Africa | Both | Age-standardized | Depressive disorders | Rate | 2012 | 4238.166737 | 4695.735971 | 3826.400749 |
| Prevalence | Southern Sub-Saharan Africa | Both | Age-standardized | Depressive disorders | Rate | 2013 | 4272.196504 | 4735.39716 | 3848.687628 |
| Prevalence | Southern Sub-Saharan Africa | Both | Age-standardized | Depressive disorders | Rate | 2014 | 4299.68737 | 4776.896734 | 3865.259558 |
| Prevalence | Southern Sub-Saharan Africa | Both | Age-standardized | Depressive disorders | Rate | 2015 | 4312.477323 | 4801.497047 | 3863.19678 |
| Prevalence | Southern Sub-Saharan Africa | Both | Age-standardized | Depressive disorders | Rate | 2016 | 4317.237353 | 4798.922699 | 3876.101325 |
| Prevalence | Southern Sub-Saharan Africa | Both | Age-standardized | Depressive disorders | Rate | 2017 | 4324.533625 | 4817.202068 | 3894.780742 |
| Prevalence | Southern Sub-Saharan Africa | Both | Age-standardized | Depressive disorders | Rate | 2018 | 4332.477062 | 4831.895189 | 3910.493954 |
| Prevalence | Southern Sub-Saharan Africa | Both | Age-standardized | Depressive disorders | Rate | 2019 | 4339.114136 | 4848.979481 | 3910.495222 |
| Prevalence | Southern Sub-Saharan Africa | Both | Age-standardized | Depressive disorders | Rate | 2020 | 4931.092047 | 5590.144344 | 4396.790681 |
| Prevalence | Southern Sub-Saharan Africa | Both | Age-standardized | Depressive disorders | Rate | 2021 | 5113.034266 | 5818.203593 | 4540.526393 |
