# Supplementary material for: Unmasking the rising global burden of depression: A 32-year GBD analysis of gender disparities and regional hotspots in Sub-Saharan Africa
Source: PLoS One. 2025 Jul 31;20(7):e0326974. doi: 10.1371/journal.pone.0326974 (PMC12312894; doi:10.1371/journal.pone.0326974)
Supplement: S13 Table — (DOCX) [file pone.0326974.s012.docx]

| **Supplementary Table 13 Global and Regional Time Series Data on Depression DALYs (1990-2021)** | | | | | | | | | |
| --- | --- | --- | --- | --- | --- | --- | --- | --- | --- |
| **measure** | **location** | **sex** | **age** | **cause** | **metric** | **year** | **value** | **upper** | **lower** |
| DALYs | Global | Both | Age-standardized | Depressive disorders | Rate | 1990 | 600.5165145 | 818.4484039 | 420.9410044 |
| DALYs | High-income Asia Pacific | Both | Age-standardized | Depressive disorders | Rate | 1990 | 367.9259435 | 504.4642019 | 252.3993724 |
| DALYs | East Asia | Both | Age-standardized | Depressive disorders | Rate | 1990 | 470.8112738 | 635.9524744 | 329.3284428 |
| DALYs | Central Europe | Both | Age-standardized | Depressive disorders | Rate | 1990 | 472.3900209 | 642.4452901 | 328.2419727 |
| DALYs | High-income North America | Both | Age-standardized | Depressive disorders | Rate | 1990 | 640.7505764 | 864.8100122 | 446.8604815 |
| DALYs | Central Asia | Both | Age-standardized | Depressive disorders | Rate | 1990 | 582.5582549 | 795.9547014 | 401.1337327 |
| DALYs | Southeast Asia | Both | Age-standardized | Depressive disorders | Rate | 1990 | 413.3581532 | 564.4632682 | 286.1574131 |
| DALYs | Tropical Latin America | Both | Age-standardized | Depressive disorders | Rate | 1990 | 694.2972843 | 940.8609452 | 485.3787643 |
| DALYs | Central Latin America | Both | Age-standardized | Depressive disorders | Rate | 1990 | 522.1124431 | 711.5160114 | 361.2116172 |
| DALYs | Oceania | Both | Age-standardized | Depressive disorders | Rate | 1990 | 490.4096267 | 672.5972043 | 337.6586227 |
| DALYs | Australasia | Both | Age-standardized | Depressive disorders | Rate | 1990 | 787.7380101 | 1065.625361 | 546.9119759 |
| DALYs | South Asia | Both | Age-standardized | Depressive disorders | Rate | 1990 | 737.6802057 | 996.6293026 | 508.9773933 |
| DALYs | Eastern Europe | Both | Age-standardized | Depressive disorders | Rate | 1990 | 638.2646919 | 874.642306 | 441.0510846 |
| DALYs | Andean Latin America | Both | Age-standardized | Depressive disorders | Rate | 1990 | 474.6895472 | 644.8233335 | 323.595551 |
| DALYs | Southern Latin America | Both | Age-standardized | Depressive disorders | Rate | 1990 | 575.287112 | 784.1874635 | 398.2514417 |
| DALYs | North Africa and Middle East | Both | Age-standardized | Depressive disorders | Rate | 1990 | 788.1349288 | 1078.257027 | 536.4709571 |
| DALYs | Caribbean | Both | Age-standardized | Depressive disorders | Rate | 1990 | 697.3621807 | 954.7400262 | 470.0363326 |
| DALYs | Western Sub-Saharan Africa | Both | Age-standardized | Depressive disorders | Rate | 1990 | 732.2388741 | 999.6369993 | 502.8902429 |
| DALYs | South Asia | Both | Age-standardized | Depressive disorders | Rate | 1991 | 757.0344055 | 1025.643702 | 521.821145 |
| DALYs | Eastern Sub-Saharan Africa | Both | Age-standardized | Depressive disorders | Rate | 1990 | 917.7875212 | 1236.02279 | 628.4206647 |
| DALYs | Central Sub-Saharan Africa | Both | Age-standardized | Depressive disorders | Rate | 1990 | 1080.055333 | 1470.356702 | 735.7077166 |
| DALYs | High-income North America | Both | Age-standardized | Depressive disorders | Rate | 1991 | 663.1238148 | 898.4849246 | 461.1490171 |
| DALYs | East Asia | Both | Age-standardized | Depressive disorders | Rate | 1991 | 481.9591199 | 652.9994061 | 336.443721 |
| DALYs | Central Latin America | Both | Age-standardized | Depressive disorders | Rate | 1991 | 524.9737844 | 715.3233184 | 363.481755 |
| DALYs | Central Asia | Both | Age-standardized | Depressive disorders | Rate | 1991 | 579.9716066 | 791.8283227 | 400.0586227 |
| DALYs | Central Europe | Both | Age-standardized | Depressive disorders | Rate | 1991 | 471.8365627 | 644.4282349 | 326.8091223 |
| DALYs | Southern Sub-Saharan Africa | Both | Age-standardized | Depressive disorders | Rate | 1990 | 733.1611932 | 997.0163141 | 511.8718931 |
| DALYs | Oceania | Both | Age-standardized | Depressive disorders | Rate | 1991 | 489.5775361 | 671.2469848 | 336.8409175 |
| DALYs | Western Europe | Both | Age-standardized | Depressive disorders | Rate | 1990 | 739.9258653 | 999.7537951 | 517.8764837 |
| DALYs | High-income Asia Pacific | Both | Age-standardized | Depressive disorders | Rate | 1991 | 360.7863596 | 493.4312703 | 247.8668029 |
| DALYs | Caribbean | Both | Age-standardized | Depressive disorders | Rate | 1991 | 693.8151051 | 944.9013594 | 471.2815236 |
| DALYs | Eastern Europe | Both | Age-standardized | Depressive disorders | Rate | 1991 | 643.2903367 | 879.0900489 | 445.6736884 |
| DALYs | North Africa and Middle East | Both | Age-standardized | Depressive disorders | Rate | 1991 | 786.7095915 | 1071.373987 | 535.5579321 |
| DALYs | Southeast Asia | Both | Age-standardized | Depressive disorders | Rate | 1991 | 412.0658826 | 562.7863087 | 285.4195095 |
| DALYs | Western Sub-Saharan Africa | Both | Age-standardized | Depressive disorders | Rate | 1991 | 731.2337541 | 997.1829484 | 502.9546635 |
| DALYs | Australasia | Both | Age-standardized | Depressive disorders | Rate | 1991 | 793.3197261 | 1071.524271 | 556.3983294 |
| DALYs | Western Europe | Both | Age-standardized | Depressive disorders | Rate | 1991 | 732.7361705 | 990.9486846 | 513.168015 |
| DALYs | Central Europe | Both | Age-standardized | Depressive disorders | Rate | 1992 | 471.2851567 | 645.5314173 | 327.3951912 |
| DALYs | North Africa and Middle East | Both | Age-standardized | Depressive disorders | Rate | 1992 | 785.2121038 | 1072.925504 | 535.5525755 |
| DALYs | Eastern Sub-Saharan Africa | Both | Age-standardized | Depressive disorders | Rate | 1991 | 919.4041025 | 1238.105719 | 631.4359971 |
| DALYs | Central Sub-Saharan Africa | Both | Age-standardized | Depressive disorders | Rate | 1991 | 1078.582441 | 1470.238869 | 732.6417547 |
| DALYs | Southeast Asia | Both | Age-standardized | Depressive disorders | Rate | 1992 | 410.6310951 | 559.5546989 | 285.4198831 |
| DALYs | Southern Sub-Saharan Africa | Both | Age-standardized | Depressive disorders | Rate | 1991 | 724.6835018 | 988.1778538 | 505.5075253 |
| DALYs | Western Sub-Saharan Africa | Both | Age-standardized | Depressive disorders | Rate | 1992 | 730.5502073 | 996.3774226 | 503.0566598 |
| DALYs | Central Asia | Both | Age-standardized | Depressive disorders | Rate | 1992 | 577.8895313 | 787.0784812 | 399.4878584 |
| DALYs | Southern Latin America | Both | Age-standardized | Depressive disorders | Rate | 1991 | 574.8673847 | 782.2475699 | 399.1717639 |
| DALYs | Eastern Europe | Both | Age-standardized | Depressive disorders | Rate | 1992 | 647.1507181 | 889.8100331 | 447.1018908 |
| DALYs | Oceania | Both | Age-standardized | Depressive disorders | Rate | 1992 | 488.6191607 | 669.6020633 | 338.7852531 |
| DALYs | Caribbean | Both | Age-standardized | Depressive disorders | Rate | 1992 | 689.8428773 | 939.6277663 | 469.6989169 |
| DALYs | Eastern Sub-Saharan Africa | Both | Age-standardized | Depressive disorders | Rate | 1992 | 921.1701675 | 1243.750574 | 633.951715 |
| DALYs | Australasia | Both | Age-standardized | Depressive disorders | Rate | 1992 | 797.9559123 | 1075.037442 | 561.2387316 |
| DALYs | Andean Latin America | Both | Age-standardized | Depressive disorders | Rate | 1991 | 474.4375206 | 644.4887799 | 322.1365458 |
| DALYs | Tropical Latin America | Both | Age-standardized | Depressive disorders | Rate | 1991 | 691.3936536 | 936.7213381 | 481.9790477 |
| DALYs | Oceania | Both | Age-standardized | Depressive disorders | Rate | 1993 | 488.1231363 | 671.1326353 | 335.9621643 |
| DALYs | Western Europe | Both | Age-standardized | Depressive disorders | Rate | 1992 | 726.2424606 | 983.4116407 | 508.7837219 |
| DALYs | South Asia | Both | Age-standardized | Depressive disorders | Rate | 1992 | 774.0688869 | 1049.99002 | 533.2900507 |
| DALYs | Andean Latin America | Both | Age-standardized | Depressive disorders | Rate | 1992 | 474.095088 | 643.8648912 | 323.7802363 |
| DALYs | Central Asia | Both | Age-standardized | Depressive disorders | Rate | 1993 | 576.126004 | 782.8673572 | 398.0669399 |
| DALYs | Eastern Europe | Both | Age-standardized | Depressive disorders | Rate | 1993 | 650.2972468 | 894.3716826 | 447.3628997 |
| DALYs | Southeast Asia | Both | Age-standardized | Depressive disorders | Rate | 1993 | 409.2524086 | 557.2638719 | 284.989147 |
| DALYs | Southern Sub-Saharan Africa | Both | Age-standardized | Depressive disorders | Rate | 1992 | 716.7602494 | 975.7012507 | 496.8871489 |
| DALYs | Global | Both | Age-standardized | Depressive disorders | Rate | 1992 | 612.4890946 | 835.0906376 | 429.2791398 |
| DALYs | Global | Both | Age-standardized | Depressive disorders | Rate | 1991 | 606.9409608 | 827.322095 | 425.4839067 |
| DALYs | Caribbean | Both | Age-standardized | Depressive disorders | Rate | 1993 | 686.2833288 | 929.0477819 | 469.7249651 |
| DALYs | Central Sub-Saharan Africa | Both | Age-standardized | Depressive disorders | Rate | 1992 | 1076.273455 | 1459.273829 | 731.1396934 |
| DALYs | Southern Latin America | Both | Age-standardized | Depressive disorders | Rate | 1992 | 574.2215738 | 784.1275405 | 398.7889053 |
| DALYs | Western Europe | Both | Age-standardized | Depressive disorders | Rate | 1993 | 721.0958518 | 976.4190235 | 506.6038266 |
| DALYs | South Asia | Both | Age-standardized | Depressive disorders | Rate | 1993 | 787.727218 | 1072.738132 | 541.511443 |
| DALYs | Central Latin America | Both | Age-standardized | Depressive disorders | Rate | 1992 | 527.7459806 | 718.8174576 | 364.5144938 |
| DALYs | East Asia | Both | Age-standardized | Depressive disorders | Rate | 1992 | 490.5039627 | 666.1935523 | 344.155311 |
| DALYs | Tropical Latin America | Both | Age-standardized | Depressive disorders | Rate | 1992 | 690.8937635 | 936.3763419 | 480.3511175 |
| DALYs | Australasia | Both | Age-standardized | Depressive disorders | Rate | 1993 | 802.3638375 | 1086.720002 | 558.9911786 |
| DALYs | Australasia | Both | Age-standardized | Depressive disorders | Rate | 1994 | 806.0781149 | 1089.629592 | 561.2274249 |
| DALYs | Central Sub-Saharan Africa | Both | Age-standardized | Depressive disorders | Rate | 1993 | 1074.782474 | 1459.806739 | 729.2800599 |
| DALYs | Western Europe | Both | Age-standardized | Depressive disorders | Rate | 1994 | 717.3191871 | 969.7562593 | 503.2081629 |
| DALYs | Andean Latin America | Both | Age-standardized | Depressive disorders | Rate | 1993 | 474.0210048 | 647.7270318 | 322.5520656 |
| DALYs | High-income Asia Pacific | Both | Age-standardized | Depressive disorders | Rate | 1992 | 355.6829032 | 487.2508491 | 245.010223 |
| DALYs | Southern Sub-Saharan Africa | Both | Age-standardized | Depressive disorders | Rate | 1993 | 709.8737691 | 964.542753 | 492.400316 |
| DALYs | Southern Latin America | Both | Age-standardized | Depressive disorders | Rate | 1994 | 573.3868554 | 777.2183162 | 401.4162551 |
| DALYs | Southern Latin America | Both | Age-standardized | Depressive disorders | Rate | 1993 | 573.8886013 | 774.7026452 | 401.1182605 |
| DALYs | High-income North America | Both | Age-standardized | Depressive disorders | Rate | 1993 | 706.8122148 | 955.4355357 | 495.5142353 |
| DALYs | High-income North America | Both | Age-standardized | Depressive disorders | Rate | 1992 | 685.4248883 | 928.7859185 | 479.5887839 |
| DALYs | South Asia | Both | Age-standardized | Depressive disorders | Rate | 1994 | 797.2079669 | 1087.411369 | 548.0975126 |
| DALYs | Global | Both | Age-standardized | Depressive disorders | Rate | 1993 | 616.9839005 | 841.3130891 | 432.5423158 |
| DALYs | Central Sub-Saharan Africa | Both | Age-standardized | Depressive disorders | Rate | 1994 | 1073.281692 | 1460.997226 | 723.801612 |
| DALYs | Western Europe | Both | Age-standardized | Depressive disorders | Rate | 1995 | 715.2580601 | 965.9839621 | 501.8523341 |
| DALYs | Tropical Latin America | Both | Age-standardized | Depressive disorders | Rate | 1993 | 691.8588438 | 937.5781129 | 479.2658177 |
| DALYs | Andean Latin America | Both | Age-standardized | Depressive disorders | Rate | 1994 | 473.6272721 | 643.0666198 | 319.7141611 |
| DALYs | Global | Both | Age-standardized | Depressive disorders | Rate | 1994 | 620.1419046 | 845.1870254 | 434.8047795 |
| DALYs | Central Asia | Both | Age-standardized | Depressive disorders | Rate | 1994 | 574.8195331 | 780.4181519 | 396.9089077 |
| DALYs | South Asia | Both | Age-standardized | Depressive disorders | Rate | 1995 | 801.332323 | 1094.955991 | 550.179352 |
| DALYs | Southern Sub-Saharan Africa | Both | Age-standardized | Depressive disorders | Rate | 1994 | 704.6298847 | 960.2664362 | 490.6694161 |
| DALYs | East Asia | Both | Age-standardized | Depressive disorders | Rate | 1993 | 496.3666827 | 674.4382908 | 348.1530305 |
| DALYs | Eastern Europe | Both | Age-standardized | Depressive disorders | Rate | 1994 | 652.7012085 | 896.5329126 | 449.3324674 |
| DALYs | Caribbean | Both | Age-standardized | Depressive disorders | Rate | 1994 | 682.1560091 | 919.6549033 | 468.9490832 |
| DALYs | Central Latin America | Both | Age-standardized | Depressive disorders | Rate | 1993 | 530.3893252 | 722.4501299 | 367.2027637 |
| DALYs | Central Europe | Both | Age-standardized | Depressive disorders | Rate | 1993 | 470.4163847 | 643.5545738 | 326.4555049 |
| DALYs | High-income Asia Pacific | Both | Age-standardized | Depressive disorders | Rate | 1993 | 352.4616583 | 482.9562129 | 244.4080022 |
| DALYs | Tropical Latin America | Both | Age-standardized | Depressive disorders | Rate | 1994 | 694.3347486 | 940.6311548 | 480.7164448 |
| DALYs | Eastern Europe | Both | Age-standardized | Depressive disorders | Rate | 1995 | 654.2874643 | 898.2499509 | 450.379592 |
| DALYs | Western Europe | Both | Age-standardized | Depressive disorders | Rate | 1996 | 715.889929 | 966.1621818 | 502.4295723 |
| DALYs | Central Asia | Both | Age-standardized | Depressive disorders | Rate | 1995 | 573.9489705 | 774.4627031 | 395.1012483 |
| DALYs | Caribbean | Both | Age-standardized | Depressive disorders | Rate | 1995 | 678.3885133 | 917.09125 | 468.7825103 |
| DALYs | East Asia | Both | Age-standardized | Depressive disorders | Rate | 1994 | 499.294224 | 677.370445 | 350.0834321 |
| DALYs | South Asia | Both | Age-standardized | Depressive disorders | Rate | 1996 | 800.6254652 | 1092.564627 | 550.2036107 |
| DALYs | Central Latin America | Both | Age-standardized | Depressive disorders | Rate | 1994 | 532.9162758 | 724.90679 | 369.2195638 |
| DALYs | High-income North America | Both | Age-standardized | Depressive disorders | Rate | 1994 | 726.1992861 | 984.346055 | 512.1142622 |
| DALYs | Eastern Europe | Both | Age-standardized | Depressive disorders | Rate | 1996 | 654.6318992 | 896.7272055 | 450.4146765 |
| DALYs | Central Asia | Both | Age-standardized | Depressive disorders | Rate | 1996 | 573.6342512 | 778.5678791 | 394.567846 |
| DALYs | Central Europe | Both | Age-standardized | Depressive disorders | Rate | 1994 | 469.3163448 | 640.4185133 | 325.5157908 |
| DALYs | Caribbean | Both | Age-standardized | Depressive disorders | Rate | 1996 | 674.3426663 | 907.0642304 | 461.6102925 |
| DALYs | Central Sub-Saharan Africa | Both | Age-standardized | Depressive disorders | Rate | 1995 | 1072.089494 | 1460.84843 | 724.5824482 |
| DALYs | Southern Sub-Saharan Africa | Both | Age-standardized | Depressive disorders | Rate | 1995 | 701.4822349 | 951.5889318 | 489.3152899 |
| DALYs | South Asia | Both | Age-standardized | Depressive disorders | Rate | 1997 | 797.6093876 | 1088.145715 | 548.5984266 |
| DALYs | Andean Latin America | Both | Age-standardized | Depressive disorders | Rate | 1995 | 473.0593394 | 644.9002713 | 319.4788655 |
| DALYs | Southern Latin America | Both | Age-standardized | Depressive disorders | Rate | 1995 | 572.9438844 | 776.546465 | 401.5838838 |
| DALYs | Central Asia | Both | Age-standardized | Depressive disorders | Rate | 1997 | 573.8140285 | 777.6670728 | 394.6228611 |
| DALYs | High-income Asia Pacific | Both | Age-standardized | Depressive disorders | Rate | 1994 | 350.6005294 | 478.234336 | 243.1586884 |
| DALYs | Caribbean | Both | Age-standardized | Depressive disorders | Rate | 1997 | 670.4839031 | 908.4751033 | 462.2619897 |
| DALYs | Eastern Europe | Both | Age-standardized | Depressive disorders | Rate | 1997 | 653.7575593 | 896.4470435 | 450.0189552 |
| DALYs | Eastern Sub-Saharan Africa | Both | Age-standardized | Depressive disorders | Rate | 1993 | 922.7557125 | 1245.353625 | 635.0774209 |
| DALYs | North Africa and Middle East | Both | Age-standardized | Depressive disorders | Rate | 1993 | 783.881623 | 1068.089308 | 535.0935529 |
| DALYs | Global | Both | Age-standardized | Depressive disorders | Rate | 1995 | 621.7334508 | 847.2138315 | 436.1511282 |
| DALYs | Tropical Latin America | Both | Age-standardized | Depressive disorders | Rate | 1995 | 697.5905711 | 943.9856403 | 484.217734 |
| DALYs | North Africa and Middle East | Both | Age-standardized | Depressive disorders | Rate | 1994 | 782.7239643 | 1067.252956 | 536.374565 |
| DALYs | Southeast Asia | Both | Age-standardized | Depressive disorders | Rate | 1994 | 407.9622056 | 555.134099 | 284.7658543 |
| DALYs | Eastern Europe | Both | Age-standardized | Depressive disorders | Rate | 1998 | 651.9623276 | 891.4859284 | 448.2743217 |
| DALYs | Central Asia | Both | Age-standardized | Depressive disorders | Rate | 1998 | 574.362483 | 780.9924877 | 393.8979022 |
| DALYs | Caribbean | Both | Age-standardized | Depressive disorders | Rate | 1998 | 666.5173399 | 897.3601986 | 461.7255598 |
| DALYs | High-income North America | Both | Age-standardized | Depressive disorders | Rate | 1995 | 743.1797363 | 1008.629949 | 523.9804861 |
| DALYs | Eastern Sub-Saharan Africa | Both | Age-standardized | Depressive disorders | Rate | 1994 | 923.6557852 | 1246.59585 | 638.1360149 |
| DALYs | Western Sub-Saharan Africa | Both | Age-standardized | Depressive disorders | Rate | 1993 | 730.3353249 | 996.6326088 | 504.2157208 |
| DALYs | Central Latin America | Both | Age-standardized | Depressive disorders | Rate | 1995 | 534.8909683 | 729.000377 | 370.3347355 |
| DALYs | Central Asia | Both | Age-standardized | Depressive disorders | Rate | 1999 | 574.4175271 | 781.9334385 | 393.9585056 |
| DALYs | Western Sub-Saharan Africa | Both | Age-standardized | Depressive disorders | Rate | 1994 | 730.4153566 | 996.028924 | 505.1741566 |
| DALYs | Eastern Europe | Both | Age-standardized | Depressive disorders | Rate | 1999 | 650.2608168 | 889.8630409 | 448.2905725 |
| DALYs | East Asia | Both | Age-standardized | Depressive disorders | Rate | 1995 | 499.0227929 | 676.1329442 | 350.0953929 |
| DALYs | Western Europe | Both | Age-standardized | Depressive disorders | Rate | 1997 | 718.9883864 | 969.6331808 | 503.5224206 |
| DALYs | Western Europe | Both | Age-standardized | Depressive disorders | Rate | 1998 | 723.4309768 | 976.8262505 | 507.2066307 |
| DALYs | Oceania | Both | Age-standardized | Depressive disorders | Rate | 1994 | 487.4992188 | 672.0707596 | 335.2881864 |
| DALYs | Caribbean | Both | Age-standardized | Depressive disorders | Rate | 1999 | 662.2414921 | 891.5670207 | 459.6166575 |
| DALYs | Central Europe | Both | Age-standardized | Depressive disorders | Rate | 1995 | 468.2825481 | 639.3789119 | 325.4235216 |
| DALYs | South Asia | Both | Age-standardized | Depressive disorders | Rate | 1998 | 793.7993252 | 1083.248788 | 546.2056068 |
| DALYs | Oceania | Both | Age-standardized | Depressive disorders | Rate | 1995 | 487.4282567 | 666.9584604 | 335.5635122 |
| DALYs | Global | Both | Age-standardized | Depressive disorders | Rate | 1996 | 622.1542858 | 847.1701376 | 436.8881699 |
| DALYs | Western Europe | Both | Age-standardized | Depressive disorders | Rate | 1999 | 727.0900555 | 978.9479292 | 509.8663716 |
| DALYs | High-income Asia Pacific | Both | Age-standardized | Depressive disorders | Rate | 1995 | 349.8595362 | 477.4959689 | 242.1782865 |
| DALYs | Southeast Asia | Both | Age-standardized | Depressive disorders | Rate | 1995 | 406.9759566 | 553.7286935 | 283.9613043 |
| DALYs | Western Europe | Both | Age-standardized | Depressive disorders | Rate | 2000 | 728.356366 | 982.199024 | 510.4418602 |
| DALYs | East Asia | Both | Age-standardized | Depressive disorders | Rate | 1996 | 494.5064343 | 670.935792 | 347.5899006 |
| DALYs | South Asia | Both | Age-standardized | Depressive disorders | Rate | 1999 | 790.6943039 | 1076.965472 | 543.7978784 |
| DALYs | Tropical Latin America | Both | Age-standardized | Depressive disorders | Rate | 1996 | 710.5588577 | 961.0794066 | 494.0663916 |
| DALYs | High-income North America | Both | Age-standardized | Depressive disorders | Rate | 1996 | 760.928977 | 1030.867952 | 536.1647846 |
| DALYs | North Africa and Middle East | Both | Age-standardized | Depressive disorders | Rate | 1995 | 781.8777777 | 1066.454175 | 536.5465285 |
| DALYs | Eastern Sub-Saharan Africa | Both | Age-standardized | Depressive disorders | Rate | 1995 | 923.9709338 | 1244.043432 | 638.2951677 |
| DALYs | South Asia | Both | Age-standardized | Depressive disorders | Rate | 2000 | 789.6583246 | 1076.209248 | 542.2168436 |
| DALYs | Eastern Europe | Both | Age-standardized | Depressive disorders | Rate | 2000 | 648.6003232 | 886.6409175 | 446.2086825 |
| DALYs | Central Asia | Both | Age-standardized | Depressive disorders | Rate | 2000 | 574.1253044 | 783.1263256 | 395.027396 |
| DALYs | Caribbean | Both | Age-standardized | Depressive disorders | Rate | 2000 | 658.2513802 | 890.1666791 | 456.8891705 |
| DALYs | Western Sub-Saharan Africa | Both | Age-standardized | Depressive disorders | Rate | 1995 | 730.6498389 | 994.9030106 | 506.846354 |
| DALYs | Central Europe | Both | Age-standardized | Depressive disorders | Rate | 1996 | 466.6933982 | 637.2079846 | 323.3567955 |
| DALYs | High-income Asia Pacific | Both | Age-standardized | Depressive disorders | Rate | 1996 | 352.9927521 | 479.9510231 | 243.4594588 |
| DALYs | Central Latin America | Both | Age-standardized | Depressive disorders | Rate | 1996 | 537.3452441 | 733.2044141 | 372.726231 |
| DALYs | Western Europe | Both | Age-standardized | Depressive disorders | Rate | 2001 | 726.4983432 | 978.3818371 | 508.9348325 |
| DALYs | Central Asia | Both | Age-standardized | Depressive disorders | Rate | 2001 | 573.1919309 | 779.2696356 | 391.2221509 |
| DALYs | Oceania | Both | Age-standardized | Depressive disorders | Rate | 1996 | 486.7846186 | 667.7657812 | 335.8606808 |
| DALYs | South Asia | Both | Age-standardized | Depressive disorders | Rate | 2001 | 792.475432 | 1080.500887 | 544.2911424 |
| DALYs | Caribbean | Both | Age-standardized | Depressive disorders | Rate | 2001 | 654.6050416 | 882.2836375 | 452.8908748 |
| DALYs | Eastern Europe | Both | Age-standardized | Depressive disorders | Rate | 2001 | 645.7954398 | 883.4772014 | 443.6671645 |
| DALYs | Australasia | Both | Age-standardized | Depressive disorders | Rate | 1995 | 809.5316223 | 1091.26524 | 565.1689107 |
| DALYs | North Africa and Middle East | Both | Age-standardized | Depressive disorders | Rate | 1996 | 780.2278028 | 1061.64172 | 538.4577673 |
| DALYs | Eastern Sub-Saharan Africa | Both | Age-standardized | Depressive disorders | Rate | 1996 | 925.3358056 | 1245.948525 | 640.1469994 |
| DALYs | Western Sub-Saharan Africa | Both | Age-standardized | Depressive disorders | Rate | 1996 | 732.65302 | 998.4572172 | 507.1364683 |
| DALYs | East Asia | Both | Age-standardized | Depressive disorders | Rate | 1997 | 485.8018043 | 660.1086123 | 342.4429499 |
| DALYs | High-income North America | Both | Age-standardized | Depressive disorders | Rate | 1997 | 780.9048861 | 1057.611036 | 551.9816191 |
| DALYs | Central Latin America | Both | Age-standardized | Depressive disorders | Rate | 1997 | 540.7416879 | 736.8774892 | 375.7279536 |
| DALYs | Western Europe | Both | Age-standardized | Depressive disorders | Rate | 2002 | 722.2759895 | 971.9497012 | 505.2899623 |
| DALYs | Central Asia | Both | Age-standardized | Depressive disorders | Rate | 2002 | 572.3499423 | 778.7995033 | 393.2407889 |
| DALYs | Eastern Europe | Both | Age-standardized | Depressive disorders | Rate | 2002 | 640.892761 | 876.912853 | 442.1712774 |
| DALYs | High-income Asia Pacific | Both | Age-standardized | Depressive disorders | Rate | 1997 | 360.8958021 | 491.4472966 | 249.8230748 |
| DALYs | Australasia | Both | Age-standardized | Depressive disorders | Rate | 1996 | 810.534398 | 1095.229375 | 569.2125797 |
| DALYs | Caribbean | Both | Age-standardized | Depressive disorders | Rate | 2002 | 651.4528408 | 880.5718159 | 451.0105371 |
| DALYs | South Asia | Both | Age-standardized | Depressive disorders | Rate | 2002 | 798.3283863 | 1087.469597 | 547.6922079 |
| DALYs | Southern Sub-Saharan Africa | Both | Age-standardized | Depressive disorders | Rate | 1996 | 699.3084632 | 949.2867125 | 488.3212034 |
| DALYs | Southeast Asia | Both | Age-standardized | Depressive disorders | Rate | 1996 | 405.4058625 | 551.4325989 | 283.6743451 |
| DALYs | Central Europe | Both | Age-standardized | Depressive disorders | Rate | 1997 | 464.5238558 | 635.1534716 | 323.0174592 |
| DALYs | Andean Latin America | Both | Age-standardized | Depressive disorders | Rate | 1996 | 473.3417656 | 644.3343724 | 321.4099231 |
| DALYs | Southern Latin America | Both | Age-standardized | Depressive disorders | Rate | 1996 | 572.3224765 | 772.5364641 | 400.8660259 |
| DALYs | Oceania | Both | Age-standardized | Depressive disorders | Rate | 1997 | 486.503078 | 663.6441069 | 337.5035487 |
| DALYs | Southeast Asia | Both | Age-standardized | Depressive disorders | Rate | 1997 | 403.2530664 | 548.5284303 | 282.5907489 |
| DALYs | Central Sub-Saharan Africa | Both | Age-standardized | Depressive disorders | Rate | 1996 | 1071.3991 | 1454.876281 | 723.1585347 |
| DALYs | Eastern Sub-Saharan Africa | Both | Age-standardized | Depressive disorders | Rate | 1997 | 928.208583 | 1253.007133 | 643.6208824 |
| DALYs | North Africa and Middle East | Both | Age-standardized | Depressive disorders | Rate | 1997 | 777.4784954 | 1057.594307 | 538.678555 |
| DALYs | Western Sub-Saharan Africa | Both | Age-standardized | Depressive disorders | Rate | 1997 | 736.8725718 | 1001.486784 | 511.4457423 |
| DALYs | Australasia | Both | Age-standardized | Depressive disorders | Rate | 1997 | 810.5895722 | 1097.785542 | 574.1820376 |
| DALYs | Central Asia | Both | Age-standardized | Depressive disorders | Rate | 2003 | 571.4548756 | 776.4195632 | 391.4812203 |
| DALYs | Eastern Europe | Both | Age-standardized | Depressive disorders | Rate | 2003 | 634.9268001 | 870.268943 | 437.752998 |
| DALYs | Western Europe | Both | Age-standardized | Depressive disorders | Rate | 2003 | 717.1155362 | 968.7922586 | 501.7223795 |
| DALYs | Caribbean | Both | Age-standardized | Depressive disorders | Rate | 2003 | 648.5196085 | 877.6985838 | 447.8449433 |
| DALYs | Southern Latin America | Both | Age-standardized | Depressive disorders | Rate | 1997 | 571.7999719 | 777.0292419 | 399.414599 |
| DALYs | South Asia | Both | Age-standardized | Depressive disorders | Rate | 2003 | 804.5712271 | 1096.582416 | 552.4615509 |
| DALYs | Central Sub-Saharan Africa | Both | Age-standardized | Depressive disorders | Rate | 1997 | 1070.517361 | 1462.820353 | 723.7573613 |
| DALYs | Tropical Latin America | Both | Age-standardized | Depressive disorders | Rate | 1997 | 736.1152076 | 993.2397878 | 511.8276748 |
| DALYs | Southern Sub-Saharan Africa | Both | Age-standardized | Depressive disorders | Rate | 1997 | 696.9376068 | 942.0871244 | 488.033447 |
| DALYs | Andean Latin America | Both | Age-standardized | Depressive disorders | Rate | 1997 | 473.5061378 | 642.3294474 | 322.4433526 |
| DALYs | Oceania | Both | Age-standardized | Depressive disorders | Rate | 1998 | 485.9233584 | 660.9294294 | 334.6279554 |
| DALYs | Global | Both | Age-standardized | Depressive disorders | Rate | 1997 | 621.8915456 | 847.4328794 | 437.3250594 |
| DALYs | Eastern Sub-Saharan Africa | Both | Age-standardized | Depressive disorders | Rate | 1998 | 930.778154 | 1255.962139 | 644.5673065 |
| DALYs | North Africa and Middle East | Both | Age-standardized | Depressive disorders | Rate | 1998 | 774.3833662 | 1049.74355 | 537.7645579 |
| DALYs | Australasia | Both | Age-standardized | Depressive disorders | Rate | 1998 | 809.8319682 | 1094.034226 | 574.0513781 |
| DALYs | Western Sub-Saharan Africa | Both | Age-standardized | Depressive disorders | Rate | 1998 | 741.9145961 | 1008.891927 | 512.9150326 |
| DALYs | Southeast Asia | Both | Age-standardized | Depressive disorders | Rate | 1998 | 400.7403093 | 545.4104605 | 280.9789107 |
| DALYs | Central Asia | Both | Age-standardized | Depressive disorders | Rate | 2004 | 570.5586735 | 772.9586777 | 391.9484974 |
| DALYs | Southern Latin America | Both | Age-standardized | Depressive disorders | Rate | 1998 | 570.5044136 | 773.8877247 | 396.4304825 |
| DALYs | Eastern Europe | Both | Age-standardized | Depressive disorders | Rate | 2004 | 628.7843665 | 862.0691322 | 433.7599227 |
| DALYs | Oceania | Both | Age-standardized | Depressive disorders | Rate | 1999 | 485.5092378 | 659.6666667 | 332.6022821 |
| DALYs | Southern Sub-Saharan Africa | Both | Age-standardized | Depressive disorders | Rate | 1998 | 694.3741643 | 937.6518724 | 484.1368259 |
| DALYs | Caribbean | Both | Age-standardized | Depressive disorders | Rate | 2004 | 645.4535666 | 879.0410958 | 445.3663951 |
| DALYs | Western Europe | Both | Age-standardized | Depressive disorders | Rate | 2004 | 712.2403199 | 962.3276265 | 498.8072155 |
| DALYs | South Asia | Both | Age-standardized | Depressive disorders | Rate | 2004 | 809.5714997 | 1100.70953 | 555.2314329 |
| DALYs | Central Sub-Saharan Africa | Both | Age-standardized | Depressive disorders | Rate | 1998 | 1070.079771 | 1464.709394 | 721.3216933 |
| DALYs | Global | Both | Age-standardized | Depressive disorders | Rate | 1998 | 621.2924149 | 847.3890585 | 436.6790427 |
| DALYs | Andean Latin America | Both | Age-standardized | Depressive disorders | Rate | 1998 | 473.9578843 | 641.5377076 | 324.0753213 |
| DALYs | Southeast Asia | Both | Age-standardized | Depressive disorders | Rate | 1999 | 398.661887 | 543.5719689 | 279.0264985 |
| DALYs | Australasia | Both | Age-standardized | Depressive disorders | Rate | 1999 | 810.8259876 | 1088.14262 | 574.1548491 |
| DALYs | Andean Latin America | Both | Age-standardized | Depressive disorders | Rate | 1999 | 474.3545215 | 644.9875492 | 323.405739 |
| DALYs | East Asia | Both | Age-standardized | Depressive disorders | Rate | 1998 | 475.7744299 | 646.0168608 | 335.4538121 |
| DALYs | Southern Latin America | Both | Age-standardized | Depressive disorders | Rate | 1999 | 569.9394725 | 776.479279 | 391.9972424 |
| DALYs | Tropical Latin America | Both | Age-standardized | Depressive disorders | Rate | 1998 | 766.1492684 | 1031.205127 | 531.5676766 |
| DALYs | Central Latin America | Both | Age-standardized | Depressive disorders | Rate | 1998 | 544.5623018 | 743.1005739 | 378.8316168 |
| DALYs | High-income North America | Both | Age-standardized | Depressive disorders | Rate | 1998 | 799.7047576 | 1083.069823 | 563.4059295 |
| DALYs | Central Asia | Both | Age-standardized | Depressive disorders | Rate | 2005 | 569.1055983 | 778.4264233 | 389.806547 |
| DALYs | Eastern Europe | Both | Age-standardized | Depressive disorders | Rate | 2005 | 622.8854361 | 854.5267651 | 429.2284669 |
| DALYs | Central Sub-Saharan Africa | Both | Age-standardized | Depressive disorders | Rate | 1999 | 1069.160985 | 1459.891451 | 726.3148099 |
| DALYs | Western Europe | Both | Age-standardized | Depressive disorders | Rate | 2005 | 708.8362184 | 956.9885627 | 496.8904556 |
| DALYs | Caribbean | Both | Age-standardized | Depressive disorders | Rate | 2005 | 642.069379 | 877.7100026 | 442.0067561 |
| DALYs | Global | Both | Age-standardized | Depressive disorders | Rate | 1999 | 620.6213088 | 846.2352033 | 436.4344804 |
| DALYs | High-income Asia Pacific | Both | Age-standardized | Depressive disorders | Rate | 1998 | 370.8742839 | 505.3088105 | 256.0867702 |
| DALYs | Central Europe | Both | Age-standardized | Depressive disorders | Rate | 1998 | 462.0860147 | 629.9161755 | 321.9841259 |
| DALYs | Southern Sub-Saharan Africa | Both | Age-standardized | Depressive disorders | Rate | 1999 | 692.4337022 | 933.7374455 | 485.4788055 |
| DALYs | Andean Latin America | Both | Age-standardized | Depressive disorders | Rate | 2000 | 473.8358012 | 642.3115313 | 327.3411535 |
| DALYs | South Asia | Both | Age-standardized | Depressive disorders | Rate | 2005 | 810.7490878 | 1103.199345 | 555.379062 |
| DALYs | Southern Latin America | Both | Age-standardized | Depressive disorders | Rate | 2000 | 570.0206985 | 776.8425722 | 391.0375637 |
| DALYs | Global | Both | Age-standardized | Depressive disorders | Rate | 2000 | 620.1305351 | 846.1915579 | 436.1933757 |
| DALYs | Southern Sub-Saharan Africa | Both | Age-standardized | Depressive disorders | Rate | 2000 | 690.7348247 | 927.7213183 | 484.4026651 |
| DALYs | Tropical Latin America | Both | Age-standardized | Depressive disorders | Rate | 1999 | 791.7361631 | 1067.194515 | 551.2830052 |
| DALYs | Central Latin America | Both | Age-standardized | Depressive disorders | Rate | 1999 | 547.7662058 | 747.4293447 | 381.8987362 |
| DALYs | Central Sub-Saharan Africa | Both | Age-standardized | Depressive disorders | Rate | 2000 | 1068.524455 | 1452.956306 | 724.4374427 |
| DALYs | East Asia | Both | Age-standardized | Depressive disorders | Rate | 2000 | 460.4725351 | 626.3444415 | 326.1527552 |
| DALYs | East Asia | Both | Age-standardized | Depressive disorders | Rate | 1999 | 466.4571302 | 632.7445086 | 330.1872211 |
| DALYs | High-income Asia Pacific | Both | Age-standardized | Depressive disorders | Rate | 1999 | 380.0453376 | 514.7569226 | 262.4484782 |
| DALYs | High-income North America | Both | Age-standardized | Depressive disorders | Rate | 1999 | 814.124468 | 1103.184585 | 573.2217999 |
| DALYs | Andean Latin America | Both | Age-standardized | Depressive disorders | Rate | 2001 | 473.3242361 | 644.0389548 | 324.2866558 |
| DALYs | High-income Asia Pacific | Both | Age-standardized | Depressive disorders | Rate | 2000 | 385.5504295 | 525.08313 | 266.7408522 |
| DALYs | Tropical Latin America | Both | Age-standardized | Depressive disorders | Rate | 2000 | 804.5107568 | 1081.847072 | 560.6463784 |
| DALYs | Eastern Europe | Both | Age-standardized | Depressive disorders | Rate | 2006 | 616.5626423 | 844.7157993 | 426.6872691 |
| DALYs | Central Europe | Both | Age-standardized | Depressive disorders | Rate | 1999 | 459.5953818 | 627.6685914 | 321.5309139 |
| DALYs | High-income North America | Both | Age-standardized | Depressive disorders | Rate | 2000 | 820.7862838 | 1108.929993 | 576.0189401 |
| DALYs | Caribbean | Both | Age-standardized | Depressive disorders | Rate | 2006 | 637.4788491 | 868.5611564 | 440.4712085 |
| DALYs | Western Europe | Both | Age-standardized | Depressive disorders | Rate | 2006 | 708.0856591 | 959.2123741 | 496.3690788 |
| DALYs | Global | Both | Age-standardized | Depressive disorders | Rate | 2001 | 620.3289557 | 846.289355 | 436.510358 |
| DALYs | Central Asia | Both | Age-standardized | Depressive disorders | Rate | 2006 | 566.9447741 | 772.9280802 | 389.6444859 |
| DALYs | Central Latin America | Both | Age-standardized | Depressive disorders | Rate | 2000 | 549.86292 | 750.0658224 | 383.6126528 |
| DALYs | Eastern Sub-Saharan Africa | Both | Age-standardized | Depressive disorders | Rate | 1999 | 932.3671604 | 1259.293758 | 646.0509265 |
| DALYs | South Asia | Both | Age-standardized | Depressive disorders | Rate | 2006 | 795.8489568 | 1085.070184 | 547.9080821 |
| DALYs | East Asia | Both | Age-standardized | Depressive disorders | Rate | 2001 | 457.8518379 | 621.4276747 | 324.2932763 |
| DALYs | Tropical Latin America | Both | Age-standardized | Depressive disorders | Rate | 2001 | 807.6403678 | 1082.134263 | 563.0492497 |
| DALYs | Western Sub-Saharan Africa | Both | Age-standardized | Depressive disorders | Rate | 1999 | 746.1207105 | 1012.229962 | 516.0369964 |
| DALYs | North Africa and Middle East | Both | Age-standardized | Depressive disorders | Rate | 2000 | 771.2919512 | 1043.105731 | 538.4279432 |
| DALYs | Central Europe | Both | Age-standardized | Depressive disorders | Rate | 2000 | 457.1523545 | 623.551165 | 318.7562602 |
| DALYs | High-income North America | Both | Age-standardized | Depressive disorders | Rate | 2001 | 820.1105532 | 1109.002698 | 577.9045631 |
| DALYs | North Africa and Middle East | Both | Age-standardized | Depressive disorders | Rate | 1999 | 772.065054 | 1045.405844 | 537.997518 |
| DALYs | Australasia | Both | Age-standardized | Depressive disorders | Rate | 2000 | 813.5035567 | 1089.946749 | 576.531231 |
| DALYs | East Asia | Both | Age-standardized | Depressive disorders | Rate | 2002 | 456.4497529 | 618.6989928 | 322.7131416 |
| DALYs | High-income Asia Pacific | Both | Age-standardized | Depressive disorders | Rate | 2001 | 388.1136051 | 528.0042294 | 268.9334355 |
| DALYs | Oceania | Both | Age-standardized | Depressive disorders | Rate | 2000 | 485.1521522 | 659.4748821 | 332.9395331 |
| DALYs | Central Latin America | Both | Age-standardized | Depressive disorders | Rate | 2001 | 551.1090291 | 750.6231779 | 383.804972 |
| DALYs | Western Sub-Saharan Africa | Both | Age-standardized | Depressive disorders | Rate | 2000 | 748.4369796 | 1015.955184 | 516.9888791 |
| DALYs | Eastern Sub-Saharan Africa | Both | Age-standardized | Depressive disorders | Rate | 2000 | 932.4576682 | 1257.187993 | 646.185996 |
| DALYs | Oceania | Both | Age-standardized | Depressive disorders | Rate | 2001 | 485.1777572 | 659.9477787 | 333.0568964 |
| DALYs | Tropical Latin America | Both | Age-standardized | Depressive disorders | Rate | 2002 | 810.2113473 | 1088.858594 | 565.0222341 |
| DALYs | High-income Asia Pacific | Both | Age-standardized | Depressive disorders | Rate | 2002 | 390.4617913 | 530.4890181 | 271.8278609 |
| DALYs | High-income North America | Both | Age-standardized | Depressive disorders | Rate | 2002 | 816.0681184 | 1102.575458 | 575.0070582 |
| DALYs | Southeast Asia | Both | Age-standardized | Depressive disorders | Rate | 2000 | 397.3036795 | 542.3156261 | 278.1891126 |
| DALYs | Central Europe | Both | Age-standardized | Depressive disorders | Rate | 2001 | 453.729044 | 618.581872 | 316.1853755 |
| DALYs | Western Europe | Both | Age-standardized | Depressive disorders | Rate | 2007 | 709.5082345 | 958.0535163 | 496.1252642 |
| DALYs | Central Asia | Both | Age-standardized | Depressive disorders | Rate | 2007 | 563.9453009 | 765.4932492 | 387.4140468 |
| DALYs | Eastern Europe | Both | Age-standardized | Depressive disorders | Rate | 2007 | 608.558667 | 833.6914101 | 421.597207 |
| DALYs | South Asia | Both | Age-standardized | Depressive disorders | Rate | 2007 | 762.1106989 | 1038.217829 | 526.5314956 |
| DALYs | Central Europe | Both | Age-standardized | Depressive disorders | Rate | 2002 | 448.3276349 | 609.9224312 | 312.4565504 |
| DALYs | Central Latin America | Both | Age-standardized | Depressive disorders | Rate | 2002 | 552.2739724 | 750.0755575 | 384.0006612 |
| DALYs | Caribbean | Both | Age-standardized | Depressive disorders | Rate | 2007 | 631.0156977 | 859.0992866 | 438.1865032 |
| DALYs | Oceania | Both | Age-standardized | Depressive disorders | Rate | 2002 | 485.6290845 | 662.7841346 | 333.660231 |
| DALYs | Southern Latin America | Both | Age-standardized | Depressive disorders | Rate | 2001 | 570.5153646 | 781.5012888 | 392.8395215 |
| DALYs | Australasia | Both | Age-standardized | Depressive disorders | Rate | 2001 | 822.5151993 | 1092.886725 | 586.8750515 |
| DALYs | High-income Asia Pacific | Both | Age-standardized | Depressive disorders | Rate | 2003 | 392.029468 | 533.4918908 | 274.1010698 |
| DALYs | North Africa and Middle East | Both | Age-standardized | Depressive disorders | Rate | 2001 | 772.808957 | 1045.943462 | 538.4427998 |
| DALYs | Central Latin America | Both | Age-standardized | Depressive disorders | Rate | 2003 | 553.1543797 | 752.4759153 | 384.6391946 |
| DALYs | Eastern Sub-Saharan Africa | Both | Age-standardized | Depressive disorders | Rate | 2001 | 931.0431352 | 1255.5605 | 644.7321404 |
| DALYs | Western Sub-Saharan Africa | Both | Age-standardized | Depressive disorders | Rate | 2001 | 749.6487249 | 1018.624832 | 518.7764053 |
| DALYs | Southeast Asia | Both | Age-standardized | Depressive disorders | Rate | 2001 | 396.8826851 | 541.3349581 | 277.8939107 |
| DALYs | Eastern Sub-Saharan Africa | Both | Age-standardized | Depressive disorders | Rate | 2002 | 929.2057926 | 1250.142867 | 643.8025602 |
| DALYs | Australasia | Both | Age-standardized | Depressive disorders | Rate | 2002 | 835.8750511 | 1117.88392 | 594.8754158 |
| DALYs | Andean Latin America | Both | Age-standardized | Depressive disorders | Rate | 2002 | 472.1357561 | 643.9955078 | 323.3413382 |
| DALYs | North Africa and Middle East | Both | Age-standardized | Depressive disorders | Rate | 2002 | 775.3508967 | 1051.490629 | 542.2676792 |
| DALYs | Central Europe | Both | Age-standardized | Depressive disorders | Rate | 2003 | 442.1699028 | 602.5548187 | 307.2612884 |
| DALYs | Oceania | Both | Age-standardized | Depressive disorders | Rate | 2003 | 485.8048724 | 662.6793657 | 335.257333 |
| DALYs | Central Sub-Saharan Africa | Both | Age-standardized | Depressive disorders | Rate | 2001 | 1067.293534 | 1454.402811 | 728.5719663 |
| DALYs | Western Sub-Saharan Africa | Both | Age-standardized | Depressive disorders | Rate | 2002 | 751.2018643 | 1019.570989 | 519.804984 |
| DALYs | North Africa and Middle East | Both | Age-standardized | Depressive disorders | Rate | 2003 | 778.5866438 | 1058.64444 | 544.0189692 |
| DALYs | Southern Sub-Saharan Africa | Both | Age-standardized | Depressive disorders | Rate | 2001 | 688.7431 | 924.5587742 | 482.667583 |
| DALYs | Central Sub-Saharan Africa | Both | Age-standardized | Depressive disorders | Rate | 2002 | 1065.833515 | 1451.213148 | 726.7939717 |
| DALYs | Global | Both | Age-standardized | Depressive disorders | Rate | 2002 | 621.0073405 | 847.9686921 | 436.8709248 |
| DALYs | Western Europe | Both | Age-standardized | Depressive disorders | Rate | 2008 | 712.1389492 | 960.2917845 | 497.5850724 |
| DALYs | Central Asia | Both | Age-standardized | Depressive disorders | Rate | 2008 | 560.45477 | 758.5633691 | 386.4018867 |
| DALYs | Eastern Europe | Both | Age-standardized | Depressive disorders | Rate | 2008 | 600.4856922 | 821.4554038 | 416.4342876 |
| DALYs | Southeast Asia | Both | Age-standardized | Depressive disorders | Rate | 2003 | 397.5361526 | 543.1992173 | 279.2477238 |
| DALYs | Caribbean | Both | Age-standardized | Depressive disorders | Rate | 2008 | 625.0243336 | 849.1861919 | 434.0728726 |
| DALYs | South Asia | Both | Age-standardized | Depressive disorders | Rate | 2008 | 721.9363276 | 980.7373401 | 500.3477673 |
| DALYs | Southeast Asia | Both | Age-standardized | Depressive disorders | Rate | 2002 | 397.1078992 | 541.5978313 | 279.1844518 |
| DALYs | Australasia | Both | Age-standardized | Depressive disorders | Rate | 2003 | 850.2873159 | 1130.569556 | 600.8735002 |
| DALYs | Southern Latin America | Both | Age-standardized | Depressive disorders | Rate | 2003 | 572.1218903 | 783.8912135 | 392.4462028 |
| DALYs | Southern Latin America | Both | Age-standardized | Depressive disorders | Rate | 2002 | 571.2251151 | 784.0888955 | 392.7178694 |
| DALYs | Oceania | Both | Age-standardized | Depressive disorders | Rate | 2004 | 486.1124801 | 664.6913281 | 334.7714435 |
| DALYs | Andean Latin America | Both | Age-standardized | Depressive disorders | Rate | 2003 | 471.0088195 | 643.6555088 | 323.3420492 |
| DALYs | Eastern Sub-Saharan Africa | Both | Age-standardized | Depressive disorders | Rate | 2003 | 927.0205882 | 1248.097304 | 641.1259654 |
| DALYs | Central Sub-Saharan Africa | Both | Age-standardized | Depressive disorders | Rate | 2003 | 1063.892075 | 1448.953244 | 726.4677626 |
| DALYs | Western Sub-Saharan Africa | Both | Age-standardized | Depressive disorders | Rate | 2003 | 752.8927375 | 1021.449276 | 522.2802806 |
| DALYs | Southern Sub-Saharan Africa | Both | Age-standardized | Depressive disorders | Rate | 2002 | 685.9433371 | 921.0304607 | 482.2558108 |
| DALYs | Australasia | Both | Age-standardized | Depressive disorders | Rate | 2004 | 861.5379944 | 1148.165268 | 607.523717 |
| DALYs | North Africa and Middle East | Both | Age-standardized | Depressive disorders | Rate | 2004 | 781.1757583 | 1063.167021 | 543.5816672 |
| DALYs | Eastern Sub-Saharan Africa | Both | Age-standardized | Depressive disorders | Rate | 2004 | 924.3100571 | 1243.190939 | 639.7210294 |
| DALYs | Global | Both | Age-standardized | Depressive disorders | Rate | 2003 | 621.7046554 | 848.4033935 | 437.3844235 |
| DALYs | Southern Latin America | Both | Age-standardized | Depressive disorders | Rate | 2004 | 572.6101212 | 782.5908557 | 394.848197 |
| DALYs | Southern Sub-Saharan Africa | Both | Age-standardized | Depressive disorders | Rate | 2003 | 682.7877845 | 915.3465961 | 481.3401215 |
| DALYs | Western Sub-Saharan Africa | Both | Age-standardized | Depressive disorders | Rate | 2004 | 754.207963 | 1023.818806 | 523.9886796 |
| DALYs | Andean Latin America | Both | Age-standardized | Depressive disorders | Rate | 2004 | 469.3214824 | 639.5457405 | 321.308815 |
| DALYs | Southeast Asia | Both | Age-standardized | Depressive disorders | Rate | 2004 | 397.9649867 | 542.3554645 | 279.7436405 |
| DALYs | East Asia | Both | Age-standardized | Depressive disorders | Rate | 2003 | 455.5138995 | 617.726455 | 322.8018118 |
| DALYs | Tropical Latin America | Both | Age-standardized | Depressive disorders | Rate | 2003 | 811.669677 | 1086.72477 | 565.3880153 |
| DALYs | Central Sub-Saharan Africa | Both | Age-standardized | Depressive disorders | Rate | 2004 | 1062.789546 | 1442.811113 | 727.5165565 |
| DALYs | High-income North America | Both | Age-standardized | Depressive disorders | Rate | 2003 | 810.9124484 | 1097.268059 | 570.7780707 |
| DALYs | Global | Both | Age-standardized | Depressive disorders | Rate | 2004 | 622.1750527 | 848.0190649 | 436.9688547 |
| DALYs | South Asia | Both | Age-standardized | Depressive disorders | Rate | 2009 | 687.8262163 | 929.5855282 | 477.4931673 |
| DALYs | Central Asia | Both | Age-standardized | Depressive disorders | Rate | 2009 | 557.662036 | 753.3601037 | 384.4399891 |
| DALYs | Southern Sub-Saharan Africa | Both | Age-standardized | Depressive disorders | Rate | 2004 | 680.3915758 | 912.695894 | 481.3596632 |
| DALYs | Eastern Europe | Both | Age-standardized | Depressive disorders | Rate | 2009 | 593.7426909 | 814.1897451 | 411.0861824 |
| DALYs | Caribbean | Both | Age-standardized | Depressive disorders | Rate | 2009 | 619.8754536 | 841.236824 | 431.1852985 |
| DALYs | Tropical Latin America | Both | Age-standardized | Depressive disorders | Rate | 2004 | 811.8588907 | 1083.905798 | 566.2313857 |
| DALYs | Western Europe | Both | Age-standardized | Depressive disorders | Rate | 2009 | 714.8777445 | 967.0961901 | 500.4413682 |
| DALYs | Southeast Asia | Both | Age-standardized | Depressive disorders | Rate | 2005 | 397.8553993 | 542.6124849 | 278.170937 |
| DALYs | East Asia | Both | Age-standardized | Depressive disorders | Rate | 2004 | 454.3786027 | 616.4171029 | 322.3615043 |
| DALYs | High-income Asia Pacific | Both | Age-standardized | Depressive disorders | Rate | 2004 | 393.15898 | 535.0815714 | 274.579248 |
| DALYs | Central Europe | Both | Age-standardized | Depressive disorders | Rate | 2004 | 436.4950526 | 594.566551 | 302.9898146 |
| DALYs | High-income North America | Both | Age-standardized | Depressive disorders | Rate | 2004 | 806.4045895 | 1091.664426 | 568.4726839 |
| DALYs | Central Latin America | Both | Age-standardized | Depressive disorders | Rate | 2004 | 554.3012466 | 751.479272 | 386.6759081 |
| DALYs | Global | Both | Age-standardized | Depressive disorders | Rate | 2005 | 621.7881373 | 847.4508971 | 436.994303 |
| DALYs | Central Europe | Both | Age-standardized | Depressive disorders | Rate | 2005 | 432.4514097 | 589.5801227 | 300.1591414 |
| DALYs | North Africa and Middle East | Both | Age-standardized | Depressive disorders | Rate | 2005 | 782.3440048 | 1063.613628 | 543.5545599 |
| DALYs | Central Sub-Saharan Africa | Both | Age-standardized | Depressive disorders | Rate | 2005 | 1061.317841 | 1435.76646 | 727.6150765 |
| DALYs | Oceania | Both | Age-standardized | Depressive disorders | Rate | 2005 | 485.9984469 | 666.6829708 | 335.4977177 |
| DALYs | Western Sub-Saharan Africa | Both | Age-standardized | Depressive disorders | Rate | 2005 | 754.5418782 | 1025.332554 | 524.776408 |
| DALYs | Eastern Sub-Saharan Africa | Both | Age-standardized | Depressive disorders | Rate | 2005 | 921.2685878 | 1240.33101 | 637.4226876 |
| DALYs | North Africa and Middle East | Both | Age-standardized | Depressive disorders | Rate | 2006 | 782.6657538 | 1064.917959 | 545.7378539 |
| DALYs | Central Europe | Both | Age-standardized | Depressive disorders | Rate | 2006 | 429.2789049 | 584.6575592 | 298.1118903 |
| DALYs | Western Sub-Saharan Africa | Both | Age-standardized | Depressive disorders | Rate | 2006 | 751.699652 | 1020.296658 | 523.121352 |
| DALYs | Southeast Asia | Both | Age-standardized | Depressive disorders | Rate | 2006 | 396.835436 | 540.6035415 | 278.0718509 |
| DALYs | Eastern Sub-Saharan Africa | Both | Age-standardized | Depressive disorders | Rate | 2006 | 916.5834648 | 1231.454352 | 633.1934934 |
| DALYs | Western Sub-Saharan Africa | Both | Age-standardized | Depressive disorders | Rate | 2007 | 744.7555067 | 1011.896162 | 519.5327103 |
| DALYs | East Asia | Both | Age-standardized | Depressive disorders | Rate | 2005 | 452.2846846 | 612.6469591 | 321.0612594 |
| DALYs | Central Europe | Both | Age-standardized | Depressive disorders | Rate | 2007 | 425.5338605 | 577.3197093 | 296.2374426 |
| DALYs | North Africa and Middle East | Both | Age-standardized | Depressive disorders | Rate | 2007 | 783.1353946 | 1063.01773 | 544.5675236 |
| DALYs | Eastern Sub-Saharan Africa | Both | Age-standardized | Depressive disorders | Rate | 2007 | 909.4331618 | 1222.393415 | 629.321477 |
| DALYs | Central Europe | Both | Age-standardized | Depressive disorders | Rate | 2008 | 422.1057393 | 570.9898123 | 294.4615645 |
| DALYs | Southeast Asia | Both | Age-standardized | Depressive disorders | Rate | 2007 | 395.2752992 | 536.6839763 | 276.9609658 |
| DALYs | North Africa and Middle East | Both | Age-standardized | Depressive disorders | Rate | 2008 | 783.2667928 | 1064.441264 | 543.9748628 |
| DALYs | Southeast Asia | Both | Age-standardized | Depressive disorders | Rate | 2008 | 393.6698822 | 533.9364316 | 276.3547263 |
| DALYs | Eastern Sub-Saharan Africa | Both | Age-standardized | Depressive disorders | Rate | 2008 | 901.231768 | 1212.277015 | 623.6864536 |
| DALYs | Western Sub-Saharan Africa | Both | Age-standardized | Depressive disorders | Rate | 2008 | 736.0865259 | 998.4358463 | 515.6335687 |
| DALYs | Central Europe | Both | Age-standardized | Depressive disorders | Rate | 2009 | 419.2198674 | 563.6837192 | 293.4215757 |
| DALYs | Eastern Sub-Saharan Africa | Both | Age-standardized | Depressive disorders | Rate | 2009 | 893.7168672 | 1203.412196 | 619.2680831 |
| DALYs | North Africa and Middle East | Both | Age-standardized | Depressive disorders | Rate | 2009 | 783.5063282 | 1061.650321 | 544.698333 |
| DALYs | Southeast Asia | Both | Age-standardized | Depressive disorders | Rate | 2009 | 392.39171 | 532.5969514 | 274.7869706 |
| DALYs | Western Sub-Saharan Africa | Both | Age-standardized | Depressive disorders | Rate | 2009 | 727.9978865 | 987.3518067 | 511.700816 |
| DALYs | Tropical Latin America | Both | Age-standardized | Depressive disorders | Rate | 2005 | 810.0933431 | 1086.083332 | 566.3793756 |
| DALYs | Central Latin America | Both | Age-standardized | Depressive disorders | Rate | 2005 | 555.1978675 | 752.6493072 | 387.8150103 |
| DALYs | Southeast Asia | Both | Age-standardized | Depressive disorders | Rate | 2010 | 391.5438749 | 529.0292546 | 273.9836755 |
| DALYs | High-income North America | Both | Age-standardized | Depressive disorders | Rate | 2005 | 804.524469 | 1091.739741 | 567.730276 |
| DALYs | North Africa and Middle East | Both | Age-standardized | Depressive disorders | Rate | 2010 | 783.8001829 | 1063.951941 | 544.4767358 |
| DALYs | Eastern Sub-Saharan Africa | Both | Age-standardized | Depressive disorders | Rate | 2010 | 888.0430038 | 1197.255299 | 617.979213 |
| DALYs | Western Sub-Saharan Africa | Both | Age-standardized | Depressive disorders | Rate | 2010 | 722.5508498 | 980.7910505 | 507.7017742 |
| DALYs | Central Europe | Both | Age-standardized | Depressive disorders | Rate | 2010 | 417.4943243 | 561.6167884 | 291.9376926 |
| DALYs | Central Europe | Both | Age-standardized | Depressive disorders | Rate | 2011 | 416.2751162 | 561.420927 | 290.3184438 |
| DALYs | Southeast Asia | Both | Age-standardized | Depressive disorders | Rate | 2011 | 391.7112066 | 530.3718617 | 274.4016932 |
| DALYs | North Africa and Middle East | Both | Age-standardized | Depressive disorders | Rate | 2011 | 785.7551978 | 1066.893185 | 542.9292285 |
| DALYs | Western Sub-Saharan Africa | Both | Age-standardized | Depressive disorders | Rate | 2011 | 718.7299737 | 977.7837353 | 504.262132 |
| DALYs | Eastern Sub-Saharan Africa | Both | Age-standardized | Depressive disorders | Rate | 2011 | 883.7487923 | 1194.396842 | 613.5898108 |
| DALYs | Australasia | Both | Age-standardized | Depressive disorders | Rate | 2005 | 867.1707796 | 1155.131555 | 609.020897 |
| DALYs | Southern Latin America | Both | Age-standardized | Depressive disorders | Rate | 2005 | 572.2023255 | 781.5535224 | 395.7599445 |
| DALYs | Andean Latin America | Both | Age-standardized | Depressive disorders | Rate | 2005 | 467.7789014 | 636.2770789 | 320.7642641 |
| DALYs | Southern Sub-Saharan Africa | Both | Age-standardized | Depressive disorders | Rate | 2005 | 679.4406814 | 911.0802222 | 480.1197761 |
| DALYs | High-income Asia Pacific | Both | Age-standardized | Depressive disorders | Rate | 2005 | 393.4665484 | 535.6606596 | 275.4317801 |
| DALYs | Southern Sub-Saharan Africa | Both | Age-standardized | Depressive disorders | Rate | 2006 | 680.3985035 | 911.9057005 | 480.9454361 |
| DALYs | Central Sub-Saharan Africa | Both | Age-standardized | Depressive disorders | Rate | 2006 | 1058.775108 | 1437.194875 | 726.8824764 |
| DALYs | Southern Latin America | Both | Age-standardized | Depressive disorders | Rate | 2006 | 566.9141046 | 771.709808 | 391.9621569 |
| DALYs | Andean Latin America | Both | Age-standardized | Depressive disorders | Rate | 2006 | 464.8458739 | 633.053711 | 320.4244221 |
| DALYs | Tropical Latin America | Both | Age-standardized | Depressive disorders | Rate | 2006 | 795.6204228 | 1067.068859 | 556.379672 |
| DALYs | Global | Both | Age-standardized | Depressive disorders | Rate | 2006 | 617.5854463 | 841.6041097 | 434.7125187 |
| DALYs | High-income North America | Both | Age-standardized | Depressive disorders | Rate | 2006 | 805.1796453 | 1095.251976 | 568.4084675 |
| DALYs | Central Latin America | Both | Age-standardized | Depressive disorders | Rate | 2006 | 556.8451403 | 754.4100047 | 388.9003032 |
| DALYs | High-income Asia Pacific | Both | Age-standardized | Depressive disorders | Rate | 2006 | 393.0314867 | 534.4094533 | 275.3733543 |
| DALYs | East Asia | Both | Age-standardized | Depressive disorders | Rate | 2006 | 448.0662185 | 604.6364956 | 317.5901124 |
| DALYs | Oceania | Both | Age-standardized | Depressive disorders | Rate | 2006 | 484.9878175 | 662.3751391 | 333.9532386 |
| DALYs | Global | Both | Age-standardized | Depressive disorders | Rate | 2007 | 608.5304781 | 828.70662 | 428.0885304 |
| DALYs | High-income North America | Both | Age-standardized | Depressive disorders | Rate | 2007 | 806.5690258 | 1098.902479 | 569.9155341 |
| DALYs | Australasia | Both | Age-standardized | Depressive disorders | Rate | 2006 | 866.1006589 | 1162.397043 | 608.4377308 |
| DALYs | East Asia | Both | Age-standardized | Depressive disorders | Rate | 2007 | 441.6385968 | 595.8258138 | 313.6294568 |
| DALYs | Tropical Latin America | Both | Age-standardized | Depressive disorders | Rate | 2007 | 764.1553242 | 1025.417975 | 535.1363737 |
| DALYs | Central Latin America | Both | Age-standardized | Depressive disorders | Rate | 2007 | 559.8140178 | 761.199897 | 390.4408281 |
| DALYs | Tropical Latin America | Both | Age-standardized | Depressive disorders | Rate | 2008 | 726.4162913 | 974.8398593 | 512.3671185 |
| DALYs | High-income Asia Pacific | Both | Age-standardized | Depressive disorders | Rate | 2007 | 392.094475 | 531.7590028 | 274.5708463 |
| DALYs | High-income North America | Both | Age-standardized | Depressive disorders | Rate | 2008 | 808.2721158 | 1102.108827 | 570.6831856 |
| DALYs | East Asia | Both | Age-standardized | Depressive disorders | Rate | 2008 | 434.7992251 | 584.4948664 | 309.0998651 |
| DALYs | Central Latin America | Both | Age-standardized | Depressive disorders | Rate | 2008 | 563.1296842 | 763.3753642 | 393.0895673 |
| DALYs | Southeast Asia | Both | Age-standardized | Depressive disorders | Rate | 2012 | 392.6297301 | 532.0915902 | 274.4576127 |
| DALYs | Oceania | Both | Age-standardized | Depressive disorders | Rate | 2007 | 482.8064489 | 658.2860561 | 334.9665482 |
| DALYs | Central Europe | Both | Age-standardized | Depressive disorders | Rate | 2012 | 415.3577344 | 561.2426875 | 289.6917753 |
| DALYs | High-income Asia Pacific | Both | Age-standardized | Depressive disorders | Rate | 2008 | 390.7588063 | 532.4007874 | 274.9833144 |
| DALYs | Australasia | Both | Age-standardized | Depressive disorders | Rate | 2007 | 861.2418633 | 1151.01888 | 607.3721058 |
| DALYs | Oceania | Both | Age-standardized | Depressive disorders | Rate | 2008 | 480.4402967 | 654.671396 | 333.1635248 |
| DALYs | Western Sub-Saharan Africa | Both | Age-standardized | Depressive disorders | Rate | 2012 | 714.5830154 | 974.9104816 | 500.0462147 |
| DALYs | North Africa and Middle East | Both | Age-standardized | Depressive disorders | Rate | 2012 | 790.4488013 | 1073.498507 | 547.3332482 |
| DALYs | Southern Latin America | Both | Age-standardized | Depressive disorders | Rate | 2007 | 555.845298 | 754.0336926 | 384.6068089 |
| DALYs | Eastern Sub-Saharan Africa | Both | Age-standardized | Depressive disorders | Rate | 2012 | 879.2626602 | 1188.450045 | 609.8985296 |
| DALYs | Andean Latin America | Both | Age-standardized | Depressive disorders | Rate | 2007 | 460.0938477 | 629.3488695 | 315.2503144 |
| DALYs | Southern Sub-Saharan Africa | Both | Age-standardized | Depressive disorders | Rate | 2007 | 682.948584 | 912.7897227 | 480.918076 |
| DALYs | Australasia | Both | Age-standardized | Depressive disorders | Rate | 2008 | 855.2335768 | 1143.809363 | 601.1505758 |
| DALYs | Central Europe | Both | Age-standardized | Depressive disorders | Rate | 2013 | 414.7194406 | 564.0779865 | 289.2392898 |
| DALYs | Southern Latin America | Both | Age-standardized | Depressive disorders | Rate | 2008 | 543.3923206 | 736.4430608 | 378.7720393 |
| DALYs | Central Sub-Saharan Africa | Both | Age-standardized | Depressive disorders | Rate | 2007 | 1055.769682 | 1435.483051 | 719.1659974 |
| DALYs | High-income Asia Pacific | Both | Age-standardized | Depressive disorders | Rate | 2009 | 389.7383652 | 529.2112547 | 273.5979534 |
| DALYs | Global | Both | Age-standardized | Depressive disorders | Rate | 2008 | 597.8551348 | 813.4093096 | 420.8558561 |
| DALYs | Southeast Asia | Both | Age-standardized | Depressive disorders | Rate | 2013 | 394.0882355 | 534.5338535 | 274.9225086 |
| DALYs | Central Sub-Saharan Africa | Both | Age-standardized | Depressive disorders | Rate | 2008 | 1052.7518 | 1426.444702 | 717.7831367 |
| DALYs | Central Asia | Both | Age-standardized | Depressive disorders | Rate | 2010 | 555.8121714 | 752.132123 | 383.5924786 |
| DALYs | North Africa and Middle East | Both | Age-standardized | Depressive disorders | Rate | 2013 | 796.0163569 | 1084.405189 | 549.1113993 |
| DALYs | Eastern Sub-Saharan Africa | Both | Age-standardized | Depressive disorders | Rate | 2013 | 875.3451065 | 1182.385951 | 606.6736622 |
| DALYs | Southern Sub-Saharan Africa | Both | Age-standardized | Depressive disorders | Rate | 2008 | 686.3807816 | 919.5701835 | 483.3709682 |
| DALYs | Western Sub-Saharan Africa | Both | Age-standardized | Depressive disorders | Rate | 2013 | 710.6766643 | 968.0565511 | 496.2055903 |
| DALYs | Oceania | Both | Age-standardized | Depressive disorders | Rate | 2009 | 478.454345 | 652.192971 | 329.7333611 |
| DALYs | Eastern Europe | Both | Age-standardized | Depressive disorders | Rate | 2010 | 589.6580292 | 807.3991621 | 409.2122112 |
| DALYs | South Asia | Both | Age-standardized | Depressive disorders | Rate | 2010 | 672.3353237 | 907.4939726 | 464.8860155 |
| DALYs | Australasia | Both | Age-standardized | Depressive disorders | Rate | 2009 | 849.1707934 | 1138.898649 | 597.6304424 |
| DALYs | Caribbean | Both | Age-standardized | Depressive disorders | Rate | 2010 | 617.2938197 | 833.8587827 | 426.0311925 |
| DALYs | Western Europe | Both | Age-standardized | Depressive disorders | Rate | 2010 | 716.82599 | 971.8479264 | 500.8156285 |
| DALYs | Oceania | Both | Age-standardized | Depressive disorders | Rate | 2010 | 477.2431064 | 647.2695515 | 328.4319322 |
| DALYs | Andean Latin America | Both | Age-standardized | Depressive disorders | Rate | 2008 | 455.7746984 | 621.4217853 | 315.0637729 |
| DALYs | Central Europe | Both | Age-standardized | Depressive disorders | Rate | 2014 | 414.3324181 | 563.6743251 | 289.3356617 |
| DALYs | Southern Sub-Saharan Africa | Both | Age-standardized | Depressive disorders | Rate | 2009 | 689.9260155 | 924.0891996 | 485.6182554 |
| DALYs | Southeast Asia | Both | Age-standardized | Depressive disorders | Rate | 2014 | 395.4087651 | 536.6543008 | 275.9223791 |
| DALYs | Andean Latin America | Both | Age-standardized | Depressive disorders | Rate | 2009 | 451.7728341 | 614.7034641 | 313.7537796 |
| DALYs | North Africa and Middle East | Both | Age-standardized | Depressive disorders | Rate | 2014 | 800.6393782 | 1093.712335 | 550.5920099 |
| DALYs | Australasia | Both | Age-standardized | Depressive disorders | Rate | 2010 | 842.8176805 | 1131.506555 | 589.235327 |
| DALYs | Southern Latin America | Both | Age-standardized | Depressive disorders | Rate | 2009 | 532.7730383 | 716.2069772 | 374.0870187 |
| DALYs | Eastern Sub-Saharan Africa | Both | Age-standardized | Depressive disorders | Rate | 2014 | 872.5851927 | 1181.757748 | 604.9183343 |
| DALYs | Western Sub-Saharan Africa | Both | Age-standardized | Depressive disorders | Rate | 2014 | 707.8491152 | 964.375554 | 492.6500454 |
| DALYs | High-income North America | Both | Age-standardized | Depressive disorders | Rate | 2009 | 809.471942 | 1104.269883 | 571.9773447 |
| DALYs | Central Sub-Saharan Africa | Both | Age-standardized | Depressive disorders | Rate | 2009 | 1050.282693 | 1431.830655 | 714.5942711 |
| DALYs | Tropical Latin America | Both | Age-standardized | Depressive disorders | Rate | 2009 | 692.0672023 | 926.8787448 | 492.3031302 |
| DALYs | Global | Both | Age-standardized | Depressive disorders | Rate | 2009 | 588.8957813 | 799.6600887 | 414.1114054 |
| DALYs | Central Latin America | Both | Age-standardized | Depressive disorders | Rate | 2009 | 565.827309 | 768.634909 | 393.6570222 |
| DALYs | Central Asia | Both | Age-standardized | Depressive disorders | Rate | 2011 | 555.439152 | 751.9176957 | 383.195761 |
| DALYs | Southern Latin America | Both | Age-standardized | Depressive disorders | Rate | 2010 | 527.6977807 | 705.1324041 | 370.6805847 |
| DALYs | Central Sub-Saharan Africa | Both | Age-standardized | Depressive disorders | Rate | 2010 | 1049.375106 | 1423.814751 | 710.4820037 |
| DALYs | East Asia | Both | Age-standardized | Depressive disorders | Rate | 2009 | 429.3148459 | 575.979165 | 303.903496 |
| DALYs | Andean Latin America | Both | Age-standardized | Depressive disorders | Rate | 2010 | 449.9160363 | 610.8196402 | 309.7371533 |
| DALYs | Caribbean | Both | Age-standardized | Depressive disorders | Rate | 2011 | 616.5389962 | 832.0865753 | 427.3045858 |
| DALYs | Eastern Europe | Both | Age-standardized | Depressive disorders | Rate | 2011 | 588.7297315 | 806.5582233 | 408.8093595 |
| DALYs | Central Europe | Both | Age-standardized | Depressive disorders | Rate | 2015 | 414.1295455 | 565.4516888 | 288.1816571 |
| DALYs | Southern Sub-Saharan Africa | Both | Age-standardized | Depressive disorders | Rate | 2010 | 693.1802769 | 927.4155799 | 486.4868126 |
| DALYs | Global | Both | Age-standardized | Depressive disorders | Rate | 2010 | 584.9768761 | 793.8728942 | 411.5512895 |
| DALYs | Southeast Asia | Both | Age-standardized | Depressive disorders | Rate | 2015 | 396.5806772 | 539.1272175 | 276.5057627 |
| DALYs | North Africa and Middle East | Both | Age-standardized | Depressive disorders | Rate | 2015 | 802.7586393 | 1100.660054 | 548.4785334 |
| DALYs | Australasia | Both | Age-standardized | Depressive disorders | Rate | 2011 | 838.722607 | 1131.846303 | 589.9002697 |
| DALYs | Western Sub-Saharan Africa | Both | Age-standardized | Depressive disorders | Rate | 2015 | 707.0700484 | 963.4716236 | 491.8824916 |
| DALYs | Western Europe | Both | Age-standardized | Depressive disorders | Rate | 2011 | 717.2516989 | 973.1330966 | 502.057305 |
| DALYs | Eastern Europe | Both | Age-standardized | Depressive disorders | Rate | 2012 | 589.6029802 | 810.0787752 | 408.7288627 |
| DALYs | Central Asia | Both | Age-standardized | Depressive disorders | Rate | 2012 | 555.6428836 | 754.7348956 | 385.2536638 |
| DALYs | Central Latin America | Both | Age-standardized | Depressive disorders | Rate | 2010 | 567.5338056 | 771.274292 | 394.7655589 |
| DALYs | Eastern Sub-Saharan Africa | Both | Age-standardized | Depressive disorders | Rate | 2015 | 871.6865921 | 1181.32018 | 603.4695989 |
| DALYs | Tropical Latin America | Both | Age-standardized | Depressive disorders | Rate | 2010 | 671.9262794 | 900.6737076 | 481.0161372 |
| DALYs | South Asia | Both | Age-standardized | Depressive disorders | Rate | 2011 | 670.5884139 | 907.2130086 | 464.3931702 |
| DALYs | Central Sub-Saharan Africa | Both | Age-standardized | Depressive disorders | Rate | 2011 | 1049.155561 | 1423.000596 | 714.6648282 |
| DALYs | Caribbean | Both | Age-standardized | Depressive disorders | Rate | 2012 | 616.3806486 | 828.1622301 | 424.2849658 |
| DALYs | East Asia | Both | Age-standardized | Depressive disorders | Rate | 2010 | 426.8379748 | 571.111792 | 302.794609 |
| DALYs | High-income Asia Pacific | Both | Age-standardized | Depressive disorders | Rate | 2010 | 388.895652 | 525.7787881 | 273.446633 |
| DALYs | High-income North America | Both | Age-standardized | Depressive disorders | Rate | 2010 | 809.8123291 | 1104.570825 | 571.0825304 |
| DALYs | Southern Sub-Saharan Africa | Both | Age-standardized | Depressive disorders | Rate | 2011 | 697.8141424 | 936.2748604 | 489.993141 |
| DALYs | Southern Latin America | Both | Age-standardized | Depressive disorders | Rate | 2011 | 527.9940227 | 708.8760598 | 367.6108919 |
| DALYs | Andean Latin America | Both | Age-standardized | Depressive disorders | Rate | 2011 | 449.3492456 | 611.935176 | 309.6704752 |
| DALYs | Western Europe | Both | Age-standardized | Depressive disorders | Rate | 2012 | 716.3505498 | 971.3165918 | 501.1284427 |
| DALYs | Global | Both | Age-standardized | Depressive disorders | Rate | 2011 | 585.1420352 | 794.7531319 | 411.3472012 |
| DALYs | South Asia | Both | Age-standardized | Depressive disorders | Rate | 2012 | 669.7760244 | 906.5743452 | 463.8081119 |
| DALYs | Western Europe | Both | Age-standardized | Depressive disorders | Rate | 2013 | 715.0175882 | 973.5666807 | 498.9182136 |
| DALYs | Tropical Latin America | Both | Age-standardized | Depressive disorders | Rate | 2011 | 662.7193099 | 887.1561605 | 474.0904242 |
| DALYs | High-income Asia Pacific | Both | Age-standardized | Depressive disorders | Rate | 2011 | 388.0725286 | 526.9027758 | 272.1043849 |
| DALYs | Southeast Asia | Both | Age-standardized | Depressive disorders | Rate | 2016 | 397.9163004 | 540.8898111 | 277.7092137 |
| DALYs | Central Latin America | Both | Age-standardized | Depressive disorders | Rate | 2011 | 567.918273 | 772.3996604 | 396.1423196 |
| DALYs | Eastern Sub-Saharan Africa | Both | Age-standardized | Depressive disorders | Rate | 2016 | 872.521406 | 1181.851514 | 603.9625635 |
| DALYs | South Asia | Both | Age-standardized | Depressive disorders | Rate | 2013 | 669.5313504 | 907.5057541 | 463.6704919 |
| DALYs | Western Sub-Saharan Africa | Both | Age-standardized | Depressive disorders | Rate | 2016 | 707.5558411 | 960.1793023 | 490.8343575 |
| DALYs | High-income North America | Both | Age-standardized | Depressive disorders | Rate | 2011 | 806.6376593 | 1100.198335 | 571.3885448 |
| DALYs | East Asia | Both | Age-standardized | Depressive disorders | Rate | 2011 | 427.3067046 | 573.9162898 | 303.2942799 |
| DALYs | Southern Latin America | Both | Age-standardized | Depressive disorders | Rate | 2012 | 529.012152 | 711.7061038 | 369.0648581 |
| DALYs | Oceania | Both | Age-standardized | Depressive disorders | Rate | 2011 | 477.3290592 | 646.3915697 | 328.3260962 |
| DALYs | Eastern Europe | Both | Age-standardized | Depressive disorders | Rate | 2013 | 591.3471388 | 812.6945488 | 409.0508499 |
| DALYs | Andean Latin America | Both | Age-standardized | Depressive disorders | Rate | 2012 | 448.9665684 | 613.7310398 | 306.6172398 |
| DALYs | Central Sub-Saharan Africa | Both | Age-standardized | Depressive disorders | Rate | 2012 | 1049.633327 | 1423.522376 | 715.9191753 |
| DALYs | Caribbean | Both | Age-standardized | Depressive disorders | Rate | 2013 | 616.364669 | 829.3930061 | 424.2201154 |
| DALYs | Central Asia | Both | Age-standardized | Depressive disorders | Rate | 2013 | 556.6024991 | 754.7390091 | 383.5090478 |
| DALYs | Global | Both | Age-standardized | Depressive disorders | Rate | 2012 | 585.9406793 | 796.6699989 | 411.7012382 |
| DALYs | Southern Sub-Saharan Africa | Both | Age-standardized | Depressive disorders | Rate | 2012 | 704.636503 | 946.1607705 | 497.2639018 |
| DALYs | Western Europe | Both | Age-standardized | Depressive disorders | Rate | 2014 | 713.9368392 | 973.6076472 | 499.2010396 |
| DALYs | Tropical Latin America | Both | Age-standardized | Depressive disorders | Rate | 2012 | 654.4859518 | 877.6258308 | 467.7031134 |
| DALYs | East Asia | Both | Age-standardized | Depressive disorders | Rate | 2012 | 429.1240925 | 575.5381811 | 304.6243146 |
| DALYs | Central Latin America | Both | Age-standardized | Depressive disorders | Rate | 2012 | 568.0619884 | 775.1768152 | 396.2300973 |
| DALYs | South Asia | Both | Age-standardized | Depressive disorders | Rate | 2014 | 669.6721156 | 907.7269181 | 464.2293393 |
| DALYs | High-income North America | Both | Age-standardized | Depressive disorders | Rate | 2012 | 799.1421736 | 1088.512819 | 566.3252159 |
| DALYs | Global | Both | Age-standardized | Depressive disorders | Rate | 2013 | 587.1321154 | 799.3484025 | 412.3877007 |
| DALYs | High-income Asia Pacific | Both | Age-standardized | Depressive disorders | Rate | 2012 | 386.8581981 | 527.0447926 | 271.4732397 |
| DALYs | Eastern Europe | Both | Age-standardized | Depressive disorders | Rate | 2014 | 593.0314537 | 813.8495172 | 410.3493976 |
| DALYs | Western Europe | Both | Age-standardized | Depressive disorders | Rate | 2015 | 714.017991 | 973.5321722 | 499.9774231 |
| DALYs | Central Asia | Both | Age-standardized | Depressive disorders | Rate | 2014 | 557.2826984 | 756.5527773 | 386.5524908 |
| DALYs | Caribbean | Both | Age-standardized | Depressive disorders | Rate | 2014 | 616.4913968 | 833.7623552 | 422.0860663 |
| DALYs | Tropical Latin America | Both | Age-standardized | Depressive disorders | Rate | 2013 | 647.2367271 | 869.9379066 | 462.3979079 |
| DALYs | High-income North America | Both | Age-standardized | Depressive disorders | Rate | 2013 | 790.1398904 | 1073.351235 | 559.1252652 |
| DALYs | Central Latin America | Both | Age-standardized | Depressive disorders | Rate | 2013 | 567.9367497 | 775.5850267 | 394.6766337 |
| DALYs | North Africa and Middle East | Both | Age-standardized | Depressive disorders | Rate | 2016 | 801.9723914 | 1098.360276 | 546.9630631 |
| DALYs | Central Europe | Both | Age-standardized | Depressive disorders | Rate | 2016 | 415.9039032 | 567.7644109 | 289.992297 |
| DALYs | Central Asia | Both | Age-standardized | Depressive disorders | Rate | 2015 | 557.5670564 | 755.7387148 | 385.768784 |
| DALYs | East Asia | Both | Age-standardized | Depressive disorders | Rate | 2013 | 431.4681116 | 579.9429517 | 305.1765707 |
| DALYs | South Asia | Both | Age-standardized | Depressive disorders | Rate | 2015 | 669.7481324 | 908.8851254 | 463.9868675 |
| DALYs | Eastern Europe | Both | Age-standardized | Depressive disorders | Rate | 2015 | 593.7371044 | 816.9556264 | 410.0172363 |
| DALYs | High-income Asia Pacific | Both | Age-standardized | Depressive disorders | Rate | 2013 | 385.3186633 | 525.1547608 | 269.790104 |
| DALYs | Oceania | Both | Age-standardized | Depressive disorders | Rate | 2012 | 477.4790722 | 646.2751664 | 328.7084395 |
| DALYs | Caribbean | Both | Age-standardized | Depressive disorders | Rate | 2015 | 617.1610804 | 835.665604 | 423.5904353 |
| DALYs | South Asia | Both | Age-standardized | Depressive disorders | Rate | 2016 | 669.5785829 | 912.1373569 | 463.8987331 |
| DALYs | Central Asia | Both | Age-standardized | Depressive disorders | Rate | 2016 | 557.9667684 | 755.4415695 | 384.2496856 |
| DALYs | Australasia | Both | Age-standardized | Depressive disorders | Rate | 2012 | 834.616548 | 1137.188145 | 585.78296 |
| DALYs | Eastern Europe | Both | Age-standardized | Depressive disorders | Rate | 2016 | 592.9308095 | 814.0495805 | 408.7063704 |
| DALYs | Caribbean | Both | Age-standardized | Depressive disorders | Rate | 2016 | 618.0892421 | 838.8946129 | 423.1646965 |
| DALYs | Western Sub-Saharan Africa | Both | Age-standardized | Depressive disorders | Rate | 2017 | 707.9910528 | 960.7671049 | 488.9334359 |
| DALYs | Oceania | Both | Age-standardized | Depressive disorders | Rate | 2013 | 477.8169567 | 648.3688376 | 329.9411199 |
| DALYs | Eastern Sub-Saharan Africa | Both | Age-standardized | Depressive disorders | Rate | 2017 | 874.0995041 | 1181.362972 | 603.4079754 |
| DALYs | North Africa and Middle East | Both | Age-standardized | Depressive disorders | Rate | 2017 | 800.0834314 | 1096.56307 | 541.6057678 |
| DALYs | Central Europe | Both | Age-standardized | Depressive disorders | Rate | 2017 | 419.9657365 | 573.975536 | 291.961866 |
| DALYs | Western Europe | Both | Age-standardized | Depressive disorders | Rate | 2016 | 715.4838682 | 975.0619911 | 499.2872124 |
| DALYs | Eastern Europe | Both | Age-standardized | Depressive disorders | Rate | 2017 | 591.6120284 | 813.7931187 | 406.5354025 |
| DALYs | Central Asia | Both | Age-standardized | Depressive disorders | Rate | 2017 | 558.5866431 | 755.2081095 | 384.7115661 |
| DALYs | Southeast Asia | Both | Age-standardized | Depressive disorders | Rate | 2017 | 399.7765569 | 544.3392472 | 279.2197365 |
| DALYs | Central Latin America | Both | Age-standardized | Depressive disorders | Rate | 2014 | 567.7471648 | 775.4665946 | 395.070549 |
| DALYs | Central Europe | Both | Age-standardized | Depressive disorders | Rate | 2018 | 424.2883324 | 582.5991306 | 294.4832012 |
| DALYs | Eastern Europe | Both | Age-standardized | Depressive disorders | Rate | 2018 | 590.1573777 | 811.3857229 | 405.6396518 |
| DALYs | Caribbean | Both | Age-standardized | Depressive disorders | Rate | 2017 | 619.2912473 | 844.9897468 | 424.3221261 |
| DALYs | East Asia | Both | Age-standardized | Depressive disorders | Rate | 2014 | 433.5947922 | 583.3238156 | 306.4889036 |
| DALYs | Central Asia | Both | Age-standardized | Depressive disorders | Rate | 2018 | 559.2269578 | 756.1179944 | 386.3329487 |
| DALYs | High-income North America | Both | Age-standardized | Depressive disorders | Rate | 2014 | 782.0810742 | 1059.025588 | 553.3946357 |
| DALYs | Australasia | Both | Age-standardized | Depressive disorders | Rate | 2013 | 830.5365335 | 1131.661735 | 579.5982428 |
| DALYs | North Africa and Middle East | Both | Age-standardized | Depressive disorders | Rate | 2018 | 797.3395876 | 1093.550957 | 538.8687314 |
| DALYs | High-income Asia Pacific | Both | Age-standardized | Depressive disorders | Rate | 2014 | 384.1978784 | 522.7150583 | 269.1550029 |
| DALYs | Western Sub-Saharan Africa | Both | Age-standardized | Depressive disorders | Rate | 2018 | 708.3635942 | 961.1968209 | 487.2284794 |
| DALYs | Caribbean | Both | Age-standardized | Depressive disorders | Rate | 2018 | 620.4099894 | 853.5044625 | 420.7185909 |
| DALYs | Eastern Sub-Saharan Africa | Both | Age-standardized | Depressive disorders | Rate | 2018 | 875.5380157 | 1180.25301 | 601.9211849 |
| DALYs | Southern Sub-Saharan Africa | Both | Age-standardized | Depressive disorders | Rate | 2013 | 711.8353325 | 958.8215786 | 497.9375903 |
| DALYs | North Africa and Middle East | Both | Age-standardized | Depressive disorders | Rate | 2019 | 794.6022749 | 1091.493424 | 535.7846976 |
| DALYs | Western Europe | Both | Age-standardized | Depressive disorders | Rate | 2017 | 717.256741 | 977.6433195 | 501.7603203 |
| DALYs | Western Europe | Both | Age-standardized | Depressive disorders | Rate | 2018 | 718.4441696 | 978.7197169 | 502.7852565 |
| DALYs | South Asia | Both | Age-standardized | Depressive disorders | Rate | 2017 | 669.7036445 | 912.1168941 | 465.8405494 |
| DALYs | Central Europe | Both | Age-standardized | Depressive disorders | Rate | 2019 | 426.5764482 | 585.7033429 | 293.9064135 |
| DALYs | Southern Latin America | Both | Age-standardized | Depressive disorders | Rate | 2013 | 530.4222815 | 709.4886528 | 369.5903007 |
| DALYs | Central Sub-Saharan Africa | Both | Age-standardized | Depressive disorders | Rate | 2013 | 1050.357928 | 1431.677017 | 714.6750261 |
| DALYs | Caribbean | Both | Age-standardized | Depressive disorders | Rate | 2019 | 621.5952103 | 856.7058091 | 420.9310583 |
| DALYs | Andean Latin America | Both | Age-standardized | Depressive disorders | Rate | 2013 | 448.6358223 | 612.5409414 | 305.6605927 |
| DALYs | South Asia | Both | Age-standardized | Depressive disorders | Rate | 2018 | 669.595316 | 911.2396528 | 466.3509212 |
| DALYs | Oceania | Both | Age-standardized | Depressive disorders | Rate | 2014 | 478.1253437 | 649.6230212 | 330.2993901 |
| DALYs | Southeast Asia | Both | Age-standardized | Depressive disorders | Rate | 2018 | 401.4416032 | 547.4453247 | 279.4450453 |
| DALYs | Western Europe | Both | Age-standardized | Depressive disorders | Rate | 2019 | 717.9949186 | 978.8096671 | 501.0821716 |
| DALYs | Southeast Asia | Both | Age-standardized | Depressive disorders | Rate | 2019 | 402.3256691 | 546.7829567 | 279.2265327 |
| DALYs | Western Sub-Saharan Africa | Both | Age-standardized | Depressive disorders | Rate | 2019 | 708.6904225 | 963.3103962 | 486.5971259 |
| DALYs | South Asia | Both | Age-standardized | Depressive disorders | Rate | 2019 | 669.7361674 | 910.6654877 | 466.8191272 |
| DALYs | Central Europe | Both | Age-standardized | Depressive disorders | Rate | 2020 | 488.7644826 | 665.7497315 | 337.062543 |
| DALYs | Western Europe | Both | Age-standardized | Depressive disorders | Rate | 2020 | 857.657335 | 1170.368091 | 600.487341 |
| DALYs | Eastern Europe | Both | Age-standardized | Depressive disorders | Rate | 2019 | 588.6550462 | 808.7135988 | 404.1289578 |
| DALYs | Central Asia | Both | Age-standardized | Depressive disorders | Rate | 2019 | 559.7518668 | 755.8943243 | 385.8892235 |
| DALYs | Australasia | Both | Age-standardized | Depressive disorders | Rate | 2014 | 826.2780207 | 1138.359794 | 570.7804712 |
| DALYs | Eastern Sub-Saharan Africa | Both | Age-standardized | Depressive disorders | Rate | 2019 | 876.5048337 | 1178.687772 | 602.7247171 |
| DALYs | South Asia | Both | Age-standardized | Depressive disorders | Rate | 2020 | 777.7020872 | 1048.802085 | 542.1944789 |
| DALYs | Oceania | Both | Age-standardized | Depressive disorders | Rate | 2015 | 478.1193189 | 651.4504241 | 331.2260449 |
| DALYs | Western Europe | Both | Age-standardized | Depressive disorders | Rate | 2021 | 858.1624201 | 1164.615743 | 600.3664055 |
| DALYs | Andean Latin America | Both | Age-standardized | Depressive disorders | Rate | 2014 | 447.8043065 | 609.2086208 | 306.0809057 |
| DALYs | North Africa and Middle East | Both | Age-standardized | Depressive disorders | Rate | 2020 | 917.2222619 | 1266.094673 | 609.3444866 |
| DALYs | Central Asia | Both | Age-standardized | Depressive disorders | Rate | 2020 | 626.6650711 | 864.6995534 | 427.524876 |
| DALYs | Southern Sub-Saharan Africa | Both | Age-standardized | Depressive disorders | Rate | 2014 | 717.2805654 | 966.7662099 | 501.9735099 |
| DALYs | Eastern Europe | Both | Age-standardized | Depressive disorders | Rate | 2020 | 664.4846061 | 906.1877735 | 461.093473 |
| DALYs | Global | Both | Age-standardized | Depressive disorders | Rate | 2014 | 588.4851352 | 802.1754131 | 412.7355005 |
| DALYs | South Asia | Both | Age-standardized | Depressive disorders | Rate | 2021 | 777.7950463 | 1049.654105 | 542.5608142 |
| DALYs | Southeast Asia | Both | Age-standardized | Depressive disorders | Rate | 2020 | 435.5185387 | 591.4732927 | 301.3880291 |
| DALYs | Caribbean | Both | Age-standardized | Depressive disorders | Rate | 2020 | 713.990906 | 1007.624369 | 486.7722352 |
| DALYs | Eastern Sub-Saharan Africa | Both | Age-standardized | Depressive disorders | Rate | 2020 | 947.3608335 | 1270.40276 | 647.7721197 |
| DALYs | Eastern Europe | Both | Age-standardized | Depressive disorders | Rate | 2021 | 735.8342749 | 1005.599558 | 510.5775931 |
| DALYs | Caribbean | Both | Age-standardized | Depressive disorders | Rate | 2021 | 737.8083433 | 1028.878141 | 507.6419274 |
| DALYs | Southern Latin America | Both | Age-standardized | Depressive disorders | Rate | 2014 | 531.2166605 | 713.3893655 | 366.9254271 |
| DALYs | Central Asia | Both | Age-standardized | Depressive disorders | Rate | 2021 | 644.2781469 | 883.5587875 | 441.8339362 |
| DALYs | Central Europe | Both | Age-standardized | Depressive disorders | Rate | 2021 | 521.7481851 | 710.0964912 | 358.8027634 |
| DALYs | Western Sub-Saharan Africa | Both | Age-standardized | Depressive disorders | Rate | 2020 | 757.3831223 | 1026.431007 | 519.8080305 |
| DALYs | Central Sub-Saharan Africa | Both | Age-standardized | Depressive disorders | Rate | 2014 | 1050.659442 | 1434.698632 | 715.3214169 |
| DALYs | North Africa and Middle East | Both | Age-standardized | Depressive disorders | Rate | 2021 | 900.6785672 | 1242.727109 | 598.4567371 |
| DALYs | Australasia | Both | Age-standardized | Depressive disorders | Rate | 2015 | 820.5277061 | 1123.528966 | 569.4189479 |
| DALYs | Central Sub-Saharan Africa | Both | Age-standardized | Depressive disorders | Rate | 2015 | 1051.598573 | 1431.772475 | 715.1856517 |
| DALYs | Eastern Sub-Saharan Africa | Both | Age-standardized | Depressive disorders | Rate | 2021 | 974.6488995 | 1308.671338 | 668.6723228 |
| DALYs | Global | Both | Age-standardized | Depressive disorders | Rate | 2015 | 589.7894479 | 803.6718854 | 413.661004 |
| DALYs | Southern Latin America | Both | Age-standardized | Depressive disorders | Rate | 2015 | 531.3200406 | 715.2449486 | 368.5290699 |
| DALYs | Southeast Asia | Both | Age-standardized | Depressive disorders | Rate | 2021 | 467.6284287 | 634.8758512 | 323.7427426 |
| DALYs | Western Sub-Saharan Africa | Both | Age-standardized | Depressive disorders | Rate | 2021 | 736.5297464 | 1002.773451 | 502.6058301 |
| DALYs | Oceania | Both | Age-standardized | Depressive disorders | Rate | 2016 | 478.2652368 | 654.8437212 | 330.7835969 |
| DALYs | Andean Latin America | Both | Age-standardized | Depressive disorders | Rate | 2015 | 447.8865552 | 610.7898823 | 303.9151506 |
| DALYs | Tropical Latin America | Both | Age-standardized | Depressive disorders | Rate | 2014 | 641.5780494 | 861.0924752 | 457.7491781 |
| DALYs | East Asia | Both | Age-standardized | Depressive disorders | Rate | 2015 | 434.6041755 | 585.7828764 | 307.1507984 |
| DALYs | Southern Sub-Saharan Africa | Both | Age-standardized | Depressive disorders | Rate | 2015 | 719.8318103 | 972.7420095 | 505.4924847 |
| DALYs | Tropical Latin America | Both | Age-standardized | Depressive disorders | Rate | 2015 | 637.6799036 | 857.2423904 | 454.29036 |
| DALYs | Central Latin America | Both | Age-standardized | Depressive disorders | Rate | 2015 | 567.9564325 | 777.8900101 | 394.6734052 |
| DALYs | High-income Asia Pacific | Both | Age-standardized | Depressive disorders | Rate | 2015 | 383.633137 | 522.3072825 | 267.518068 |
| DALYs | Australasia | Both | Age-standardized | Depressive disorders | Rate | 2016 | 813.3846175 | 1106.680398 | 563.9815876 |
| DALYs | High-income North America | Both | Age-standardized | Depressive disorders | Rate | 2015 | 777.3185913 | 1052.152948 | 548.565812 |
| DALYs | Southern Sub-Saharan Africa | Both | Age-standardized | Depressive disorders | Rate | 2016 | 720.8505239 | 977.928011 | 502.2806962 |
| DALYs | Central Sub-Saharan Africa | Both | Age-standardized | Depressive disorders | Rate | 2016 | 1052.789145 | 1438.806182 | 714.6756317 |
| DALYs | Andean Latin America | Both | Age-standardized | Depressive disorders | Rate | 2016 | 448.3028645 | 610.531415 | 303.774409 |
| DALYs | Australasia | Both | Age-standardized | Depressive disorders | Rate | 2017 | 804.0754268 | 1092.564631 | 556.7709731 |
| DALYs | Southern Latin America | Both | Age-standardized | Depressive disorders | Rate | 2016 | 530.556463 | 713.8983911 | 366.452292 |
| DALYs | Global | Both | Age-standardized | Depressive disorders | Rate | 2016 | 590.8444959 | 805.0395109 | 415.2112082 |
| DALYs | Central Sub-Saharan Africa | Both | Age-standardized | Depressive disorders | Rate | 2017 | 1054.339627 | 1442.191526 | 716.6632869 |
| DALYs | Southern Sub-Saharan Africa | Both | Age-standardized | Depressive disorders | Rate | 2017 | 722.0504787 | 979.5547749 | 503.9236633 |
| DALYs | Southern Latin America | Both | Age-standardized | Depressive disorders | Rate | 2017 | 528.530052 | 712.1392555 | 367.6588453 |
| DALYs | East Asia | Both | Age-standardized | Depressive disorders | Rate | 2016 | 433.9456387 | 583.8997735 | 306.6168126 |
| DALYs | High-income Asia Pacific | Both | Age-standardized | Depressive disorders | Rate | 2016 | 383.2622067 | 521.6220113 | 266.1666523 |
| DALYs | High-income North America | Both | Age-standardized | Depressive disorders | Rate | 2016 | 775.4644118 | 1051.210553 | 548.2725607 |
| DALYs | Andean Latin America | Both | Age-standardized | Depressive disorders | Rate | 2017 | 448.3857749 | 609.6484125 | 304.5844333 |
| DALYs | Global | Both | Age-standardized | Depressive disorders | Rate | 2017 | 591.7351454 | 807.096069 | 415.7370376 |
| DALYs | Central Latin America | Both | Age-standardized | Depressive disorders | Rate | 2016 | 568.7388534 | 778.26051 | 394.895902 |
| DALYs | Tropical Latin America | Both | Age-standardized | Depressive disorders | Rate | 2016 | 634.4767886 | 853.8690241 | 452.0248079 |
| DALYs | Tropical Latin America | Both | Age-standardized | Depressive disorders | Rate | 2017 | 631.3386072 | 848.506395 | 451.5165152 |
| DALYs | Oceania | Both | Age-standardized | Depressive disorders | Rate | 2017 | 478.192848 | 658.2489444 | 330.7900581 |
| DALYs | East Asia | Both | Age-standardized | Depressive disorders | Rate | 2017 | 432.0825867 | 581.8929815 | 305.9043732 |
| DALYs | High-income Asia Pacific | Both | Age-standardized | Depressive disorders | Rate | 2017 | 382.4624056 | 520.9481682 | 265.9147542 |
| DALYs | Southern Latin America | Both | Age-standardized | Depressive disorders | Rate | 2018 | 526.7450767 | 712.7080881 | 363.6113871 |
| DALYs | High-income North America | Both | Age-standardized | Depressive disorders | Rate | 2017 | 774.0647085 | 1049.989745 | 548.5721887 |
| DALYs | Southern Sub-Saharan Africa | Both | Age-standardized | Depressive disorders | Rate | 2018 | 723.6926284 | 985.0422837 | 503.1897302 |
| DALYs | Andean Latin America | Both | Age-standardized | Depressive disorders | Rate | 2018 | 448.4246885 | 604.1171442 | 305.5323693 |
| DALYs | Central Sub-Saharan Africa | Both | Age-standardized | Depressive disorders | Rate | 2018 | 1055.860439 | 1444.012396 | 715.8496967 |
| DALYs | Global | Both | Age-standardized | Depressive disorders | Rate | 2018 | 592.5246007 | 809.2049717 | 414.2360846 |
| DALYs | Central Latin America | Both | Age-standardized | Depressive disorders | Rate | 2017 | 569.7463774 | 777.9199593 | 395.4225858 |
| DALYs | East Asia | Both | Age-standardized | Depressive disorders | Rate | 2018 | 429.9888552 | 579.5513115 | 304.2329524 |
| DALYs | High-income North America | Both | Age-standardized | Depressive disorders | Rate | 2018 | 773.8973596 | 1050.717373 | 546.7533279 |
| DALYs | Global | Both | Age-standardized | Depressive disorders | Rate | 2019 | 593.4992938 | 810.0670049 | 413.3381056 |
| DALYs | Tropical Latin America | Both | Age-standardized | Depressive disorders | Rate | 2019 | 627.3668803 | 839.8826405 | 447.8829486 |
| DALYs | High-income Asia Pacific | Both | Age-standardized | Depressive disorders | Rate | 2018 | 381.9082129 | 520.4151448 | 265.2684494 |
| DALYs | Tropical Latin America | Both | Age-standardized | Depressive disorders | Rate | 2018 | 628.7997031 | 841.8493003 | 450.1947363 |
| DALYs | East Asia | Both | Age-standardized | Depressive disorders | Rate | 2019 | 428.6605528 | 579.0278047 | 302.7669068 |
| DALYs | High-income North America | Both | Age-standardized | Depressive disorders | Rate | 2019 | 775.3084888 | 1052.248652 | 548.5449075 |
| DALYs | Central Latin America | Both | Age-standardized | Depressive disorders | Rate | 2019 | 570.9104715 | 777.2520569 | 395.503663 |
| DALYs | Central Latin America | Both | Age-standardized | Depressive disorders | Rate | 2018 | 570.7667725 | 777.7181218 | 396.2231683 |
| DALYs | High-income Asia Pacific | Both | Age-standardized | Depressive disorders | Rate | 2019 | 382.1633602 | 520.6142415 | 265.1975348 |
| DALYs | Oceania | Both | Age-standardized | Depressive disorders | Rate | 2019 | 478.6023965 | 650.9710329 | 330.4303889 |
| DALYs | Oceania | Both | Age-standardized | Depressive disorders | Rate | 2018 | 478.3614868 | 653.6529364 | 329.1427463 |
| DALYs | Australasia | Both | Age-standardized | Depressive disorders | Rate | 2018 | 795.1986026 | 1087.355417 | 550.5100248 |
| DALYs | East Asia | Both | Age-standardized | Depressive disorders | Rate | 2020 | 440.7955722 | 598.7708296 | 313.5018239 |
| DALYs | High-income North America | Both | Age-standardized | Depressive disorders | Rate | 2020 | 982.0452408 | 1322.834766 | 689.0538537 |
| DALYs | Central Latin America | Both | Age-standardized | Depressive disorders | Rate | 2020 | 699.3568049 | 956.6093886 | 479.6046817 |
| DALYs | High-income Asia Pacific | Both | Age-standardized | Depressive disorders | Rate | 2020 | 422.637584 | 577.6971725 | 291.106672 |
| DALYs | Australasia | Both | Age-standardized | Depressive disorders | Rate | 2019 | 790.0770761 | 1076.504935 | 545.3624772 |
| DALYs | Southern Sub-Saharan Africa | Both | Age-standardized | Depressive disorders | Rate | 2019 | 724.6577851 | 985.134838 | 504.7691524 |
| DALYs | Andean Latin America | Both | Age-standardized | Depressive disorders | Rate | 2019 | 448.0086314 | 608.8465032 | 303.7603941 |
| DALYs | Southern Latin America | Both | Age-standardized | Depressive disorders | Rate | 2019 | 525.604217 | 711.745647 | 361.5792235 |
| DALYs | Oceania | Both | Age-standardized | Depressive disorders | Rate | 2021 | 507.9046168 | 704.0350686 | 329.2026797 |
| DALYs | Oceania | Both | Age-standardized | Depressive disorders | Rate | 2020 | 482.4442643 | 672.7277123 | 318.6162747 |
| DALYs | Australasia | Both | Age-standardized | Depressive disorders | Rate | 2020 | 854.6389044 | 1202.591827 | 578.1958429 |
| DALYs | Central Sub-Saharan Africa | Both | Age-standardized | Depressive disorders | Rate | 2019 | 1056.317509 | 1447.288482 | 717.9882485 |
| DALYs | Andean Latin America | Both | Age-standardized | Depressive disorders | Rate | 2020 | 607.2055345 | 842.0798469 | 401.5164702 |
| DALYs | Australasia | Both | Age-standardized | Depressive disorders | Rate | 2021 | 849.3108817 | 1201.644152 | 569.1345866 |
| DALYs | Southern Sub-Saharan Africa | Both | Age-standardized | Depressive disorders | Rate | 2021 | 880.6939773 | 1219.629846 | 609.0909378 |
| DALYs | Southern Latin America | Both | Age-standardized | Depressive disorders | Rate | 2020 | 659.4167884 | 920.5730104 | 440.4649474 |
| DALYs | Tropical Latin America | Both | Age-standardized | Depressive disorders | Rate | 2020 | 739.3483708 | 996.8616694 | 523.3749884 |
| DALYs | Southern Sub-Saharan Africa | Both | Age-standardized | Depressive disorders | Rate | 2020 | 845.5305714 | 1164.063988 | 583.8387853 |
| DALYs | Central Sub-Saharan Africa | Both | Age-standardized | Depressive disorders | Rate | 2020 | 1172.249729 | 1618.408395 | 782.0795131 |
| DALYs | Southern Latin America | Both | Age-standardized | Depressive disorders | Rate | 2021 | 652.6160483 | 904.8159892 | 439.6207463 |
| DALYs | Central Sub-Saharan Africa | Both | Age-standardized | Depressive disorders | Rate | 2021 | 1136.909012 | 1588.383818 | 756.8827797 |
| DALYs | Global | Both | Age-standardized | Depressive disorders | Rate | 2020 | 676.7556371 | 919.1507338 | 471.8171266 |
| DALYs | Andean Latin America | Both | Age-standardized | Depressive disorders | Rate | 2021 | 578.7370473 | 804.9708557 | 389.3150346 |
| DALYs | Tropical Latin America | Both | Age-standardized | Depressive disorders | Rate | 2021 | 780.1498801 | 1062.15175 | 539.0607293 |
| DALYs | Global | Both | Age-standardized | Depressive disorders | Rate | 2021 | 681.1421969 | 923.8253658 | 475.1893022 |
| DALYs | East Asia | Both | Age-standardized | Depressive disorders | Rate | 2021 | 429.6691133 | 585.4152713 | 304.2511206 |
| DALYs | High-income North America | Both | Age-standardized | Depressive disorders | Rate | 2021 | 982.776437 | 1322.412345 | 685.3341279 |
| DALYs | High-income Asia Pacific | Both | Age-standardized | Depressive disorders | Rate | 2021 | 447.8641967 | 606.649809 | 307.9948624 |
| DALYs | Central Latin America | Both | Age-standardized | Depressive disorders | Rate | 2021 | 682.9349609 | 935.0172863 | 468.2830689 |
